# Storytelling as a Research Tool Used to Explore Insights and as an Intervention in Public Health: A Systematic Narrative Review

**DOI:** 10.3389/ijph.2021.1604262

**Published:** 2021-11-02

**Authors:** Becky McCall, Laura Shallcross, Michael Wilson, Chris Fuller, Andrew Hayward

**Affiliations:** ^1^ Institute of Health, University College London, London, United Kingdom; ^2^ School of Design and Creative Arts, Loughborough University, Loughborough, United Kingdom

**Keywords:** Public Health, intervention, HIV, storytelling, insight, methodological tool, review

## Abstract

**Objectives:** Studies of storytelling (ST) used as a research tool to extract information and/or as an intervention to effect change in the public knowledge, attitudes, and behavior/practice (KAB/P) were sought and analyzed.

**Methods:** Medline, EMBASE, PsycINFO, ERIC, Web of Science, Art and Humanities database, Scopus, and Google Scholar were searched, and a basic and broad quantitative analysis was performed, followed by an in-depth narrative synthesis of studies on carefully selected topics.

**Results:** From this search, 3,077 studies were identified. 145 studies entered quantitative analysis [cancer and cancer screening (32/145), HIV (32/145), mental health (10/145), vaccination (8/145), and climate change (3/145)]. Ten studies entered final analysis [HIV/AIDs (5), climate change (1), sexual health (3), and croup (1)]. ST techniques included digital ST (DST), written ST, verbal ST, and use of professional writers. Of the ten studies, seven used ST to change KAB/P; the remainder used ST to extract insights. Follow-up and evaluation were very limited.

**Conclusion:** ST reveals insights and serves as an intervention in public health. Benefits of ST largely outweigh the limitations, but more follow-up/evaluation is needed. ST should play a more significant role in tackling public health issues.

**PROSPERO registration number:** CRD42019124704

## Introduction

The properties of storytelling (ST) are such that a personal experience, told with nuanced detail, resonates with the listener and may even validate their own experiences, helping them and the teller make sense both retrospectively and prospectively of real-life events [1].

Back in 1964, phenomenologist Maurice Merleau Ponty wrote that the making of stories, “reveals things to us that we know but didn’t know we knew”, suggesting that ST provides access to a richness of information not necessarily available *via* other means [2].

Increasingly, ST is used as a methodological tool in research, including that of health and social science [3–6]. It is likely that many incidences of ST in health research remain undocumented for reasons of confidentiality.

In many contexts, storytelling might be considered as lying outside the arena of entertainment. Lugmayr et al refer to such “serious storytelling” as narration that “progresses as a sequence of patterns impressive in quality, relates to a serious context, and is a matter of thoughtful process.” [7] ST with purpose beyond entertainment is one focus of this review, that is, ST as a research tool in public health.

It also addresses both the development of a story and the process of telling it. Many ST studies have employed narrative-as-message (story as text without detail on the process of telling) but few have explored how people identify, craft, and tell their personal stories. Fewer still are studies on the assessment of ST as a tool for change in knowledge, attitude and, behavior/practice (KAB/P).

ST formats vary and include verbal, written, “photovoice” (use of photos as prompts), or, increasingly, digital storytelling (DST), which captures lived personal experiences through the creation of 3–5-min digital films.

DST may manifest as digital video, gaming, Instagram stories (or “Instastories”), or interactive television as examples. Within the boundaries of this review, DST primarily refers to the production of a short (3–4 min) digital video where participants craft personal experiences, possibly using photo, music, and voice recordings [8].

As a research tool, ST generates more nuanced, contextualized, and culturally-reflective information than some other qualitative research methods, e.g., interview, but it can also be used as an intervention to facilitate change in KAB/P. In this respect, the transformative properties of ST can be conceived as relating to the stimulation of human emotions to engage with new knowledge and, as such, influence attitude and ultimately behavior, as illustrated by Wong J. P. et al in the prevention of HIV/sexually transmitted infections (STIs). ST contextualizes health-related information to one’s own situation, potentially encouraging beneficial behavioral change [9].

Conventionally, social and health research largely uses quantitative cross-sectional surveys to measure change in KAB/P, while most qualitative work exploring KAB/P involves interviews and/or focus groups. For example, with antimicrobial resistance (AMR), most research on the KAB/P adopts health behavior surveys [10], interviews, and focus groups [11, 12]. The researchers are particularly interested in AMR and intend to use ST as a future research tool on the topic. No studies were identified using ST to explore AMR.

ST largely remains an emergent research method and its validity has not been established. However, this does not justify dismissing the potential value of ST in this capacity, and this review aimed to identify evidence of the validity of ST as used in the research context whether to gather information and/or used as an intervention to change KAB/P.

In summary, this systematic review aimed to, firstly, conduct a basic quantitative evaluation of the distribution of peer-reviewed, published studies that use ST as a research tool across a broad range of public health issues and, secondly, to qualitatively analyze 10 carefully selected studies that use ST as 1) a research tool to gain insight into KAB/P, and/or 2) an intervention to effect change. Essentially, the public health issues selected for final analysis were defined as bearing a personal cost in the immediate term but potentially providing a population-level health benefit in the long-term, for example, vaccination or AMR (foregoing an antibiotic in the short-term to help prevent the development of resistance at a population level in the long-term). The review was conducted using the process of narrative synthesis and was designed to inform future research using ST in AMR.

## Methods

### Sources

Peer-reviewed and published studies were identified from eight major databases: Medline, Embase, PsycINFO, ERIC (Proquest), Web of Science, Art and Humanities database (ProQuest), Scopus, and Google Scholar. Two librarians aided formulation of the search strategies. Key words and synonyms can be found in Table 1.

**TABLE 1:** Search terms comprised of concepts and synonyms (broadly based on PICO), United Kingdom, 2021.

PICO (adapted from)	Intervention	Context (comparator)	Outcome
Concepts	Storytelling	Public health	Attitude*
Synonyms	Story	Health information	Knowledge
	Stories	Health education	Behaviour*
	Narrative*	Health communication	Perception*
			Misperception*
			Misinformation*
	Narration	Health behaviour*	Belief*
		Screening	Value*
		HIV or HIV/AIDS	Perception*
		Vaccination or vaccine*	
		“Climate change”	
		Cancer	
		Obesity or overweight	
		Smoking or “smoking cessation”	
		“Sexual health” or “sexually transmitted infection*” or STI* or STD*	
		“Antimicrobial resistance” or “AMR” or “antibiotic resistance” Antimicrobi* or antibiotic*	
		Resistan*	
		“Drug resistan*”	

### Study Selection and Screening

Endnote reference management software was used to organize studies and systematic review management software (SUMARI) [13] aided initial title and abstract screening (BM and CF). Full texts were reviewed and reference lists checked (BM and CF). The recommendations of the Preferred Reporting Items for Systematic Review and Meta-Analysis Protocols (PRISMA-P) were followed.

### Inclusion Criteria

The stage 1, basic, quantitative analysis included all studies that met the following inclusion criteria: qualitative, quantitative, or mixed-method, peer-reviewed, primary public health studies using ST within the context of research and in the English language; publication 1990–present; all ages and demographic backgrounds; and ST (verbal or written or digital) used as a research tool to understand and/or effect change in KAB/P.

The stage 2, qualitative analysis included studies that primarily addressed public health topics involving a personal cost in the immediate term but population-level health benefit in the longer term e.g., HIV involves individual sacrifice of testing in the short-term but reduced HIV population-level spread in the long-term. This criterion parallels the nature of AMR–an area of research interest to the authors. Other inclusion criteria are in Supplementary File S1.

Definitions used in screening and selection were: story–a story derived from interviews, role model stories, or narrative accounts; storytelling–DST, “Photovoice”, plays, theatre, or film, usually involves the crafting and telling of a story; and personal narrative–personal experience not formatted as a story.

### Exclusion Criteria

Studies where health was a secondary consideration, or subject matter was more clinical than public health, were excluded.

### Critical Appraisal of Selected Studies

Critical appraisal of included studies used the 16-item QATSDD tool for qualitative, quantitative, and mixed-methods studies [14]. This provides a score (0 = very poor, 3 = very good) for each criteria in each study, and an overall score.

### Data Extraction and Synthesis

JBI SUMARI [13] software helped data extraction from the 145 studies in the quantitative analysis, and the 10 final studies (Supplementary Files S2A, S2B respectively). The latter included phenomena of interest, overall design/methods (using ST) and analysis, location, setting including context/culture, participant characteristics, sample size, and description of main results.

Data synthesis used the narrative synthesis approach developed by the Economic and Social Research Council (ESRC) [15], comprising an initial description of study results describing patterns observed (preliminary synthesis), followed by an exploration of relationships within and between studies, as well as interpretations and possible explanations (narrative synthesis).

### Outcomes

The primary outcome was the narrative synthesis review of the 10 final studies. Secondary outcomes were drawn from the 145 studies for quantitative review and included, for example, frequency of the public health topics by storytelling method, the geographical spread stratified by topic, and study dates.

## Results

The initial search for studies using stories, ST, or personal narrative in the public health arena provided a total of 3,077 studies, after removal of duplicates. All of these were title and abstract screened (necessary for sufficient information), and 2,852 were removed due to the following (Supplementary File S3: Reasons for exclusion): being off topic (1072), having an unsuitable method (439), subject matter a poor fit for the inclusion criteria (1,073), study paper unobtainable or a conference abstract only (13) not a primary study (160), or topic not associated with short-term personal sacrifice and long-term population level gain (95). Full text review involved 216 studies.

**FIGURE 1:**
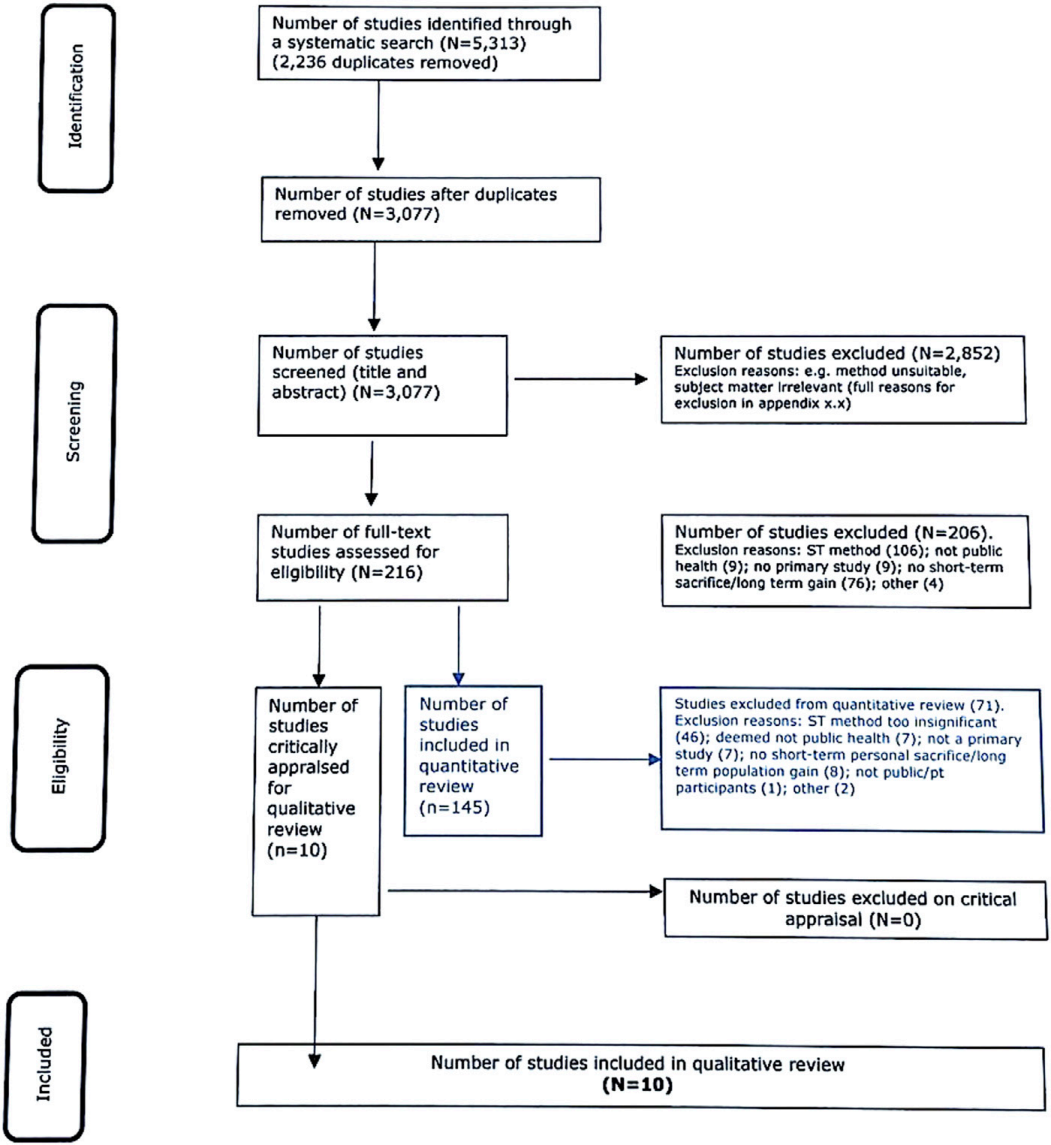
PRISMA flow chart representing selection process for the use of storytelling as a tool to extract information or as an intervention across a range of public health issues. Studies (145) in the quantitative analysis are in blue, while those [10] in the final analysis are in black. United Kingdom, 2021.

Insert: Figure 1: PRISMA flow chart representing selection process for the use of storytelling as a tool to extract information or as an intervention across a range of public health issues. Studies (145) in the quantitative analysis are in blue, while those (10) in the final analysis are in black. United Kingdom, 2021.

### Critical Appraisal of Studies in the Final Analysis

Critical appraisal of the 10 studies included for final analysis was challenging due to the heterogeneity of methods and the subjectivity of the qualitative content (8/10 studies contained qualitative data only; 2/10 contained qualitative and quantitative data). See Supplementary Files S4A, S4B for critical appraisal scores.

Each study was scored according to the relevant criteria. Qualitative studies were assessed using 13/16 total criteria, while mixed-method studies used 16/16 criteria. Qualitative only (8/10 studies) generated an overall score of 64%, and mixed method (2/10 studies) generated the highest overall score of 70%. The overall score for all studies combined reflected a weighting towards qualitative data at 66% that drew strength from detailed descriptions of context and setting and articulated thematic descriptions and interpretations.

### Study Characteristics

#### Quantitative Analysis

Of 145 studies identified, ST comprised 80/145 (54.8%), story 44/145 (30.1%), and personal narrative 21/145 (14.4%) as research tools to address a total of 16 public health topics.

Cancer/cancer screening comprised most studies 32/145 (22.1%) matched by HIV 32/145 (22.1%); the remaining studies focused on mental health 10/145 (6.8%), cardiovascular health 8/145 (5.5%), and vaccination 8/145 (5.5%). No studies referred to AMR.

Analysis by study location showed that 69/145 (47.6%) were based in the United States (US), 22/145 (152%) in Africa, 20/145 (13.8%) in Canada, 18/145 (12.4%) in Europe, 9/145 (6.2%) in Australasia, and only 4/145 (2.8%) in the United Kingdom (UK).

Study dates showed a leap in the number of studies from 8 in 2001–2005, to 26 in 2006–2010, and then doubling to 57 studies in 2011–2015 and 49 in 2016–2019 see Table 2 for a breakdown of quantitative findings.

**TABLE 2:** Number of studies by public health topic, geographical region, and dates of studies, United Kingdom, 2021.

(A) Study count by public health topic.	
Public health topic	Count of Public health topic (%)
Alcohol and drug abuse	1 (0.7%)
Cancer	32 (22.1%)
Cardiovascular health	8 (5.5%)
Chronic disease	1 (0.7%)
Climate change	3 (2.1%)
Diabetes	7 (4.8%)
HIV	32 (22.1%)
Mental health	10 (6.9%)
Nutrition	4 (2.8%)
Obesity	6 (4.1%)
Other	19 (13.1%)
Physical activity	2 (1.4%)
Rare disease	1 (0.7%)
Reproductive and sexual health	7 (4.8%)
Smoking	4 (2.8%)

(B) Study count by geographical region and public health topic.	
Geographic region and public health topic	Count of studies by region and topic
Africa	22 (15.2%)
HIV	17
Other	2
Reproductive and sexual health	2
Smoking	1
Asia	3 (2.1%)
Mental health	1
Other	1
Vaccination	1
Australasia	9 (6.2%)
Chronic disease	1
HIV	1
Mental health	3
Nutrition	1
Other	2
Smoking	1
Canada	20 (13.8%)
Cancer	3
Climate change	3
Diabetes	1
HIV	5
Obesity	1
Other	4
Physical activity	2
Smoking	1
Europe	18 (12.4%)
Cancer	4
Diabetes	2
Mental health	2
Obesity	2
Other	4
Rare disease	1
Reproductive and sexual health	1
Smoking	1
Vaccination	1
South America	1 (0.7%)
Cancer	1
United Kingdom	3 (2.1%)
Mental health	2
Other	1
HIV	1
US	69 (47.6%)
Alcohol and drug abuse	1
Cancer	24
Cardiovascular health	8
Diabetes	4
HIV	8
Mental health	2
Nutrition	3
Obesity	3
Other	5
Reproductive and sexual health	4
Vaccination	6
Grand Total	145

(C) Dates of studies in 5-years brackets.	
Row labels	Count of 5-years date bracket
1996–2000	3
2001–2005	8
2006–2010	26
2011–2015	57
2016–2020	49
2021–2025	2

*United Kingdom separated from Europe because it is a country of special interest to the authors.

#### Final Qualitative Analysis of 10 Storytelling Studies

##### Study Characteristics

The 10 studies included in the final analysis all used ST as a qualitative research method. Two papers referred to one study, but were distinct enough to be counted as two studies [16, 17]. Use of ST was heterogeneous: as an intervention to effect change in 7/10 studies [18–22], and as a means to extract information (3/10) [23–25]. Topics included HIV (5/10 studies) [18, 20, 22, 23, 25], sexual and reproductive health (3/10) [16, 17, 24], climate change (1/10) [21], and emergency care of croup (1/10) [19]. Of the studies, three out of ten used DST [16, 17, 25], two out of ten employed a professional storyteller to craft and perform participants’ stories [19, 22], two employed ST by written word [21, 24], and three used verbal ST [18, 20, 23]. Two out of ten studies used mixed methods using both quantitative and qualitative data [16, 17] while eight primarily used qualitative data only [18–25]. The locations used were Uganda (1/10) [24], the US (3/10); [16, 17, 20], Canada (3/10) [19, 21, 23], and South Africa (3/10) [18, 22, 25]. Participant numbers ranged from six to 200, with most involving around 25–30 participants. See Table 3 for key characteristics of the 10 studies and Supplementary File S5 for the list of studies for final analysis.

**TABLE 3:** Key characteristics of 10 studies included for final analysis. United Kingdom, 2021.

Author/title/year	Population studied and objective/s	Outputs/outcomes/results	Take-away (value of storytelling as shown by study)
DiFulvio GT, Gubrium AC, et al. Digital Storytelling as a Narrative Health Promotion Process: Evaluation of a Pilot Study. *Int Q Community Health Educ*. 2016; 36(3): 157−164	Puerto Rican, socially deprived women of 15−21 years. Pregnant, parents, or non-parents	Quantitative: At 3 months, participants (ppts) experienced greater positive social interactions (p=0.076); “Instrumentality” (defined as “attitude towards enjoying physical sex”) lowered (*p* = 0.097). Optimism for future increased immediately after workshop but not at 3 months (*p* = 0.093)	One of few studies to assess benefits of the DST process for ppts: created positive feelings associated with telling one’s own story in a group, and having their voices heard. Relevance to marginalized, specific and culturally-defined groups who benefit most from ST
	To explore the process of DST as an intervention to encourage positive change in KAB/P towards sexual health, inc. self-esteem, social support, and control of future Quantitative scales and qualitative data		
	Extracted info and intervention	Qualitative: ST led to improvements in: Truth telling - ppts told their own story rather than being the subject of someone else’s story. Affective impact*-* moved emotionally by listening to others’ stories, and telling and having their stories listened to. Empowerment - reported feeling confident, strong and respected, optimistic, accomplished, and able to speak up after writing their stories	Qualitative results of storytelling evaluation were more revealing than quantitative findings. Sample size (29 stories) too small for meaningful, statistically significant quantitative findings. (see Gubrium below for qualitative findings)
	Evaluated		
Gubrium AC, Fiddian-Green A, Lowe S, DiFulvio G, Del Toro-Mejías L. Measuring Down: Evaluating Digital Storytelling as a Process for Narrative Health Promotion. *Qual Health Res*. 2016; 26(13): 1787−1801.	Same population as above (a different analysis of same study)	Qualitative: Truth telling (as above). Cathartic and therapeutic*:* through the crafting of a digital story that voices authentic experience. Sense making: reflected on personal and social memories, writing made sense of their lived experiences, instilled a sense of control over health and experiences to formulate goals for the future. Solidarity: ppts feel connected to others and less socially isolated in their experiences. Social support: space for acceptance, overcoming fear of social stigma. Empathy: opportunity to listen to others’ stories, encouraging empathy and understanding	The variety of qualitative data sources including workshop transcripts, follow-up interviews and ppt evaluations together instilled a sense of trustworthiness (validity) in the data
	Aimed to assess the process of ppts making their own digital stories. This analysis “measured down” to reflect the locally grounded, felt experiences of ppts who engage in the process, as current quantitative scales do not “measure up” to accurately capture these effects		
	Extracted info and intervention		Qualitative findings: “present a broader picture of the benefits of the DST process, substantiating claim that the DST process positively affects participants and has tremendous potential as a mechanism for health promotion”
	Evaluated		
Hartling L, Scott S, Pandya R, Johnson D, Bishop T, Klassen TP. Storytelling as a communication tool for health consumers: development of an intervention for parents of children with croup. Stories to communicate health information. *BMC Pediatr*. 2010; 10:64. Published 2010 Sep 2	Parents of children admitted to emergency department (ED) with croup	Parents valued the writer’s ability to capture the parents’ emotions. Reviewers (one medic and one English professor) considered the stories an excellent potential source of medical advice for parents	Take-aways relate to story development. Challenges identified staying true to the story versus being evidence based especially for this “medical” text; balancing comprehensive content and wider application while being succinct. This study deviates from phenomenological idea (the way the world appears to the person experiencing the world) of accepting each personal story on its own merits without modifying
	To use ppt stories in the development of a booklet for parents (and children)	Conflict between ST and authenticity based on truthful experiences vs. correct medical knowledge	Story length, reading level, representation of different demographics and illness experiences, graphics and layout all important. The process also showed temptation to modify a story if the story does not quite fit the end purpose (is diversion from authenticity that might be justified depending on end goal)
	After interviewing parents in the ED, a writer wrote a typical story (based on real experiences) for the booklet. Booklet aimed to provide info and reduce anxieties around such an ED visit. Focus groups discussed story development and general perceptions of stories; content and emotional by-products of the stories; graphics etc.		
	Extracted info and intervention	Used illustrations broadened appeal to kids and parents (practical tool for explaining to the child what may happen in the ED) Ease of relating to characters important	
	Evaluated		
(Not part of final analysis because effectively an extension of the above study adding evaluation of the story booklet use in the ED.) Hartling L, Scott SD, Johnson DW, Bishop T, Klassen TP. A Randomized Controlled Trial of Storytelling as a Communication Tool. PLoS One. 2013; 8(10): 1–11	To compare effectiveness of story booklet compared to standard information sheets for parents of children with croup attending the ED using measures of anxiety between recruitment in ED and discharge, and follow up phone interviews. 205 received standard information booklets, and 208 the story booklet	There was no significant difference in the primary outcome of change in parental anxiety between recruitment and ED discharge (5 points change for story group vs.6 points for comparison gp, *p* = 0.78). Story group showed significantly greater decision regret regarding decision to go to the ED (*p* < 0.001): 6.7% of the story group vs. 1.5% of the comparison group	The finding of greater regret in deciding to attend the ED might be related to parents feeling that they could have managed at home and avoided the trip to the ED after reading the stories
Joshi A. Multiple sexual partners: perceptions of young men in Uganda. *J Health Organ Manag*. 2010; 24(5): 520–527	Teen boys 15-19yrs, in Uganda	Personal stories written, and also wrote and acted out dramas. ST unveiled subtle, often unarticulated influences on ppt’s behavior e.g., ignorance of health risks (often assumed to be the problem) only partly explains risky sexual behavior	ST unpacked the less obvious behavioral reasons and insights on the importance of adhering to the social expectations of gender roles including MSP. ST in this study used proxy characters in the dramas and ST was written to overcome, both measures to overcome inhibitions around a sensitive topic
	To derive and understand perceptions and KABs of regarding their sexual behavior (sex with multiple sexual partners, MSP), and the drivers of MSP, within the context of HIV. Ultimately, aims to inform health promotion in order to reduce HIV incidence in this group		
	Extract info	Ppts believe risky sexual behavior asserts masculinity - this outweighs any health risks. MSP associated with power e.g., the provider, in charge, the decision-maker. Any risks associated with MSP identified by ppts as challenges to their pleasure, and control (not health)	
	No evaluation		
Malena-Chan R. A narrative model for exploring climate change engagement among young community leaders. *Health Promot Chronic Dis Prev Can*. 2019; 39(4): 157–166	Community leaders, Saskatchewan, Canada (age 20–40 years)	ST/sharing helped articulate difficult emotions about climate change and elicits feelings of solidarity. Stumbling blocks were identified e.g. nuanced experiences of agency, responsibility, capacity, and activation, e.g., “I see climate news… and I tell myself “I can’t afford to look at this right now” …avoidance is part self-care but also part unhealthy willful ignorance … Ppts had difficulty identifying the meaning of actions e.g., minimizing emissions or environmentally-friendly food choice in context of hugeness of climate change problem. Stages towards action in narratives identified (1) moving from knowledge of the challenge to a sense of agency about it; (2) from agency to a sense of responsibility to choose to address it; (3) from responsibility to a sense of capacity to produce desirable outcomes despite contextual challenges; and (4) from capacity to a moral sense of activation in context	ST seeks to inform a more compelling story around climate change to present to policymakers. ST identified opportunities for transforming knowledge into emotions that mobilize collective action. ST highlights information deficit approach to climate change engagement may not translate into effective action. Authors suggest ST could be a mechanism for bringing emotions around climate change to the surface and managing feelings of helplessness in face of the huge challenge of climate change
	To capture contextual and cultural barriers to everyday actions in response to climate change in Saskatchewan, Canada. Effectively develops a model to help bridge the gap between knowledge and action, and to inform a framework to overcome barriers to engagement Extracted info and intervention		
	Not evaluated		
Rand JR. Inuit women’s stories of strength: informing Inuit community-based HIV and STI prevention and sexual health promotion programming. Int J Circumpolar Health 2016; 75:32135	Inuit women and families in Canadian Arctic with high prevalence STIs and HIV	ST revealed the right agent/s for any particular community is important: e.g., in the Innuit community, led by elders, HIV/STIs present a relatively new issue so elders cannot advise. ST highlights less overt aspects of daily life e.g., women consider alcohol instrumental in negative sexual health outcomes and should be incorporated into health programs. ST helps identify hard-to-reach groups e.g., youth out of school. ST links the epidemiology with daily lives.	Here, ST highlights the importance of localizing the research e.g. community-based participatory research (CBPR) design; primary researcher was 15 years local resident so familiar with culture, facilitating interpretation of stories (esoteric nature so difficult for an outsider to understand fully). Used Two-eyed seeing theoretical framework (Inuit concept – incorporates multiple world views)
	To source/extract information and perceptions of Inuit women and families on determinants of sexual health and HIV. No change intervention		
	Aims to inform relevant programming and policy		
	Extracted inf		
	Not evaluated		
Treffry-Goatley A, Sykes P, et al. Understanding Specific Contexts of Antiretroviral Therapy Adherence in Rural South Africa: A Thematic Analysis of Digital Stories from a Community with High HIV Prevalence. *PLoS One*. 2016; 11(2): e0148801. Published 2016 Feb 29	HIV/AIDS Black, Zulu-speaking, poverty-stricken, patients in resource-limited settings in sub-Saharan Africa	20 digital stories produced *via* written word and art (drawings and photos). Major themes emerged: the way it used to be, change, family, intimate relationships. Through metaphor, ST provides insight into how culture and history impact meaning e.g. Apartheid and armed struggle - Black resistance seen with Apartheid, referenced in the context of ART, HIV too can be overcome. ART considered as weaponry (common in S. Africa) to protect against HIV. ST revealed issues around fear, stigma, disclosure, side-effects and how adherence intersects with the everyday. ST revealed importance of social support (emotional, financial from family). ST shows reluctance to disclose. ST and stigma: A mother refused to take her pills due discrimination from her children. Some plurality with traditional healers vs. Western medicine	ST unveils multiple layers of influences on adherence KAB/Ps. References that underpin the making of meaning around issues related to adherence to ART - there’s clear evidence of cultural references within this study e.g., weaponry and Apartheid. ST revealed that stigma, disclosure, and traditional healing, were all shown to influence adherence behavior, effectively attesting to the complexity of ART adherence
	To overcome challenges of adherence of antiretroviral therapy (ART) among To use DST to understand specific individual and structural drivers/contexts of adherence to ART. To use CBPR. To thematically analyze stories to identify barriers and facilitators to adherence		Used voices of those affected by events rather than experts (CBPR)
	Extracted info only		Study showed a leaning towards social desirability in ST e.g., avoidance of reference to faith healers because facilitators Western (risk if non-locals run the ST workshops)
	Not evaluated		
Zeelen, J, et al.(2010), “Beyond silence and rumor: Storytelling as an educational tool to reduce the stigma around HIV/AIDS in South Africa”, Health Education, Vol. 110 No. 5, pp. 382–398	Local (Limpopo) communities rural South Africa, high poverty, local dialects, pregnant women attending ante-natal clinics (HIV prevalence 20.7%)	ST on sensitive topics more productive if proxy characters e.g., animals of the region, used to create a sense of removal and encourage openness, overcoming difficulties addressing gender inequality which is a key reason for HIV infection rates increasing. ST revealed coexistence of different belief systems e.g., traditional healers and Western medicine	ST shown as accessible, transparent and contextualized research tool/intervention for local rural communities. Bringing ST to the waiting room/to the target community can be effective. ST as means of access target groups. ST as ‘edutainment’: education enriched with entertainment overcomes access to people frustrated with conventional education. Easy to relate to animal characters as proxy -, overcomes inhibitions of sensitive topic. Value of local ST practitioner for local language, knowledge, social norms (prevalent myths on stigma etc.) e.g., hospital beds spread HIV, also helps to overcome an information deficit model and internalizing of messages
	To use ST as a tool for informal education in 5 health clinics to tackle HIV stigma. To create a conversational space to elicit personal and localized stories about HIV, and ease stigma. To use professional storyteller from the local museum to tell stories Followed by interviews with some audience members, HCPs at clinics to draw on the dialogue stimulated by the ST and elicit personal stories		
	Extracted info only		
	Evaluated		
Leukefeld C, et al., HIV prevention among high-risk and hard-to-reach rural residents. *J Psychoactive Drugs*. 2003; 35(4): 427–434	Probationers with HIV and drug abuse problems in rural Kentucky, US (perception of low risk in rural vs. urban environment)	ST led to self-exploring, personal insights into behavior that sometimes resonate with others	ST is about cause and effect. The ST process in this study created structure *via* thought mapping helping ppts to link cause and consequence. ST related facts and events around HIV risk (sex and drugs). to the rural context
	To use ST intervention called Enhanced Probation Focused Intervention based on transtheoretical model of behavior change with personalized strategies to change thinking around drug use and sexual KAB/P and HIB risk. Compare standard vs. Enhanced version of intervention (tailored to rural issues)	Mutual ST precipitated sense of solidarity and strength	
		Ppts reported high illicit drug use and risky sexual behavior (*via* ST processes). Ppts reported limited KABs on HIV and hepatitis risks	ST draws on power of imagination - ppts tried out stories with different endings i.e., more positive ones offering hope for change
	Extracted info and intervention	Limited to preliminary findings of comparison NIDA (standard government) and Enhanced program	
		Ppts connected actions lead to consequences *via* ST	ST helped ppts understand how to connect cause and effect. ST enabled ppts use own words to develop possible strategies to overcome challenges. ST is used to access high risk individuals in rural setting and obtain information on prevalence and behaviors around HIV risk. Targeting ST at prisoners means results localized to that group (cannot generalize) – but target groups seem to lend to ST
	Not evaluated	(6-month evaluation on effect on ST workshops on ppts was planned but not reported here)	
Dickinson D. Myths, science and stories: working with peer educators to counter HIV/AIDS myths. *Afr J AIDS Res*. 2011; 10 Suppl 1: 335–344	Workplace peer educators in a mining village in South Africa. To use ST to overcome stigma, misunderstandings, and myths around HIV/AIDs and as an alternative to repeating factual, scientific messages (information deficit). ST aimed to explore motivations and unearth ingrained misunderstandings that perpetuate stigma and misinformed myths in the area	Recorded 80 HIV/AIDS myths and used them as content to develop 16 stories *via* 6 workshops. ST identified incorrect information in the myths e.g., and attempted to clarify. Workshops where myth was recounted, a story created and told to counter the myth. Stories often created characters e.g. The Shepherd and behavior around HIV risks was conveyed in metaphorical terms e.g. the shepherd takes a shortcut across the rive – take a quick fix from a faith healer rather than the longer route of seeking out ART. Stories developed illustrated 4 maxims e.g. appearances can be deceptive, or small problems become large ones if ignored. Interviews with 23/28 peer educators on the ST process and effects found some peer educators enjoyed conceiving and telling stories; others clearly found the idea of telling stories daunting and unattractive	ST revealed sources of stigma and misunderstanding around HIV/AIDs in the local area. ST uses the power of real, lived experience to counter HIV/AIDS myths, and the stories created resonate with ppts’ lives. ST can be propagated *via* word of mouth in the same way that myths circulate
	Interviews on impact of ST afterwards		
	Extracted info and intervention		ST impact best where similarity between message source and recipient; breaks down barriers. ST more enjoyable than an information deficit type of delivery. Study revealed that sometimes ST might be optimal tool instead of, or alongside other forms of information delivery. ST is effective way of communicating for some but not all people. Messages *via* ST arguably greater purchase than scientific explanations in this group. Peer educators, using ST, seen as agents of change
	Evaluated		

Key: ST, storytelling; ppt, participant.

### The Process of Storytelling

Considerable heterogeneity in ST methods remained within the 10 final studies. ST involves a process of triggering participant memories and crafting these into a story, e.g., in the climate change study, participants jotted down memories of personal experiences which were incorporated into their story [21]. Thought-mapping (nodes representing feelings, thoughts, and actions with lines between them denoting cause and effect) was a technique used to stimulate thinking around risk-taking behaviors [20].

Proxy characters including cards featuring characters of similar demographic to participants or animals instead of humans were used [22, 24]. South African miners aimed to dispel HIV stigma by gathering 300 local myths and using them to inform stories [18]. Images recorded pivotal moments to aid DST [17].

### Storytelling as Agent of Change or Exploratory Tool

The majority (7/10) of studies served to both extract information and serve as an intervention.

The study exploring young Ugandan men’s attitudes towards multiple sexual partners (MSP) mainly sourced insights but briefly touched on intervention [24]. Changing the stigma around HIV/AIDS and STIs was central to a number of studies [18, 22, 25]. ST acted as an agent of change to correct misinformed, stigmatizing myths with factually correct stories [18]. ST was used to overcome challenges around adherence to antiretroviral therapy (ART) for HIV treatment [25].

Malena-Chan used Ganz’s narrative framework to gain a deeper understanding, through ST, about how barriers (narrative dissonance) and opportunities (narrative fidelity) arise and contribute towards the translation of knowledge on climate change into action [21].

ST by teenage Latino women encouraged positive change in sexual health attitudes and behaviors *via* DST. The impact of ST on participants found that women experienced improvement in positive social interactions, sense of empowerment, optimism, and control over their futures [16, 17].

Information and insights on the determinants of, and potential ways to, improve sexual health and HIV was sourced *via* ST in Inuit communities [23].

### Reported Benefits of Using Storytelling as a Research Tool

The benefits of ST were evident from direct participant feedback or from feedback from authors. The teenage Latino women said DST provided them with a voice when they had so often felt shamed into silence, and an opportunity for them to reflect on their personal memories and make sense of their lives [16, 17].

ST also revealed nuanced insights, e.g., Ugandan young men revealed that ignorance of health risks was not the primary driver of STIs/HIV risk. Instead they felt that having MSP asserted their masculinity through entrenched cultural views, and this was the dominant factor in determining their health outcomes [24].

ST with respect to mitigating climate change uncovered various social, cultural, and structural barriers to taking action in the everyday lives of the residents of Saskatchewan, Canada. For example, the authors write “dissonance may result from a lack of perceived power to intervene meaningfully through individual roles,” such as struggling to find meaning behind personal actions which seemed insignificant given the scale and scope of the challenge of climate change.

A study exploring HIV stigma in South Africa used ST as an intervention in clinic waiting rooms to open a dialogue on the issue [22]. Interestingly, the stories generated by South African miners based on dispelling myths could easily be shared *via* word of mouth (effectively, *via* the same communication route that spread the misinformation initially) [18].

### ST Aims, Methods, and Analysis of Stories

Only one study explicitly articulated a clear aim to their ST research project, comprising the use of stories as booklets to help patients understand the management of croup in the emergency department [19]. The aims of other studies were less clear but included informing future policy, e.g., on climate change [21], or sexual health/HIV [23], and improving understanding around health risks of unprotected sex and HIV risks of injectable drug use [20].

Thematic analysis was used by 8/10 studies to identify common themes across a number of stories [16, 17, 21–25]. Other methods of analysis included visual text analysis [25]; coding using participant input to develop over-riding themes [23]; analyses of descriptive statistics e.g., age, gender, and birthplace from surveys [16, 17]; and content analysis–the presence, meanings, and relationships between certain words, themes, or concepts to analyze a story about croup [19]. The study on climate change identified points of narrative dissonance (barriers) and narrative fidelity (facilitators) in the ST data to help frame challenges, choices, and outcomes regarding climate change action [21].

## Discussion

In determining the value of ST as a qualitative research method in public health, a narrative synthesis was conducted.

Frameworks to guide the use of ST in research were noticeably absent across most studies, other than one used by Ganz in the study on climate change, which referred to the nestling of personal stories within public narratives based on relationships with others, cultural context, and shared values to convey meaning [21].

### Storytelling’s Value in Extracting Information and Insights on KAB/P

ST elicits nuanced information and emotions that might be inaccessible *via* other means of qualitative or quantitative investigation, especially given the personal and public sensitivities around some topics e.g., HIV stigma. ST can reveal subtle insights on behavior e.g., those related to STI/HIV in Inuit communities that found men in the community were the hard-to-reach ones (for public health messages) and who lacked the social networks that women have through sewing groups and ante-natal groups [23]. Young men in Uganda at high risk for HIV through a lifestyle with MSP were more concerned about things that challenge their pleasure and masculinity than health risks, possibly indicating a need to consider gender power dynamics in any potential health intervention [24].

### Storytelling’s Value as an Intervention to Change KAB/P

Follow-up time, across all studies, was too short to draw conclusions about the impact of ST in changing KAB/P. Changing behavior over the short and long-term is complex and any evaluation would need to reflect that [26].

However, marked positive effects of group ST were documented in the studies by Difulvio, Gubrium, Leukefeld, and Dickinson: women in the studies by Difulvio and Gubrium reported feeling enhanced positivity when telling their own story in a group setting, feeling less socially isolated, discovering more empathy, and, in particular, having the opportunity to have their voices heard [16, 17]. Likewise, positive change in KAB/P in probationers was reported, as structured ST led to insights into personal behavior that sometimes resonated with others [20].

All participants in the 10 studies were members of marginalized groups, and insights into the benefits of ST in changing KAB/P speaks to the finding elsewhere that marginalized groups often reap optimal benefit from ST activities. Researchers find personal stories can illuminate the nuances and the human aspects that underpin challenging social issues often involving marginalized groups [27].

Dickinson defines stories as agents of change that have the power to overcome stalemates around KAB/P relating to stigma, misunderstanding, and myths around HIV and AIDS [18].

### The Storytelling Process and its Value as a Unique Research Tool

The 10 studies revealed that multiple processes could be followed to elicit a meaningful story.

Sensitivities around the public health topic of concern, as well demographics and cultural and/or social norms, were all instrumental in determining the nature of the ST process followed, including the actors deployed. Use of a professional storyteller can facilitate interactions around sensitive topics, e.g., Zeelen used a professional storyteller who created dialogues between animals as a metaphor for real people discussing HIV stigma in their community. Arguably, use of an intermediary agent might diminish the authenticity of the story eventually told because stories can adopt a new form with each telling. That stories are multiplicities, and ST is temporary in nature, whereby stories resist definition and documentation, is supported by core ST literature [28].

The significance of story authenticity also played out with respect to the inherent conflict between the accuracy of medical content versus authenticity of a personal story in the study on children with croup, where details around an X-ray were omitted for not being standard clinical practice [19].

The study in South African ante-natal clinic waiting rooms used a storyteller who knew the local language and culture, and referred to local animals to help listeners find meaning and internalize the messages about protective health behavior rather than just receive information, as per the information deficit model of imparting knowledge [29]. Joshi overcame inhibitions around discussing MSP and risk of HIV/STIs by using written stories and proxy characters [24].

ST reveals insights not necessarily accessible by other means, e.g., subtle cultural influences in health decision-making, providing something potentially additive to other qualitative or quantitative research. The study on climate change unearthed how barriers manifest within the narratives of people who appear to accept climate science, uncovering novel nuances of emotional and moral reasoning, and insights that might not have been readily available *via* other research methods [21].

Thought-mapping used with ST around drug use and sexual practices helped participants work through problems and solutions/strategies, providing the opportunity to create an alternative, more positive story end [20].

Data from the study by Gubrium supports the role of qualitative data as complementary to quantitative data. The author argues for the importance of reflecting the “locally grounded, felt experiences” of participants, as current quantitative scales do not measure up to accurately capture these effects [17].

The majority of studies in the quantitative review (145 studies) were from a chronic disease background that play out over an extended period of time, e.g., HIV and cancer together that comprised the majority of public health topics covered, followed by mental health. The quantitative analysis also showed that most studies were conducted in the US (47.6%) with cancer and cancer screening being most often studied (24/68 studies), possibly reflecting a dominant health concern in that country.

Cardiovascular health featured in only 8/68 studies, possibly suggesting a trend for ST in some areas of public health and scope to extend the range of public health areas which use ST as a research tool.

Finally, the study by Rand illustrates how ST and stories forge a bridge between the intricate realities of everyday lives (qualitative research) and quantitative epidemiology to provide both a close-up and wider view of the public health issue at stake and ultimately help formulate more effective public health interventions [23].

### ST Lends Itself to Teasing out Nuanced Personal Experience

ST thrives on the nuances and non-explicit influences of cultural or ethnic nature. Here, studies featured participants defined by social deprivation, employment status, criminal record, local prevalence of HIV, teenage pregnancy or STIs, and descriptions of community hierarchies, to name a few.

Many of the ST cohorts belonged to hard-to-reach or vulnerable communities, for example, the probationers in rural Kentucky, US, of whom 61% had a history of drug convictions [20], or the teenage Puerto Rican women from an inner city in New England, US [16, 17].

Sensitive or controversial topics frequently form the focus of ST research projects because discussion around them is often avoided in communities precipitating misunderstanding and stigma. ST among probationers created a secure space for open discussion of drug use and HIV risk [20]. In the study in African ante-natal clinics, ST facilitated access to otherwise hard-to-reach members of the community [22].

Choice of facilitators and storytellers was also found to influence ST success. In the Inuit study, the workshop facilitator, a local settler and resident, had an ingrained understanding of the cultural nuances of participants’ lives, e.g., importantly, highly revered Inuit elders were included in the ST study [23].

Stories in the study of ART adherence in South Africa drew on cultural references and social norms, e.g., a belief that HIV medication echoes a subtheme around armed struggle, which alludes to the historical fight in South Africa against Apartheid. Weaponry was also referred to in the struggle to defeat HIV [25].

Prior reference to drawing on culture to tailor narrative research to the study population is made by Larkey and Hecht with their culture-centric, narrative theory-Narrative Communication Prevention Model [30]. This refers to how people structure reality, including health decisions, by telling stories that tap into implicit local cultural values and codes. This approach through the medium of ST can be more effective at reaching certain audiences who might be less involved, or even resistant to, the relevant message, and that narrative or ST might appeal where information provision does not.

### Limitations of ST as a Research Method

The ‘validity’ of data generated by the studies is fundamental to the use of ST as a research tool, but interpretation of the term “validity” as used in qualitative studies is a challenging concept. Noble et al. parallel reliability and “consistency” with trustworthiness, suggesting a need for clarity around the methods undertaken with a “decision-trail” that is transparent enough for reproducibility. In the 10 studies chosen for final qualitative review, few studies satisfied these requirements. Gubrium came closest to a measure of trustworthiness or validity of the qualitative ST data generated by cross-referencing findings to a variety of data sources, for example, transcripts of key DST activities, individual interviews with participants, field notes from workshops, and workshop evaluations [17].

Treffry-Goatley attempted to “validate” storytelling by using triangulation with drawings and music [25].

The value of ST resides in taking the data on their own merit, without generalizing, unlike quantitative research. However, the study by Hartling demonstrates how authenticity can be lost in the effort to meet project objectives, when the story was modified to fit the end purpose-an information booklet [19].

Most ST studies lacked rigorous follow-up and evaluation even over the short term. Going forward, if ST is to be recognized as an effective research method, especially within health and medicine, far greater emphasis needs to be placed on evaluation. Some quantitative analysis might have value in assessing the impact of ST as a tool for research or as an intervention, as seen in the studies by Gubrium and Difulvio [16, 17]. Generally, health and medical research lends to quantitative analysis, e.g., assessing change in a certain clinical parameter, but in contrast, ST often comprises an individual’s perceived truth, and as such it is difficult to construct a measure of such a variable entity.

### Strengths and Limitations of this Systematic Review

This review has several strengths. It was conducted transparently, following established guidelines and a prospectively specified protocol. Multiple databases were searched–covering science, humanities, and arts. To reduce the risk of missing relevant studies, two reviewers were involved (BM and CF).

Limitations to this review include that the topics entered into the full review (stage 2) had to satisfy the key criterion of short-term personal sacrifice with long-term population level gain in order to reflect aspects of the public health scenario presented by AMR. This excluded many studies that may have contained valuable information on ST process and value. ST is also a broad term that might have included stories as told *via* social media or in the mainstream and specialist media. This review was limited to the telling of personal stories.

A few studies using story, ST, or personal narrative were found in the last 18–24 months and were included in the quantitative analysis, for example, a study on beliefs and intentions behind flu vaccination uptake, however, none of these met the tight criteria for the final selection.

### Conclusion

This review found evidence to support ST as having inherent value in revealing nuanced insights on a wide variety of public health topics and played an active role in participants making sense of real-life events and changing KAB/P. The review found most ST studies relate to chronic conditions, specifically HIV and cancer/cancer screening. By uncovering subtle influences on the development of KAB/P, ST lends itself to intractable public health issues that are strongly influenced by peripheral factors e.g., social norms and culture, specific to different communities.

Also, the review suggests that ST could play a more robust and formalized role in public health research. However, in this respect much greater attention is needed in ensuring solid evaluation of ST studies. The value of ST as additive to other qualitative and quantitative data cannot be overstated if a fully rounded and balanced perspective on a public health situation is sought to either inform an intervention or to serve as an intervention itself.
