# Supplementary material for: Storytelling as a Research Tool Used to Explore Insights and as an Intervention in Public Health: A Systematic Narrative Review
Source: Int J Public Health. 2021 Nov 2;66:1604262. doi: 10.3389/ijph.2021.1604262 (PMC8592844; doi:10.3389/ijph.2021.1604262)
Supplement: Supplementary file 1 [file DataSheet1.docx]

**Supplementary material**

**Appendices (1-5)**

**Storytelling as a research tool used to explore insights and as an intervention in public health: a systematic narrative review**

**May 24, 2021**

**Supplementary file 1: Characteristic criteria of public health issues used to select topics for full text review. Four examples are provided. The criteria used here will be applied to other public health issues found as part of the search and selection process.**

| **Criteria (used to determine inclusion/exclusion of public health topic)** | **Is there an individual, immediate cost but a long-term population gain? Issue involves an element of prevention.** | **Is there misunderstanding, misinformation, or misconception associated with the issue?** | **Is there a need to change knowledge,**  **attitudes and practices (perceptions) associated with the issue?** | **Which public populations are most relevant to this issue?** | **Are there peer-reviewed publications on storytelling used as a research method?** |
| --- | --- | --- | --- | --- | --- |
|  |  |  |  |  |  |
| **Public health topic:** |  |  |  |  |  |
| **Antimicrobial resistance (AMR)** | Avoid using antibiotics unnecessarily in immediate term to prevent population-level resistance developing in the long-term. Might mean some personal cost of forgoing antibiotic and slightly longer illness in the short-term. | Yes. The nature of AMR, e.g. which diseases antibiotics treat, bacteria vs. virus, or perceptions that personal actions will not have an impact on a global issue of this magnitude. Defining the appropriateness and inappropriateness of antibiotic prescribing in primary care. | Yes. Knowledge might be wrongly informed, attitudes formed through experiences, behaviours as a result of knowledge, attitudes or external circumstances. | AMR is a global problem and applies universally, however certain populations are more likely to use antibiotics.  Potentially parents and young children or people with chronic diseases, People with chronic diseases e.g. COPD  HCPs. Approx. 80% antibiotics are prescribed by primary care. | Limited, if any, evidence of storytelling used as a research method in the context of this public health issue.  No peer-reviewed papers, only blog posts, news articles, short reports from charities, not-for profit organisations e.g. MESH; https://mesh.tghn.org/its-complicated-workshop-2017/narrative-and-storytelling/ |
| **Cancer screening and prevention** | Screening may be unpleasant or unwanted on a personal level in the immediate term but is preventatively beneficial long term for the population. | Yes. Misinformation and misperceptions about false positives; perceived risk and fear; dislike/discomfort with screening and/or biopsy procedures, doubts about self-efficacy (of performing self-administered screening test can inhibit screening uptake. | Yes. Misinformation may lead to incorrect knowledge, misinformed/mis-influenced attitude and consequently behaviour. More nuanced understanding of these factors via storytelling research might contribute to change. | Mainly adults in who meet ‘at-risk’ criteria e.g. age bracket for colorectal or breast cancer screening. Special groups at high risk e.g. Alaska natives (2x rate CRC as US general population). | Some evidence of storytelling used as a research method in the context of this public health issue. |
| **Vaccinations** | Vaccinate a child in the immediate term at personal cost of discomfort and inconvenience, and mild sickness possibly to prevent population level disease in the longer-term. | Misinformation and misperceptions about risks associated with disease and with vaccination-related adverse effects, especially post-MMR/autism debacle in late 90s).  Storytelling interventions published in relation  to understanding mother-daughter interactions/perceptions that contribute to HPV vaccination uptake.  The individual vaccinated might receive a vaccination for a disease that has very low prevalence and perceived risk can influence uptake of vaccine, as can negative media reporting e.g. MMR.  Anti-vaccine messaging through media- social media etc.. carefully controlled by site administrators. | Yes. Need to dispel misinformation and counter anti-vaccine groups. Stories to combat anti-vaccine misinformation are needed. Some experts advocate using the anti-vaccine campaigners tactics of powerful stories to reverse vaccine-hesitancy).  Awareness and knowledge of importance of vaccination, side effects and efficacy.  Misinformation in media and hearsay, social media, word of mouth.  Very strong examples of the power of storytelling leaving lasting impression: stories about childhood vaccinations causing sickness. Hep B, MMR (‘overnight autism’).  Anti-vaccine messaging through media, including social media etc.. can be carefully controlled by site administrators. | General public: especially parents of young children  Subgroups documented in peer-reviewed literature e.g Cambodian mother-daughter pairs. Healthcare professionals (HCPs). | Some evidence of storytelling used as a research method in the context of this public health issue. |
| **Climate change and care of the natural environment** | Personal actions and cost in the immediate term e.g. recycling or walking rather than driving, buying local produce rather than goods that have travelled across the globe, will contribute to reducing climate change in the long-term, and improving health-related effects of climate change e.g. flooding, over-heating and respiratory disease. | Yes. Doubts about what can be done about the status of climate change as a scientific and physical phenomenon; and response scepticism, relating to doubts about the efficacy of action taken to address climate change.  Differentiation according to perceived costs seem to be appropriate to classify climate-friendly actions. People’s perception of climate change influences their level of  concern, which ultimately affects their motivation to act. | Yes. Need to change motivation to act in a climate sparing way. UK public say they feel powerless e.g. the sentiment that individual actions made little difference). People in the UK perceive government responsible for implementing climate change adaptation.  If people feel they cannot change a situation, they will very likely retreat into apathy and resignation and thus will be less likely to address environmental issues | Published storytelling research within the context of climate change, to date, has applied to specialist populations e.g. Inuits – northern Canada.  Inupiat people, Alaska | Some published storytelling research e.g. Community participatory multi-media storytelling after week-long workshop to engage in project design, data extraction to explore climate-health relationships.  e.g. traditional storytelling of Inupuit community, Alaska.  Production 3-5 minute digital A/V stories on effects of CC in students’ countries. |

**Supplementary file 2a: Data extraction for 145 studies included in the quantitative analysis (stage 1)**

| **Title and author** | **Date** | **Reason for quantitative analysis inclusion** | **Public health topic** | **Nature of story** | **Method for story** | **Process of ST** | **Evaluation of ST method** | **Relevance to research question** | **Ethnic or cultural group, or more specific location** | **Geographical location** | **Main reason for study exclusion** |
| --- | --- | --- | --- | --- | --- | --- | --- | --- | --- | --- | --- |
| Hopfer S. Effects of a narrative HPV vaccination intervention aimed at reaching college women: a randomized controlled trial. Prev Sci. 2012 Apr;13(2):173-82. doi: 10.1007/s11121-011-0254-1. PMID: 21993613.l | 2012 |  | vaccination | story/ies | written personal stories | no | yes | potential to change KAB |  | US | Storytelling method too insignificant |
| Hopfer S, Garcia S, Duong HT, Russo JA, Tanjasiri SP. A Narrative Engagement Framework to Understand HPV Vaccination Among Latina and Vietnamese Women in a Planned Parenthood Setting. Health Educ Behav. 2017 Oct;44(5):738-747. doi: 10.1177/1090198117728761. Epub 2017 Aug 30. PMID: 28854812; PMCID: PMC5741467. | 2017 | Personal narrative and PH | vaccination | narratives/narrative accounts | personal narrative/s | no | no | extracts information, provides insights | Latino and Vietnamese | US | Storytelling method too insignificant |
| Collins D, Villagran MM, Sparks L. Crossing borders, crossing cultures: barriers to communication about cancer prevention and treatment along the U.S./Mexico border. Patient Educ Couns. 2008 Jun;71(3):333-9. doi: 10.1016/j.pec.2008.03.013. Epub 2008 Apr 23. PMID: 18436416. | 2008 | Personal narrative and PH | cancer | narratives/narrative accounts | in depth interview | no | no | extracts information, provides insights | Mexicans | US | Storytelling method too insignificant |
| Geerlings E, Kaselitz E, Aborigo RA, Williams J, Youngblood J, Avrakotos A, Chatio S, Moyer C. 'I am still confused as to what caused the problem': Perceptions of mothers on communication regarding newborn illness and death in Northern Ghana. Glob Public Health. 2019 Dec;14(12):1784-1792. doi: 10.1080/17441692.2019.1642930. Epub 2019 Jul 19. PMID: 31322063. newborn illness and death in Northern Ghana | 2019 | Personal narrative and PH | reproductive and sexual health | storytelling as minor part of mixed method study | in depth interview | no | no | extracts information, provides insights | Ghana | Africa | Storytelling method too insignificant |
| Hennelly MO, Sly JR, Villagra C, Jandorf L. Narrative message targets within the decision-making process to undergo screening colonoscopy among Latinos: a qualitative study. J Cancer Educ. 2015 Jun;30(2):268-76. doi: 10.1007/s13187-014-0765-0. PMID: 25516413. | 2015 | Personal narrative and PH | cancer | narratives/narrative accounts | in depth interview | NA | NA | both extract and intervention | Latino | US | Storytelling method too insignificant |
| Hussey W. Slivers of the journey: the use of photovoice and storytelling to examine female to male transsexuals' experience of health care access. J Homosex. 2006;51(1):129-58. doi: 10.1300/J082v51n01_07. PMID: 16893829. | 2006 | Personal narrative and PH | reproductive and sexual health | storytelling | Photovoice | yes | yes | extracts information, provides insights | transgender | US | No short term personal sacrifice/long term population gain |
| Janzen, R; Pancer, S. M.; Janzen, R., Pancer, S. M., Nelson, G., Loomis, C., & Hasford, J. (2010). Evaluating community participation as prevention: Life narratives of youth. Journal of Community Psychology, 38(8), 992–1006. https://doi.org/10.1002/jcop.20410 | 2010 | Personal narrative and PH | other | storytelling as minor part of mixed method study | in depth interview | no | no | NA |  | Canada | Storytelling method too insignificant |
| Jinks C, Ong BN, O'Neill T. "Well, it's nobody's responsibility but my own." A qualitative study to explore views about the determinants of health and prevention of knee pain in older adults. BMC Public Health. 2010 Mar 22;10:148. doi: 10.1186/1471-2458-10-148. PMID: 20307283; PMCID: PMC2853508. | 2010 | Personal narrative and PH | other | storytelling as minor part of mixed method study | semi-structured interview | no | no | extracts information, provides insights |  | UK | Storytelling method too insignificant |
| Johnson JL, Bottorff JL, Balneaves LG, Grewal S, Bhagat R, Hilton BA, Clarke H. South Asian womens' views on the causes of breast cancer: images and explanations. Patient Educ Couns. 1999 Jul;37(3):243-54. doi: 10.1016/s0738-3991(98)00118-9. PMID: 14528550. | 1999 | Personal narrative and PH | cancer | narratives/narrative accounts | in depth interview | no | yes | extracts information, provides insights | Soutth Asian | Canada | Storytelling method too insignificant |
| Joseph M, Rab F, Panabaker K, Nisker J. Feelings of Women With Strong Family Histories Who Subsequent to Their Breast Cancer Diagnosis Tested BRCA Positive. Int J Gynecol Cancer. 2015 May;25(4):584-92. doi: 10.1097/IGC.0000000000000403. PMID: 25675043. | 2015 | Personal narrative and PH | cancer | story/ies | written personal stories | yes | no | extracts information, provides insights | familial history breast cancer | Canada | No short term personal sacrifice/long term population gain |
| Koo K. Carers' representations of affective mental disorders in British Chinese communities. Sociol Health Illn. 2012 Nov;34(8):1140-55. doi: 10.1111/j.1467-9566.2012.01461.x. Epub 2012 Feb 14. PMID: 22332911. | 2012 | Personal narrative and PH | mental health | narratives/narrative accounts | in depth interview | no | no | extracts information, provides insights | Chinese | UK | No short term personal sacrifice/long term population gain |
| Mataya R, Mathanga D, Chinkhumba J, Chibwana A, Chikaphupha K, Cardiello J. A qualitative study exploring attitudes and perceptions of HIV positive women who stopped breastfeeding at six months to prevent transmission of HIV to their children. Malawi Med J. 2013 Mar;25(1):15-9. PMID: 23717750; PMCID: PMC3653193. | 2013 | Personal narrative and PH | HIV | storytelling as minor part of mixed method study | in depth interview | no | no | extracts information, provides insights | (Malawi) | Africa | Storytelling method too insignificant |
| McHenry MS, Apondi E, McAteer CI, Nyandiko WM, Fischer LJ, Ombitsa AR, Aluoch J, Scanlon ML, Vreeman RC. Tablet-based disclosure counselling for HIV-infected children, adolescents, and their caregivers: a pilot study. Afr J AIDS Res. 2018 Sep;17(3):249-258. doi: 10.2989/16085906.2018.1509101. Epub 2018 Oct 14. PMID: 30319030; PMCID: PMC6376488. | 2018 | Personal narrative and PH | HIV | storytelling as minor part of mixed method study | digital or video storytelling | no | no | potential to change KAB | Kenya | Africa | Storytelling method too insignificant |
| Murphy, G., Peters, K., Wilkes, L. et al. Adult children of parents with mental illness: parenting journeys. BMC Psychol 6, 37 (2018). https://doi.org/10.1186/s40359-018-0248-x | 2018 | Personal narrative and PH | mental health | narratives/narrative accounts | in depth interview | NA | NA | extracts information, provides insights |  | Australasia | Storytelling method too insignificant |
| Ng'ang'a CM, Bukachi SA, Bett BK. Lay perceptions of risk factors for Rift Valley fever in a pastoral community in northeastern Kenya. BMC Public Health. 2016 Jan 13;16:32. doi: 10.1186/s12889-016-2707-8. PMID: 26762147; PMCID: PMC4712502. | 2016 | Personal narrative and PH | other | narratives/narrative accounts | personal narrative/s | no | yes | potential to change KAB | (Kenya) | Africa | Storytelling method too insignificant |
| Peter Robinson & Paula Geldens (2014) Stories from two generations of gay men living in the midst of HIV-AIDS, Journal of Australian Studies, 38:2, 233-245, DOI: 10.1080/14443058.2014.895957 | 2014 | Personal narrative and PH | HIV | not story narrative but overarching narrative theme | in depth interview | NA | NA | extracts information, provides insights | Australia | Australasia | Storytelling method too insignificant |
| Rosén A, Emmelin M, Carlsson A, Hammarroth S, Karlsson E, Ivarsson A. Mass screening for celiac disease from the perspective of newly diagnosed adolescents and their parents: a mixed-method study. BMC Public Health. 2011 Oct 21;11:822. doi: 10.1186/1471-2458-11-822. PMID: 22017750; PMCID: PMC3229548. | 2011 | Personal narrative and PH | other | NA | NA | NA | NA | extracts information, provides insights | (UK) | Europe | Storytelling method too insignificant |
| Sallinen, M; Kukkurainen, M. Sallinen M, Kukkurainen ML, Peltokallio L. Finally heard, believed and accepted--peer support in the narratives of women with fibromyalgia. Patient Educ Couns. 2011 Nov;85(2):e126-30. doi: 10.1016/j.pec.2011.02.011. Epub 2011 Mar 17. PMID: 21419588. | 2011 | Personal narrative and PH | other | not story narrative but overarching narrative theme | in depth interview | no | no | extracts information, provides insights | Finland | Europe | Storytelling method too insignificant |
| Sanchez-Birkhead AC, Kennedy HP, Callister LC, Miyamoto TP. Navigating a new health culture: experiences of immigrant Hispanic women. J Immigr Minor Health. 2011 Dec;13(6):1168-74. doi: 10.1007/s10903-010-9369-x. PMID: 20607608. | 2011 | Personal narrative and PH | other | not story narrative but overarching narrative theme | in depth interview | no | no | extracts information, provides insights | Hispanic | US | Storytelling method too insignificant |
| Stern E, Cooper D. Experiences and conceptualizations of sexual debut from the narratives of South African men and women in the context of HIV/AIDS. Afr J AIDS Res. 2014;13(2):121-31. doi: 10.2989/16085906.2014.943252. PMID: 25174629. | 2014 | Personal narrative and PH | HIV | narratives/narrative accounts | in depth interview | NA | NA | extracts information, provides insights | South Africa | Africa | Storytelling method too insignificant |
| Sumankuuro J, Mahama MY, Crockett J, Wang S, Young J. Narratives on why pregnant women delay seeking maternal health care during delivery and obstetric complications in rural Ghana. BMC Pregnancy Childbirth. 2019 Jul 23;19(1):260. doi: 10.1186/s12884-019-2414-4. PMID: 31337348; PMCID: PMC6651920. | 2019 | Personal narrative and PH | reproductive and sexual health | narratives/narrative accounts | focus group | no | no | extracts information, provides insights | Ghana | Africa | Storytelling method too insignificant |
| O'Donnell L, Fuxman S. Effectiveness of a Brief Home Parenting Intervention for Reducing Early Sexual Risks Among Latino Adolescents: Salud y Éxito. J Sch Health. 2017 Nov;87(11):858-864. doi: 10.1111/josh.12560. PMID: 29023839; PMCID: PMC6070345. | 2017 | Story and PH | reproductive and sexual health | story/ies | role model story | no | no | potential to change KAB | Latino | US | Storytelling method too insignificant |
| Bentley, M. E; Corneli, A. L.; Piwoz, E.; Moses, A.; Nkhoma, J.; Tohill, B. C.; Ahmed, Y.; Adair, L.; Jamieson, D. J.; van der Horst, C.; B. A. N. Formative Study Grp Perceptions of the role of maternal nutrition in HIV-positive breast-feeding women in Malawi | 2005 | Story and PH | HIV | story/ies | semi-structured interview | no | no | extracts information, provides insights | Malawi | Africa | Storytelling method too insignificant |
| Beres LK, Winskell K, Neri EM, Mbakwem B, Obyerodhyambo O. Making sense of HIV testing: social representations in young Africans' HIV-related narratives from six countries. Glob Public Health. 2013;8(8):890-903. doi: 10.1080/17441692.2013.827734. Epub 2013 Sep 5. PMID: 24004339; PMCID: PMC3925508. | 2013 | Story and PH | HIV | narratives/narrative accounts | scriptwriting narratives | no | no | extracts information, provides insights | six nattions | Africa | Storytelling method too insignificant |
| Berkley-Patton J, Goggin K, Liston R, Bradley-Ewing A, Neville S. Adapting effective narrative-based HIV-prevention interventions to increase minorities' engagement in HIV/AIDS services. Health Commun. 2009 Apr;24(3):199-209. doi: 10.1080/10410230902804091. PMID: 19415552; PMCID: PMC2746743. | 2009 | Story and PH | HIV | story/ies | role model storywriting | yes | no | both extract and intervention |  | US | Storytelling method too insignificant |
| Bhattacharya G. Self-management of type 2 diabetes among African Americans in the Arkansas Delta: a strengths perspective in social-cultural context. J Health Care Poor Underserved. 2012 Feb;23(1):161-78. doi: 10.1353/hpu.2012.0035. PMID: 22643469. | 2012 | Story and PH | diabetes | story/ies | personal narrative/s | no | no | potential to change KAB | African Americans | US | No short term personal sacrifice/long term population gain |
| Bird SM, Wiles JL, Okalik L, Kilabuk J, Egeland GM. Living with diabetes on Baffin Island: Inuit storytellers share their experiences. Can J Public Health. 2008 Jan-Feb;99(1):17-21. doi: 10.1007/BF03403734. PMID: 18435384; PMCID: PMC6975636. | 2008 | Story and PH | diabetes | narratives/narrative accounts | personal narrative/s |  | no | extracts information, provides insights | Inuit | Canada | No short term personal sacrifice/long term population gain |
| Bokhour BG, Fix GM, Gordon HS, Long JA, DeLaughter K, Orner MB, Pope C, Houston TK. Can stories influence African-American patients' intentions to change hypertension management behaviors? A randomized control trial. Patient Educ Couns. 2016 Sep;99(9):1482-8. doi: 10.1016/j.pec.2016.06.024. Epub 2016 Jun 21. PMID: 27387121. change hypertension management behaviors? A randomized control trial | 2016 | Story and PH | cardiovascular health | story/ies | digital or video storytelling | no | yes | changes KAB | African-American | US | No short term personal sacrifice/long term population gain |
| Dillard AJ, Fagerlin A, Dal Cin S, Zikmund-Fisher BJ, Ubel PA. Narratives that address affective forecasting errors reduce perceived barriers to colorectal cancer screening. Soc Sci Med. 2010 Jul;71(1):45-52. doi: 10.1016/j.socscimed.2010.02.038. Epub 2010 Mar 21. PMID: 20417005; PMCID: PMC4033575. screening | 2010 | Story and PH | cancer | narratives/narrative accounts | third person storytelling/narrative | yes | yes | both extract and intervention | colorectal cancer | US | No short term personal sacrifice/long term population gain |
| Esacove AW, Andringa KR. The process of preventing pregnancy: women's experiences and emergency contraception use. Qual Health Res. 2002 Nov;12(9):1235-47. doi: 10.1177/1049732302238247. PMID: 12448669. | 2002 | Story and PH | reproductive and sexual health | story/ies | in depth interview | no | no | extracts information, provides insights |  | US | Storytelling method too insignificant |
| Falzon C, Radel R, Cantor A, d'Arripe-Longueville F. Understanding narrative effects in physical activity promotion: the influence of breast cancer survivor testimony on exercise beliefs, self-efficacy, and intention in breast cancer patients. Support Care Cancer. 2015 Mar;23(3):761-8. doi: 10.1007/s00520-014-2422-x. Epub 2014 Sep 4. PMID: 25186211. | 2015 | Story and PH | cancer | story/ies | personal narrative/s | no | yes | changes KAB | breast cancer; France | Europe | No short term personal sacrifice/long term population gain |
| Goddu AP, Raffel KE, Peek ME. A story of change: The influence of narrative on African-Americans with diabetes. Patient Educ Couns. 2015 Aug;98(8):1017-24. doi: 10.1016/j.pec.2015.03.022. Epub 2015 Apr 6. PMID: 25986500; PMCID: PMC4492448. | 2015 | Story and PH | diabetes | story/ies | in depth interview | no | yes | both extract and intervention |  | US | No short term personal sacrifice/long term population gain |
| Haxaire C, Tromeur C, Couturaud F, Leroyer C. A Qualitative Study to Appraise Patients and Family Members Perceptions, Knowledge, and Attitudes towards Venous Thromboembolism Risk. PLoS One. 2015 Nov 4;10(11):e0142070. doi: 10.1371/journal.pone.0142070. PMID: 26536460; PMCID: PMC4633063. | 2015 | Story and PH | other | story/ies | in depth interview | no | no | extracts information, provides insights | France | Europe | Storytelling method too insignificant |
| Heilemann MV, Soderlund PD, Kehoe P, Brecht ML. A Transmedia Storytelling Intervention With Interactive Elements to Benefit Latinas' Mental Health: Feasibility, Acceptability, and Efficacy. JMIR Ment Health. 2017 Oct 19;4(4):e47. doi: 10.2196/mental.8571. PMID: 29051135; PMCID: PMC5668652. | 2017 | Story and PH | mental health | storytelling as minor part of mixed method study | part of multi-method study | no | no | extracts information, provides insights | Latinos | US | Storytelling method too insignificant |
| Heaton B, Gebel C, Crawford A, Barker JC, Henshaw M, Garcia RI, Riedy C, Wimsatt MA. Using Storytelling to Address Oral Health Knowledge in American Indian and Alaska Native Communities. Prev Chronic Dis. 2018 May 24;15:E63. doi: 10.5888/pcd15.170305. PMID: 29806581; PMCID: PMC5985855. | 2018 | Story and PH | other | story/ies | role model storywriting | yes | yes | extracts information, provides insights | American Indian and Alaskan Native | US | No short term personal sacrifice/long term population gain |
| Jamner MS, Wolitski RJ, Corby NH. Impact of a longitudinal community HIV intervention targeting injecting drug users' stage of change for condom and bleach use. Am J Health Promot. 1997 Sep-Oct;12(1):15-24. doi: 10.4278/0890-1171-12.1.15. PMID: 10170430. | 1997 | Story and PH | HIV | story/ies | role model storywriting | no | yes | both extract and intervention |  | US | Storytelling method too insignificant |
| Kamat VR. Dying under the bird's shadow: narrative representations of degedege and child survival among the Zaramo of Tanzania. Med Anthropol Q. 2008 Mar;22(1):67-93. doi: 10.1111/j.1548-1387.2008.00004.x. PMID: 18610814. | 2008 | Story and PH | other | narratives/narrative accounts | personal narrative/s | no | no | extracts information, provides insights | Zaramo people of Tanzania | Africa | No short term personal sacrifice/long term population gain |
| Kangas I. Making Sense of Depression: Perceptions of Melancholia in Lay Narratives. Health. 2001;5(1):76-92. doi:10.1177/136345930100500104 | 2001 | Story and PH | mental health | narratives/narrative accounts | in depth interview | no | no | extracts information, provides insights | Finland | Europe | No short term personal sacrifice/long term population gain |
| . Kim J, Nan X. Temporal Framing Effects Differ for Narrative Versus Non-Narrative Messages: The Case of Promoting HPV Vaccination. Communication Research. 2019;46(3):401-417. doi:10.1177/0093650215626980 | 2019 | Story and PH | vaccination | story/ies | online activity | no | no | extracts information, provides insights |  | US | Storytelling method too insignificant |
| Lamb RLB, Ramos Jaraba SM, Graciano Tangarife V, Garcés-Palacio IC. Evaluation of Entertainment Education Strategies to Promote Cervical Cancer Screening and Knowledge in Colombian Women. J Cancer Educ. 2018 Oct;33(5):1094-1101. doi: 10.1007/s13187-017-1213-8. PMID: 28374230. | 2018 | Story and PH | cancer | story/ies | third person storytelling/narrative | no | no | extracts information, provides insights | Columbia | South America | Storytelling method too insignificant |
| Lee MJ, Bichard SL. Effective message design targeting college students for the prevention of binge-drinking: basing design on rebellious risk-taking tendency. Health Commun. 2006;20(3):299-308. doi: 10.1207/s15327027hc2003_9. PMID: 17137421. | 2006 | Story and PH | alcohol and drug abuse | story/ies | online activity | yes | no | potential to change KAB |  | US | No short term personal sacrifice/long term population gain |
| Lemal M, Van den Bulck J. Testing the effectiveness of a skin cancer narrative in promoting positive health behavior: a pilot study. Prev Med. 2010 Aug;51(2):178-81. doi: 10.1016/j.ypmed.2010.04.019. Epub 2010 May 4. PMID: 20450930. | 2010 | Story and PH | cancer | story/ies | online activity | no | yes | potential to change KAB | Holland | Europe | No short term personal sacrifice/long term population gain |
| .Lindvall K, Larsson C, Weinehall L, Emmelin M. Weight maintenance as a tight rope walk - a Grounded Theory study. BMC Public Health. 2010 Feb 1;10:51. doi: 10.1186/1471-2458-10-51. PMID: 20122140; PMCID: PMC2835685. | 2010 | Story and PH | obesity | story/ies | in depth interview | no | no | both extract and intervention | Sweden | Europe | No short term personal sacrifice/long term population gain |
| López JA, García RF, Martí TS. Drugs and Mental Health Problems among the Roma: Protective Factors Promoted by the Iglesia Evangélica Filadelfia. Int J Environ Res Public Health. 2018 Feb 14;15(2):335. doi: 10.3390/ijerph15020335. PMID: 29443877; PMCID: PMC5858404. | 2018 | Story and PH | mental health | story/ies | focus group | no | no | extracts information, provides insights | Roma populatioon, Spain | Europe | No short term personal sacrifice/long term population gain |
| Lu AS, Thompson D, Baranowski J, Buday R, Baranowski T. Story Immersion in a Health Videogame for Childhood Obesity Prevention. Games Health J. 2012 Feb 15;1(1):37-44. doi: 10.1089/g4h.2011.0011. PMID: 24066276; PMCID: PMC3779587. | 2012 | Story and PH | obesity | story/ies | digital or video storytelling | no | yes | both extract and intervention | African-American, Caucasian, and Hispanic | US | No short term personal sacrifice/long term population gain |
| McGregor LM, von Wagner C, Vart G, Yuen WC, Raine R, Wardle J, Robb KA. The impact of supplementary narrative-based information on colorectal cancer screening beliefs and intention. BMC Cancer. 2015 Mar 21;15:162. doi: 10.1186/s12885-015-1167-3. PMID: 25884168; PMCID: PMC4397889. | 2015 | Story and PH | cancer | narratives/narrative accounts | written personal stories | no | yes | both extract and intervention | England | Europe | No short term personal sacrifice/long term population gain |
| McQueen A, Caburnay C, Kreuter M, Sefko J. Improving Adherence to Colorectal Cancer Screening: A Randomized Intervention to Compare Screener vs. Survivor Narratives. J Health Commun. 2019;24(2):141-155. doi: 10.1080/10810730.2019.1587109. Epub 2019 Mar 29. PMID: 30924402; PMCID: PMC6459702. | 2019 | Story and PH | cancer | story/ies | role model story | no | no | changes KAB |  | US | No short term personal sacrifice/long term population gain |
| Frank LB, Murphy ST, Chatterjee JS, Moran MB, Baezconde-Garbanati L. Telling stories, saving lives: creating narrative health messages. Health Commun. 2015;30(2):154-63. doi: 10.1080/10410236.2014.974126. PMID: 25470440; PMCID: PMC5608451. | 2015 | Story and PH | cancer | story/ies | film | no | yes | both extract and intervention | Amerixcans mixed origin | US | Storytelling method too insignificant |
| Murphy ST, Frank LB, Chatterjee JS, Baezconde-Garbanati L. Narrative versus Non-narrative: The Role of Identification, Transportation and Emotion in Reducing Health Disparities. J Commun. 2013 Feb;63(1):10.1111/jcom.12007. doi: 10.1111/jcom.12007. PMID: 24347679; PMCID: PMC3857102. | 2013 | Story and PH | cancer | story/ies | film | NA | NA | potential to change KAB | Hispnaic and mixed American | US | Storytelling method too insignificant |
| Murphy ST, Frank LB, Chatterjee JS, Moran MB, Zhao N, Amezola de Herrera P, Baezconde-Garbanati LA. Comparing the Relative Efficacy of Narrative vs Nonnarrative Health Messages in Reducing Health Disparities Using a Randomized Trial. Am J Public Health. 2015 Oct;105(10):2117-23. doi: 10.2105/AJPH.2014.302332. Epub 2015 Apr 23. PMID: 25905845; PMCID: PMC4566521. | 2015 | Story and PH | cancer | story/ies | film | NA | NA | potential to change KAB | non-Hispanic mixed Americans | US | Storytelling method too insignificant |
| Okuhara T, Ishikawa H, Okada M, Kato M, Kiuchi T. Persuasiveness of Statistics and Patients' and Mothers' Narratives in Human Papillomavirus Vaccine Recommendation Messages: A Randomized Controlled Study in Japan. Front Public Health. 2018 Apr 12;6:105. doi: 10.3389/fpubh.2018.00105. PMID: 29707533; PMCID: PMC5906532. | 2018 | Story and PH | vaccination | story/ies | role model story | NA | NA | potential to change KAB | mothers daughters in Japan | Asia | Storytelling method too insignificant |
| Ornelas IJ, Ho K, Jackson JC, Moo-Young J, Le A, Do HH, Lor B, Magarati M, Zhang Y, Taylor VM. Results From a Pilot Video Intervention to Increase Cervical Cancer Screening in Refugee Women. Health Educ Behav. 2018 Aug;45(4):559-568. doi: 10.1177/1090198117742153. Epub 2017 Dec 4. PMID: 29202606; PMCID: PMC7012240. | 2018 | Story and PH | cancer | story/ies | film | yes | yes | extracts information, provides insights | Karen-Burmese and Nepali-Bhutanese refugees, | US | Storytelling method too insignificant |
| Penn C, Watermeyer J, Evans M. Why don't patients take their drugs? The role of communication, context and culture in patient adherence and the work of the pharmacist in HIV/AIDS. Patient Educ Couns. 2011 Jun;83(3):310-8. doi: 10.1016/j.pec.2011.02.018. Epub 2011 Apr 7. PMID: 21474263. | 2011 | Story and PH | HIV | storytelling as minor part of mixed method study | in depth interview | no | no | extracts information, provides insights | South Africa | Africa | Storytelling method too insignificant |
| Rietmeijer CA, Kane MS, Simons PZ, Corby NH, Wolitski RJ, Higgins DL, Judson FN, Cohn DL. Increasing the use of bleach and condoms among injecting drug users in Denver: outcomes of a targeted, community-level HIV prevention program. AIDS. 1996 Mar;10(3):291-8. PMID: 8882669. | 1996 | Story and PH | HIV | storytelling as minor part of mixed method study | role model story | no | NA | changes KAB |  | US | Storytelling method too insignificant |
| Ritchie D, Schulz S, Bryce A. One size fits all? A process evaluation--the turn of the 'story' in smoking cessation. Public Health. 2007 May;121(5):341-8. doi: 10.1016/j.puhe.2006.12.001. Epub 2007 Feb 9. PMID: 17292931. | 2007 | Story and PH | smoking | story/ies | personal narrative/s | no | no | extracts information, provides insights | Scotland | Europe | Storytelling method too insignificant |
| Taylor AM, Hutchings M. Using video narratives of women's lived experience of breastfeeding in midwifery education: exploring its impact on midwives' attitudes to breastfeeding. Matern Child Nutr. 2012 Jan;8(1):88-102. doi: 10.1111/j.1740-8709.2010.00258.x. Epub 2010 Aug 16. PMID: 22136222; PMCID: PMC6860724. | 2012 | Story and PH | reproductive and sexual health | narratives/narrative accounts | film | no | no | both extract and intervention | UK | Europe | No short term personal sacrifice/long term population gain |
| Unger JB, Cabassa LJ, Molina GB, Contreras S, Baron M. Evaluation of a fotonovela to increase depression knowledge and reduce stigma among Hispanic adults. J Immigr Minor Health. 2013 Apr;15(2):398-406. doi: 10.1007/s10903-012-9623-5. PMID: 22485012; PMCID: PMC3602405. | 2013 | Story and PH | mental health | story/ies | role model story | no | no | extracts information, provides insights | Hispanic/Latino | US | Storytelling method too insignificant |
| Victor RG, Ravenell JE, Freeman A, Bhat DG, Storm JS, Shafiq M, Knowles P, Hannan PJ, Haley R, Leonard D. A barber-based intervention for hypertension in African American men: design of a group randomized trial. Am Heart J. 2009 Jan;157(1):30-6. doi: 10.1016/j.ahj.2008.08.018. PMID: 19081393; PMCID: PMC2638989. trial | 2009 | Story and PH | cardiovascular health | story/ies | role model story | no | no | both extract and intervention | African-Americans | US | Storytelling method too insignificant |
| Williams IC, Utz SW, Hinton I, Yan G, Jones R, Reid K. Enhancing diabetes self-care among rural African Americans with diabetes: results of a two-year culturally tailored intervention. Diabetes Educ. 2014 Mar-Apr;40(2):231-9. doi: 10.1177/0145721713520570. Epub 2014 Jan 29. PMID: 24478047; PMCID: PMC4692724. | 2014 | Story and PH | diabetes | story/ies | role model story | no | no | both extract and intervention | African-Americans | US | Storytelling method too insignificant |
| Winskell K, Beres LK, Hill E, Mbakwem BC, Obyerodhyambo O. Making sense of abstinence: social representations in young Africans' HIV-related narratives from six countries. Cult Health Sex. 2011 Sep;13(8):945-59. doi: 10.1080/13691058.2011.591431. PMID: 21787256; PMCID: PMC3358789. | 2011 | Story and PH | HIV | story/ies | scriptwriting narratives | no | no | both extract and intervention |  | Africa | Other (see notes) |
| Winskell K, Beres LK, Hill E, Mbakwem BC, Obyerodhyambo O. Making sense of abstinence: social representations in young Africans' HIV-related narratives from six countries. Cult Health Sex. 2011 Sep;13(8):945-59. doi: 10.1080/13691058.2011.591431. PMID: 21787256; PMCID: PMC3358789. | 2011 | Story and PH | HIV | story/ies | scriptwriting narratives | no | no | extracts information, provides insights | Nigeria | Africa | Storytelling method too insignificant |
| Winskell K, Hill E, Obyerodhyambo O. Comparing HIV-related symbolic stigma in six African countries: social representations in young people's narratives. Soc Sci Med. 2011 Oct;73(8):1257-65. doi: 10.1016/j.socscimed.2011.07.007. Epub 2011 Jul 28. PMID: 21864965; PMCID: PMC3358781. | 2011 | Story and PH | HIV | story/ies | scriptwriting narratives | no | no | extracts information, provides insights |  | Africa | Storytelling method too insignificant |
| Winskell, K; Holmes, K.; Neri, E.; Berkowitz, R.; Mbakwem, B.; Obyerodhyambo, O. Making sense of HIV stigma: Representations in young Africans' HIV-related narratives | 2015 | Story and PH | HIV | story/ies | scriptwriting narratives | yes | no | extracts information, provides insights |  | Africa | Storytelling method too insignificant |
| Nyhan B, Reifler J, Richey S, Freed GL. Effective messages in vaccine promotion: a randomized trial. Pediatrics. 2014 Apr;133(4):e835-42. doi: 10.1542/peds.2013-2365. Epub 2014 Mar 3. PMID: 24590751. | 2014 | Story and PH | vaccination | narratives/narrative accounts | role model story | NA | NA | both extract and intervention |  | US | Storytelling method too insignificant |
| Leukefeld C, Roberto H, Hiller M, Webster M, Logan TK, Staton-Tindall M. HIV prevention among high-risk and hard-to-reach rural residents. J Psychoactive Drugs. 2003 Oct-Dec;35(4):427-34. doi: 10.1080/02791072.2003.10400489. PMID: 14986871. residents | 2003 | Storytelling and PH | HIV | storytelling | verbal storytelling | no | no | both extract and intervention | probationers | US |  |
| Nowak GJ, Evans NJ, Wojdynski BW, Ahn SJG, Len-Rios ME, Carera K, Hale S, McFalls D. Using immersive virtual reality to improve the beliefs and intentions of influenza vaccine avoidant 18-to-49-year-olds: Considerations, effects, and lessons learned. Vaccine. 2020 Jan 29;38(5):1225-1233. doi: 10.1016/j.vaccine.2019.11.009. Epub 2019 Dec 2. PMID: 31806533. | 2020 | Storytelling and PH | vaccination | story/ies | film | yes | yes | both extract and intervention |  | US |  |
| Ellis JM, Freeman JT, Midgette EP, Sanghvi AP, Sarathy B, Johnson CG, Greenway SB, Whited MC. Sharing the Story of the Cardiac Rehab Patient Experience: A QUALITATIVE STUDY. J Cardiopulm Rehabil Prev. 2019 Jul;39(4):E13-E15. doi: 10.1097/HCR.0000000000000429. PMID: 31241522. | 2019 | Storytelling and PH | cardiovascular health | narratives/narrative accounts | semi-structured interview | yes | no | potential to change KAB |  | US | No short term personal sacrifice/long term population gain |
| 2015. Hammer, Brent A; Vallianatos, Helen; Nykiforuk, Candace I.; Nieuwendyk, Laura M. Perceptions of healthy eating in four Alberta communities: a photovoice project | 2015 | Storytelling and PH | obesity | storytelling | Photovoice | yes | no | extracts information, provides insights |  | Canada | No short term personal sacrifice/long term population gain |
| McDonough S, Colucci E. People of immigrant and refugee background sharing experiences of mental health recovery: reflections and recommendations on using digital storytelling. Visual Communication. 2021;20(1):134-156. | 2021 | Storytelling and PH | mental health | storytelling | digital or video storytelling | yes | yes | extracts information, provides insights | Australia | Australasia | No short term personal sacrifice/long term population gain |
| Bond, C; Chelsea Bond, Mark Brough, Geoffrey Spurling & Noel Hayman (2012) ‘It had to be my choice’ Indigenous smoking cessation and negotiations of risk, resistance and resilience, Health, Risk & Society, 14:6, 565-581, DOI: 10.1080/13698575.2012.701274 | 2012 | Storytelling and PH | smoking | storytelling | personal narrative/s | no | no | extracts information, provides insights | indiginoous peoples, Australia | Australasia | No short term personal sacrifice/long term population gain |
| Dickinson D. Myths, science and stories: working with peer educators to counter HIV/AIDS myths. Afr J AIDS Res. 2011;10 Suppl 1:335-44. doi: 10.2989/16085906.2011.637733. PMID: 25865510. | 2011 | Storytelling and PH | HIV | storytelling | verbal storytelling | yes | yes | both extract and intervention | miming community | Africa |  |
| DiFulvio GT, Gubrium AC, Fiddian-Green A, Lowe SE, Del Toro-Mejias LM. Digital Storytelling as a Narrative Health Promotion Process: Evaluation of a Pilot Study. Int Q Community Health Educ. 2016 Apr;36(3):157-64. doi: 10.1177/0272684X16647359. Epub 2016 May 10. PMID: 27166356. | 2016 | Storytelling and PH | other | storytelling | digital or video storytelling | yes | yes | both extract and intervention | Latinos | US |  |
| Gubrium, A. C; Fiddian-Green, A.; Lowe, S.; Gubrium AC, Fiddian-Green A, Lowe S, DiFulvio G, Del Toro-Mejías L. Measuring Down: Evaluating Digital Storytelling as a Process for Narrative Health Promotion. Qual Health Res. 2016 Nov;26(13):1787-1801. doi: 10.1177/1049732316649353. Epub 2016 Jul 9. PMID: 27184518. | 2016 | Storytelling and PH | reproductive and sexual health | storytelling | digital or video storytelling | yes | yes | potential to change KAB | Latnos | US |  |
| Hartling L, Scott S, Pandya R, Johnson D, Bishop T, Klassen TP. Storytelling as a communication tool for health consumers: development of an intervention for parents of children with croup. Stories to communicate health information. BMC Pediatr. 2010 Sep 2;10:64. doi: 10.1186/1471-2431-10-64. PMID: 20813044; PMCID: PMC2940891. children with croup. Stories to communicate health information | 2010 | Storytelling and PH | other | storytelling | written personal stories | yes | yes | both extract and intervention | parents and children with croup | Canada |  |
| Holdsworth C, Robinson JE. 'I've never ever let anyone hold the kids while they've got ciggies': moral tales of maternal smoking practices. Sociol Health Illn. 2008 Nov;30(7):1086-100. doi: 10.1111/j.1467-9566.2008.01102.x. Epub 2008 Jun 18. PMID: 18564973. | 2008 | Storytelling and PH | smoking | storytelling | in depth interview | yes | no | extracts information, provides insights | Uganda | Africa | No short term personal sacrifice/long term population gain |
| Joshi A. Multiple sexual partners: perceptions of young men in Uganda. J Health Organ Manag. 2010;24(5):520-7. doi: 10.1108/14777261011070547. PMID: 21033646. | 2010 | Storytelling and PH | HIV | storytelling | written personal stories | yes | no | extracts information, provides insights | Uganda | Africa |  |
| Kim M, Lee H, Allison J. Challenges and Lessons Learned From a Mobile Health, Web-Based Human Papillomavirus Intervention for Female Korean American College Students: Feasibility Experimental Study. JMIR Form Res. 2020 Jan 29;4(1):e14111. doi: 10.2196/14111. PMID: 32012036; PMCID: PMC7016616. Form Res. 2020;4(1):e14111. Published 2020 Jan 29. doi:10.2196/14111 | 2020 | Storytelling and PH | vaccination | storytelling | online activity | yes | yes | potential to change KAB | Korean girls | US | Storytelling method too insignificant |
| Koski, K; Holst, J. Exploring Vaccine Hesitancy Through an Artist-Scientist Collaboration : Visualizing Vaccine-Critical Parents' Health Beliefs | 2017 | Storytelling and PH | vaccination | storytelling | storytelling through art | yes | yes | extracts information, provides insights | Netherlands and Finland | Europe | Storytelling method too insignificant |
| Lee S, Yoon H, Chen L, Juon HS. Culturally appropriate photonovel development and process evaluation for hepatitis B prevention in Chinese, Korean, and Vietnamese American communities. Health Educ Behav. 2013 Dec;40(6):694-703. doi: 10.1177/1090198112474003. Epub 2013 Jan 31. PMID: 23372031; PMCID: PMC3830675. | 2013 | Storytelling and PH | cancer | storytelling | theatre/play/performance storytelling | yes | yes | potential to change KAB | Korean, Vietnamese | US | Storytelling method too insignificant |
| Malena-Chan R. A narrative model for exploring climate change engagement among young community leaders. Health Promot Chronic Dis Prev Can. 2019 Apr;39(4):157-166. doi: 10.24095/hpcdp.39.4.07. PMID: 31021067; PMCID: PMC6553576. | 2019 | Storytelling and PH | climate change | storytelling | written personal stories | yes | no | both extract and intervention |  | Canada |  |
| Owen G, Catalan J. 'We never expected this to happen': narratives of ageing with HIV among gay men living in London, UK. Cult Health Sex. 2012;14(1):59-72. doi: 10.1080/13691058.2011.621449. Epub 2011 Nov 14. PMID: 22077645. | 2012 | Storytelling and PH | HIV | storytelling | in depth interview | yes | no | both extract and intervention |  | Canada |  |
| Patten CA, Windsor RA, Renner CC, Enoch C, Hochreiter A, Nevak C, Smith CA, Decker PA, Bonnema S, Hughes CA, Brockman T. Feasibility of a tobacco cessation intervention for pregnant Alaska Native women. Nicotine Tob Res. 2010 Feb;12(2):79-87. doi: 10.1093/ntr/ntp180. Epub 2009 Dec 17. PMID: 20018946; PMCID: PMC2816194.2010en | 2010 | Storytelling and PH | smoking | storytelling as minor part of mixed method study | digital or video storytelling | no | no | both extract and intervention | Saskatchewan | Canada |  |
| Rand, J. R. Building community-based HIV and STI prevention programs on the tundra: Drawing on inuit women's strengths and resiliencies | 2014 | Storytelling and PH | HIV | storytelling | verbal storytelling | yes | no | both extract and intervention | Inuit | Canada | Other (see notes) |
| 8.Rand JR. Inuit women's stories of strength: informing Inuit community-based HIV and STI prevention and sexual health promotion programming. Int J Circumpolar Health. 2016 Dec 9;75:32135. doi: 10.3402/ijch.v75.32135. PMID: 27938640; PMCID: PMC5149655. health promotion programming. Int J Circumpolar Health 2016;75:32135 | 2016 | Storytelling and PH | HIV | storytelling | verbal storytelling | yes | no | extracts information, provides insights | Inuit | Canada |  |
| Teti M, Hayes D, Farnan R, Shaffer V, Gerkovich M. "Poems in the Entrance Area": Using Photo-Stories to Promote HIV Medication Adherence. Health Promot Pract. 2018 Jul;19(4):601-612. doi: 10.1177/1524839917728049. Epub 2017 Sep 9. PMID: 28891334. | 2018 | Storytelling and PH | HIV | storytelling | Photovoice | yes | yes | both extract and intervention |  | US | Storytelling method too insignificant |
| Treffry-Goatley, A; Lessells, R.; Sykes, P.; Barnighausen, T.; de Oliveira, T.; Moletsane, Teti M, Hayes D, Farnan R, Shaffer V, Gerkovich M. "Poems in the Entrance Area": Using Photo-Stories to Promote HIV Medication Adherence. Health Promot Pract. 2018 Jul;19(4):601-612. doi: 10.1177/1524839917728049. Epub 2017 Sep 9. PMID: 28891334. Prevalence | 2018 | Storytelling and PH | HIV | storytelling | digital or video storytelling | yes | no | extracts information, provides insights | Suth Africa | Africa |  |
| Viljoen L, Ndubani R, Bond V, Seeley J, Reynolds L, Hoddinott G. Community narratives about women and HIV risk in 21 high-burden communities in Zambia and South Africa. Int J Womens Health. 2017 Nov 29;9:861-870. doi: 10.2147/IJWH.S143397. PMID: 29238230; PMCID: PMC5716400. | 2017 | Storytelling and PH | HIV | storytelling | third person storytelling/narrative | yes | no | extracts information, provides insights | Zambia and South Africa | Africa | Storytelling method too insignificant |
| Wong JP, Kteily-Hawa R, Chambers LA, Hari S, Vijaya C, Suruthi R, Islam S, Vahabi M. Exploring the use of fact-based and story-based learning materials for HIV/STI prevention and sexual health promotion with South Asian women in Toronto, Canada. Health Educ Res. 2019 Feb 1;34(1):27-37. doi: 10.1093/her/cyy042. PMID: 30496410. HIV/STI prevention and sexual health promotion with South Asian women in Toronto, Canada | 2019 | Storytelling and PH | HIV | storytelling | written personal stories | no | yes | both extract and intervention | South Asian women | Canada | Storytelling method too insignificant |
| Zeelen, J., Wijbenga, H., Vintges, M., & de Jong, G. (2010). Beyond Silence and Rumor: Story telling as an educational tool to reduce the Stigma around HIV/AIDS in rural areas of South Africa. Health Education, 110(5), 382-398. https://doi.org/10.1108/09654281011068531 | 2010 | Storytelling and PH | HIV | storytelling | verbal storytelling | yes | yes | both extract and intervention | South Africa | Africa |  |
| Ashton CM, Houston TK, Williams JH, Larkin D, Trobaugh J, Crenshaw K, Wray NP. A stories-based interactive DVD intended to help people with hypertension achieve blood pressure control through improved communication with their doctors. Patient Educ Couns. 2010 May;79(2):245-50. doi: 10.1016/j.pec.2009.09.021. Epub 2009 Oct 14. PMID: 19833472. | 2010 | Storytelling and PH | 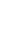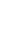   | cardiovascular health | | --- | | storytelling | digital or video storytelling | no | no | potential to change KAB | African American and Caucasian American | US | No short term personal sacrifice/long term population gain |
| Belon AP, Nieuwendyk LM, Vallianatos H, Nykiforuk CI. Community Lenses Revealing the Role of Sociocultural Environment on Physical Activity. Am J Health Promot. 2016 Jan-Feb;30(3):e92-100. doi: 10.4278/ajhp.140428-QUAL-169. Epub 2015 May 14. PMID: 25973966; PMCID: PMC4945163. | 2016 | Storytelling and PH | physical activity | storytelling | Photovoice | yes | no | extracts information, provides insights | Alberta | Canada | No short term personal sacrifice/long term population gain |
| Belon AP, Nieuwendyk LM, Vallianatos H, Nykiforuk CI. Perceived community environmental influences on eating behaviors: A Photovoice analysis. Soc Sci Med. 2016 Dec;171:18-29. doi: 10.1016/j.socscimed.2016.11.004. Epub 2016 Nov 4. PMID: 27863286; PMCID: PMC5241160. | 2016 | Storytelling and PH | physical activity | storytelling | Photovoice | yes | no | extracts information, provides insights | Alberta | Canada | No short term personal sacrifice/long term population gain |
| Bottorff JL, Grewal SK, Balneaves LG, Naidu P, Johnson JL, Sawhney R. Punjabi women's stories of breast cancer symptoms: Gulti (lumps), bumps, and Darad (pain). Cancer Nurs. 2007 Jul-Aug;30(4):E36-45. doi: 10.1097/01.NCC.0000281738.15307.d8. PMID: 17666972.) | 2007 | Storytelling and PH | cancer | storytelling | in depth interview | yes | no | extracts information, provides insights | Punjabi women | Canada | No short term personal sacrifice/long term population gain |
| Bradford LE, Bharadwaj LA. Whiteboard animation for knowledge mobilization: a test case from the Slave River and Delta, Canada. Int J Circumpolar Health. 2015 Oct 26;74:28780. doi: 10.3402/ijch.v74.28780. PMID: 26507716; PMCID: PMC4623287. | 2015 | Storytelling and PH | climate change | storytelling | whiteboard animation | yes | yes | extracts information, provides insights | Northern Canadian communities | Canada | Not public health |
| Caputo A. Exploring quality of life in Italian patients with rare disease: a computer-aided content analysis of illness stories. Psychol Health Med. 2014;19(2):211-21. doi: 10.1080/13548506.2013.793372. Epub 2013 May 7. PMID: 23651424. | 2014 | Storytelling and PH | rare disease | storytelling | written personal stories | no | no | extracts information, provides insights | Italy | Europe |  |
| Colón-Ramos U, Monge-Rojas R, Stevenson TR, Burns H, Thurman S, Gittelsohn J, Gurman TA. How Do African-American Caregivers Navigate a Food Desert to Feed Their Children? A Photovoice Narrative. J Acad Nutr Diet. 2018 Nov;118(11):2045-2056. doi: 10.1016/j.jand.2018.04.016. Epub 2018 Jun 19. PMID: 29934282. Children? A Photovoice Narrative | 2018 | Storytelling and PH | nutrition | storytelling | Photovoice | yes | yes | extracts information, provides insights | African Americans in Texas | US | No short term personal sacrifice/long term population gain |
| Corby NH, Enguídanos SM, Kay LS. Development and use of role model stories in a community level HIV risk reduction intervention. Public Health Rep. 1996;111 Suppl 1(Suppl 1):54-8. PMID: 8862158; PMCID: PMC1382044. | 2016 | Storytelling and PH | HIV | storytelling | role model storywriting | yes | yes | both extract and intervention | California | US |  |
| Cordova D, Alers-Rojas F, Lua FM, Bauermeister J, Nurenberg R, Ovadje L, Fessler K, Delva J, Salas-Wright CP, Council YL. The Usability and Acceptability of an Adolescent mHealth HIV/STI and Drug Abuse Preventive Intervention in Primary Care. Behav Med. 2018 Jan-Mar;44(1):36-47. doi: 10.1080/08964289.2016.1189396. Epub 2016 Jul 15. PMID: 27223646; PMCID: PMC6201193. | 2018 | Storytelling and PH | HIV | storytelling | part of multi-method study | yes | yes | extracts information, provides insights | SE Michigan | US |  |
| Cordova D, Mendoza Lua F, Muñoz-Velázquez J, Street K, Bauermeister JA, Fessler K, Adelman N; Youth Leadership Council, Neilands TB, Boyer CB. A multilevel mHealth drug abuse and STI/HIV preventive intervention for clinic settings in the United States: A feasibility and acceptability study. PLoS One. 2019 Aug 22;14(8):e0221508. doi: 10.1371/journal.pone.0221508. PMID: 31437240; PMCID: PMC6705861. acceptability study. PLoS One. | 2019 | Storytelling and PH | HIV | community participation | part of multi-method study | yes | yes | NA | SE Michigan | US |  |
| Cueva M, Kuhnley R, Revels L, Schoenberg NE, Dignan M. Digital storytelling: a tool for health promotion and cancer awareness in rural Alaskan communities. Int J Circumpolar Health. 2015 Sep 4;74:28781. doi: 10.3402/ijch.v74.28781. PMID: 26343881; PMCID: PMC4561227. | 2015 | Storytelling and PH | cancer | storytelling as minor part of mixed method study | written personal stories | NA | yes | extracts information, provides insights | Alaskan n | US | No short term personal sacrifice/long term population gain |
| Cueva M, Kuhnley R, Revels L, Schoenberg NE, Dignan M. Digital storytelling: a tool for health promotion and cancer awareness in rural Alaskan communities. Int J Circumpolar Health. 2015 Sep 4;74:28781. doi: 10.3402/ijch.v74.28781. PMID: 26343881; PMCID: PMC4561227. | 2015 | Storytelling and PH | cancer | storytelling | digital or video storytelling | yes | yes | both extract and intervention | Alaskan natives | US | No short term personal sacrifice/long term population gain |
| Cueva M, Kuhnley R, Lanier A, Dignan M, Revels L, Schoenberg NE, Cueva K. Promoting Culturally Respectful Cancer Education Through Digital Storytelling. Int J Indig Health. 2016;11(1):34-49. doi: 10.18357/ijih111201616013. PMID: 27429956; PMCID: PMC4943464. | 2016 | Storytelling and PH | cancer | storytelling | digital or video storytelling | yes | yes | both extract and intervention | Alaskan natives | US | No short term personal sacrifice/long term population gain |
| Cueva M, Kuhnley R, Revels L, Schoenberg NE, Dignan M. Digital storytelling: a tool for health promotion and cancer awareness in rural Alaskan communities. Int J Circumpolar Health. 2015 Sep 4;74:28781. doi: 10.3402/ijch.v74.28781. PMID: 26343881; PMCID: PMC4561227. | 2015 | Storytelling and PH | cancer | community participation | digital or video storytelling | yes | yes | both extract and intervention | Alaskan natives | US | No short term personal sacrifice/long term population gain |
| Cueva M, Kuhnley R, Revels L, Schoenberg NE, Lanier A, Dignan M. Engaging Elements of Cancer-Related Digital Stories in Alaska. J Cancer Educ. 2016 Sep;31(3):500-5. doi: 10.1007/s13187-015-0826-z. PMID: 25865400; PMCID: PMC4605835. | 2016 | Storytelling and PH | cancer | storytelling | digital or video storytelling | yes | yes | both extract and intervention | Alaskan natives | US | No short term personal sacrifice/long term population gain |
| Cueva M, Kuhnley R, Slatton J, Dignan M, Underwood E, Landis K. Telenovela: an innovative colorectal cancer screening health messaging tool. Int J Circumpolar Health. 2013 Aug 5;72:21301. doi: 10.3402/ijch.v72i0.21301. PMID: 23930245; PMCID: PMC3736451. | 2013 | Storytelling and PH | cancer | storytelling | film | yes | yes | both extract and intervention | Alaskan natives | US | No short term personal sacrifice/long term population gain |
| Melany Cueva, Regina Kuhnley & Katie Cueva (2012) Enhancing cancer education through the arts: building connections with Alaska Native people, cultures and communities, International Journal of Lifelong Education, 31:3, 341-357, DOI: 10.1080/02601370.2012.683615 | 2012 | Storytelling and PH | cancer | storytelling as minor part of mixed method study | storytelling through art | yes | yes | both extract and intervention | Alaskan natives | US | No short term personal sacrifice/long term population gain |
| Curtis P, Stapleton H, James A. Intergenerational relations and the family food environment in families with a child with obesity. Ann Hum Biol. 2011 Jul;38(4):429-37. doi: 10.3109/03014460.2011.590530. Epub 2011 Jun 17. PMID: 21682573. | 2011 | Storytelling and PH | obesity | storytelling as minor part of mixed method study | in depth interview | no | no | extracts information, provides insights | Northern England | Europe | Storytelling method too insignificant |
| Davis C, Darby K, Moore M, Cadet T, Brown G. Breast care screening for underserved African American women: Community-based participatory approach. J Psychosoc Oncol. 2017 Jan-Feb;35(1):90-105. doi: 10.1080/07347332.2016.1217965. Epub 2016 Aug 11. PMID: 27662263. | 2017 | Storytelling and PH | cancer | community participation | theatre/play/performance storytelling | yes | yes | both extract and intervention | Menphis | US | No short term personal sacrifice/long term population gain |
| Drew SE, Duncan RE, Sawyer SM. Visual storytelling: a beneficial but challenging method for health research with young people. Qual Health Res. 2010 Dec;20(12):1677-88. doi: 10.1177/1049732310377455. Epub 2010 Aug 20. PMID: 20729503. | 2010 | Storytelling and PH | chronic disease | storytelling | Photovoice | yes | yes | extracts information, provides insights | Melbourne, Australia | Australasia | No short term personal sacrifice/long term population gain |
| Egeland, G., Yohannes, S., Okalik, L., Kilabuk, J., Racicot, C., Wilcke, M., Kuluguqtuq, J., Kısa, S., Kuhnlein, H., Erasmus, B., Spigelski, D., & Burlingame, B. (2013). The value of Inuit elders' storytelling to health promotion during times of rapid climate change and uncertain food security. | 2013 | Story and PH | climate change | storytelling | digital or video storytelling | yes | yes | both extract and intervention | Canadian Arctic, Inuit | Canada | Not a primary study |
| Findholt NE, Michael YL, Davis MM. Photovoice engages rural youth in childhood obesity prevention. Public Health Nurs. 2011 Mar-Apr;28(2):186-92. doi: 10.1111/j.1525-1446.2010.00895.x. Epub 2010 Oct 18. PMID: 21732973. | 2011 | Storytelling and PH | obesity | storytelling | Photovoice | yes | yes | both extract and intervention | Oregon | US | No short term personal sacrifice/long term population gain |
| Fitzpatrick AL, Steinman LE, Tu SP, Ly KA, Ton TG, Yip MP, Sin MK. Using photovoice to understand cardiovascular health awareness in Asian elders. Health Promot Pract. 2012 Jan;13(1):48-54. doi: 10.1177/1524839910364381. Epub 2010 Nov 5. PMID: 21057047. | 2012 | Storytelling and PH | cardiovascular health | storytelling | Photovoice | yes | no | both extract and intervention | Iowa | US | No short term personal sacrifice/long term population gain |
| Fletcher S, Mullett J. Digital stories as a tool for health promotion and youth engagement. Can J Public Health. 2016 Aug 15;107(2):e183-e187. doi: 10.17269/cjph.107.5266. PMID: 27526216; PMCID: PMC6972260. | 2016 | Storytelling and PH | other | storytelling | digital or video storytelling | yes | no | both extract and intervention | Vancouver | Canada | No short term personal sacrifice/long term population gain |
| GGreenhalgh T, Collard A, Campbell-Richards D, Vijayaraghavan S, Malik F, Morris J, Claydon A. Storylines of self-management: narratives of people with diabetes from a multiethnic inner city population. J Health Serv Res Policy. 2011 Jan;16(1):37-43. doi: 10.1258/jhsrp.2010.009160. Epub 2010 Sep 6. PMID: 20819914. multiethnic inner city population | 2011 | Storytelling and PH | diabetes | storytelling | verbal storytelling | yes | no | extracts information, provides insights | London, UK | Europe | No short term personal sacrifice/long term population gain |
| Grimes TS, Hou SI. "A Breast Ain't Nothing but a Sandwich": narratives of Ella, an African American social worker breast cancer survivor. Soc Work Public Health. 2013;28(1):44-53. doi: 10.1080/19371918.2010.513667. PMID: 23369021. | 2013 | Storytelling and PH | cancer | storytelling | in depth interview | no | no | extracts information, provides insights | African American breast cancer survivor | US | No short term personal sacrifice/long term population gain |
| Gunnarsson N, Hydén LC. Organizing allergy and being a 'good' parent: parents' narratives about their children's emerging problems. Health (London). 2009 Mar;13(2):157-74. doi: 10.1177/1363459308099682. PMID: 19228826. | 2009 | Storytelling and PH | other | storytelling | in depth interview | no | no | extracts information, provides insights | Sweden | Europe | No short term personal sacrifice/long term population gain |
| Briant KJ, Halter A, Marchello N, Escareño M, Thompson B. The Power of Digital Storytelling as a Culturally Relevant Health Promotion Tool. Health Promot Pract. 2016 Nov;17(6):793-801. doi: 10.1177/1524839916658023. Epub 2016 Jul 8. PMID: 27402721; PMCID: PMC5065376. | 2016 | Storytelling and PH | other | storytelling | digital or video storytelling | yes | yes | both extract and intervention | Yakima Valley | US | No short term personal sacrifice/long term population gain |
| Hartling L, Scott SD, Johnson DW, Bishop T, Klassen TP. A randomized controlled trial of storytelling as a communication tool. PLoS One. 2013 Oct 25;8(10):e77800. doi: 10.1371/journal.pone.0077800. PMID: 24204974; PMCID: PMC3808406. | 2013 | Storytelling and PH | other | story/ies | third person storytelling/narrative | yes | yes | extracts information, provides insights | Emergency departments | Canada | No short term personal sacrifice/long term population gain |
| Houston TK, Allison JJ, Sussman M, Horn W, Holt CL, Trobaugh J, Salas M, Pisu M, Cuffee YL, Larkin D, Person SD, Barton B, Kiefe CI, Hullett S. Culturally appropriate storytelling to improve blood pressure: a randomized trial. Ann Intern Med. 2011 Jan 18;154(2):77-84. doi: 10.7326/0003-4819-154-2-201101180-00004. Erratum in: Ann Intern Med. 2011 May 17;154(10):708. PMID: 21242364. appropriate storytelling to improve blood pressure: a randomized trial | 2011 | Storytelling and PH | cardiovascular health | storytelling | digital or video storytelling | no | no | both extract and intervention | Southern US states | US | No short term personal sacrifice/long term population gain |
| Houston TK, Fix GM, Shimada SL, Long JA, Gordon HS, Pope C, Volkman J, Allison JJ, DeLaughter K, Orner M, Bokhour BG. African American Veterans Storytelling: A Multisite Randomized Trial to Improve Hypertension. Med Care. 2017 Sep;55 Suppl 9 Suppl 2:S50-S58. doi: 10.1097/MLR.0000000000000766. PMID: 28806366. | 2017 | Storytelling and PH | cardiovascular health | storytelling | digital or video storytelling | no | yes | extracts information, provides insights | African American veterens | US | No short term personal sacrifice/long term population gain |
| Høybye MT, Johansen C, Tjørnhøj-Thomsen T. Online interaction. Effects of storytelling in an internet breast cancer support group. Psychooncology. 2005 Mar;14(3):211-20. doi: 10.1002/pon.837. PMID: 15386774. | 2005 | Storytelling and PH | cancer | storytelling | written personal stories | no | yes | both extract and intervention | Denmark | Europe | No short term personal sacrifice/long term population gain |
| Ishida DN, Toomata-Mayer TF, Braginsky NS. Beliefs and attitudes of Samoan women toward early detection of breast cancer and mammography utilization. Cancer. 2001 Jan 1;91(1 Suppl):262-6. doi: 10.1002/1097-0142(20010101)91:1+<262::aid-cncr16>3.0.co;2-r. PMID: 11148591. | 2001 | Storytelling and PH | cancer | storytelling | personal narrative/s | no | no | extracts information, provides insights | Hawaii | US | No short term personal sacrifice/long term population gain |
| Ka'opua LS. Developing a culturally responsive breast cancer screening promotion with Native Hawaiian women in churches. Health Soc Work. 2008 Aug;33(3):169-77. doi: 10.1093/hsw/33.3.169. PMID: 18773792; PMCID: PMC2938766. | 2008 | Storytelling and PH | cancer | storytelling | verbal storytelling | yes | yes | both extract and intervention | Hawaii | US |  |
| Keo, Phalla Duong J ProQuest LLC. Understanding How Participants Become Champions and Succeed in Adopting Healthy Lifestyles: A Storytelling of a Community Health and Nutrition Program at a Land-Grant University | 2016 | Storytelling and PH | nutrition | storytelling | in depth interview | yes | yes | potential to change KAB | Minnesota | US | No short term personal sacrifice/long term population gain |
| Koenigstorfer J. Childhood Experiences and Sporting Event Visitors' Preference for Unhealthy versus Healthy Foods: Priming the Route to Obesity? Nutrients. 2018 Nov 5;10(11):1670. doi: 10.3390/nu10111670. PMID: 30400562; PMCID: PMC6267172. | 2018 | Storytelling and PH | obesity | storytelling | in depth interview | no | no | potential to change KAB | all over | US | No short term personal sacrifice/long term population gain |
| Larkey LK, Gonzalez J. Storytelling for promoting colorectal cancer prevention and early detection among Latinos. Patient Educ Couns. 2007 Aug;67(3):272-8. doi: 10.1016/j.pec.2007.04.003. Epub 2007 May 23. PMID: 17524595. | 2007 | Storytelling and PH | cancer | storytelling | in depth interview | yes | yes | potential to change KAB | Latinos | US | No short term personal sacrifice/long term population gain |
| Larkey LK, Lopez AM, Minnal A, Gonzalez J. Storytelling for promoting colorectal cancer screening among underserved Latina women: a randomized pilot study. Cancer Control. 2009 Jan;16(1):79-87. doi: 10.1177/107327480901600112. PMID: 19078934. | 2009 | Storytelling and PH | cancer | storytelling | in depth interview | yes | yes | potential to change KAB | Latinos | US | No short term personal sacrifice/long term population gain |
| Larkey LK, McClain D, Roe DJ, Hector RD, Lopez AM, Sillanpaa B, Gonzalez J. Randomized controlled trial of storytelling compared to a personal risk tool intervention on colorectal cancer screening in low-income patients. Am J Health Promot. 2015 Nov-Dec;30(2):e59-70. doi: 10.4278/ajhp.131111-QUAN-572. Epub 2015 Jan 23. PMID: 25615708. | 2015 | Storytelling and PH | cancer | storytelling | digital or video storytelling | yes | yes | potential to change KAB | low income patients | US | No short term personal sacrifice/long term population gain |
| LeBron AM, Schulz AJ, Bernal C, Gamboa C, Wright C, Sand S, Valerio M, Caver D. Storytelling in community intervention research: lessons learned from the walk your heart to health intervention. Prog Community Health Partnersh. 2014 Winter;8(4):477-85. doi: 10.1353/cpr.2014.0066. PMID: 25727980; PMCID: PMC4374989. | 2014 | Storytelling and PH | cardiovascular health | story/ies | verbal storytelling | no | yes | potential to change KAB | African American and Hispanics, Detroit | US | No short term personal sacrifice/long term population gain |
| McDonough S, Colucci E. People of immigrant and refugee background sharing experiences of mental health recovery: reflections and recommendations on using digital storytelling. Visual Communication. 2021;20(1):134-156. | 2021 | Storytelling and PH | mental health | storytelling | digital or video storytelling | yes | yes | extracts information, provides insights | Immigrant and migrant in Australia | Australasia | No short term personal sacrifice/long term population gain |
| McIntosh C, Stephens C, Lyons A. "Remember the bubbles hurt you when you cook in the pan": young children's views of illness causality. Psychol Health Med. 2013;18(1):21-9. doi: 10.1080/13548506.2012.687829. Epub 2012 May 29. PMID: 22639797. | 2012 | Storytelling and PH | other | storytelling | written personal stories | yes | yes | extracts information, provides insights | 4 year olds in New Zealand | Australasia | No short term personal sacrifice/long term population gain |
| Moskowitz JT, Wrubel J, Hult JR, Maurer S, Acree M. Illness appraisals and depression in the first year after HIV diagnosis. PLoS One. 2013 Oct 25;8(10):e78904. doi: 10.1371/journal.pone.0078904. PMID: 24205346; PMCID: PMC3808295. Diagnosis | 2013 | Storytelling and PH | HIV | storytelling | in depth interview | no | no | extracts information, provides insights | California | US |  |
| Moyo Z, Perumal J. Perceptions of school principals and experiences of disclosure of teachers living with HIV. Afr J AIDS Res. 2019 Jul;18(2):148-157. doi: 10.2989/16085906.2019.1629469. PMID: 31282300. | 2019 | Storytelling and PH | HIV | narratives/narrative accounts | semi-structured interview | no | no | extracts information, provides insights | Gauteng, South Africa | Africa | Storytelling method too insignificant |
| Nurser, K.P., Rushworth, I., Shakespeare, T. and Williams, D. (2018), "Personal storytelling in mental health recovery", Mental Health Review Journal, Vol. 23 No. 1, pp. 25-36. | 2018 | Storytelling and PH | mental health | storytelling | in depth interview | yes | yes | both extract and intervention |  | UK | No short term personal sacrifice/long term population gain |
| Saini M, Roche S, Papadopoulos A, Markwick N, Shiwak I, Flowers C, Wood M, Edge VL, Ford J; Rigolet Inuit Community Government; Nunatsiavut Government; IHACC Research Team, Wright C, Harper SL. Promoting Inuit health through a participatory whiteboard video. Can J Public Health. 2020 Feb;111(1):50-59. doi: 10.17269/s41997-019-00189-1. Epub 2019 Apr 25. PMID: 31025298; PMCID: PMC7046868. | 2020 | Storytelling and PH | other | storytelling | whiteboard animation | yes | yes | both extract and intervention | Alaska, Inuit | US | No short term personal sacrifice/long term population gain |
| SStreet J, Cox H, Lopes E, Motlik J, Hanson L. Supporting youth wellbeing with a focus on eating well and being active: views from an Aboriginal community deliberative forum. Aust N Z J Public Health. 2018 Apr;42(2):127-132. doi: 10.1111/1753-6405.12763. Epub 2018 Feb 14. PMID: 29442406. | 2018 | Storytelling and PH | nutrition | storytelling as minor part of mixed method study | focus group | yes | no | both extract and intervention | Aboriginal in Austalia | Australasia | No short term personal sacrifice/long term population gain |
| Tang JP, Tse S, Davidson L. The big picture unfolds: Using photovoice to study user participation in mental health services. Int J Soc Psychiatry. 2016 Dec;62(8):696-707. doi: 10.1177/0020764016675376. Epub 2016 Oct 26. PMID: 27798049. | 2016 | Storytelling and PH | mental health | storytelling | Photovoice | yes | yes | extracts information, provides insights | Hong Kng | Asia | No short term personal sacrifice/long term population gain |
| Thomas B, Priscilla Rebecca B, Dhanalakshmi A, Rani S, Deepa Lakshmi A, Watson B, Vijayalakshmi R, Muniyandi M, Karikalan N. Effectiveness of TB sensitization initiatives in improving the involvement of self help group members in rural TB control in south India. Trans R Soc Trop Med Hyg. 2016 Dec 1;110(12):714-720. doi: 10.1093/trstmh/trx006. PMID: 28938052.f self help group members in rural TB control in south India | 2016 | Storytelling and PH | other | storytelling as minor part of mixed method study | theatre/play/performance storytelling | no | no | both extract and intervention | South India | Asia | Storytelling method too insignificant |
| Toussaint DW, Villagrana M, Mora-Torres H, de Leon M, Haughey MH. Personal stories: voices of Latino youth health advocates in a diabetes prevention initiative. Prog Community Health Partnersh. 2011 Fall;5(3):313-6. doi: 10.1353/cpr.2011.0038. PMID: 22080780. | 2011 | Storytelling and PH | diabetes | storytelling as minor part of mixed method study | semi-structured interview | no | no | extracts information, provides insights | Latinos | US | Storytelling method too insignificant |
| Wang ML, Lemon SC, Clausen K, Whyte J, Rosal MC. Design and methods for a community-based intervention to reduce sugar-sweetened beverage consumption among youth: H2GO! study. BMC Public Health. 2016 Nov 9;16(1):1150. doi: 10.1186/s12889-016-3803-5. PMID: 27829397; PMCID: PMC5103444. | 2016 | Storytelling and PH | nutrition | storytelling as minor part of mixed method study | digital or video storytelling | yes | no | both extract and intervention | Massachusetts | US | No short term personal sacrifice/long term population gain |
| Williams-Brown S, Baldwin DM, Bakos A. Storytelling as a method to teach African American women breast health information. J Cancer Educ. 2002 Winter;17(4):227-30. doi: 10.1080/08858190209528843. PMID: 12556061. | 2002 | Storytelling and PH | cancer | storytelling | focus group | yes | yes | both extract and intervention | African American women | US |  |
| Williams L, Moeke-Maxwell T, Kothari S, Pearson S, Gott M, Black S, Frey R, Wharemate R, Hansen W. PA12 Is digital storytelling ka pai for new zealand māori? using digital storytelling as a method to explore whānau end of life caregiving experiences: a pilot study. BMJ Support Palliat Care. 2015 Apr;5 Suppl 1:A23. doi: 10.1136/bmjspcare-2015-000906.72. PMID: 25960498. | 2015 | Storytelling and PH | other | storytelling | digital or video storytelling | no | no | extracts information, provides insights | Maori in New Zealand | Australasia | Other (see notes) |
| Winskell K, Singleton R, Sabben G. Enabling Analysis of Big, Thick, Long, and Wide Data: Data Management for the Analysis of a Large Longitudinal and Cross-National Narrative Data Set. Qual Health Res. 2018 Aug;28(10):1629-1639. doi: 10.1177/1049732318759658. Epub 2018 Mar 20. PMID: 29557295; PMCID: PMC7384251. | 2018 | Storytelling and PH | HIV | narratives/narrative accounts | written personal stories | no | no | extracts information, provides insights | sub-Saharan Africa | Africa |  |
| Greenhalgh T, Collard A, Begum N. Sharing stories: complex intervention for diabetes education in minority ethnic groups who do not speak English. BMJ. 2005 Mar 19;330(7492):628. doi: 10.1136/bmj.330.7492.628. PMID: 15774990; PMCID: PMC554907. | 2005 | Storytelling and PH | diabetes | storytelling | written personal stories | yes | yes | extracts information, provides insights | London, UK | Europe | No short term personal sacrifice/long term population gain |
| Fielden SJ, Chapman GE, Cadell S. Managing stigma in adolescent HIV: silence, secrets and sanctioned spaces. Cult Health Sex. 2011 Mar;13(3):267-81. doi: 10.1080/13691058.2010.525665. PMID: 21049313. | 2011 | Storytelling and PH | HIV | storytelling as minor part of mixed method study | semi-structured interview | no | no | extracts information, provides insights | Adolescents, Canada, Australia,US | Canada | Not public/pt participants |

**Supplementary file 2b: Data extraction form showing characteristics of included studies**

# Table: Characteristics of Included Studies (10 final studies)

| **Study** | **Phenomena of interest** | **Overall design stages of study (inc. extracting info and/or intervention)** | **Methods for data collection (storytelling) and analysis** | **Location** | **Setting/context/culture** | |
| --- | --- | --- | --- | --- | --- | --- |
| DiFulvio GT,. 2016.  Process of storytelling  Intervention (change KAB)  Culturally defined teen Latino girls, specific issue in that defined population ethnic group | Digital storytelling (DST) in vulnerable youth to encourage positive change in sexual health attitudes and behaviours, self-esteem, social support, control of future (so change in KAB): group activity to create and share narrative accounts of life events. Unlike most studies that focus on impact of narrative-as-message on audiences, this study **looks at the impact of the storytelling (own personal stories) *process* on workshop participants as a health intervention.** | 3 x 4-day DST workshop: 1- 10 x nulliparous nor pregnant; 2- 4x pregnant/parenting + 6 non-parenting; 3- 10 x pregnant/parenting. Facilitator – experienced DST.  29 completed DST. | **DST:** Created individual 1 to 3-minute digital story (specifics of the DST workshop format not available (StoryCenter format)  **3 surveys:** pre- survey (baseline), workshop end (day 4), each participant was given a postsurvey to complete. 3 months follow up. Survey data included demographics.  **Survey categories:** Self-Esteem measured, Social Support Survey, Empowerment (contains self-efficacy, power/powerlessness, community activism and autonomy optimism and control over the future, and righteous anger. The Brief Sexual Attitudes Scale to measure sexual attitudes and behaviour.  **Workshop evaluation:**  standard evaluation tool for ppts experiences in workshop.  **Descriptive statistics** to characterize the study population. Age, birth place, year migrated from Puerto Rico to the mainland US (if applicable), marital status, education, race/ethnicity, employment status, sexual orientation, parenting status, and living situation. Mean calculated. Statistical differences across the 3 survey scales.  **Qualitative data analysis** assesses open responses from the workshop evaluation form. Open coding the responses to develop initial categories; categories grouped to identify three core themes. | MA, US. mid-sized New England city | mid-sized New England city (here called the City) Puerto Ricans. 45% of the population in the city is Puerto Rican, represent- ing the highest percentage of Puerto Ricans living in any mainland U.S city. among all cities in the state with populations of greater than 40,000,23 an average annual rate of HIV diag- nosis double the state rate, and a rate of Chlamydia infection among teens 3.5 times the state rate. | |
|  |  |  |  |  |  | |
|  | **Participant characteristics and sample size** | | **Description of main results** | | | |
|  | Puerto Rican Latinas. Ages of 15 to 21. Study sample comprised 16 non-parent and 14 parent or pregnant Latinas. Mean age 17 yrs. The vast majority of the participants were born in US (83.3%). Approx. 70% were in high school. One (3.3%) married and 4 (13.3%) employed. Nearly half (43.3%) lived with one parent. Over a third (36.7%) of those with children lived with their children. Four (13.3%) participants reported being bisexual and two (6.7%) identified as lesbian. | | **Over 3 survey time points**, ppts experienced greater positive social interactions (pre to follow up(p=0.076)); under empowerment, Optimism and Control over the Future increased from pre to post surveys (p=0.093). Attitudes toward enjoying physical sex decreased 3-month post intervention (p=0.097)  **Workshop evaluation:** 3 themes of positive impact of workshop on ppts (a) truth telling (refers to confronting dominant circulating narratives and revising misrepresentations), (b) KEY FINDING affective impact of storytelling (moving someone emotionally by telling and listening to others stories and having their stories listened to), and (c) empowerment (reported feeling “confident,” “strong and respected,” “optimistic,” “accomplished”, “able to speak up”). All contributed to development of social support and social ties.  **POINTS OF DISCUSSION Benefits of the process of DST** one of first study to **assess benefits of the DST process for workshop** ppts. No changes in self-efficacy, self-esteem, or attitudes in sexual behaviour or attitudes as a result of the workshop. STORYTELLING PROCESS WAS USEFUL: positive feelings associated with telling their own story in a group setting and in particular, having the opportunity to have their voices heard SPEAKS TO THESE MARGINALISED GROUPS OFTEN BENEFIT FROM ST MOST – often adversely impacted by racist, sexist, and classist media and messaging SPEAKS TO THE SIGNIFICANCE OF SPECIFIC CONTEXT AND CULTURE FOR SUCCESS OF ST PROCESS. Constructing a coherent narrative through individual expressive writing gives participants a SENSE OF CONTROL over their health or experience to create goals for the future, resulting in improved overall health as well as a sense of collective control (see quant results – improved control and optimism for the future). SOLIDARITY: process created feel connected to others and less socially isolated in their experiences, and translates to larger collective experience of marginalization so that individuals do not blame themselves for their own hardships. INCREASED SOCIAL CONNECTION (QUAL DATA), which was captured by the subscale of positive social interactions. EMPATHY: link between listening to the other participants’ stories and increased understanding of and empathy for others. PRIDE IN CREATING A STORY: a sense of accomplishment and pride after sharing their story and self-efficacy (qual data show this not the quant). **QUAL results more revealing. POINT about qual results ore meaningful with small sample (vs. quant- sample not large enough).** | | | |
|  |  |  |  |  |  | |
| **Study** | **Phenomena of interest** | **Overall design stages of study (inc. extracting info and/or intervention)** | **Methods for data collection (storytelling) and analysis** | **Location** | **Setting/context/culture** | |
| Gubrium AC,. 2016.  Process of ST  Intervention (change KAB)  Group of high poverty, socially deprived.  Qual vs. quant data. Qual drills down and works especially well in sub-populations with high ethnicity, cultural definition  Only study to look at trustworthiness or validity of qual data | DST scientifically Evaluated the effect of the PROCESS of DST. | Study aimed at assessing the process of individuals creatively identifying, producing their own digital stories has not been well assessed as a mechanism of health behavior change. Examines findings from a 2-year, mixed-methods study that focuses on the effects of the DST process on workshop participants.  Does DST process affect ppts’ self-esteem, sense of empowerment, social sup- port, or sexual attitudes or behaviors?  3 x 4-day DST workshop (as Difulvio, 2016) | Ppts wrote their story about an important experience, images to visually narrate, and learning basics of digital editing software. Part 2) The group process of story circle and story screening. Ppts share the story in a story circle, receive structured feedback; group discussion around the content of each story; facilitators help with script-editing, collection of images, in an iterative process of storytelling; story screening on the last day of the workshop. REFLEXIVITY IMPT. Observations in field notes so researcher interpretations or reflections were kept separate from descriptive material. Debriefs to discuss perspectives. Mixed-methods evaluation of the effect of the DST process on project participants. **Descriptive statistics as for Difulvio 2016 (same study)** Quantitative scales as for Difulvio.  **3 surveys** (Difulvio, 2016). Survey measured effects of the DST wksp Ethnographic approach (4 observers) to qual assess the effect of the DST process on ppts. | US, New England | “The City,” a post-industrial, New England city challenged on numerous sexual and reproductive health with highest teen birth rate (age 15–19) among all cities in NE. Adolescent (<15) birth rate 4 x greater than rate in Massachusetts. Priority to increase access to sexual health services and reduce the high teen pregnancy. | |
|  | **Participant characteristics and sample size** | | **Description of main results** | | | |
| DST provides a voice to these ppts who often feel shamed into silence (part of process of changing KABs)  Solidarity and support not often found in these groups  No long-term follow up. Only 3 mths after end of workshops.  . | LATINO VERY DISADVANTAGED POPULATION: 46.3% of the population lives below 200% poverty (family of 4 under $50,000 approx), and 51% of households are single-parent homes living below 100% poverty. Unemployment rate >31%, and the annual per capita income for Latina/os in the City is $7,757.  Inequity is further accentuated for City youth: 42% of youth <18 in poverty. Puerto Rican Latina; (b) living or receiving services in the project city; (c) being between the ages of 15 to 21, and pregnant, parenting, or neither. 18+ years. | | Quantitative data captured few positive changes associated with the DST process but qual findings suggest positive, health- bearing benefits of the DST process on participants. SHOWS THE VALUE OF QUAL DATA VS. QUANT BUT ALSO RELATED OT STUDY DESIGN. SO SMALL SAMPLE, TOO SMALL FOR MEANINGFUL QUANT. VALIDITY DISCUSSED To optimize trustworthiness of the qualitative results (Lincoln & Guba, 1985), we considered a variety of data eg sources: transcripts of key DST activities, individual interviews with participants, field notes from workshops, and workshop evaluations.  GOOD COMPLIANCE 86.7% completed all three data points. IMPROVED SOCIAL, OPTIMISM AND CONTROL OVER FUTURE, AND ENJOYMENT OF SEX BUT QUANT FDGS LIMITED AND SUGGEST NO EFFECT. Moderate effect sizes were detected for positive social interaction and positive optimism but no detectable effect size was noted for instrumentality, indicating no true change in this measure.  QUAL FDGS MORE POSITIVE: benefitsof the DST process, substantiating our claim that the DST process positively affects ppts, and 'has tremendous potential as a mechanism for health promotion.'  See Difulvio for breakdown qual findings. 4 THEMES EMERGED A) truth telling, (b) sense making, (c) social support, and (d) feeling valued. PREGNANT WOMEN OR THOSE WITH CHILDREN: REVEALED SHAMING AS TECHNIQUE AND MARGINALISING Disciplining- through-shame those young women deemed “at risk” for teen pregnancy, shaming actively reinforces a silencing among the already marginalized. DST workshop afforded time and space to create stories that “talked back” through truth telling they transcend discourse of shame. QUTES: I think I gotta start telling the truth about me and my son, what’s going on. ‘Cause I just be like, “Lie to people about your problem” . . . [but] it’s not really gonna get you nowhere. DST process as helping to break the protective “shell” of untruths they have told about themselves and others.  OTHERS' STORIES PROMPT PPL TO TELL STORIES PREVIOUSLY TOO SHY TO TELL. Through DST process ppts served as participant observers of their own lives as they reflected on personal and social memories and made sense of their lived experiences.  Truth telling also involved creating a digital story in solidarity with other group members, as a way to collaboratively address difficult/traumatic experiences.  REFLEXIVITY OF ppts on their past behaviour stimulated by DST PROCESS: one expressed remorse at distancing herself from her friend, not supporting her, and stigmatizing her for her pregnancy, through a reflexive process in the DST workshop.  SOCIAL SUPPORT FOUND VIA DST DST workshop created a space for acceptance as participants gradually began sharing their experiences over the 4 days, overcoming fears of social stigma. Felt cared for by being listened to. DST process - also non-verbal shows of sppt. ‘A tap on the shoulder, a smile, a nod,’. | | | |
|  |  |  |  |  |  | |
| **Study** | **Phenomena of interest** | **Overall design stages of study (inc. extracting info and/or intervention)** | **Methods for data collection (storytelling) and analysis** | **Location** | **Setting/context/culture** | |
| Hartling L, 2010.  Only study where stories had clear end goal that included distribution and use of stories as booklets.  Process: Variety of storytelling production. Here stories were created and final product (booklet).  Not individuals telling of their personal stories but writer interviews and writes. (So doesn’t explore process of storytelling for the ppt and how it makes them feel) ANOTHER MEANS OF ARRIVING AT END STORY OF A PERSONAL STORY BUT TOLD BY PROFESSIONAL. | Croup, chosen due frequency of its presentation to the emergency department (ED), and the anxiety that it causes for parents. | Intervention.  Designed as story-based intervention for delivery of health evidence to parents of children with croup in order to affect parental and child outcomes, and healthcare utilization.  Description of story-based intervention and receive parental feeÅdback. | A creative writer interviewed parents of children with croup presenting to pediatric ED and drafted stories (sequence of events from time onset of symptoms to post-ED follow-up, to elicit parents’ emotional reaction to the experience and perspectives on the ED management of their child). Revised stories based on written ppt feedback and incorporated research evidence and health information. Illustrator and graphic designer developed story booklets which were evaluated through focus groups. Phone follow up 10 days after ED visit.  5 stories by the creative writer were reviewed and amalgamated into 3. Iterative revision of stories – with expert in creative non- fiction, and reviewed for clinical accuracy by 3 ED physicians 1 nurse. Paper story booklets produced to be given to parents in the ED.  3 story booklets were examined by focus groups of parents (kids who had had croup -age 3-12 yrs) for presentation, interest, style, and clarity. Questions e.g., length, writing style, accuracy, illustrations) to provide stimulation for discussion. | Alberta, Canada | Storytelling in context of pediatric ED (setting parental anxiety high uncertainty of expectation disease management, intense pace of ED). Context of ED is very specific. | |
|  |  |  |  |  |  | |
|  | **Participant characteristics and sample size** | | **Description of main results** | | | |
| This process brought in medical reviewers – interesting and different because it can conflict with the actual story told by a parent. Story was edited to reflect medical accuracy. POINT OF CONFLICT (taking story for the good and bad as it is ie authentic)  Ease of relating to characters.  Study looked at end role of the stories | Child patient 3 months-6 yrs, clinical diagnosis of croup. Parents 18 + years, English spkg. | | Aimed to characterize different experiences. Sentences awkward. Parents feedback included stories that largely engaged due to the ability of the writer to c**apture the parents’ emotions.** Reviewers generally wanted as much information as possible about medical procedures and practices and considered the stories to be an excellent potential source of medical advice for parents. CONFLICT BETWEEN AUTHENTIC STORY AND MEDICAL INFORMATION. Results focus groups were categorized 1) general perceptions of stories; 2) content and emotional by-products of the stories; 3) preferences; and, 4) graphics, layout, and illustrations. Used illustrations to broaden appeal to kids and parents (practical aim of story book ie aid explaining to their child what may happen in the ED. One story was preferred by parents –EASE OF RELATING to this story; in their words, they saw themselves in the story. First-person mode held their attention. Having father as the main character in one of the stories and the fact that he accessed the internet for information (easy to relate to character) ENSURE VERY CLEAR PURPOSE FOR THE STORYTELLING INTERVENTION AT OUTSE. Authors say this is critical not only for the development of the interventions, but also to evaluate their effectiveness. eg pur-pose of the intervention should be directly related to the outcomes to be assessed in its evaluation.  STAYING TRUE TO STORY VS.EVIDENCE BASED INFO: eg.. in one case the child was given an X-ray, despite the fact that this is not standard practice for croup and does not conform to accepted clinical practice guidelines. In the end, they did not include the X-ray account and aimed to make the stories reflect typical cases of mild, moderate, and severe disease and how they would be managed on average. THIS IS DEBATELBE COS NOT TRUE REFLECTION OF TE STORY TOLD.. They included example of misdiagnosis of asthma for croup. ILLUSTRATED THAT ERROR IN DIAGNOSIS HAPPENS.GOOD EXAMPLE CONFLICT MEDICAL EVIDENCE PRACTICE VS REAL LIVED EXPERIENCE. HOW MUCH MEDICAL INFO TO INCLUDE IN STORY? Lots in these booklets (considering aim) (e.g., signs and symptoms, what is a steroid, how.. GENERALISABILITY: A major challenge was developing stories that would be widely generalizable and appealing – BALANCE INFO LEVEL AND READABILITY. | | | |
|  |  |  |  |  |  | |
| **Study** | **Phenomena of interest** | **Overall design stages of study (inc. extracting info and/or intervention)** | **Methods for data collection (storytelling) and analysis** | **Location** | **Setting/context/culture** | |
|  |  |  |  |  |  | |
| Joshi A. 2010.  SENSITIVE TOPIC HOW TO APPROACH: Used writing rather then speaking to tell the story. Overcomes inhibition. Used proxy character of young man the same age. Removed inhibitions of young people in front of peers discussing their own sensitive issues.  HIGHLY NUANCED BEHAVIOUR STEMMING FROM BEING TAUGHT GENDER ROLE AT YOUNG AGE (FATHER, HUSBAND) AND THIS SHAPES SEXUAL BEHVR. | Perceptions of young men in Uganda regarding their sexual behaviour (sex with multiple sexual partners) considered high risk wrt HIV, specifically at engaging with multiple sexual partners (MSP). | Writing personal stories and acted in dramas  Extracting information on drivers of MSP | Ppts played an attribute-ranking game, wrote personal stories and acted in small dramas as part of the data collection.  Participatory Action Research (PAR) involves researchers and participants working together to examine a problematic situation to change it. Participant-determined stories create memory, emotion, meaning, and personal and collective identity. Story *writing* their own experiences and knowledge, without the pressure of speaking out loud in front of their peers.  Used proxy character – young man their age, called Musa. Musa finds Jane very attractive, and wants her to be his girlfriend. How would he approach Jane? What would he say? Where would he take her? What would he tell his friends? How does he feel?  Drama also used as method to invoke stories, participant-determined drama was used. Prepared and act out a script highlighting the challenges of beginning a new relationship with a young woman. | Eastern Uganda, rural village of Buwunda in the Kamuli district, | Six 15-19 year old school-going young men in rural Uganda 15-to-24 age-group.  Area has highest rate of HIV infection in Uganda, similar throughout sub-Saharan Africa (SSA). Most research in this field is on the vulnerabilities of young women and less on one of the causes of some of their vulnerabilities, that is, young men.  At early age, young men in Uganda are taught specific behaviours and skills according to the role they are expected to play as husbands and fathers. These gender norms and expectations shape a young man’s sexual scripts. Very high rate of polygymy and poor secondary education. | |
|  | **Participant characteristics and sample size** | | **Description of main results** | | | |
| This study clearly demonstrates how storytelling uncovers cultural influences ie ignorance of health risks small part, gender assertion.  The storytelling experience reveals keys to unlocking health issues via power dynamics.  Shows how using an alias or proxy character can help if the subject matter is close to home and people find it embarrassing to discuss their own personal story. | Six 15-19 year-old school-going young men in rural Uganda | | BY INGORNANCE OF HEALTH RISKS only partly explains risky sexual behaviour. Young men find it asserts their gender identity, expresses their masculinity, and this outweighs the risks. Young men preferred younger girls as their main girlfriend as they were thought to be more submissive, and they would “listen more to you”. Or the challenge of older, more sophisticated, urban young women, with the idea that the reward would be greater in terms of sexual satisfaction, reputation with peers, and sexual experience. Ultimately it was all aimed at sexual experience to “know what to do on their wedding night”. Social norms. GENDER NORMS EXPRESSED THROUGH PRACTISING MSP AND RELATE TO POWER DYNAMICS: gender norms of males, by aspiring to be the provider, the one in charge, the decision maker , expressed via gifts as a symbol of possession and the preference of a younger, more submissive girl as a main girlfriend.  HEALTH RISKS SECONDARY - MORE INPORTANT IS CHALLENGE TOO POWER AND CONTROL: young men identified the risks of MSP to be less associated with the adverse health consequences and more about the things which challenge their pleasure, experience and control seeking behaviours, e.g. money, manipulative girls, and parental control and masculinity.  Indicates CHANGE THE POWER DYNAMICS TO IMPROVE HEALTH: change gender norms as seen in HIV studies.  CONDOMS AND IMPORTANCE TO HEALTH MENTIONED BY YOUNG MEN: but their own sexual pleasure was considered more important. Storytelling came up with ways to make condom use more attractive eg For example, hiding a condom on the body and asking the partner to find it, or wrapping condoms as a gift and presenting it after a romantic meeting.  IN CONCLUSION: having a greater understanding of young men’s perspectives provides a significant opportunity to augment HIV prevention strategies. | | | |
|  |  | |  | | | |
| **Study** | **Phenomena of interest** | **Overall design stages of study (inc. extracting info and/or intervention)** | **Methods for data collection (storytelling) and analysis** | **Location** | **Setting/context/culture** | |
|  |  | |  | | | |
| Malena-Chan R. 2019.  Nature of the topical issue is similar to AMR. Here, the realities of the issue in terms of the structural and social realities of daily life.  Context and culturally-based issues need exploration. Storytelling is suited to this.  Ganz’s theories about public narrative to interpret perceptions of climate change.  Different approach- journaling thoughts on the personal experience.(different | Exploring context and culturally-based perceptions and interpretation, and capture contextual and cultural barriers of climate change as it relates to everyday events.  ﻿﻿Modelling narrative dissonance highlights opportunities to frame the challenges, choices, and outcomes related to climate change in a way that mobilizes population health stakeholders.. | Qualitative study has employed narrative methodology to interpret the meaning of climate change. Study designed to inform a framework to overcomes barriers to engagement and examines how climate change is experienced within the structural and social realities of daily life. The study aims to understand how to bridge the gap between knowledge and action. | Climate change is a multidimensional problem.  Semi-structured 1 hr interviews, then each ppt given a journal with 5 research questions to document thoughts about their personal story as they emerge over following weeks. These were added to the story in follow up visits.  Thematic analysis: Narrative patterns and relationships between core themes within participant plots identified.  Participant narratives were considered together, and individually until interpretations about their perceptions emerged clearly. Examples of narrative dissonance and narrative fidelity were identified. Marshall argued that it is a key element in mobilizing action to address climate change, because only by offering a more more compelling story will faulty interpretations of climate change be abandoned. | Saskatchewan, Canada  Community leaders, Saskatoon, Saskatchewan, Canada (age 20–40 years) | Disagreement among stakeholders about the future of Canada’s climate policies (2019). Need to use eco-social lens to understand health issues related to climate change. | |
|  |  |  |  |  |  | |
|  | **Participant characteristics and sample size** | | **Description of main results** | | | |
| About mobilising knowledge into emotion into collective action via story.  This study shows how barriers manifest within narratives of people who accept climate science.just raising awareness is not enough to increase community capacity.  Storytelling and sharing – a means of articulating difficult emotions about climate change and elicits feelings of **solidarity**. **(Storytelling and solidarity. THEME)**  A narrative lens captures the complexities surrounding personal and public realms, the nuance of emotional and moral reasoning, and the contingencies that characterize the context in which mobilization occurs. (Parallels with AMR – awareness but action even amongst those that know)  **Storytelling reveals that information deficit model is unproductive.** | Age 20-40 years (n = 10).  Community leaders in Saskatoon, Saskatchewan, Canada. | | Identifies key POINTS OF NARATIVE DISSONANCE, MOBILISING MOMENTS AND NARRATIVE FIDELITY ACROSS DIFFERENT EXPERIENCES TO BUILD A MODEL of how to frame individuals challenges, choices, and outcomes related to climate change.  Moments identified -opportunities for transforming **knowledge into emotions** that mobilize collective action. Manifestations of narrative dissonance and narrative fidelity within participant narratives found: a narrative model of engagement has illuminated a pathway from knowledge to action, outlining stumbling blocks as well as strategies for overcoming them along the way.  Experiences categorised into a model comprised of experiences of agency, responsibility, capacity, and activation. E**.g. Agency/role:** I'’ve noticed that sometimes if I see a headline or a snippet of some depressing news about the climate and I’m having a bad day, I will consciously tell myself ‘I can’t afford to look at this right now’ and I will skip past the news…I think my avoidance is part self-care but also part unhealthy wilful ignorance…' (narrative dissonance). ‘So that’s sort of how I would characterize my climate change lens, I guess; it’s constantly in the background of everything else that meanwhile everything is burning (mobilising moment);' I feel like I need to challenge those emotions and … I guess, be more rational – like what do I need to do? How do I get people’s attention, how do I engage people back home?' (narrative fidelity).  Regarding capacity (**narrative dissonance between responsibility and capacity**): ppts perceived a decreasing capacity to address climate change with each generation to come, paradoxically inverse to the responsibility to act to address climate change, which can only increase over time.  Sharing stories could be a means of bringing difficult emotions about climate change to the surface and could elicit feelings of **solidarity**.  By determining narrative dissonance in the ppt stories there is guide to potentially improving engagement barriers of knowledgeable, motivated people (ie not the non-believers but those who are very aware of climate change but find it hard to act), and a narrative lens captures the complexities surrounding personal and public realms, the nuance of emotional and moral reasoning, and the contingencies that characterize the context in which mobilization occurs.  Study shows that **information deficit approach to climate change engagement may not translate into strategic, well-resourced plans for urgent and disruptive systems change**. | | |
|  |  |  |  |  |  | |
| **Study** | **Phenomena of interest** | **Overall design stages of study (inc. extracting info and/or intervention)** | **Methods for data collection (storytelling) and analysis** | **Location** | **Setting/context/culture** | |
| Rand JR, 2014. | Sexual health and HIV of Inuit women and families.  Primary researcher had lived in community >15 yrs. Immediate familiarity with culture and customs. Facilitates storytelling process. | Aims to inform relevant programming and policy.  Extracting information | Indigenous methods drawing on framework of Two-Eyed Seeing, and using storytelling sessions to gather data.  9 storytelling sessions took place with 21 Inuit women.  Storytelling sessions were audio recorded and transcribed and collaborative thematic analysis over 3 groups of 13 ppts.  The study sought to explore the women’s perceptions of STIs/HIV in their community? What women think would benefit the community in regard to sexual health; and what women think are the determinants that influence sexual health of women and communities?  REFELXIVITY OF KEY RESEARCHER Primary researcher was of settler ancestry and lived in community > 15 years. Well-established, long-term relationship was key strength.  COMMUNITY BASED PARTICIPATORY RESEARCH (CBPR) design. Grounded in local realities, and uses a theoretical framework relevant to participants (Two-Eyed Seeing - a guiding principle for bringing together multiple worldviews and bridged between the Innuit way of knowing and the academic one).  Storytelling sessions began with semi-structured, guiding questions, that prompted sharing stories related to STIs and HIV in their community (eg influences on sexual health (STI/HIV) of women, what health promotion would work STI and HIV prevention.  Sessions 30 to 75 min. Groups of one to 6 ppts. Atlas.ti. roughly coded data. | Canadian Arctic | Canadian Arctic.  Often there are obstacles to accessing sexual health services due to geographic location and cultural and linguistic obstacles.  Some STIs 10 x higher prevalence than elsewhere in Canada. Colonization and westernization have been identified in the literature as contributing to the high rates of STIs within the Arctic.  A need for ongoing Inuit-specific sexual health promotion, education, disease prevention and care programs. | |
|  | **Participant characteristics and sample size** | | **Description of main results** | | | |
| IMPORTANCE OF CULTURAL RESPECT Essential to include elders in research process – they have a central role in community. They ensure respect and indigenous ways of knowing are upheld in research.  Innuit community demonstrates th strength of storytelling in culturally refined environment. | 21 Inuit women aged 18to 61 years, and families. Education from primary to university. Unemployed to employed.  Sample recruited via community programs, a feast and information session, Facebook, adverts in grocery stores. | | Five major themes and 27 subthemes emerged from data analysis of stories. 5 major: the way it used to be, change, family, intimate relationships and holistic strategies. Historically, STIs such as HIV did not exist among Inuit, thus Elders (key ‘teachers’ in the community could not teach about it.  ESSENTIAL TO INCLUDE ELDERS IN RESEARCH PROCESS: IMPORTANCE OF CULTURAL RESPECT Essential to include elders in research process – they have a central role in community. They ensure respect and indigenous ways of knowing are upheld in research.  ALCOHOL USE: most salient subtheme emerging from all 9 storytelling sessions. Stories of alcohol related to what women considered contributed to negative sexual health outcomes and STIs. The women emphasized that any sexual health programming/promotion needed to include discussions around alcohol use and sexual decision-making.  CHILDREN'S EDUCATION AND FAMILY FOCUS SEXUAL HEALTH EDUCATION: Woven through many women’s stories. ‘I tell my children’ was a key sub-theme. Although most women did not learn about sexual health from their parents, they stressed that they are committed to educating their own children. This theme emphasizes the family focus of sexual health programming.  INTIMATE RELATIONSHIPS: a theme throughout women’s stories about their observations of other people’s relationships, as well as their personal experiences. Use of role models is in line with Inuit ways of learning through observation.  STORYTELLING HELPS IDENTIFY GROUPS HARD TO REACH e.g. youth not attending school, and men who lack the community programming and social networks that women have through sewing groups and prenatal groups.  INUIT WOMEN ARE EXPERTS IN THEIR OWN LIVES: Their ideas and insights need to be sought as the basis for STI and HIV prevention and sexual health promotion programming.  STORIES link up the daily lives and epidemiology. NEED TO ADDRESS sexual health on the ground in the community. | | |
| **Study** | **Phenomena of interest** | **Overall design stages of study (inc. extracting info and/or intervention)** | **Methods for data collection (storytelling) and analysis** | **Location** | **Setting/context/culture** | |
|  |  |  |  |  |  | |
| Treffry-Goatley A, 2016.  Underserved, minority communities responded well to recruitment to DST.  Storycenter 7-step process. Established DST process used in other studies.  Illustrates a carefully planned process with full participation. Lots new skills for ppt to learn. | Overcoming challenges to adherence to antiretroviral therapy (ART) to prevent and treat HIV/AIDS among patients living in resource-limited settings in sub-Saharan Africa. Part of issue is the development of drug resistance as linked to poor adherence. | Data extraction using DST.  To understand reasons and drivers (specific individual and structural factors) of adherence to ART in low resourced, rural community South Africa.  To identify specific individual and structural factors that can either challenge or support adherence in this context | Analysis of 20 digital stories to elucidate the experiences, understandings, and contexts of participants, and to identify potential barriers and facilitators for those.  DST is relevant with minority and underserved communities and can encourage individuals to take part in research on local health issues.  Advertised workshop about ART called ‘Adherence Stories’ at primary healthcare clinics and recruited a convenience sample of attendees. All HIV status included.  96 applied and 20 selected.  2 DST workshops held.  **Day 1**: Introduced concept of DST showed relevant digital stories and explained the workshop process.  Workshop involved ‘seven step’ process taught by the StoryCenter: Story circle - each ppts has 20 min to share story.  Photography: tutorial use their own photographs in storytelling, for direct illustration/abstract metaphor; techniques for making anonymous portraits e.g. silhouettes or extreme close-ups.  Afternoon writing 200-300 words story.  **Day 2:** art workshop. Used photos. Read scripts with ppts and approved. Audio recording. Collage of images and excerpts on cardboard. Facilitators physical editing (not digital) due technical issues and literacy.  **Day 3:** edited with individual ppt - add photos, art etc, soundtracks. DST 1-3 min approx..  **Day 4:** signed release form, shared and discussed stories.  Narrative analysis techniques (used for variety of texts) to interrogate the text, music, photographs and original artwork used in the digital stories.  Applied thematic analysis to find common thematic elements across ppt stories.  Nvivo Qualitative Data analysis software used for inductive analysis. | A rural district of KwaZulu-Natal, South Africa | HIV pts in resource-limited (poverty, high unemployment) settings in sub-Saharan Africa.  HIV prevalence high (29% in the adult population aged 15– 49) | |
|  | **Participant characteristics and sample size** | | **Description of main results** | | | |

| Study ‘validates’ using triangulation with drawings and music. NOT MANY WAYS TO VALIDATE IN THESE STORYTELLING STUDIES SO THIS ATTEMPT IS INTERESTING  Comparison to weaponary – cultural reference.  Illustrates uncovering of underpinning influences on adherence eg stigma, disclosure, traditional healing.  Reveals how adherence to ART intersects with everyday behaviours, daily routines etc. It takes the central issue out of isolation. (somewhere between structured interviews/questionnaire and ethnography)  Ppts parallel and use metaphor with other significant events I their lives e.g. apartheid  Some stories enforce already established understandings e g. stigma’s role in impeding adherence. | 19 workshop attendees. Black South African. 1 man. HIV pts. Zulu spoken. | | Thematic analysis and triangulated results with visual and musical study of participant drawings and soundtracks to provide insight into ppt experiences of HIV and ART adherence. Revealed six overlapping and non-exclusive themes.  THEMES: Adherence: Treatment is for life 15 of the stories addressed the general topic of adherence, eleven of which were specifically about ART. E.g. ART described as **weaponry** to protect an individual against HIV: ‘I took responsibility when the time came for me to take treatment. When I came back with my treatment, I told the virus that it could never defeat me. I also told myself that I am armed now and I am not going back’ (woman, aged 53).  Illness and death: BLACK NATION DYING. 11/20 stories describe death of one or more family members.  HEALTH SEEKING BEHAVIOUR AND EXPERIENCE: HIV facing the fear.  STIGMA: Beware! She might infect us.  DISCLOSURE: Disclosure is not easy.  SOCIAL SUPPORT: we help each other.  FEAR OF SIDE EFFECTS: stories about HIV speak about starting ART, including the fear of possible side effects e.g.’My mother said I should wait a bit [before starting treatment]. She told me there is traditional concoction that she will look for.’  FREEDOM TO DISCUSS BROAD RANGE TOPICS : Storytelling in this context is a sensitive process and we did not want to coerce participants into sharing more than they were comfortable with.  INSIGHT INTO EVERYDAY: reveals how adherence intersects with the everyday lives of people, rather than learning about it in isolation.  CO-MORBIDITIES: suggestive that HIV needs to be understood in the context of co-morbidities.  CULTURAL REFERENCE IS STRONG IN STORYTELLING: APARTHEID: A belief in HIV medication is echoed in the armed struggle subtheme, which alludes to the historical fight against Apartheid. Since Apartheid was overcome after many years of black resistance the reference to the resistance movement in this context can be seen to imply that in the context of ART, HIV can be overcome. SOCIAL DESIRABILITY: ppts aware that the centre supports Western medicne so stories might have leant towards this and away from faith healers for this reason. PAST BAD EXPERIENCES AND MEDIA: bad media stories about HIV transmission through breastfeeding influences ppts still.  DRAWINGS OF ACCESS CLINICS ON FOOT: show that this could limit access to testing, treatment and care.  STIGMA STILL PREVALENT: numerous accounts of dishonesty around HV, testing fear ,and individuals who failed to disclose. Stigma is key impediment to the success of long-term treatment and these narratives provide personal evidence. | | |
| --- | --- | --- | --- | --- | --- |
|  |  |  |  |  |  | |
| **Study** | **Phenomena of interest** | **Overall design stages of study (inc. extracting info and/or intervention)** | **Methods for data collection (storytelling) and analysis** | **Location** | **Setting/context/culture** | |
|  |  |  |  |  |  | |
| Zeelen J,. 2010.  Authors see storytelling as as accessible, transparent and contextualized to local rural communities in South Africa which are affected by poverty and where a significant number of its inhabitants are infected by HIV.  OVERCOME INFORMATION DEFICIT MODEL. Researchers hope listeners internalise the messages rather than just hear information (information deficit model reference)  Used professional storytelling to stimulate thinking around the topic and encourage openness. Later, during interview pps told personal stories. | Stigma related to HIV/AIDs | A project called “Storytelling in the waiting room” centred on storytelling as a form of informal education in 5 health clinics in rural areas.  Aimed at decreasing the stigma around HIV/AIDS and to start an open dialogue in local communities about the disease, and ease stigma.  Professional storyteller visits clinics and uses dialogues between animal characters as proxies for real people. | “Storytelling in the waiting room” is to make use of the skills of a storyteller, who visits different clinics in rural areas in Limpopo the storyteller uses dialogues between animals as a metaphor in order to create openness around the disease.  Data collection: Semi-structured interviews were held in 5 different clinics with 10 nurses, 25 respondents from the audience (in clinic waiting room) and one storyteller.  Audience interviews were in local languages and taped. The questions were all open ended to stimulate interaction and sharing of personal stories.  The storyteller was interviewed and observed. He was local villager and told stories in traditional way of the village.  Data analysis: Observations and interviews continued until no more new insights were found. Deeper analysis followed in which the data were integrated with insights derived from the theoretical concepts concerning health education, storytelling and the creation of conversational space. | Limpopo Province in South Africa. | One of South Africa’s poorest provinces, majority live in rural areas. HIV prevalence among pregnant women attending antenatal clinics for follow up of their pregnancy was 20.7%.  Storyteller is local and knows the language, culture. | |
|  | **Participant characteristics and sample size** | | **Description of main results** | | | |
| Researchers claim creating a conversational space for storytelling was key strength of project.  Storytellers need to have the SKILLS TO EDUCATE AS WELL AS ENTERTAIN which are both entertaining and also have an important message imbedded. Storytelling is therefore “edutainment”: education enriched with entertainment.  SEE THE CROUP STUDY FOR EDUCATING AND STRIKING A BALANCE BETWEEN EDUCATION AND STORY  Stories can be shared and are non-intrusive unlike some informational messages that tell the listener what is good for them (patronising) | Storytelling audience were mainly female, under 40 years, 19 respondents were women, compared to six male interviewees. | | Observations on how to create a conversational space for storytelling, with ppts comments about experience with HIV.  CONVERSATIONAL SPACE created environment in which people feel calm and free to share their thoughts with the storyteller and learn from each other.  USED PROXY CHARACTERS EG giraffe. Animal dialogues as proxy to tell the tale. It does not make people uncomfortable.  FEELING COMFORTABLE TALKING in their communities, openness is still a problem but wanted anonymity.  COMFORTABLE STORY TELLING ENVIRONMENT OVERCOMES encourages openness and this helps overcome the gender inequality which is one reasons why HIV infection rates keep increasing.  LOCAL facilitator, local language by storyteller.  PHYSICALLY ACCESSIBLE: people attend the storytelling while they are waiting for consultation in the waiting room of a clinic.  TAILOR THE EDUCATIONAL APPROACH TO THE LOCAL CULTURAL BELIEFS.  PEOPLE CAN SHARE WITH EACH OTHER – stories can be shared and are non-intrusive unlike some informational messages that tell the listener what is good for them (patronising).  Storytellers need to have the SKILLS TO EDUCATE AS WELL AS ENTERTAIN which are both entertaining and also have an important message imbedded. Storytelling is therefore “edutainment”: education enriched with entertainment. It challenges people to think critically. | | |
|  |  |  |  |  |  | |
| **Study** | **Phenomena of interest** | **Overall design stages of study (inc. extracting info and/or intervention)** | **Methods for data collection (storytelling) and analysis** | **Location** | **Setting/context/culture** | |
| Leukefeld C, Roberto H, Hiller M, Webster M, Logan TK, Staton-Tindall M. 2003.  Illustrates how Storytelling can be used to understand more **about an issue TO INFORM POLICY** (extract info).  Stories are about cause and effect.. so structured storytelling via thought mapping helps ppts to link cause and consequence.  Storytelling ENABLED RELATING OF FACTS AND EVENTS TO THE RURAL CONTEXT  ALSO STORIES CAN BE USED TO TRY OUT DIFFERENT ENDINGS IE MORE POSITIVE ONES. | HIV and drug abuse in rural areas | Hypothesis that those in less dense populations were less likely to see themselves as at-risk from HIV  To develop new solutions, generated by the ppts towards HIV and drug abuse. | Intervention: thought mapping and structured stories among probationers in focus gps.  (The Enhanced Probation Focus Intervention)  Thought mapping added as a visual tool related to drug use and risky sex. Uses probationers own words, helps process thinking through the story - the problems and solutions all related to HIV risk. Cognitive node mapping (thought mapping) places nodes representing feelings, thoughts, actions and lines between them showing the relationships. Feedback and comments integrated these maps into structured stories. Thought maps visualise how actions link to consequences.  The intervention helps:  - develop personalised strategies and skills to protect themselves and others, and understanding their risk behaviours.  Control is NIDA standard intervention:  - information provision eg bleach equipment, safe sex.  Structured stories led to self-exploring, personal insights into behaviour that sometimes resonate with others, reinforcing the 'finding'.  Stories also allow ppts to relate their events to rural situation and context (rather than just actions, behaviours out of context).  Structured stories can be used to try out new solutions (more positive behaviours). | US, Kentucky. | In rural Kentucky, US, 61% prisoners had illegal drugs in months prior to incarceration.  Increasing number drug abusers in criminal justice system.  Rural AND urban areas experience HIV and drug abuse although policymakers assume it is urban only | |
|  | **Participant characteristics and sample size** | | **Description of main results** | | | |
|  | 200 rural probationers, 69% male, 92% white, 77% religious affiliation. Mean age 33 years. 40% court-ordered drug treatment, 87% marijuana use,50% cocaine, 45% opioids. | | Average 18.2 sexual partners. No positive HIV tests (tests included in the project) but self-reported positives for other STIs.  CHOICE OF STORYTELLING MEDUIUM IN RURAL SETTING fitting because most ppts hadn't received any info on risks previously.  STORYTELLING OR OTHER- THE RURAL GROUP WERE UNDERSERVED IN TERMS OF INFO PROVISION. No evaluation of behavioural change linked to enhanced NIDA intervention. | | |
|  |  |  |  |  |  | |
| **Study** | **Phenomena of interest** | **Overall design stages of study (inc. extracting info and/or intervention)** | **Methods for data collection (storytelling) and analysis** | **Location** | **Setting/context/culture** | |
| Dickinson D. 2011.  Stigma and misunderstanding around HIV/AIDs.  Uses the power of real lived experience to counter HIV/AIDS myths. Stories resonate with their lives I a way factual info does not.  Stories can be propagated via word of mouth in the same way that the myths circulate.( as per the anti-vaccination movement with their stories that have been shared so widely that people stop vaccinating and associated diseases increase.  Peers used not experts to encourage similarity between message source and recipient, that is vital to the ultimate impact of the message”.  All about breaking down barriers and making it enjoyable not a rigid information deficit delivery (hence 3 ppts performance of the story). |  | Overcoming stigma and misunderstandings/myths around HIV/AIDs.  Using storytelling as means of to overcome misunderstandings as opposed to repeating factual, scientific messages. | -Recorded HIV/AIDS myths  -Used these myths as content around which to develop stories  -16 stories developed.  - stories relate to parables, fables, morality tales and metaphors.  USES PEER (EDUCATORS): “similarity between message source and recipient is vital to the ultimate impact of the message”.  Myths gathered : Dictaphones used to report the HIV/AIDS myths they encountered during four recording periods (300 recordings)  Six workshops of 4-6 hrs each to discuss the HIV/AIDS myths that were recorded and to develop stories as a response to particular myths.  Stories told in informal settings while being observed by the rest of the peer educators and the researcher. One person was selected to introduce the HIV/AIDS myth, a second person was tasked with recounting the myth; and a third person would tell the story as a response to the myth. 1-3 hrs activity. Role play in vernacular (usually Zulu) to stimulate real-world use.  Following workshops, interviews with 23 of the 28 participating peer educators. |  | South African workplace. Most large South African workplaces have peer educators operating within company HIV/AIDS programmes. Mining company.  7000 employees. | |
|  | **Participant characteristics and sample size** | | **Description of main results** | | | |
| Good examples of metaphors that also reflect the way of life in South Africa. MAKE STORY RELEVANT TO AUDIENCE....RESONATES with their experiences and beliefs. Used aspects of contemporary African, working-class life e.g dogs in yards, faith-healing, second-hand cars, all reflects South African working class life.  NEED TO RESPECT LOCAL CULTURAL BELIEFS:e.g. cannot mock ancestors appearing in dreams and telling what to do (this is cultural belief), or risk alienation.  Story used here to overcome stalemate. Stories here act as agents of change.  Storytelling won’t suit all – some take to it naturally, others are uncomfortable. It is one tool in a mix. | 29 peer educators active throughout project. HIV/AIDS peer educators at SA mining company | | 80 myths identified and then distilled down to 16 for use in storytelling workshops, depending on frequency of hearing the various myths (3 major folk theories: traditional African beliefs, Christian theology, and racial understandings)  Typical stories created relating to myths e.g. The Shepard. Traditional healers have a cure for HIV and AIDS. Shepard boys decide to save time and effort by taking a shortcut across a river. Instead of crossing over the bridge, which is some way downstream, they grab the tails of cows that are swimming across the river. In the strong current they lose their grip and drown. [Don’t believe in shortcuts — if you are infected with HIV you need to use ARTs even if takes longer].  e.g. Tibbos. If there is no blood on the penis/vagina, there is no risk of HIV infection. Tibbos cycles to see his girlfriend. He falls off his bike and hits his head. He picks himself up and finds that he is not bleeding and carries on. Later in the evening he starts to get a headache, but ignores this as he is enjoying himself too much. In the morning he is rushed to the hospital, unconscious, and then dies before he can be helped. [Appearances can be dangerously deceptive — including the absence of blood.]  USE OF STORIES IN WIDER SETTING: It was evident from interactions in the workshop that some peer educators enjoyed conceiving and telling stories; others clearly found the idea of telling stories daunting and unattractive. | | | | |

**Supplementary file 3: Studies excluded by initial screening**

**Breakdown of reasons for exclusion**

Studies that were ‘off topic’ i.e not storytelling (1072);

Studies where the method was unsuitable (so there might have been elements of storytelling but not enough to warrant the methodology to be categorised as ‘storytelling’ (439)

Studies where subject or subject matter did not fit the inclusion criteria (1,073)

Studies that could not be located either unavailable or were a conference abstract with no further information (13);

Studies that were not a primary study (160);

Studies that did not satisfy the criterium that the subject matter/public health topic involved no short term sacrifice and long term population-wide gain (95).

Aaltonen J. Script as a hypothesis: Scriptwriting for documentary film. Journal of Screenwriting. 2017;8(1):55–65.

**Reason for exclusion: off topic**

Aasen M. The polarization of public concern about climate change in Norway. Climate Policy. 2017;17(2):213–30.

**Reason for exclusion: method unsuitable**

Abadie R, Goldenberg S, Welch-Lazoritz M, Fisher CB. Establishing trust in HIV/HCV research among people who inject drugs (PWID): Insights from empirical research. PLoS ONE [Electronic Resource]. 2018;13(12).

**Reason for exclusion: method unsuitable**

Abara WE, Qaseem A, Schillie S, McMahon BJ, Harris AM, High Value Care Task Force Amer C. Hepatitis B Vaccination, Screening, and Linkage to Care: Best Practice Advice From the American College of Physicians and the Centers for Disease Control and Prevention. Annals of Internal Medicine. 2017;167(11):794–+.

**Reason for exclusion: subject matter**

Abboud S, Lanier Y, Sweet Jemmott L, Sommers MS. Navigating virginities: enactment of sexual agency among Arab women in the USA. Culture, Health and Sexuality. 2019;21(10):1103–16.

**Reason for exclusion: off topic**

Abdat F, Leclercq S, Cuny X, Tissot C. Extracting recurrent scenarios from narrative texts using a Bayesian network: Application to serious occupational accidents with movement disturbance. Accident Analysis and Prevention. 2014;70:155–66.

**Reason for exclusion: subject matter**

Abebe T, Aase A. Children, AIDS and the politics of orphan care in Ethiopia: The extended family revisited. Social Science & Medicine. 2007;64(10):2058–69.

**Reason for exclusion: off topic**

Abeysinghe S. Vaccine narratives and public health: Investigating criticisms of H1N1 pandemic vaccination. PLoS Currents. 2015;7(OUTBREAKS).

**Reason for exclusion: off topic**

Abrahams S. Executive dysfunction in ALS is not the whole story. Journal of Neurology, Neurosurgery and Psychiatry. 2013;84(5):474–5.

**Reason for exclusion: subject matter**

Abramson M. Toward a more holistic understanding of ethics in social work. Social Work in Health Care. 1996;23(2):1–14.

**Reason for exclusion: method unsuitable**

Abrums M. “Jesus will fix it after awhile”: meanings and health. Social Science & Medicine. 2000;50(1):89–105.

**Reason for exclusion: subject matter**

Absar SM, Preston BL. Extending the Shared Socioeconomic Pathways for sub-national impacts, adaptation, and vulnerability studies. Global Environmental Change-Human and Policy Dimensions. 2015;33:83–96.

**Reason for exclusion: off topic**

Ackumey M, Gyapong M, Pappoe M, Weiss MG. Help-seeking for pre-ulcer and ulcer conditions of buruli ulcer disease in Ghana. Tropical Medicine and International Health. 2011;1):182.

**Reason for exclusion: subject matter**

Ackumey MM, Gyapong M, Pappoe M, Kwakye-Maclean C, Weiss MG. Illness meanings and experiences for pre-ulcer and ulcer conditions of Buruli ulcer in the Ga-West and Ga-South Municipalities of Ghana. BMC Public Health. 2012;12.

**Reason for exclusion: subject matter**

Ackumey MM, Gyapong M, Pappoe M, Maclean CK, Weiss MG. Socio-cultural determinants of timely and delayed treatment of Buruli ulcer: Implications for disease control. Infectious Diseases of Poverty. 2012;1(1):1–13.

**Reason for exclusion: subject matter**

Ackumey MM, Gyapong M, Pappoe M, Weiss MG. Help-seeking for pre-ulcer and ulcer conditions of Mycobacterium ulcerans disease (Buruli ulcer) in Ghana. American Journal of Tropical Medicine and Hygiene. 2011;85(6):1106–13.

**Reason for exclusion: subject matter**

Actis GC, Pazienza P, Rosina F. Mesalamine for inflammatory bowel disease: Recent reappraisals. Inflammation and Allergy - Drug Targets. 2008;7(1):1–5.

**Reason for exclusion: subject matter**

Adam-Poupart A, Labreche F, Smargiassi A, Duguay P, Busque MA, Gagne C, et al. Climate Change and Occupational Health and Safety in a Temperate Climate: Potential Impacts and Research Priorities in Quebec, Canada. Industrial Health. 2013;51(1):68–78.

**Reason for exclusion: off topic**

Adams AM, Gathercole SE. Phonological working memory and spoken language development in young children. Quarterly Journal of Experimental Psychology Section a-Human Experimental Psychology. 1996;49(1):216–33.

**Reason for exclusion: off topic**

Adams CF. Healthy Communities and public policy: four success stories. Public Health Reports. 2000;115(2–3):212–5.

**Reason for exclusion: off topic**

Addison SJ, Thorpe SJ. Factors involved in the formation of attitudes towards those who are mentally ill. Social Psychiatry and Psychiatric Epidemiology. 2004;39(3):228–34.

**Reason for exclusion: off topic**

Adegboyega A, Hatcher J. Sub-Saharan African immigrant men’s knowledge and support related to cervical cancer screening: A qualitative descriptive study. Cancer Epidemiology Biomarkers and Prevention Conference: 10th AACR Conference on the Science of Cancer Health Disparities in Racial/Ethnic Minorities and the Medically Underserved United States. 2018;27(7 Supplement).

**Reason for exclusion: off topic**

Adeniyi VO, Thomson E, Ter Goon D, Ajayi IA. Disclosure, stigma of HIV positive child and access to early infant diagnosis in the rural communities of OR Tambo District, South Africa: a qualitative exploration of maternal perspective. BMC Pediatrics. 2015;15.

**Reason for exclusion: method unsuitable**

Adger WN. Institutional adaptation to environmental risk under the transition in Vietnam. Annals of the Association of American Geographers. 2000;90(4):738–58.

**Reason for exclusion: method unsuitable**

Agner J, Pirkle CM, Irvin L, Maddock JE, Buchthal OV, Yamauchi J, et al. The Healthy Hawai’i Initiative: Insights from two decades of building a culture of health in a multicultural state. BMC Public Health. 2020;20(1).

**Reason for exclusion: subject matter**

Agudelo-Suarez A, Gil-Gonzalez D, Ronda-Perez E, Porthe V, Paramio-Perez G, Garcia AM, et al. Discrimination, work and health in immigrant populations in Spain. Social Science & Medicine. 2009;68(10):1866–74.

**Reason for exclusion: method unsuitable**

Ahluwalia MK, Mohabir RK. Turning to Waheguru: Religious and Cultural Coping Mechanisms of Bereaved Sikhs. Omega-Journal of Death and Dying. 2019;78(3):302–13.

**Reason for exclusion: subject**

Ahmad NY, Farrell MH. Linguistic markers of emotion in mothers of sickle cell carrier infants: What are they and what do they mean? Patient Education and Counseling. 2014;94(1):128–33.

**Reason for exclusion: subject matter**

Ahmed MEK, Omer HA, Jubran I, Al-Humayed SM, Ali HH. The decline of HCV and HBV infection prevalence among haemodialysis patients in Southern Saudi Arabia: A success story. Biomedical Research (India). 2018;29(20):3688–91.

**Reason for exclusion: off topic**

Aidi H. The grand (hip-hop) chessboard: Race, rap and raison d’Etat. Middle East Report. 2011;41(260).

**Reason for exclusion: off topic**

Ainslie PN, Reilly T. Physiology of accidental hypothermia in the mountains: a forgotten story. British Journal of Sports Medicine. 2003;37(6):548–50.

**Reason for exclusion: off topic**

Ajayi AI, Nwokocha EE, Akpan W, Adeniyi OV. Use of non-emergency contraceptive pills and concoctions as emergency contraception among Nigerian University students: results of a qualitative study. BMC Public Health. 2016;16.

**Reason for exclusion: subject**

Ajibade I, McBean G, Bezner-Kerr R. Urban flooding in Lagos, Nigeria: Patterns of vulnerability and resilience among women. Global Environmental Change-Human and Policy Dimensions. 2013;23(6):1714–25.

**Reason for exclusion: subject**

Akhavan P, Ashraph S, Barzani B, Matyas D. What justice for the yazidi genocide?: Voices from below. Human Rights Quarterly. 2020;42(1):1–47.

**Reason for exclusion: subject matter**

Akita MC. Nutrition through life course-nutrition during pregnancy (time of conception to delivery)-a case study in Teshie, a suburb of Accra. Annals of Nutrition and Metabolism. 2017;71 (Supplement 2):617.

**Reason for exclusion: off topic**

Akita MC. Pregnant women’s food in take during pregnancy-a case study in teshie, a suburb of accra. Annals of Nutrition and Metabolism. 2017;71 (Supplement 2):500.

**Reason for exclusion: off topic**

Akogun OB. THE EFFECT OF SELECTED HEALTH-EDUCATION SCHEMES ON KNOWLEDGE AND ATTITUDE OF THE KANURI TOWARDS CERTAIN PARASITIC DISEASES. Journal of the Royal Society of Health. 1992;112(6):280–5.

**Reason for exclusion: method unsuitable**

Albarus N, Whitehorne-Smith P, Abel WD. Perceived Susceptibility to Mental Disorders among Marijuana Smokers Attending a Tertiary Institution. West Indian Medical Journal. 2017;66(5):562–8.

**Reason for exclusion: method unsuitable**

Alcalde J, Heinemann N, Mabon L, Worden RH, de Coninck H, Robertson H, et al. Acorn: Developing full-chain industrial carbon capture and storage in a resource- and infrastructure-rich hydrocarbon province. Journal of Cleaner Production. 2019;233:963–71.

**Reason for exclusion: subject**

Alden DL, Friend J, Fraenkel L, Jibaja-Weiss M. The effects of culturally targeted patient decision aids on medical consultation preparation for Hispanic women in the US: Results from four randomized experiments. Social Science & Medicine. 2018;212:17–25.

**Reason for exclusion: method unsuitable**

Alexander C, Bynum N, Johnson E, King U, Mustonen T, Neofotis P, et al. Linking Indigenous and Scientific Knowledge of Climate Change. BioScience. 2011;61(6):477–84.

**Reason for exclusion: method unsuitable**

Alexander KA. “Sexual safety and security: Extending the language of sexual decision-making.” Journal of Sexual Medicine. 2013;5):302.

**Reason for exclusion: subject matter**

Alho K, Vorobyev VA, Medvedev SV, Pakhomov SV, Starchenko MG, Tervaniemi M, et al. Selective attention to human voice enhances brain activity bilaterally in the superior temporal sulcus. Brain Research. 2006;1075(1):142–50.

**Reason for exclusion: off topic**

Alho K, Vorobyev VA, Medvedev SV, Pakhomov SV, Starchenko MG, Tervaniemi M, et al. Selective attention to human voice enhances brain activity bilaterally in the superior temporal sulcus. Brain Research. 2006;1075(1):142–50.

**Reason for exclusion: off topic**

Allan K, Hoddinott P, Avenell A. A qualitative study comparing commercial and health service weight loss groups, classes and clubs. Journal of Human Nutrition and Dietetics. 2011;24(1):23–31.

**Reason for exclusion: method unsuitable**

Allen JT, Cohn SR, Ahern AL. Experiences of a commercial weight-loss programme after primary care referral: a qualitative study. British Journal of General Practice. 2015;65(633):E248–55.

**Reason for exclusion: subject**

Alves A, Lopes R, Matos P, Velho L, Silva D. Reactoon: Storytelling in a tangible environment. In: DIGITEL 2010 - The 3rd IEEE International Conference on Digital Game and Intelligent Toy Enhanced Learning. 2010. p. 161–5.

**Reason for exclusion: off topic**

Alzaid A. Empa’s New Clothes: The Untold Story of the Empa-Reg Outcome Trial. Diabetes Technology & Therapeutics. 2017;19(6):324–7.

**Reason for exclusion: subject matter**

Amalberti R, Auroy Y, Berwick D, Barach P. Five system barriers to achieving ultrasafe health care. Annals of Internal Medicine. 2005;142(9):756–64.

**Reason for exclusion: subject matter**

Amarasuriya SD, Jorm AF, Reavley NJ, Mackinnon AJ. Stigmatising attitudes of undergraduates towards their peers with depression: a cross-sectional study in Sri Lanka. BMC Psychiatry. 2015;15.

**Reason for exclusion: subject**

Amarasuriya SD, Reavley NJ, Rossetto A, Jorm AF. Helping intentions of undergraduates towards their depressed peers: a cross-sectional study in Sri Lanka. BMC Psychiatry. 2017;17.

**Reason for exclusion: subject**

Ambrosio E, Walkerley S. Broadening the ethical focus: a community perspective on patient autonomy. Humane Health Care International. 1996;12(2):E10.

**Reason for exclusion: subject matter**

Aminabadi NA, Vafaei A, Erfanparast L, Oskouei SG, Jamali Z. Impact of pictorial story on pain perception, situational anxiety and behavior in children: a cognitive-behavioral schema. Journal of Clinical Pediatric Dentistry. 2011;36(2):127–32.

**Reason for exclusion: subject matter**

Amini F, Riche NH, Lee B, Hurter C, Irani P. Understanding data videos: Looking at narrative visualization through the cinematography lens. In: Conference on Human Factors in Computing Systems - Proceedings. 2015. p. 1459–68.

**Reason for exclusion: off topic**

Ammar D, Cordova A. HIV/AIDS in Lebanese prisons: challenges and proposed actions. International journal of prison health. 2014;10(3):147–54.

**Reason for exclusion: off topic**

Ampt FH, Mudogo C, Gichangi P, Lim MSC, Manguro G, Chersich M, et al. WHISPER or SHOUT study: protocol of a cluster-randomised controlled trial assessing mHealth sexual reproductive health and nutrition interventions among female sex workers in Mombasa, Kenya. BMJ Open. 2017;7(8):e017388.

**Reason for exclusion: off topic**

Anagnostouli M, Christidi F, Zalonis I, Nikolaou C, Lyrakos D, Triantafyllou N, et al. Clinical and cognitive implications of cerebrospinal fluid oligoclonal bands in multiple sclerosis patients. Neurological Sciences. 2015;36(11):2053–60.

**Reason for exclusion: off topic**

Andersen Reeh MAR, Oevrehus AOE, Titlestad KET, Thue Lillevang STL. Prescription of blood components: Impact of a new mandatory E-Learning program for clinicians. Vox Sanguinis. 2015;1):54.

**Reason for exclusion: subject matter**

Anderson S, Fast J, Keating N, Eales J, Chivers S, Barnet D. Translating Knowledge: Promoting Health Through Intergenerational Community Arts Programming. Health Promotion Practice. 2017;18(1):15–25.

**Reason for exclusion: off topic**

Anderson-Reeves T, Goodman J, Bragg B, Leruth C. House Parties: An Innovative Model for Outreach and Community-Based Health Education. Maternal & Child Health Journal. 2017;21(Suppl 1):75–80.

**Reason for exclusion: off topic**

Andersson N, Matthis J, Paredes S, Ngxowa N. Social audit of provincial health services: building the community voice into planning in South Africa. Journal of Interprofessional Care. 2004;18(4):381–90.

**Reason for exclusion: off topic**

Andrasik MP, Burgess-Hull LA, Doucet J, Nguyen H, Gilmore AK, Otto JM, et al. The use of vignettes as an STI prevention intervention for female social drinkers. Alcoholism: Clinical and Experimental Research. 2010;34 (6):52A.

**Reason for exclusion: can't locate study**

Andrews J, Philo C. James Frame’s The Philosophy of Insanity (1860). History of Psychiatry. 2017;28(1):129–41.

**Reason for exclusion: subject**

Andrews JO, Bentley G, Crawford S, Pretlow L, Tingen MS. Using community-based participatory research to develop a culturally sensitive smoking cessation intervention with public housing neighborhoods. Ethnicity & Disease. 2007;17(2):331–7.

**Reason for exclusion: method unsuitable**

Angelo T, Kinung’hi SM, Buza J, Mwanga JR, Kariuki HC, Wilson S. Community knowledge, perceptions and water contact practices associated with transmission of urinary schistosomiasis in an endemic region: a qualitative cross-sectional study. BMC Public Health. 2019;19.

**Reason for exclusion: method unsuitable**

Anisimov VN. Metformin for cancer and aging prevention: Is it a time to make the long story short? Oncotarget. 2015;6(37):39398–407.

**Reason for exclusion: subject matter**

Annas GJ. Personalized medicine or public health? Bioethics, human rights, and choice. Revista Portuguesa de Saude Publica. 2014;32(2):158–63.

**Reason for exclusion: off topic**

Anonymous. Abstracts of the 7th Asia Pacific Medical Education Conference, APMEC 2010. Medical Education, Supplement Conference: 7th Asia Pacific Medical Education Conference, APMEC. 2010;44(SUPPL. 4).

**Reason for exclusion: subject matter**

Anonymous. Announcement: Congenital Heart Defect Awareness Week - February 7-14, 2017. MMWR - Morbidity & Mortality Weekly Report. 2017;66(4):119.

**Reason for exclusion: not primary study**

Anonymous. Asia needs political commitment to fight AIDS. AIDS Weekly Plus. 1997;23–4.

**Reason for exclusion: off topic**

Anonymous. Bedside story. Cambridge quarterly of healthcare ethics : CQ : the international journal of healthcare ethics committees. 1992;1(2):185–6.

**Reason for exclusion: off topic**

Anonymous. Breast cancer: A father’s legacy. Community Oncology. 2008;5(9):513.

**Reason for exclusion: off topic**

Anonymous. Canadian Conference on Medical Education 2011. [French, English]. Medical Education, Supplement Conference: Canadian Conference on Medical Education. 2011;45(SUPPL. 1).

**Reason for exclusion: subject matter**

Anonymous. Clopidogrel and other stories. BMJ (Online). 2013;346 (7902) (no pagination)(f2051).

**Reason for exclusion: subject matter**

Anonymous. Community-level HIV intervention in 5 cities: final outcome data from the CDC AIDS Community Demonstration Projects. American Journal of Public Health. 1999;89(3):336–45.

**Reason for exclusion: off topic**

Anonymous. Condoms too costly for Ghana’s youth. AIDS Analysis Africa. 1996;6(3):13–4.

**Reason for exclusion: not primary study**

Anonymous. Correction: ACC/AHA/AAPA/ABC/ACPM/AGS/APhA/ASH/ASPC/NMA/PCNA Guideline for the Prevention, Detection, Evaluation, and Management of High Blood Pressure in Adults: A Report of the American College of Cardiology/American Heart Association Task Force on Clinical Practice Guidelines (Journal of the American College of Cardiology (2018) 71(19) (e127-e248) (S0735109717415191) (10.1016/j.jacc.2017.11.006)). Journal of the American College of Cardiology. 2018;71(19):2275–9.

**Reason for exclusion: off topic**

Anonymous. Designing and evaluating culturally specific smoking cessation interventions for American Indian Communities. Nicotine and Tobacco Research. 2014;16(1):42–9.

**Reason for exclusion: not primary study**

Anonymous. Editorial. Pacific AIDS Alert Bulletin. 1992;(3):1.

**Reason for exclusion: not primary study**

Anonymous. Education for all draws upon population education messages. Population Education in Asia and the Pacific Newsletter. 1991;(34):17–20.

**Reason for exclusion: not primary study**

Anonymous. Francis Omaswa: From cardiothoracic surgeon to village health advocate. Interview by Charles Wendo. African health sciences. 2004;4(1):71–4.

**Reason for exclusion: off topic**

Anonymous. Maldives. Package on population education for special interest groups developed. Population Education in Asia and the Pacific Newsletter. 1995;(42):10.

**Reason for exclusion: not primary study**

Anonymous. Phenylketonuria due to phenylalanine hydroxylase deficiency: an unfolding story. Medical Research Council Working Party on Phenylketonuria. BMJ. 1993;306(6870):115–9.

**Reason for exclusion: subject matter**

Anonymous. Prerana: a success story. CEDPA Network. 1995;8.

**Reason for exclusion: not primary study**

Anonymous. Results of the low-dose (20 mg) pravastatin GISSI Prevenzione trial in 4271 patients with recent myocardial infarction: do stopped trials contribute to overall knowledge? GISSI Prevenzione Investigators (Gruppo Italiano per lo Studio della Sopravvivenza nell’Infarto Miocardico). Italian Heart Journal: Official Journal of the Italian Federation of Cardiology. 2000;1(12):810–20.

**Reason for exclusion: subject matter**

Anonymous. Shankar: reproductive health teaching aids kit for adolescent boys. Adolescence Education Newsletter. 1999;2(2):7.

**Reason for exclusion: not primary study**

Anonymous. Story in numbers. Nursing standard (Royal College of Nursing (Great Britain) : 1987). 2016;30(47):10.

**Reason for exclusion: off topic**

Anonymous. Summaries for patients: Using patient stories to improve blood pressure control. Annals of Internal Medicine. 2011;154(2):I-24.

**Reason for exclusion: not primary study**

Anonymous. Top 10 health stories of 2003. Harvard health letter / from Harvard Medical School. 2003;29(2):1–3.

**Reason for exclusion: off topic**

Anonymous. True story from general practice: the granules counting patient. [German]. MMW Fortschritte der Medizin. 2005;147(5):43, 45.

**Reason for exclusion: off topic**

Anonymous. War on cancer won’t be won in the produce aisle. A large European study finds fruits and vegetables are only modestly protective against cancer, but it’s a different story for heart disease. Harvard Health Letter. 2010;35(8):4–5.

**Reason for exclusion: subject matter**

Anonymous. [How low fat milk became high fat milk: the story of the mushroom sausage and feasts for a long life]. Nordisk Medicin. 1976;92(12):306–7.

**Reason for exclusion: off topic**

Anshel MH, Sutarso T. Effect of a storyboarding technique on selected measures of fitness among university employees. Research Quarterly for Exercise & Sport. 2010;81(3):252–63.

**Reason for exclusion: subject matter**

Antia BE, Kwasari AR. Stories of chickens and dogs: a narrative metaphor for the analysis of encounters in the veterinary clinic. Communication & Medicine. 2009;6(1):3–13.

**Reason for exclusion: subject matter**

Antin TMJ, Hunt G, Kaner E, Lipperman-Kreda S. Youth perspectives on concurrent smoking and vaping: Implications for tobacco control. International Journal of Drug Policy. 2019;66:57–63.

**Reason for exclusion: method unsuitable**

Antin TMJ, Hunt G. Embodying both stigma and satisfaction: An interview study of African American women. Critical Public Health. 2013;23(1):17–31.

**Reason for exclusion: method unsuitable**

Arean PA, Alvidrez J, Feldman M, Tong LW, Shermer R. The role of provider attitudes in prescribing antidepressants to older adults: Leverage points for effective provider education. International Journal of Psychiatry in Medicine. 2003;33(3):241–56.

**Reason for exclusion: subject**

Argyro N, Valerio L, Pierre E, De Gendt E, Julie D, Patrizia L, et al. A familial adrenal incidentaloma story. Acta Clinica Belgica: International Journal of Clinical and Laboratory Medicine. 2016;71 (Supplement 3):19–20.

**Reason for exclusion: subject matter**

Aricò E, Castiello L, Capone I, Gabriele L, Belardelli F. Type i interferons and cancer: An evolving story demanding novel clinical applications. Cancers. 2019;11(12).

**Reason for exclusion: off topic**

Arifin A, Tumar A. Beyond fiction: Using character profiling in screenwriting as a tool to explore higher education teachers’ identity. Turkish Online Journal of Educational Technology. 2017;2017(October Special Issue INTE):439–48.

**Reason for exclusion: off topic**

Armstrong K, Quistberg DA, Micco E, Domchek S, Guerra C. Prescription of tamoxifen for breast cancer prevention by primary care physicians. Archives of Internal Medicine. 2006;166(20):2260–5.

**Reason for exclusion: subject**

Arnall A, Kothari U. Challenging climate change and migration discourse: Different understandings of timescale and temporality in the Maldives. Global Environmental Change-Human and Policy Dimensions. 2015;31:199–206.

**Reason for exclusion: off topic**

Arnold LDW. The Ethics of Donor Human Milk Banking. Breastfeeding Medicine. 2006;1(1):3–13.

**Reason for exclusion: method unsuitable**

Aronowitz R. “Screening” for prostate cancer in New York’s skid row: history and implications. American Journal of Public Health. 2014;104(1):70–6.

**Reason for exclusion: off topic**

Arraiz Matute A, Da Silva L, Pendleton Jiménez K, Smith A. The sex of it all: outness and queer women’s digital storytelling in teacher education. Teaching Education. 2020;

**Reason for exclusion: subject matter**

Arring N, Costello D. Fall reduction: Strategies in action. Biology of Blood and Marrow Transplantation. 2013;1):S355.

**Reason for exclusion: not primary study**

Ashton CM, Houston TK, Williams JH, Larkin D, Trobaugh J, Crenshaw K, et al. A stories-based interactive DVD intended to help people with hypertension achieve blood pressure control through improved communication with their doctors. Patient Education & Counseling. 2010;79(2):245–50.

**Reason for exclusion: subject matter**

Ashton J. The power of narrative. Journal of the Royal Society of Medicine. 2020;113(1):41–2.

**Reason for exclusion: not primary study**

Aubel J, Toure I, Diagne M. Senegalese grandmothers promote improved maternal and child nutrition practices: The guardians of tradition are not averse to change. Social Science and Medicine. 2004;59(5):945–59.

**Reason for exclusion: method unsuitable**

Audrey S, Batista-Ferrer H. Healthy urban environments for children and young people: A systematic review of intervention studies. Health & Place. 2015;36:97–117.

**Reason for exclusion: method unsuitable**

Auer C, Sarol J, Tanner M, Weiss M. Health seeking and perceived causes of tuberculosis among patients in Manila, Philippines. Tropical Medicine & International Health. 2000;5(9):648–56.

**Reason for exclusion: method unsuitable**

Aujoulat I, Marcolongo R, Bonadiman L, Deccache A. Reconsidering patient empowerment in chronic illness: A critique of models of self-efficacy and bodily control. Social Science and Medicine. 2008;66(5):1228–39.

**Reason for exclusion: not primary study**

Avanza M. Using a Feminist Paradigm (Intersectionality) to Study Conservative Women: The Case of Pro-life Activists in Italy. Politics and Gender. 2019;

**Reason for exclusion: off topic**

Aviles NB. Situated Practice and the Emergence of Ethical Research: HPV Vaccine Development and Organizational Cultures of Translation at the National Cancer Institute. Science Technology & Human Values. 2018;43(5):810–33.

**Reason for exclusion: method unsuitable**

Azevedo KJ, Mendoza S, Fernandez M, Haydel KF, Fujimoto M, Tirumalai EC, et al. TURN OFF THE TV AND DANCE! PARTICIPATION IN CULTURALLY TAILORED HEALTH INTERVENTIONS: IMPLICATIONS FOR OBESITY PREVENTION AMONG MEXICAN AMERICAN GIRLS. Ethnicity & Disease. 2013;23(4):452–61.

**Reason for exclusion: method unsuitable**

Baart I, Widdershoven G. Bipolar disorder: Idioms of susceptibility and disease and the role of “genes” in illness explanations. Health. 2013;17(6):640–57.

**Reason for exclusion: method unsuitable**

Bachhuber MA, McGinty EE, Kennedy-Hendricks A, Niederdeppe J, Barry CL. Messaging to Increase Public Support for Naloxone Distribution Policies in the United States: Results from a Randomized Survey Experiment. PLoS ONE [Electronic Resource]. 2015;10(7).

**Reason for exclusion: subject**

Bachman AS, Cohen EL, Collins T, Hatcher J, Crosby R, Vanderpool RC. Identifying Communication Barriers to Colorectal Cancer Screening Adherence among Appalachian Kentuckians. Health Communication. 2018;33(10):1284–92.

**Reason for exclusion: method unsuitable**

Backinger C. Personal service workers: a critical link in the AIDS education chain? AIDS Education & Prevention. 1989;1(1):31–8.

**Reason for exclusion: off topic**

Baeyens JP. Ensuring the willingness to vaccinate and be vaccinated. Expert Review of Vaccines. 2010;9(3 Suppl):11–4.

**Reason for exclusion: not primary study**

Bagley H. Editorial * In their own words: A parent’s story. Journal of Evidence-Based Medicine. 2015;8(3):172.

**Reason for exclusion: off topic**

Baglow L, Davis L, Shapter C. Improving cognitive testing within stroke rehabilitation: What can patient narratives tell us about the experience? International Journal of Stroke. 2013;1):30.

**Reason for exclusion: s**

Bai Z, Liu B, Lee K. Piggy banksy: A transformational playground for social change through playful co-creation. In: CHI PLAY 2019 - Extended Abstracts of the Annual Symposium on Computer-Human Interaction in Play. 2019. p. 349–56.

**Reason for exclusion: subject matter**

Baikie M, Shiwak I, Wolfrey C, Cunsolo Willox A, Harper S, Edge V. Storytelling in a digital age: Digital storytelling as an emergent narrative method for climate-health research and promotion. In: Conference Proceedings, International Polar Year Conference. 2012.

**Reason for exclusion: not primary study**

Baird B. Medical abortion in Australia: A short history. Reproductive Health Matters. 2015;23(46):169–76.

**Reason for exclusion: not primary study**

Baker JP. Immunization and the American way: 4 childhood vaccines. American Journal of Public Health. 2000;90(2):199–207.

**Reason for exclusion: not primary study**

Baker LM. Information needs at the end of life: a content analysis of one person’s story. Journal of the Medical Library Association. 2004;92(1):78–82.

**Reason for exclusion: subject matter**

Bakshi N, Sinha C, Ross D, Khemani K, Loewenstein G, Krishnamurti L. Shared decision making or physician advocate for a particular treatment option: A spectrum of approaches to decision making about disease modifying therapies in sickle cell disease. Blood Conference: 58th Annual Meeting of the American Society of Hematology, ASH. 2016;128(22).

**Reason for exclusion: off topic**

Bakshi N, Sinha CB, Ross D, Khemani K, Loewenstein G, Krishnamurti L. Proponent or collaborative: Physician perspectives and approaches to disease modifying therapies in sickle cell disease. PLoS ONE [Electronic Resource]. 2017;12(7).

**Reason for exclusion: subject**

Baldwin AE, Usher K. Going the distance--experiences of women with gynaecological cancer residing in rural remote north Queensland. International Journal of Nursing Practice. 2008;14(4):322–8.

**Reason for exclusion: subject matter**

Ball J, Hoek J, Tautolo ES, Gifford H. New Zealand policy experts’ appraisal of interventions to reduce smoking in young adults: a qualitative investigation. BMJ Open. 2017;7(12):e017837.

**Reason for exclusion: subject matter**

Baller FAE, Ludwig KV, Olivares C, Graef-Calliess IT. Exploring the ideas and expectations of German medical students towards career choices and the speciality of psychiatry. International Review of Psychiatry. 2013;25(4):425–30.

**Reason for exclusion: subject**

Balmer B. Biological warfare: the threat in historical perspective. Medicine, Conflict & Survival. 2002;18(2):120–37.

**Reason for exclusion: subject matter**

Banas JA, Rains SA. A Meta-Analysis of Research on Inoculation Theory. Communication Monographs. 2010;77(3):281–311.

**Reason for exclusion: subject**

Banks J %J PM. Storytelling to access social context and advance health equity research. 2012;55(5):394–7.

**Reason for exclusion: subject matter**

Baranowski T, Buday R, Thompson DI, Baranowski J. Playing for real: video games and stories for health-related behavior change. American Journal of Preventive Medicine. 2008;34(1):74–82.

**Reason for exclusion: off topic**

Barbaro E, Oliveira AP, Soares J, Codato G, Ferreira MJ, Mlakar P, et al. Observational characterization of the downward atmospheric longwave radiation at the surface in the city of São Paulo. Journal of Applied Meteorology and Climatology. 2010;49(12):2574–90.

**Reason for exclusion: off topic**

Barclay K. Marriage, sex, and the Church of Scotland: Exploring non-conformity amongst the lower orders. Journal of Religious History. 2019;43(2):163–79.

**Reason for exclusion: off topic**

Bardosh KL, Scoones JC, Grace D, Kalema-Zikusoka G, Jones KE, de Balogh K, et al. Engaging research with policy and action: what are the challenges of responding to zoonotic disease in Africa? Philosophical Transactions of the Royal Society B-Biological Sciences. 2017;372(1725).

**Reason for exclusion: method unsuitable**

Bardosh KL. Towards a science of global health delivery: A socio-anthropological framework to improve the effectiveness of neglected tropical disease interventions. PLoS Neglected Tropical Diseases. 2018;12 (7) (no pagination)(e0006537).

**Reason for exclusion: not primary study**

Bardsley DK, Palazzo E, Stringer R. What should we conserve? Farmer narratives on biodiversity values in the McLaren Vale, South Australia. Land Use Policy. 2019;83:594–605.

**Reason for exclusion: off topic**

Bardsley DK, Weber D, Robinson GM, Moskwa E, Bardsley AM. Wildfire risk, biodiversity and pen-urban planning in the Mt Lofty Ranges, South Australia. Applied Geography. 2015;63:155–65.

**Reason for exclusion: subject**

Bardsley DK, Wiseman ND. Socio-ecological lessons for the Anthropocene: Learning from the remote Indigenous communities of Central Australia. Anthropocene. 2016;14:58–70.

**Reason for exclusion: method unsuitable**

Barney LJ, Griffiths KM, Banfield MA. Explicit and implicit information needs of people with depression: a qualitative investigation of problems reported on an online depression support forum. BMC Psychiatry. 2011;11.

**Reason for exclusion: method unsuitable**

Barney LJ, Griffiths KM, Christensen H, Jorm AF. Exploring the nature of stigmatising beliefs about depression and help-seeking: Implications for reducing stigma. BMC Public Health. 2009;9.

**Reason for exclusion: method unsuitable**

Barr S, Pollard J. Geographies of Transition: Narrating environmental activism in an age of climate change and “Peak Oil.” Environment and Planning A. 2017;49(1):47–64.

**Reason for exclusion: method unsuitable**

Barratt CLR, Bjorndahl L, De Jonge CJ, Lamb DJ, Martini FO, McLachlan R, et al. The diagnosis of male infertility: an analysis of the evidence to support the development of global WHO guidance-challenges and future research opportunities. Human Reproduction Update. 2017;23(6):660–80.

**Reason for exclusion: subject**

Barrett RJ, Parker DB. Symbolism of community I: the boundary between hospital and community. Australian and New Zealand Journal of Psychiatry. 2006;40(4):310–7.

**Reason for exclusion: subject**

Barrington C, Kerrigan D, Urena FIC, Brudney K. La vida normal: living with HIV in Santo Domingo, Dominican Republic. Culture Health & Sexuality. 2018;20(1):40–54.

**Reason for exclusion: method unsuitable**

Barsdorf NW, Wassenaar DR. Racial differences in public perceptions of voluntariness of medical research participants in South Africa. Social Science & Medicine. 2005;60(5):1087–98.

**Reason for exclusion: subject**

Barsties B. Effects of different tasks on determination of the speaking fundamental frequency. HNO. 2013;61(7):609–16.

**Reason for exclusion: off topic**

Barth JH, O’Kane M. Obesity services: how best to develop a coherent way forward. Clinical Endocrinology. 2016;84(3):321–4.

**Reason for exclusion: subject**

Bartsch K, London K, Campbell MD. Children’s attention to beliefs in interactive persuasion tasks. Developmental Psychology. 2007;43(1):111–20.

**Reason for exclusion: off topic**

Basaran NF, Akici A. Patients’ experience and perspectives on the rational use of drugs in Turkey: A survey study. Patient Preference and Adherence. 2012;6:719–24.

**Reason for exclusion: subject matter**

Bashir A. Maternal mortality in Pakistan. A success story of the Faisalabad district. IPPF Medical Bulletin. 1991;25(2):1–3.

**Reason for exclusion: off topic**

Bass J, Mullen MA. A Narrative Model of Informed Consent with Youth: Decisions around Elective Non-Medical Circumcision. Medicine and law. 2014;33(4):147–55.

**Reason for exclusion: not primary study**

Bass SB, Greener JR, Ruggieri D, Parvanta C, Mora G, Wolak C, et al. Attitudes and Perceptions of Urban African Americans of a “Dirty Bomb” Radiological Terror Event: Results of a Qualitative Study and Implications for Effective Risk Communication. Disaster Medicine and Public Health Preparedness. 2015;9(1):9–18.

**Reason for exclusion: subject**

Basu AR. Discourse on homosexual identity: Focus group exploration of a group of men who have sex with men in Calcutta. Man in India. 1999;79(1–2):147–59.

**Reason for exclusion: method unsuitable**

Basu RN. Smallpox eradication: lessons learnt from a success story. National Medical Journal of India. 2006;19(1):33–6.

**Reason for exclusion: off topic**

Bateman C. The PPP “without the GP” - A Western Cape story. South African Medical Journal. 2013;103(3):137–9.

**Reason for exclusion: off topic**

Bauman A, Bittman M, Gershuny J. A short history of time use research; implications for public health. BMC Public Health. 2019;19.

**Reason for exclusion: subject**

Baume C, Helitzer D, Kachur SP. Patterns of care for childhood malaria in Zambia. Social Science & Medicine. 2000;51(10):1491–503.

**Reason for exclusion: method unsuitable**

Baxter SL, Collins SC, Hill AJ. “Thin people ... they”re healthy’: young children’s understanding of body weight change. Pediatric Obesity. 2016;11(5):418–24.

**Reason for exclusion: subject matter**

Bay-Jensen AC, Leeming DJ, Kleyer A, Veidal SS, Schett G, Karsdal MA. Ankylosing spondylitis is characterized by an increased turnover of several different metalloproteinase-derived collagen species: a cross-sectional study. Rheumatology International. 2012;32(11):3565–72.

**Reason for exclusion: method unsuitable**

Bay-Larsen I, Risvoll C, Vestrum I, Bjorkhaug H. Local protein sources in animal feed - Perceptions among arctic sheep farmers. Journal of Rural Studies. 2018;59:98–110.

**Reason for exclusion: subject**

Bayles BP, Katerndahl DA. CULTURE-BOUND SYNDROMES IN HISPANIC PRIMARY CARE PATIENTS. International Journal of Psychiatry in Medicine. 2009;39(1):15–31.

**Reason for exclusion: subject**

Bedford H. The reporting of medical findings needs to be overhauled to prevent the media’s obsession with health scare stories. Nursing times. 1998;94(10):41.

**Reason for exclusion: subject matter**

Beilin R, Sysak T, Hill S. Farmers and perverse outcomes: The quest for food and energy security, emissions reduction and climate adaptation. Global Environmental Change-Human and Policy Dimensions. 2012;22(2):463–71.

**Reason for exclusion: method unsuitable**

Beitsch LM, Carretta H, McKeever J, Pattnaik A, Gillen S. The quantitative story behind the quality improvement storyboards: a synthesis of quality improvement projects conducted by the multi-state learning collaborative. Journal of Public Health Management & Practice. 2013;19(4):330–40.

**Reason for exclusion: off topic**

Bekalu MA, Bigman CA, McCloud RF, Lin LK, Viswanath K. The relative persuasiveness of narrative versus non-narrative health messages in public health emergency communication: Evidence from a field experiment. Preventive Medicine. 2018;111:284–90.

**Reason for exclusion: subject matter**

Bekker HL, Winterbottom AE, Butow P, Dillard AJ, Feldman-Stewart D, Fowler FJ, et al. Do personal stories make patient decision aids more effective? A critical review of theory and evidence. BMC Medical Informatics & Decision Making. 2013;13 Suppl 2:S9.

**Reason for exclusion: subject matter**

Bekman S, Aksu-Koc A, Erguvanli-Taylan E. Effectiveness of an Intervention Program for Six Year Olds: A Summer-School Model. Turk Psikoloji Dergisi. 2012;27(70):48–64.

**Reason for exclusion: subject**

Beletsky L, Wagner KD, Arredondo J, Palinkas L, Rodriguez CM, Kalic N, et al. Implementing Mexico’s “Narcomenudeo” Drug Law Reform: A Mixed Methods Assessment of Early Experiences Among People Who Inject Drugs. Journal of Mixed Methods Research. 2016;10(4):384–401.

**Reason for exclusion: can't find study**

Bell SL, Phoenix C, Lovell R, Wheeler BW. Seeking everyday wellbeing: The coast as a therapeutic landscape. Social Science and Medicine. 2015;142:56–67.

**Reason for exclusion: off topic**

Bell SL, Phoenix C, Lovell R, Wheeler BW. Using GPS and geo-narratives: a methodological approach for understanding and situating everyday green space encounters. Area. 2015;47(1):88–96.

**Reason for exclusion: off topic**

Belon AP, Nieuwendyk LM, Vallianatos H, Nykiforuk CIJ. Community Lenses Revealing the Role of Sociocultural Environment on Physical Activity. American Journal of Health Promotion. 2016;30(3):E92–100.

**Reason for exclusion: subject matter**

Belon AP, Nieuwendyk LM, Vallianatos H, Nykiforuk CIJ. Perceived community environmental influences on eating behaviors: A Photovoice analysis. Social Science & Medicine. 2016;171:18–29.

**Reason for exclusion: subject matter**

Below TB, Schmid JC, Sieber S. Farmers’ knowledge and perception of climatic risks and options for climate change adaptation: a case study from two Tanzanian villages. Regional Environmental Change. 2015;15(7):1169–80.

**Reason for exclusion: method unsuitable**

Ben-Sasson A. Parents’ search for evidence-based practice: a personal story. Journal of Paediatrics & Child Health. 2011;47(7):415–8.

**Reason for exclusion: subject matter**

Benemariya E, Chironda G, Nkurunziza A, Katende G, Sego R, Mukeshimana M. Perceived factors for delayed consultation of cervical cancer among women at a selected hospital in Rwanda: An exploratory qualitative study. International Journal of Africa Nursing Sciences. 2018;9:129–35.

**Reason for exclusion: off topic**

Benham CF. Understanding local community attitudes toward industrial development in the Great Barrier Reef region World Heritage Area: are environmental impacts perceived to overshadow economic benefits? Natural Resources Forum. 2017;41(1):42–54.

**Reason for exclusion: method unsuitable**

Benjamin D, Por HH, Budescu D. Climate Change Versus Global Warming: Who Is Susceptible to the Framing of Climate Change? Environment and Behavior. 2017;49(7):745–70.

**Reason for exclusion: method unsuitable**

Benkirane R, Rhalem N, Abouali F, Soulaymani R. The value of toxicity information collected by poisons centres for patient safety. Clinical Toxicology. 2009;47 (5):493–4.

**Reason for exclusion: subject matter**

Benn S, Brown P, North-Samardzic A. A commentary on decision-making and organisational legitimacy in the Risk Society. Journal of Environmental Management. 2009;90(4):1655–62.

**Reason for exclusion: subject matter**

Bennett NJ. In Political Seas: Engaging with Political Ecology in the Ocean and Coastal Environment. Coastal Management. 2019;47(1):67–87.

**Reason for exclusion: method unsuitable**

Bennett ST, Liu JH. Historical trajectories for reclaiming an indigenous identity in mental health interventions for Aotearoa/New Zealand-Maori values, biculturalism, and multiculturalism. International Journal of Intercultural Relations. 2018;62:93–102.

**Reason for exclusion: method unsuitable**

Bensadon BA, Odenheimer GL. Listening to our elders: A story of resilience and recovery. Patient Education and Counseling. 2014;95(3):433–4.

**Reason for exclusion: not primary study**

Benson CA, Cappiello S, Campbell B. Subsea multilateral technology step change in Australia. In: Proceedings - SPE Annual Technical Conference and Exhibition. 2017.

**Reason for exclusion: off topic**

Bentley C, Browman GP, Poole B. Conceptual and practical challenges for implementing the communities of practice model on a national scale--a Canadian cancer control initiative. BMC Health Services Research. 2010;10:3.

**Reason for exclusion: subject matter**

Bentley ME, Corneli AL, Piwoz E, Moses A, Nkhoma J, Tohill BC, et al. Perceptions of the role of maternal nutrition in HIV-positive breast-feeding women in Malawi. Journal of Nutrition. 2005;135(4):945–9.

**Reason for exclusion: off topic**

Benton A. Exceptional Suffering? Enumeration and Vernacular Accounting in the HIV-Positive Experience. Medical Anthropology. 2012;31(4):310–28.

**Reason for exclusion: method unsuitable**

Beres LK, Winskell K, Neri EM, Mbakwem B, Obyerodhyambo O. Making sense of HIV testing: Social representations in young Africans’ HIV-related narratives from six countries. Global Public Health. 2013;8(8):890–903.

**Reason for exclusion: off topic**

Berghs M, Dyson SM, Atkin K. Resignifying the sickle cell gene: Narratives of genetic risk, impairment and repair. Health (London, England : 1997). 2017;21(2):171–88.

**Reason for exclusion: subject matter**

Bergquist M, Nilsson A, Schultz PW. Experiencing a Severe Weather Event Increases Concern About Climate Change. Frontiers in Psychology. 2019;10.

**Reason for exclusion: method unsuitable**

Berkley-Patton J, Goggin K, Liston R, Bradley-Ewing A, Neville S. Adapting effective narrative-based HIV-prevention interventions to increase minorities’ engagement in HIV/AIDS services. Health Communication. 2009;24(3):199–209.

**Reason for exclusion: off topic**

Berman EP, Milanes-Reyes LM. The Politicization of Knowledge Claims: The “Laffer Curve” in the US Congress. Qualitative Sociology. 2013;36(1):53–79.

**Reason for exclusion: off topic**

Bernardon S, Pernice-Duca F. Integrating Recovery and the Narrative Attachment Systems Perspective to Working through Borderline Personality Disorder. Family Journal: Counseling and Therapy for Couples and Families. 2012;20(3):239–48.

**Reason for exclusion: subject matter**

Bernasconi O. Being decent, being authentic: The moral self in shifting discourses of sexuality across three generations of chilean women. Sociology. 2010;44(5):860–75.

**Reason for exclusion: subject matter**

Berry M, Batty C. The stories of supervision: Creative writing in a critical space. New Writing. 2016;13(2):247–60.

**Reason for exclusion: n**

Betsch C, Renkewitz F, Haase N. Effect of narrative reports about vaccine adverse events and bias-awareness disclaimers on vaccine decisions: a simulation of an online patient social network. Medical decision making : an international journal of the Society for Medical Decision Making. 2013;33(1):14–25.

**Reason for exclusion: off topic**

Bettigole C, Farley TA. The Philadelphia Story: Attacking Behavioral and Social Determinants of Health. Annals of Internal Medicine. 2016;165(8):593–4.

**Reason for exclusion: off topic**

Bettivia R. Boxes. Qualitative Inquiry. 2014;20(3):356–8.

**Reason for exclusion: off topic**

Beymer-Farris BA, Bassett TJ. The REDD menace: Resurgent protectionism in Tanzania’s mangrove forests. Global Environmental Change-Human and Policy Dimensions. 2012;22(2):332–41.

**Reason for exclusion: off topic**

Bezold C. The future of patient-centered care: scenarios, visions, and audacious goals. Journal of Alternative & Complementary Medicine. 2005;11 Suppl 1:S77-84.

**Reason for exclusion: off topic**

Bhatia DS, Harrison AD, Kubeka M, Milford C, Kaida A, Bajunirwe F, et al. The Role of Relationship Dynamics and Gender Inequalities As Barriers to HIV-Serostatus Disclosure: Qualitative Study among Women and Men Living with HIV in Durban, South Africa. Frontiers in Public Health. 2017;5.

**Reason for exclusion: method unsuitable**

Bhatia N. Censorship, “Obscenity” and Courtroom Drama: Reading Ismat Chughtai’s “Lihaaf” and “The ‘Lihaaf’ Trial.” Law and Literature. 2020;

**Reason for exclusion: off topic**

Bhattacharya G. Spirituality and Type 2 Diabetes Self-Management Among African Americans in the Arkansas Delta. Journal of Social Service Research. 2013;39(4):469–82.

**Reason for exclusion: no short term sacrifice, long term gain**

Bhende AA. Country watch: India. Aids/Std Health Promotion Exchange. 1996;(3):6–7.

**Reason for exclusion: subject matter**

Biehl J. Pharmaceuticalization: AIDS treatment and global health politics. Anthropological Quarterly. 2007;80(4):1083–126.

**Reason for exclusion: method unsuitable**

Bierer BE. The Revised and Final Common Rule: An Unfinished Story. Irb: a Review of Human Subjects Research. 2017;39(6):6–10.

**Reason for exclusion: subject matter**

Bierman KL, Welsh JA, Heinrichs BS, Nix RL, Mathis ET. Helping Head Start Parents Promote Their Children’s Kindergarten Adjustment: The Research-Based Developmentally Informed Parent Program. Child Development. 2015;86(6):1877–91.

**Reason for exclusion: subject matter**

Biffi M. The never-ending story of CIED infection prevention: shall we WRAP-IT and go ? Journal of cardiovascular electrophysiology. 2019;6.

**Reason for exclusion: off topic**

Biggar M, Ardoin NM. More than good intentions: the role of conditions in personal transportation behaviour. Local Environment. 2017;22(2):141–55.

**Reason for exclusion: subject**

Biglino G, Layton S, Lee M, Sophocleous F, Hall S, Wray J. ’Making the Invisible Visible ’: An audience response to an art installation representing the complexity of congenital heart disease and heart transplantation. Medical Humanities. 2019;45(4):399–405.

**Reason for exclusion: off topic**

Biglino G, Layton S, Lee M, Sophocleous F, Hall S, Wray J. “Making the Invisible Visible”: an audience response to an art installation representing the complexity of congenital heart disease and heart transplantation. Medical humanities. 2018;18.

**Reason for exclusion: off topic**

Bikker AP, Macdonald S, Robb KA, Conway E, Browne S, Campbell C, et al. Perceived colorectal cancer candidacy and the role of candidacy in colorectal cancer screening. Health, Risk and Society. 2019;21(7–8):352–72.

**Reason for exclusion: no short term sacrifice, long term gain**

Bilandzic H, Sukalla F. The Role of Fictional Film Exposure and Narrative Engagement for Personal Norms, Guilt and Intentions to Protect The Climate. Environmental Communication-a Journal of Nature and Culture.

**Reason for exclusion: method unsuitable**

Bill-Harvey D, Rippey R, Abeles M, Donald MJ, Downing D, Ingenito F, et al. Outcome of an osteoarthritis education program for low-literacy patients taught by indigenous instructors. Patient Education & Counseling. 1989;13(2):133–42.

**Reason for exclusion: subject matter**

Bilodeau G, Witteman H, Legare F, Lafontaine-Bruneau J, Voyer P, Kroger E, et al. Reducing complexity of patient decision AIDS for community-based older adults with dementia and their caregivers: Multiple case study of Decision Boxes. BMJ Open. 2019;9 (5) (no pagination)(e027727).

**Reason for exclusion: off topic**

Bird SM, Wiles JL, Okalik L, Kilabuk J, Egeland GM. Living with diabetes on Baffin Island: Inuit storytellers share their experiences. Canadian Journal of Public Health-Revue Canadienne De Sante Publique. 2008;99(1):17–21.

**Reason for exclusion: no short term sacrifice, long term gain**

Birungi H, Obare F, Mugisha JF, Evelia H, Nyombi J. Preventive service needs of young people perinatally infected with HIV in Uganda. AIDS Care. 2009;21(6):725–31.

**Reason for exclusion: off topic**

Bissell P, Peacock M, Holdsworth M, Powell K, Wilcox J, Clonan A. Introducing the idea of "assumed shared food narratives’ in the context of social networks: reflections from a qualitative study conducted in Nottingham, England. Sociology of Health & Illness. 2018;40(7):1142–55.

**Reason for exclusion: method unsuitable**

Bissonnette JF, Dupras J, Messier C, Lechowicz M, Dagenais D, Paquette A, et al. Moving forward in implementing green infrastructures: Stakeholder perceptions of opportunities and obstacles in a major North American metropolitan area. Cities. 2018;81:61–70.

**Reason for exclusion: method unsuitable**

Bitencourt FV, Correa HW, Toassi RFC. Tooth loss experiences in adult and elderly users of Primary Health Care. Ciencia & Saude Coletiva. 2019;24(1):169–80.

**Reason for exclusion: subject**

Black AL, Crimmins G, Henderson L. Positioning ourselves in our academic lives: exploring personal/professional identities, voice and agency. Discourse. 2019;40(4):530–44.

**Reason for exclusion: off topic**

Blackburn H. 20th-Century “medical Marco Polos” in the origins of preventive cardiology and cardiovascular disease epidemiology. American Journal of Cardiology. 2012;109(5):756–67.

**Reason for exclusion: subject matter**

Blackhall F. Novel Trial Design for Precision Medicine. Journal of Thoracic Oncology. 2018;13 (10 Supplement):S209–10.

**Reason for exclusion: off topic**

Blackman T, Harrington B, Elliott E, Greene A, Hunter DJ, Marks L, et al. Framing health inequalities for local intervention: Comparative case studies. Sociology of Health and Illness. 2012;34(1):49–63.

**Reason for exclusion: subject matter**

Blaisdell LR. One practice’s intraoral camera success story. Dental economics - oral hygiene. 1995;85(11):48–50, 52–4.

**Reason for exclusion: subject matter**

Blanco E, Imbrizi JM. Voice perception in teachers: life-story narratives between workspaces and the scenic choir. Codas. 2019;31(2):e20180200.

**Reason for exclusion: off topic**

Blanco ESDV, Imbrizi JM. Voice perception in teachers: life-story narratives between workspaces and the scenic choir. Codas. 2019;31(2):e20180200.

**Reason for exclusion: subject matter**

Blaxekjaer LO, Nielsen TD. Mapping the narrative positions of new political groups under the UNFCCC. Climate Policy. 2015;15(6):751–66.

**Reason for exclusion: method unsuitable**

Blaxter M. Caenorhabditis elegans is a nematode. Science. 1998;282(5396):2041–6.

**Reason for exclusion: subject matter**

Blennow K, Persson J, Tome M, Hanewinkel M. Climate Change: Believing and Seeing Implies Adapting. PLoS ONE [Electronic Resource]. 2012;7(11).

**Reason for exclusion: method unsuitable**

Bloodhart B, Maibach E, Myers T, Zhao XQ. Local Climate Experts: The Influence of Local TV Weather Information on Climate Change Perceptions. PLoS ONE [Electronic Resource]. 2015;10(11).

**Reason for exclusion: method unsuitable**

Bloom FR. “New beginnings”: A case study in gay men’s changing perceptions of quality of life during the course of HIV infection. Medical Anthropology Quarterly. 2001;15(1):38–57.

**Reason for exclusion: off topic**

Blum AL. Solitary views of the stomach. Digestion. 1996;57(5):287–98.

**Reason for exclusion: subject**

Blundell TL. Protein crystallography and drug discovery: recollections of knowledge exchange between academia and industry. Iucrj. 2017;4:308–21.

**Reason for exclusion: subject**

Blunt W, Gill DP, Sibbald SL, Riggin B, Pulford RW, Scott R, et al. Optimization of the Hockey Fans in Training (Hockey FIT) weight loss and healthy lifestyle program for male hockey fans. BMC Public Health. 2017;17.

**Reason for exclusion: method unsuitable**

Bockstael E, Bahia NCF, Seixas CS, Berkes F. Participation in protected area management planning in coastal Brazil. Environmental Science & Policy. 2016;60:1–10.

**Reason for exclusion: off topic**

Boeijinga A, Hoeken H, Sanders J. Storybridging: Four steps for constructing effective health narratives. Health Education Journal. 2017;76(8):923–35.

**Reason for exclusion: not primary study**

Boeri MW. 2002 Peter K. New Prize recipient - “Hell, I’m an addict, but I ain’t no junkie” - An ethnographic analysis of aging heroin users. Human Organization. 2004;63(2):236–45.

**Reason for exclusion: method unsuitable**

Boiarsky G, Rouner D, Long M. Effects of responsibility attribution and message source on young adults’ health attitudes and behaviors. Journal of Health Communication. 2013;18(7):881–94.

**Reason for exclusion: off topic**

Boivin MJ, Chernoff M, Laughton B, Bwakura-Dangarembizi M, Kamthunzi P, Barlow-Mosha L, et al. Neuropsychological outcomes in a two year african-based pediatric observational study. Topics in Antiviral Medicine. 2017;25 (1 Supplement 1):351s.

**Reason for exclusion: subject matter**

Bojczyk KE, Lehan TJ, McWey LM, Melson GF, Kaufman DR. Mothers’ and Their Adult Daughters’ Perceptions of Their Relationship. Journal of Family Issues. 2011;32(4):452–81.

**Reason for exclusion: subject**

Bokhour BG, Fix GM, Gordon HS, Long JA, DeLaughter K, Orner MB, et al. Can stories influence African-American patients’ intentions to change hypertension management behaviors? A randomized control trial. Patient Education and Counseling. 2016;99(9):1482–8.

**Reason for exclusion: no short term sacrifice, long term gain**

Bollinger S, Kreuter MW. Real-Time Moment-to-Moment Emotional Responses to Narrative and Informational Breast Cancer Videos in African American Women. Health Education Research. 2012;27(3):537–43.

**Reason for exclusion: off topic**

Bollo Gasol S, Pinol-Ripoll G, Llorente Vizcaino A, Peraita Adrados H. Everyday memory tasks distinguish mild cognitive impairment due to Alzheimer’s disease from non-converter MCI patients. European Journal of Neurology. 2012;19 (SUPPL.1):530.

**Reason for exclusion: subject matter**

Bollo-Gasol S, Pinol-Ripoll G, Cejudo-Bolivar JC, Llorente-Vizcaino A, Peraita-Adrados H. Ecological assessment of mild cognitive impairment and Alzheimer disease using the Rivermead Behavioural Memory Test. [Spanish]. Neurologia. 2014;29(6):339–45.

**Reason for exclusion: subject matter**

Boman A, Povlsen L, Dahlborg-Lyckhage E, Hanas R, Borup I. Fathers’ encounter of support from paediatric diabetes teams; the tension between general recommendations and personal experience. Health & Social Care in the Community. 2013;21(3):263–70.

**Reason for exclusion: off topic**

Bombak AE, Monaghan LF. Obesity, bodily change and health identities: a qualitative study of Canadian women. Sociology of Health & Illness. 2017;39(6):923–40.

**Reason for exclusion: method unsuitable**

Bomberg E, Hague A. Faith-based climate action in Christian congregations: mobilisation and spiritual resources. Local Environment. 2018;23(5):582–96.

**Reason for exclusion: subject**

Bond RJ, Hurst J. How Adults with Learning Disabilities View Living Independently. British Journal of Learning Disabilities. 2010;38(4):286–92.

**Reason for exclusion: subject matter**

Bond V, Chilikwela L, Simwinga M, Reade Z, Ayles H, Godfrey-Faussett P, et al. Children’s role in enhanced case finding in Zambia. International Journal of Tuberculosis and Lung Disease. 2010;14(10):1280–7.

**Reason for exclusion: off topic**

Bond V, Ngwenya F, Murray E, Ngwenya N, Viljoen L, Gumede D, et al. Value and Limitations of Broad Brush Surveys Used in Community-Randomized Trials in Southern Africa. Qualitative Health Research. 2019;29(5):700–18.

**Reason for exclusion: off topic**

Bono R, Bugliosi EH, Schiliro T, Gilli G. The Lagrange Street story: the prevention of aromatics air pollution during the last nine years in a European city. Atmospheric Environment. 2001;35:S107–13.

**Reason for exclusion: subject**

Boon B, Risselada A, Huiberts A, Riper H, Smit F. Curbing alcohol use in male adults through computer generated personalized advice: randomized controlled trial. Journal of Medical Internet Research. 2011;13(2):e43.

**Reason for exclusion: subject matter**

Booth CS, Torres T, Bowles L, Butler T. A qualitative study to evaluate blood film morphology teaching for biomedical scientists led by haematology registrars. British Journal of Haematology. 2019;185 (Supplement 1):49–50.

**Reason for exclusion: subject matter**

Borghi L. The monuments men: In the history of anesthesia, too. Anesthesiology. 2015;122(3):521–3.

**Reason for exclusion: subject matter**

Borie M, Pelling M, Ziervogel G, Hyams K. Mapping narratives of urban resilience in the global south. Global Environmental Change-Human and Policy Dimensions. 2019;54:203–13.

**Reason for exclusion: method unsuitable**

Borland R. CEOS Theory: A Comprehensive Approach to Understanding Hard to Maintain Behaviour Change. Applied Psychology-Health and Well Being. 2017;9(1):3–35.

**Reason for exclusion: subject**

Borrero S, Nikolajski C, Steinberg JR, Freedman L, Akers AY, Ibrahim S, et al. It just happens: A qualitative study exploring low-income women’s perspectives on pregnancy intention and planning. Contraception. 2015;91(2):150–6.

**Reason for exclusion: not storytelling**

Boschi B, Giotti I, Pelo E, Ricci U. Study by next generation sequencing of sudden cardiac death (SCD). Forensic Science International: Genetics Supplement Series. 2019;7(1):158–60.

**Reason for exclusion: off topic**

Botelho S, Martinho N, Regina Silva V, Marques J, Fernandes M, Riccetto C. The pelvic pigs and the big bad blow: Showing the importance of daily activity care for urogynecological health. Neurourology and Urodynamics. 2015;3):S395–6.

**Reason for exclusion: subject matter**

Botfield JR, Newman CE, Lenette C, Albury K, Zwi AB %J HEJ. Using digital storytelling to promote the sexual health and well-being of migrant and refugee young people: A scoping review. 2018;77(7):735–48.

**Reason for exclusion: not primary study**

Bottorff JL, Grewal SK, Balneaves LG, Naidu P, Johnson JL, Sawhney R. Punjabi women’s stories of breast cancer symptoms: Gulti (lumps), bumps, and darad (pain). Cancer Nursing. 2007;30(4):E36–45.

**Reason for exclusion: no short term sacrifice, long term gain**

Bottorff JL, Johnson JL, Bhagat R, Grewal S, Balneaves LG, Clarke H, et al. Beliefs related to breast health practices: The perceptions of South Asian women living in Canada. Social Science and Medicine. 1998;47(12):2075–85.

**Reason for exclusion: method unsuitable**

Bottorff JL, Johnson JL, Carey J, Hutchinson P, Sullivan D, Mowatt R, et al. A family affair: Aboriginal women’s efforts to limit second-hand smoke exposure at home. Canadian Journal of Public Health Revue Canadienne de Sante Publique. 2010;101(1):32–5.

**Reason for exclusion: method unsuitable**

Bottorff JL, Radsma J, Kelly M, Oliffe JL. Fathers’ narratives of reducing and quitting smoking. Sociology of Health & Illness. 2009;31(2):185–200.

**Reason for exclusion: method unsuitable**

Bouchard S, Gervais J, Gagnier N, Loranger C. Evaluation of a primary prevention program for anxiety disorders using story books with children aged 9-12 years. The Journal of Primary Prevention. 2013;34(5):345–58.

**Reason for exclusion: subject matter**

Boulaire C, Hervet G, Graf R. Creativity chains and playing in the crossfire on the video-sharing site YouTube. Journal of Research in Interactive Marketing. 2010;4(2):111–41.

**Reason for exclusion: off topic**

Bould H, Mars B, Moran P. Figure: The effectiveness of storytelling on raising awareness about health. 2019;

**Reason for exclusion: can't find study**

Bouman M %J EJ of PH. Storytelling makes public health statistics more accessibleMartine Bouman. 2017;27(suppl_3).

**Reason for exclusion: not primary study**

Bourgoin J. Sharpening the understanding of socio-ecological landscapes in participatory land-use planning. A case study in Lao PDR. Applied Geography. 2012;34:99–110.

**Reason for exclusion: method unsuitable**

Bowa K, Kawimbe B, Mugala D, Musowoya D, Makupe A, Njobvu M, et al. A Review of HIV and Surgery in Africa. The open AIDS journal. 2016;10:16–23.

**Reason for exclusion: not primary study**

Boyd E. Governing the Clean Development Mechanism: global rhetoric versus local realities in carbon sequestration projects. Environment and Planning A. 2009;41(10):2380–95.

**Reason for exclusion: method unsuitable**

Bozlak CT, Becker AB. Using evidence to create active communities: stories from the field-policy and research with Chicago’s child care centers: a commentary to accompany the Active Living Research Supplement to Annals of Behavioral Medicine. Annals of Behavioral Medicine. 2013;45 Suppl 1:S11-3.

**Reason for exclusion: subject matter**

Brabcova D, Kohout J, Weberova V, Komarek V. Educational video and story as effective interventions reducing epilepsy-related stigma among children. Epilepsy and Behavior. 2017;69:12–7.

**Reason for exclusion: subject matter**

Bradford LEA, Bharadwaj LA. Whiteboard animation for knowledge mobilization: a test case from the Slave River and Delta, Canada. International Journal of Circumpolar Health. 2015;74.

**Reason for exclusion: subject matter**

Bradshaw J. UK policy: A success story? Paediatrics & Child Health. 2007;12(8):681–5.

**Reason for exclusion: off topic**

Braga CP, d’Oliveira A. dChild and adolescent mental health policy: history and paths to participation. Ciencia & Saude Coletiva. 2019;24(2):401–10.

**Reason for exclusion: method unsuitable**

Bragin V, Shereshevsky G, Bragin I, Slobod E, Silenko T, Temnogorod M, et al. Cognitive assessment in dementia: The usefulness of the expanded version of the mini-mental status examination. Alzheimer’s and Dementia. 2015;1):P389.

**Reason for exclusion: subject matter**

Brand A, Gao L, Hamann A, Crayen C, Brand H, Squier SM, et al. Medical graphic narratives to improve patient comprehension and periprocedural anxiety before coronary angiography and percutaneous coronary intervention: A randomized trial. Annals of Internal Medicine. 2019;170(8):579–81.

**Reason for exclusion: subject matter**

Brandon PR, Smith NL, Grob GF. Five Years of HHS Home Health Care Evaluations: Using Evaluation to Change National Policy. American Journal of Evaluation. 2012;33(2):251–62.

**Reason for exclusion: not primary study**

Brashers DE, Neidig JL, Cardillo LW, Dobbs LK, Russell JA, Haas SM. “In an important way, I did die”: Uncertainty and revival in persons living with HIV or AIDS. AIDS Care - Psychological and Socio-Medical Aspects of AIDS/HIV. 1999;11(2):201–19.

**Reason for exclusion: subject matter**

Braten I, Stromso HI, Samuelstuen MS. Are sophisticated students always better? The role of topic-specific personal epistemology in the understanding of multiple expository texts. Contemporary Educational Psychology. 2008;33(4):814–40.

**Reason for exclusion: method unsuitable**

Braun LA, Kennedy HP, Sadler LS, Dixon J, Womack J, Wilson C. US Navy Women’s Experience of an Abnormal Cervical Cancer Screening. Journal of Midwifery and Women’s Health. 2016;61(2):249–56.

**Reason for exclusion: off topic**

Bravo MT. Voices from the sea ice: the reception of climate impact narratives. Journal of Historical Geography. 2009;35(2):256–78.

**Reason for exclusion: method unsuitable**

Breivik JK. Vulnerable but strong: Deaf people challenge established understandings of deafness. Scandinavian Journal of Public Health. 2005;33(SUPPL. 66):18–23.

**Reason for exclusion: subject matter**

Bremer S, Blanchard A, Mamnun N, Stiller-Reeve M, Haque MM, Tvinnereim E. Narrative as a Method for Eliciting Tacit Knowledge of Climate Variability in Bangladesh. Weather Climate and Society. 2017;9(4):669–86.

**Reason for exclusion: method unsuitable**

Brenes CS. The practical value of theory: Teaching aristotle’s poetics to screenwriters. Communication and Society. 2011;24(1):101–17.

**Reason for exclusion: off topic**

Breton H, Rossi S. Life stories, reciprocity of knowledge and therapeutic patient education. Medecine des Maladies Metaboliques. 2017;11(7):612–5.

**Reason for exclusion: subject matter**

Breuning M, Schafer-Fauth L, Lucius-Hoene G, Holmberg C. Connecting one’s own illness story to the illness experiences of others on a website-An evaluation study using the think aloud method. Patient Education and Counseling. 2020;103(1):199–207.

**Reason for exclusion: subject matter**

Bricker PL, Baron RJ, Scheirer JJ, DeWalt DA, Derrickson J, Yunghans S, et al. Collaboration in Pennsylvania: Rapidly Spreading Improved Chronic Care for Patients to Practices. Journal of Continuing Education in the Health Professions. 2010;30(2):114–25.

**Reason for exclusion: subject matter**

Brimnes N. BCG vaccination and WHO’s global strategy for tuberculosis control 1948-1983. Social Science & Medicine. 2008;67(5):863–73.

**Reason for exclusion: subject matter**

Brindle L. Narratives of Health Protection in Families with a Late-Onset Kidney Disease: Re-Defining Governmentality and Responsibility for Health in the Era of the “New” Genetics. Sociological Research Online. 2009;14(5).

**Reason for exclusion: method unsuitable**

Brisbois B. Bananas, pesticides and health in southwestern Ecuador: A scalar narrative approach to targeting public health responses. Social Science and Medicine. 2016;150:184–91.

**Reason for exclusion: subject matter**

Brockhaus M, Djoudi H, Kambire H. Multi-level governance and adaptive capacity in West Africa. International Journal of the Commons. 2012;6(2):200–32.

**Reason for exclusion: subject**

Broglia RA. Learning to design resistance proof drugs from folding. European Physical Journal D. 2009;51(1):137–51.

**Reason for exclusion: subject**

Brookes VJ, Degeling C, Ward MP. Going viral in PNG - Exploring routes and circumstances of entry of a rabies-infected dog into Papua New Guinea. Social Science & Medicine. 2018;196:10–8.

**Reason for exclusion: subject matter**

Brooks H, Rushton K, Walker S, Lovell K, Rogers A. Ontological security and connectivity provided by pets: a study in the self-management of the everyday lives of people diagnosed with a long-term mental health condition. BMC Psychiatry. 2016;16.

**Reason for exclusion: subject**

Brown BD, Noonan C, Harris KJ, Parker M, Wilson T, Gress S, et al. Diabetes prevention program for native american youth: The journey to native youth health feasibility study. Diabetes. 2011;1):A82.

**Reason for exclusion: off topic**

Brown I, Martin-Ortega J, Waylen K, Blackstock K. Participatory scenario planning for developing innovation in community adaptation responses: three contrasting examples from Latin America. Regional Environmental Change. 2016;16(6):1685–700.

**Reason for exclusion: subject**

Brown LD, Alter TR, Brown LG, Corbin MA, Flaherty-Craig C, McPhail LG, et al. Rural Embedded Assistants for Community Health (REACH) network: first-person accounts in a community-university partnership. American Journal of Community Psychology. 2013;51(1–2):206–16.

**Reason for exclusion: subject matter**

Brown M, Houghton R, Sharples S, Morley J. The attribution of success when using navigation aids. Ergonomics. 2015;58(3):426–33.

**Reason for exclusion: subject**

Brown P. Qualitative methods in environmental health research. Environmental Health Perspectives. 2003;111(14):1789–98.

**Reason for exclusion: not primary study**

Brown RP, Phillips A. Letting bygones be bygones: further evidence for the validity of the Tendency to Forgive scale. Personality and Individual Differences. 2005;38(3):627–38.

**Reason for exclusion: subject**

Brown T. Asia. Vancouver Conference Review. AIDS Care. 1997;9(1):43–9.

**Reason for exclusion: off topic**

Browne AL, Pullinger M, Medd W, Anderson B. Patterns of practice: a reflection on the development of quantitative/mixed methodologies capturing everyday life related to water consumption in the UK. International Journal of Social Research Methodology. 2014;17(1):27–43.

**Reason for exclusion: subject**

Brownson RC, Dodson EA, Kerner JF, Moreland-Russell S. Framing research for state policymakers who place a priority on cancer. Cancer Causes & Control. 2016;27(8):1035–41.

**Reason for exclusion: method unsuitable**

Brucker MC. Become an author and share your knowledge. Nursing for Women’s Health. 2013;17(4):269–70.

**Reason for exclusion: off topic**

Brug J, Kremers SP, van Lenthe F, Ball K, Crawford D. Environmental determinants of healthy eating: in need of theory and evidence. Proceedings of the Nutrition Society. 2008;67(3):307–16.

**Reason for exclusion: method unsuitable**

Brug J. Determinants of healthy eating: motivation, abilities and environmental opportunities. Family Practice. 2008;25:I50–5.

**Reason for exclusion: method unsuitable**

Brugge D, Edgar T, George K, Heung J, Laws B. Beyond literacy and numeracy in patient provider communication: Focus groups suggest roles for empowerment, provider attitude and language. BMC Public Health. 2009;9.

**Reason for exclusion: method unsuitable**

Brunner LK, Dever M. Work, Bodies and Boundaries: Talking sexual harassment in the New Economy. Gender, Work and Organization. 2014;21(5):459–71.

**Reason for exclusion: off topic**

Brunsberg K, Portillo E, Miller K, Critser P, Mateo C, Sectish T, et al. Dream: Developing resident emotional and mental health. Academic pediatrics. 2017;17 (5):e44.

**Reason for exclusion: subject matter**

Brusse EDA, Fransen ML, Smit EG. Framing in Entertainment-Education: Effects on Processes of Narrative Persuasion. Health Communication. 2017;32(12):1501–9.

**Reason for exclusion: not primary study**

Bryant E. A matter of planning the family. The Earth Times. 1996;3.

**Reason for exclusion: not primary study**

Bucher Della Torre S, Dudley-Martin F, Kruseman M. “Croque&bouge”: A feasible and acceptable programme for obesity prevention in preschoolers at risk and their parents. SAGE Open Medicine. 2015;3:2050312115574365.

**Reason for exclusion: not primary study**

Buchmann M, Wermeling M, Lucius-Hoene G, Himmel W. Experiences of food abstinence in patients with type 2 diabetes: a qualitative study. BMJ Open. 2016;6(1).

**Reason for exclusion: method unsuitable**

Bukowski R, Hansen NI, Willinger M, Reddy UM, Parker CB, Pinar H, et al. Fetal Growth and Risk of Stillbirth: A Population-Based Case-Control Study. Plos Medicine. 2014;11(4).

**Reason for exclusion: method unsuitable**

Buman MP, Bertmann F, Hekler EB, Winter SJ, Sheats JL, King AC, et al. A qualitative study of shopper experiences at an urban farmers’ market using the Stanford Healthy Neighborhood Discovery Tool. Public Health Nutrition. 2015;18(6):994–1000.

**Reason for exclusion: subject matter**

Bunker A, Barnighausen T, Woodward A, Bullen C. Housing structure and occupant behaviour to increase the environmental and health co-benefits of housing: Insights from expert interviews in New Zealand. Indoor and Built Environment. 2020;

**Reason for exclusion: subject matter**

Burchardt M. The self as capital in the narrative economy: how biographical testimonies move activism in the Global South. Sociology of Health & Illness. 2016;38(4):592–609.

**Reason for exclusion: off topic**

Burgdorf WHC, Hoenig LJ, Plewig G, Kohl PK. Erich Langer: The last Jewish dermatologist in Nazi Berlin. Clinics in Dermatology. 2014;32(4):532–41.

**Reason for exclusion: off topic**

Burgess RA. Policy, power, stigma and silence: Exploring the complexities of a primary mental health care model in a rural South African setting. Transcultural Psychiatry. 2016;53(6):719–42.

**Reason for exclusion: method unsuitable**

Burk L. Warning dreams preceding the diagnosis of breast cancer: A survey of the most important characteristics. Explore: The Journal of Science and Healing. 2015;11(3):193–8.

**Reason for exclusion: off topic**

Burke D, Link N, Bails D, Fang YX, Janjigian MP. A taxonomy of seven-day readmissions to an urban teaching hospital. Journal of Hospital Medicine. 2016;11(1):33–8.

**Reason for exclusion: off topic**

Burke JD. Headlines tell the story, doctors drive it home. Wisconsin Medical Journal. 1996;95(11):759.

**Reason for exclusion: not primary study**

Burkiewicz JS, Fit KE. Improving adherence--sharing experiences. Annals of Pharmacotherapy. 2007;41(12):2058–60.

**Reason for exclusion: subject matter**

Burnett C. Being together in classrooms at the interface of the physical and virtual: implications for collaboration in on/off-screen sites. Learning, Media and Technology. 2016;41(4):566–89.

**Reason for exclusion: off topic**

Burnett E, Lee K, Rushmer R, Ellis M, Noble M, Davey P. Healthcare-associated infection and the patient experience: a qualitative study using patient interviews. Journal of Hospital Infection. 2010;74(1):42–7.

**Reason for exclusion: subject**

Burris J, Cook-Deegan R, Alberts B. The Human Genome Project after a decade: policy issues. Nature Genetics. 1998;20(4):333–5.

**Reason for exclusion: off topic**

Burtscher D, Burza S. Health-seeking behaviour and community perceptions of childhood undernutrition and a community management of acute malnutrition (CMAM) programme in rural Bihar, India: a qualitative study. Public Health Nutrition. 2015;18(17):3234–43.

**Reason for exclusion: method unsuitable**

Bushell S, Buisson GS, Workman M, Colley T. Strategic narratives in climate change: Towards a unifying narrative to address the action gap on climate change. Energy Research & Social Science. 2017;28:39–49.

**Reason for exclusion: off topic**

Buston K, Parkes A, Wight D. High and low contraceptive use amongst young male offenders: a qualitative interview study. Journal of Family Planning and Reproductive Health Care. 2014;40(4):248–53.

**Reason for exclusion: method unsuitable**

Bute JJ, Jensen RE. Narrative Sensemaking and Time Lapse: Interviews with Low-income Women about Sex Education. Communication Monographs. 2011;78(2):212–32.

**Reason for exclusion: method unsuitable**

Butler C, Harris J. Pills, ills and the ugly face of aesthetic labour: “They should”ve discriminated against me’. Work, Employment and Society. 2015;29(3):508–16.

**Reason for exclusion: subject matter**

Buzzard S. Appropriate research for primary health care: an anthropologist’s view. Social Science & Medicine. 1984;19(3):273–7.

**Reason for exclusion: subject matter**

Byars D. Vertically integrated multidisciplinary multimedia (vimm) modules to teach ultrasound in emergency medicine, an acep teaching fellowship project. Annals of Emergency Medicine. 2011;1):S332.

**Reason for exclusion: subject matter**

Bygdeson-Larsson K. Giving Voice to the Swedish Pre-School Child: Inclusion through Educational Process Reflection. Child Care in Practice. 2005;11(2):161–77.

**Reason for exclusion: subject matter**

Caballero-Hoyos R, Villasenor-Farias M, Hidalgo-San Martin A, Pando-Moreno M. Vaccination messages favor mobilization and high coverage in Mexico. [Spanish]. Gaceta Medica de Mexico. 2002;138(1):31–40.

**Reason for exclusion: off topic**

Caburnay CA, Kreuter MW, Cameron G, Luke DA, Cohen EL, McDaniels L, et al. Black newspapers as a tool for cancer education in African American communities. Ethnicity & Disease. 2008;18(4):488–95.

**Reason for exclusion: not primary study**

Cai T, Steinfield C, Chiwasa H, Ganunga T. Understanding Malawian farmers’ slow adoption of composting: Stories about composting using a participatory video approach. Land Degradation and Development. 2019;30(11):1336–44.

**Reason for exclusion: subject matter**

Cairney P, Oliver K. Evidence-based policymaking is not like evidence-based medicine, so how far should you go to bridge the divide between evidence and policy? Health Research Policy & Systems. 2017;15(1):35.

**Reason for exclusion: off topic**

Caldwell C. Story structure for programmers, designers, animators, and artists. In: SA 2016 - SIGGRAPH ASIA 2016 Courses. 2016.

**Reason for exclusion: not primary study**

Caldwell C. Story: It’s not just for writers... anymore. In: ACM SIGGRAPH 2013 Courses, SIGGRAPH 2013. 2013.

**Reason for exclusion: not primary study**

Caldwell C. Story: It’s not just for writers⋯ anymore! In: ACM SIGGRAPH 2018 Courses, SIGGRAPH 2018. 2018.

**Reason for exclusion: not primary study**

Caldwell C. Story: It’s not just for writers⋯ anymore: Course Notes. In: ACM SIGGRAPH 2016 Courses, SIGGRAPH 2016. 2016.

**Reason for exclusion: not primary study**

Caldwell JC. Demographers’ involvement in twentieth-century population policy: Continuity or discontinuity? Population Research and Policy Review. 2005;24(4):359–85.

**Reason for exclusion: method unsuitable**

Califf RM. The coxib story: Some lessons and more questions. American Journal of Cardiology. 2002;89(8):971–2.

**Reason for exclusion: subject matter**

Cameron G, Wren AM. Reconstructing organizational culture: a process using multiple perspectives. Public Health Nursing. 1999;16(2):96–101.

**Reason for exclusion: subject matter**

Cameron K, Crooks VA, Chouinard V, Snyder J, Johnston R, Casey V. Motivation, justification, normalization: Talk strategies used by Canadian medical tourists regarding their choices to go abroad for hip and knee surgeries. Social Science and Medicine. 2014;106:93–100.

**Reason for exclusion: subject matter**

Camou F, Alessandrin A, Toussaint E, Eyquard F, Bonnouvrier M, Raymound I. Gaucher disease: Lived experience of patients in the context of a French national patient therapeutic education program. Molecular Genetics and Metabolism. 2015;114 (2):S28–9.

**Reason for exclusion: subject matter**

Campos L, Cavalcante JP, Machado DP, Marcal E, Silva PGB, Rolim J. Development and Evaluation of a Mobile Oral Health Application for Preschoolers. Telemedicine Journal & E Health. 2018;23:23.

**Reason for exclusion: subject matter**

Campos LFXA, Cavalcante JP, Machado DP, Marçal E, Silva PGDB, Rolim JPML. Development and Evaluation of a Mobile Oral Health Application for Preschoolers. Telemedicine and e-Health. 2019;25(6):492–8.

**Reason for exclusion: subject matter**

Canary H, Bullis C, Cummings J, Kinney AY. Structuring Health in Colorectal Cancer Screening Conversations: An Analysis of Intersecting Activity Systems. Southern Communication Journal. 2015;80(5):416–32.

**Reason for exclusion: not primary study**

Cant RP, Aroni RA. Exploring dietitians’ verbal and nonverbal communication skills for effective dietitian-patient communication. Journal of Human Nutrition and Dietetics. 2008;21(5):502–11.

**Reason for exclusion: off topic**

Cappuccio A, Bugliaro F, Caimmi SME, Caldarelli V, Caminiti L, D’Auria E, et al. Consensus communication strategies to improve doctor-patient relationship in paediatric severe asthma. Italian Journal of Pediatrics. 2019;45 (1) (no pagination)(31).

**Reason for exclusion: off topic**

Capstick S, Hemstock S, Senikula R. Perspectives of artist-practitioners on the communication of climate change in the Pacific. International Journal of Climate Change Strategies and Management. 2018;10(2):323–39.

**Reason for exclusion: off topic**

Caputo A. Exploring quality of life in Italian patients with rare disease: a computer-aided content analysis of illness stories. Psychology Health & Medicine. 2014;19(2):211–21.

**Reason for exclusion: off topic**

Carbaugh D. “Just listen”: “Listening” and landscape among the blackfeet. Western Journal of Communication. 1999;63(3):250–70.

**Reason for exclusion: off topic**

Care MN, Kuiper NA. Cognitive Representations in a Self-regulation Model of Depression: Effects of Self-Other Distinctions, Symptom Severity and Personal Experiences with Depression. Self and Identity. 2013;12(2):128–45.

**Reason for exclusion: method unsuitable**

Carillo E, Kadobayashi R, Chikama M, Shimojo S, Rodriguez-Echavarria K, Arnold D. Making history alive and interactive: Designing an iPhone app to present the summer war of Osaka Byōbu. In: 2010 16th International Conference on Virtual Systems and Multimedia, VSMM 2010. 2010. p. 365–8.

**Reason for exclusion: off topic**

Carlisle P. Religion and Spirituality as Troublesome Knowledge: The Views and Experiences of Mental Health Social Workers in Northern Ireland. British Journal of Social Work. 2016;46(3):583–98.

**Reason for exclusion: off topic**

Carothers C, Sformo TL, Cotton S, George JC, Westley PAH. Pacific salmon in the rapidly changing arctic: Exploring local knowledge and emerging fisheries in Utqiaġvik and Nuiqsut, Alaska. Arctic. 2019;72(3):273–88.

**Reason for exclusion: subject matter**

Carpenter-Song E, Chu E, Drake RE, Ritsema M, Smith B, Alverson H. Ethno-Cultural Variations in the Experience and Meaning of Mental Illness and Treatment: Implications for Access and Utilization. Transcultural Psychiatry. 2010;47(2):224–51.

**Reason for exclusion: no short term sacrifice, long term gain**

Carragher V, O’Regan B, Peters M, Moles R. Novel resource saving interventions: the case of modelling and storytelling. Local Environment. 2018;23(5):518–35.

**Reason for exclusion: method unsuitable**

Carter OB, Mills BW, Lloyd E, Phan T. An independent audit of the Australian food industry’s voluntary front-of-pack nutrition labelling scheme for energy-dense nutrition-poor foods. European Journal of Clinical Nutrition. 2013;67(1):31–5.

**Reason for exclusion: off topic**

Cartwright C, Gibson K, Read J, Cowan O, Dehar T. Long-term antidepressant use: patient perspectives of benefits and adverse effects. Patient Preference and Adherence. 2016;10:1401–7.

**Reason for exclusion: method unsuitable**

Caruso Brown AE, Suryadevara M, Welch TR, Botash AS. “Being Persistent without Being Pushy”: Student Reflections on Vaccine Hesitancy. Narrative Inquiry in Bioethics. 2017;7(1):59–70.

**Reason for exclusion: off topic**

Carver T. Making Marx Marx. Journal of Classical Sociology. 2017;17(1):57–74.

**Reason for exclusion: off topic**

Casement W. Whither the Great Books? Academic Questions. 2002;15(4):36–51.

**Reason for exclusion: off topic**

Cash SJ, Berry M. Measuring service delivery in a placement prevention program: An application to an ecological model. Administration in Social Work. 2003;27(3):65–85.

**Reason for exclusion: subject**

Cassel CK. Narratives on pain and comfort: Dr. M’s story. Journal of Law, Medicine & Ethics. 1996;24(4):290–1.

**Reason for exclusion: off topic**

Cassidy A. “Big science” in the field: experimenting with badgers and bovine TB, 1995-2015. Pubblicazioni della Stazione Zoologica di Napoli - Section Ii: History & Philosophy of the Life Sciences. 2015;37(3):305–25.

**Reason for exclusion: subject**

Cassidy BE, Alabanza-Akers MA, Akers TA, Hall DB, Ryan PB, Bayer CW, et al. Particulate matter and carbon monoxide multiple regression models using environmental characteristics in a high diesel-use area of Baguio City, Philippines. Science of the Total Environment. 2007;381(1–3):47–58.

**Reason for exclusion: subject**

Cates JR, Shafer A, Carpentier FD, Reiter PL, Brewer NT, McRee AL, et al. How parents hear about human papillomavirus vaccine: implications for uptake. Journal of Adolescent Health. 2010;47(3):305–8.

**Reason for exclusion: off topic**

Cavanaugh N, Cheney KS. Community collaboration--a weaving. Journal of Public Health Management & Practice. 2002;8(1):13–20.

**Reason for exclusion: off topic**

Cavazza M, Martin O, Charles F, Marichal X, Mead SJ. User interaction in mixed reality interactive storytelling. In: Proceedings - 2nd IEEE and ACM International Symposium on Mixed and Augmented Reality, ISMAR 2003. 2003. p. 304–5.

**Reason for exclusion: off topic**

Cavill N. Keynote. Journal of Science and Medicine in Sport. 2012;15 (SUPPL.1):S221.

**Reason for exclusion: off topic**

Cawkwell PB, Oshinsky D. Childhood vaccination requirements: Lessons from history, Mississippi, and a path forward. Vaccine. 2015;33(43):5884–7.

**Reason for exclusion: off topic**

Caxaj CS %J G qualitative nursing research. Indigenous storytelling and participatory action research: Allies toward decolonization? Reflections from the peoples’ international health tribunal. 2015;2:2333393615580764.

**Reason for exclusion: subject matter**

Caxaj CS. Indigenous Storytelling and Participatory Action Research: Allies Toward Decolonization? Reflections From the Peoples’ International Health Tribunal. Global Qualitative Nursing Research. 2015;2:2333393615580764.

**Reason for exclusion: not primary study**

Cedraschi C, Saya L, Klein P, Bordet MF, Carrat F. Representations of influenza and influenza-like illness in the community - a qualitative study. BMC Family Practice. 2013;14.

**Reason for exclusion: method unsuitable**

Cerully JL, Collins RL, Wong E, Seelam R, Yu J. Differential response to contact-based stigma reduction programs: Perceived quality and personal experience matter. Psychiatry Research. 2018;259:302–9.

**Reason for exclusion: off topic**

Chakraborty B, Yousefzadeh S, Darak S, Haisma H. “we struggle with the earth everyday”: Parents’ perspectives on the capabilities for healthy child growth in haor region of Bangladesh. BMC Public Health. 2020;20(1).

**Reason for exclusion: no short term sacrifice, long term gain**

Champion PD. Knowledge to action on aquatic invasive species: Island biosecurity - the New Zealand and South Pacific story. Management of Biological Invasions. 2018;9(4):383–94.

**Reason for exclusion: method unsuitable**

Chan AW, Skeffington P, Reid C, Marriott R. Research protocol for the exploration of experiences of Aboriginal Australian mothers and healthcare professionals when using the Edinburgh Postnatal Depression Scale: a process-oriented validation study using triangulated participatory mixed methods. BMJ Open. 2018;8(10).

**Reason for exclusion: method unsuitable**

Chan FKS, Chuah CJ, Ziegler AD, Dabrowski M, Varis O. Towards resilient flood risk management for Asian coastal cities: Lessons learned from Hong Kong and Singapore. Journal of Cleaner Production. 2018;187:576–89.

**Reason for exclusion: method unsuitable**

Chandler D. Creating the lion king: Story development, authorship and accreditation in the disney renaissance. Journal of Screenwriting. 2018;9(3):329–45.

**Reason for exclusion: off topic**

Chang JS, Behar E, Coffin PO. Narratives of people who inject drugs on factors contributing to opioid overdose. International Journal of Drug Policy. 2019;74:26–32.

**Reason for exclusion: no short term sacrifice, long term gain**

Chang VW, Christakis NA. Medical modelling of obesity: a transition from action to experience in a 20(th) century American medical textbook. Sociology of Health & Illness. 2002;24(2):151–77.

**Reason for exclusion: subject**

Chanson P. [Historical perspective of hormone replacement therapy]. Revue du Praticien. 2005;55(4):369–75.

**Reason for exclusion: subject matter**

Chapman E, Kipp W, Rubaale T. “...No stone left unturned:” how the public explains the Ugandan success story. World Health & Population [Electronic Resource]. 2008;10(3):5–13.

**Reason for exclusion: off topic**

Chapman E, Kipp W, Rubaale T. “No Stone Left Unturned”: how the public explains the Ugandan success story. Healthcare Quarterly. 2009;12(2):128–33.

**Reason for exclusion: off topic**

Chapman RS, Seung HK, Schwartz SE, Bird EK. Predicting language production in children and adolescents with Down syndrome: the role of comprehension. Journal of speech, language, and hearing research : JSLHR. 2000;43(2):340–50.

**Reason for exclusion: subject matter**

Charles A, Felton A. Exploring young people’s experiences and perceptions of mental health and well-being using photography. Child and Adolescent Mental Health. 2020;25(1):13–20.

**Reason for exclusion: no short term sacrifice, long term gain**

Chase RM, Medina MF, Mignone J. The Life Story Board: A Feasibility Study of a Visual Interview Tool for School Counsellors. Canadian Journal of Counselling and Psychotherapy. 2012;46(3):183–200.

**Reason for exclusion: subject matter**

Chatwin J, Povey A, Kennedy A, Frank T, Firth A, Booton R, et al. The mediation of social influences on smoking cessation and awareness of the early signs of lung cancer. BMC Public Health. 2014;14.

**Reason for exclusion: method unsuitable**

Chatzidamianos G, Lobban F, Jones S. A qualitative analysis of relatives’, health professionals’ and service users’ views on the involvement in care of relatives in Bipolar Disorder. BMC Psychiatry. 2015;15.

**Reason for exclusion: subject**

Chaudhury AS, Thornton TF, Helfgott A, Sova C. Applying the robust adaptation planning (RAP) framework to Ghana’s agricultural climate change adaptation regime. Sustainability Science. 2017;12(5):657–76.

**Reason for exclusion: off topic**

Chebut O, Medeiros De Bustos E, Moulin T. The story of a sleeping monster - A case report; one of the new treatments that changed the face of modern neurology can be put in danger. European Journal of Neurology. 2011;2):112.

**Reason for exclusion: subject matter**

Chen CH, Lee IJ, Lin LY. Augmented reality-based video-modeling storybook of nonverbal facial cues for children with autism spectrum disorder to improve their perceptions and judgments of facial expressions and emotions. Computers in Human Behavior. 2016;55:477–85.

**Reason for exclusion: method unsuitable**

Chen CY, Maloof AC. Revisiting the deformed high shoreline of Lake Bonneville. Quaternary Science Reviews. 2017;159:169–89.

**Reason for exclusion: method unsuitable**

Chen CY, Wu YCJ, Wu WH. A sustainable collaborative research dialogue between practitioners and academics. Management Decision. 2013;51(3):566–93.

**Reason for exclusion: off topic**

Chen H, Nester JM, Yo HJ. Acausal PGT modes and the nonlinear constraint effect. Acta Physica Polonica B. 1998;29(4):961–70.

**Reason for exclusion: method unsuitable**

Chen P, Chai J, Cheng J, Li K, Xie S, Liang H, et al. A smart web aid for preventing diabetes in rural China: preliminary findings and lessons. Journal of Medical Internet Research. 2014;16(4):e98.

**Reason for exclusion: subject matter**

Chen PC, Sim HC. The development of culture-specific health education packages to increase case-finding of leprosy in Sarawak. Southeast Asian Journal of Tropical Medicine & Public Health. 1986;17(3):427–32.

**Reason for exclusion: subject matter**

Chen PC. Human behavioural research applied to the leprosy control programme of Sarawak, Malaysia. Southeast Asian Journal of Tropical Medicine & Public Health. 1986;17(3):421–6.

**Reason for exclusion: subject matter**

Chen SB, Gunster S. “Ethereal Carbon”: legitimizing liquefied natural gas in British Columbia. Environmental Communication-a Journal of Nature and Culture. 2016;10(3):305–21.

**Reason for exclusion: off topic**

Chen XZ, Doerge RW, Heyse JF. Multiple testing with discrete data: Proportion of true null hypotheses and two adaptive FDR procedures. Biometrical Journal. 2018;60(4):761–79.

**Reason for exclusion: off topic**

Cheng CC. Polycyclic aromatic hydrocarbon concentration and acute toxicity of airborne particulate matter: Using microtox as a toxicity screening tool. Polycyclic Aromatic Compounds. 2003;23(3):249–58.

**Reason for exclusion: off topic**

Chepngeno-Langat G, Falkingham JC, Madise NJ, Evandrou M. Concern About HIV and AIDS Among Older People in the Slums of Nairobi, Kenya. Risk Analysis. 2012;32(9):1512–23.

**Reason for exclusion: off topic**

Cherry L. Young Voices on Climate Change: The Paul F-Brandwein 2010 NSTA Lecture. Journal of Science Education and Technology. 2011;20(2):208–13.

**Reason for exclusion: off topic**

Chetcuti K. Chest imaging and tuberculosis. Pediatric Radiology. 2019;49 (Supplement 2):S315–6.

**Reason for exclusion: subject matter**

Chew HSJ, Sim KLD, Cao X, Chair SY. Motivation, challenges and self-regulation in heart failure self-care: A theory-driven qualitative study. International Journal of Behavioral Medicine. 2019;No Pagination Specified.

**Reason for exclusion: off topic**

Chhabra R, Chisolm DJ, Bayldon B, Quadri M, Sharif I, Velazquez JJ, et al. Evaluation of Pediatric Human Papillomavirus Vaccination Provider Counseling Written Materials: A Health Literacy Perspective. Academic pediatrics. 2018;18(2S):S28–36.

**Reason for exclusion: off topic**

Chiarchiaro J, Ernecoff NC, Buddadhumaruk P, Rak KJ, Arnold RM, White DB. Key stakeholders’ perspectives on a Web-based advance care planning tool for advanced lung disease. Journal of Critical Care. 2015;30(6):1418.e7-1418.e12.

**Reason for exclusion: subject matter**

Chikermane V, Wong J, Hawa R, Chambers L, Vahabi M, Hari S. Storytelling for sexual health: A culturally relevant model to build HIV knowledge and capacity among South Asian women. Canadian Journal of Infectious Diseases and Medical Microbiology. 2015;SB):117B–118B.

**Reason for exclusion: can't locate study**

Childs E, Assoumou SA, Biello KB, Biancarelli DL, Drainoni ML, Edeza A, et al. Evidence-based and guideline-concurrent responses to narratives deferring HCV treatment among people who inject drugs. Harm Reduction Journal. 2019;16 (1) (no pagination)(14).

**Reason for exclusion: subject matter**

Chilibeck G, Lock M, Sehdev M. Postgenomics, uncertain futures, and the familiarization of susceptibility genes. Social Science and Medicine. 2011;72(11):1768–75.

**Reason for exclusion: off topic**

Chilvers J, Lorenzoni I, Terry G, Buckley P, Pinnegar JK, Gelcich S. Public engagement with marine climate change issues: (Re)framings, understandings and responses. Global Environmental Change-Human and Policy Dimensions. 2014;29:165–79.

**Reason for exclusion: method unsuitable**

Chinman M, Kloos B, O’Connell M, Davidson L. Service providers’ views of psychiatric mutual support groups. Journal of Community Psychology. 2002;30(4):349–66.

**Reason for exclusion: method unsuitable**

Chipperfield SR. The Effect of Group Diversity on Learning on a University-Based Foundation Course. Journal of Further and Higher Education. 2012;36(3):333–50.

**Reason for exclusion: off topic**

Chitson S, Paul R. Understanding first cervical screening experiences using narrative analysis of online blogs: a medical student perspective. Education for Primary Care. 2020;

**Reason for exclusion: no short term sacrifice, long term gain**

Chittaro L, Zuliani F. Exploring audio storytelling in mobile exergames to affect the perception of physical exercise. In: Proceedings of the 2013 7th International Conference on Pervasive Computing Technologies for Healthcare and Workshops, PervasiveHealth 2013. 2013. p. 1–8.

**Reason for exclusion: subject matter**

Chiu MYL, Wei GFW, Lee S. Personal tragedy or system failure: A qualitative analysis of narratives of caregivers of people with severe mental illness in Hong Kong and Taiwan. International Journal of Social Psychiatry. 2006;52(5):413–23.

**Reason for exclusion: method unsuitable**

Chou R, Qaseem A, High Value Care Task Force A. Cardiac Screening With Electrocardiography, Stress Echocardiography, or Myocardial Perfusion Imaging: Advice for High-Value Care From the American College of Physicians. Annals of Internal Medicine. 2015;162(6):438-U92.

**Reason for exclusion: method unsuitable**

Chowdhury A, Maiti SK, Bhattacharyya S. How to communicate climate change “impact and solutions” to vulnerable population of Indian Sundarbans? From theory to practice. Springerplus. 2016;5(1):1219.

**Reason for exclusion: off topic**

Choy S, Wärvik G-B. Integration of Learning for Refugee and Migrant Students: VET Teachers’ Practices through Practice Theory Lens. Journal of Vocational Education and Training. 2019;71(1):87–107.

**Reason for exclusion: subject matter**

Christensen JF, Bandak M, Campbell A, Jones LW, Hojman P. Treatment-related cardiovascular late effects and exercise training countermeasures in testicular germ cell cancer survivorship. Acta Oncologica. 2015;54(5):592–9.

**Reason for exclusion: subject matter**

Christianson M, Lalos A, Westman G, Johansson EE. “Eyes wide shut” - Sexuality and risk in HIV-positive youth in Sweden: A qualitative study. Scandinavian Journal of Public Health. 2007;35(1):55–61.

**Reason for exclusion: method unsuitable**

Christos L, Duijker G, Angelaki A, Tsiligianni IG, Anastasiou FS, Prokopiadou DP, et al. Practice-Based Research Network in Primary Care: A lacking story and learning points from an empirical model on Crete. European Journal of General Practice. 2014;20 (1):46–7.

**Reason for exclusion: subject matter**

Chu SL, Quek F. The effects of visual contextual structures on children’s imagination in story authoring interfaces. In: ACM International Conference Proceeding Series. 2014. p. 329–32.

**Reason for exclusion: discussion paper not a primary study, off topic**

Chu T, Hackett M, Kaur N. Exploring Caregiver Behavior and Knowledge about Unsafe Sleep Surfaces in Infant Injury Death Cases. Health Education & Behavior. 2015;42(3):293–301.

**Reason for exclusion: subject matter**

Chung B, Jones L, Terry C, Jones A, Forge N, Norris KC. Story of Stone Soup: a recipe to improve health disparities. Ethnicity & Disease. 2010;20(1 Suppl 2):S2-9–14.

**Reason for exclusion: subject matter**

Clark L. GENDER AND GENERATION IN POOR WOMENS HOUSEHOLD HEALTH PRODUCTION EXPERIENCES. Medical Anthropology Quarterly. 1993;7(4):386–402.

**Reason for exclusion: method unsuitable**

Clark MR, Treisman GJ. Optimizing treatment with opioids and beyond. Advances in Psychosomatic Medicine. 2011;30:92–112.

**Reason for exclusion: off topic**

Clarke AM, Sixsmith J, Barry MM. Evaluating the implementation of an emotional wellbeing programme for primary school children using participatory approaches. Health Education Journal. 2015;74(5):578–93.

**Reason for exclusion: subject matter**

Clarke DD. LIFE SCRIPTS - IMPLICIT REPRESENTATIONS OF LIFE-COURSE PATTERNS. Journal of Social Behavior and Personality. 1995;10(4):871–84.

**Reason for exclusion: off topic**

Clarkesmith DE, Lip GYH, Lane DA. Patients’ experiences of atrial fibrillation and non-vitamin K antagonist oral anticoagulants (NOACs), and their educational needs: A qualitative study. Thrombosis Research. 2017;153:19–27.

**Reason for exclusion: subject matter**

Clayman ML, Boberg EW, Makoul G. The use of patient and provider perspectives to develop a patient-oriented website for women diagnosed with breast cancer. Patient Education and Counseling. 2008;72(3):429–35.

**Reason for exclusion: method unsuitable**

Clayton S. Mythic structure in screenwriting. New Writing. 2007;4(3):208–23.

**Reason for exclusion: off topic**

Cleeve A, Faxelid E, Nalwadda G, Klingberg-Allvin M. Abortion as agentive action: reproductive agency among young women seeking post-abortion care in Uganda. Culture Health & Sexuality. 2017;19(11):1286–300.

**Reason for exclusion: method unsuitable**

Clement S, Jarrett M, Henderson C, Thornicroft G. Messages to use in population-level campaigns to reduce mental health-related stigma: consensus development study. Epidemiologia E Psichiatria Sociale-an International Journal for Epidemiology and Psychiatric Sciences. 2010;19(1):72–9.

**Reason for exclusion: method unsuitable**

Clift AK. Breast screening controversy and the “Mammography wars”-two sides to every story. Hong Kong Medical Journal. 2018;24(3):320–1.

**Reason for exclusion: not primary study**

Cockburn F, Barwell BE, Brenton DP, Chapple J, Clark B, Curzon G, et al. PHENYLKETONURIA DUE TO PHENYLALANINE-HYDROXYLASE DEFICIENCY - AN UNFOLDING STORY. British Medical Journal. 1993;306(6870):115–9.

**Reason for exclusion: method unsuitable**

Cockcroft A. Randomised controlled trials and changing public health practice. BMC Public Health. 2017;17(Suppl 1):409.

**Reason for exclusion: not primary study**

Cockrill K, Upadhyay UD, Turan J, Foster DG. The Stigma of Having an Abortion: Development of a Scale and Characteristics of Women Experiencing Abortion Stigma. Perspectives on Sexual and Reproductive Health. 2013;45(2):79–88.

**Reason for exclusion: method unsuitable**

Coffey M. Resistance and challenge: competing accounts in aftercare monitoring. Sociology of Health & Illness. 2011;33(5):748–60.

**Reason for exclusion: subject**

Coffey M. Time and its uses in accounts of conditional discharge in forensic psychiatry. Sociology of Health & Illness. 2013;35(8):1181–95.

**Reason for exclusion: method unsuitable**

Coggan C, Saunders C, Grenot D. Art and Safe Communities: the role of Big hART in the regeneration of an inner city housing estate. Health Promotion Journal of Australia. 2008;19(1):4–9.

**Reason for exclusion: subject matter**

Cohn J. Culturally appropriate storytelling to improve blood pressure. Annals of Internal Medicine. 2011;155(2):134–5.

**Reason for exclusion: not a personal story**

Coldwell SE, Milgrom P, Getz T, Ramsay DS. Amnestic and anxiolytic effects of alprazolam in oral surgery patients. Journal of Oral & Maxillofacial Surgery. 1997;55(10):1061–70.

**Reason for exclusion: subject matter**

Coleman R, Thorson E, Wilkins L. Testing the effect of framing and sourcing in health news stories. Journal of Health Communication. 2011;16(9):941–54.

**Reason for exclusion: not primary study**

Coleman-Fountain E. Uneasy encounters: Youth, social (dis)comfort and the autistic self. Social Science & Medicine. 2017;185:9–16.

**Reason for exclusion: method unsuitable**

Coley HL, Sadasivam RS, Williams JH, Volkman JE, Schoenberger YM, Kohler CL, et al. Crowdsourced Peer- Versus Expert-Written Smoking-Cessation Messages. American Journal of Preventive Medicine. 2013;45(5):543–50.

**Reason for exclusion: can't find study**

Collins A, McLachlan SA, Philip J. The CHOICE Project: A Randomised Exploratory Pilot Trial Testing the Acceptability and Preliminary Efficacy of a Public Health Communication Intervention. Journal of Pain and Symptom Management. 2018;56 (6):e61.

**Reason for exclusion: method unsuitable**

Collins A, McLachlan SA, Philip J. The choice project: Developing an educational public health intervention to inform community attitudes to palliative care. Palliative Medicine. 2018;32 (1 Supplement 1):56.

**Reason for exclusion: subject matter**

Collins AE. Vulnerability to coastal cholera ecology. Social Science & Medicine. 2003;57(8):1397–407.

**Reason for exclusion: off topic**

Collins C, Kohler C, Diclemente R, Wang MQ. Evaluation of the exposure effects of a theory-based street outreach HIV intervention on African-American drug users. Evaluation & Program Planning. 1999;22(3):279–93.

**Reason for exclusion: off topic**

Collins D, Villagran MA, Sparks L. Crossing borders, crossing cultures: Barriers to communication about cancer prevention and treatment along the US/Mexico border. Patient Education and Counseling. 2008;71(3):333–9.

**Reason for exclusion: off topic**

Collins L, Nerlich B. Examining User Comments for Deliberative Democracy: A Corpus-driven Analysis of the Climate Change Debate Online. Environmental Communication-a Journal of Nature and Culture. 2015;9(2):189–207.

**Reason for exclusion: method unsuitable**

Collins PA, Hayes MV. The role of urban municipal governments in reducing health inequities: A meta-narrative mapping analysis. International Journal for Equity in Health. 2010;9.

**Reason for exclusion: method unsuitable**

Colon-Ramos U, Monge-Rojas R, Stevenson TR, Burns H, Thurman S, Gittelsohn J, et al. How Do African-American Caregivers Navigate a Food Desert to Feed Their Children? A Photovoice Narrative. Journal of the Academy of Nutrition and Dietetics. 2018;118(11):2045–56.

**Reason for exclusion: no short term sacrifice, long term gain**

Comito J, Wolseth J, Morton LW. Tillage Practices, the Language of Blame, and Responsibility for Water Quality Impacts in Row Crop Agriculture. Human Ecology Review. 2012;19(2):146–58.

**Reason for exclusion: off topic**

Condrau F, Kirk RG. Negotiating hospital infections: the debate between ecological balance and eradication strategies in British hospitals, 1947-1969. Dynamis. 2011;31(2):385–405.

**Reason for exclusion: subject matter**

Conn C. Young African women must have empowering and receptive social environments for HIV prevention. AIDS Care - Psychological and Socio-Medical Aspects of AIDS/HIV. 2013;25(3):273–80.

**Reason for exclusion: off topic**

Connell P, Wolfe C, McKevitt C. Preventing stroke: a narrative review of community interventions for improving hypertension control in black adults. Health & Social Care in the Community. 2008;16(2):165–87.

**Reason for exclusion: method unsuitable**

Connors MM. STORIES OF PAIN AND THE PROBLEM OF AIDS-PREVENTION - INJECTION-DRUG WITHDRAWAL AND ITS EFFECT ON RISK BEHAVIOR. Medical Anthropology Quarterly. 1994;8(1):47–68.

**Reason for exclusion: can't find study**

Connors SC, Nyaude S, Challender A, Aagaard E, Velez C, Hakim J. Evaluating the Impact of the Medical Education Partnership Initiative at the University of Zimbabwe College of Health Sciences Using the Most Significant Change Technique. Academic Medicine. 2017;92(9):1264–8.

**Reason for exclusion: subject matter**

Conrad C, Millay B. Confronting free market romanticism: Health care reform in the least likely place. Journal of Applied Communication Research. 2001;29(2):153–70.

**Reason for exclusion: off topic**

Cook BR, Balayannis A. Co-Producing (a Fearful) Anthropocene. Geographical Research. 2015;53(3):270–9.

**Reason for exclusion: off topic**

Cook CL, Li YXJ, Newell SM, Cottrell CA, Neel R. The world is a scary place: Individual differences in belief in a dangerous world predict specific intergroup prejudices. Group Processes & Intergroup Relations. 2018;21(4):584–96.

**Reason for exclusion: off topic**

Cook JL, Sprague AE. Measuring Maternal Mortality in Canada: An Update on the Establishment of a Confidential Enquiry System for Preventing Maternal Deaths #savingmoms #savingbabies. Journal of Obstetrics and Gynaecology Canada. 2019;41(12):1768–71.

**Reason for exclusion: subject matter**

Cook PW. A great experiment in sex education--the Anaheim story. Journal of School Health. 1972;42(1):7–9.

**Reason for exclusion: date**

Cooley PD, Foley SJ, Magnussen CG. Increasing stair usage in a professional workplace: a test of the efficacy of positive and negative message prompts to change pedestrian choices. Health Promotion Journal of Australia. 2008;19(1):64–7.

**Reason for exclusion: subject matter**

Coope CM, Verlander NQ, Schneider A, Hopkins S, Welfare W, Johnson AP, et al. An evaluation of a toolkit for the early detection, management, and control of carbapenemase-producing Enterobacteriaceae: a survey of acute hospital trusts in England. Journal of Hospital Infection. 2018;99(4):381–9.

**Reason for exclusion: method unsuitable**

Cooper D, Mantell JE, Nywagi N, Cishe N, Austin-Evelyn K. Narrative Methods and Sociocultural Linguistic Approaches in Facilitating In-depth Understanding of HIV Disclosure in a Cohort of Women and Men in Cape Town, South Africa. Frontiers in Public Health. 2016;4.

**Reason for exclusion: method unsuitable**

Corby NH, Enguidanos SM, Kay LS. Development and use of role model stories in a community level HIV risk reduction intervention. Public Health Reports. 1996;111 Suppl 1:54–8.

**Reason for exclusion: not primary study**

Corby NH, Wolitski RJ. Condom use with main and other sex partners among high-risk women: intervention outcomes and correlates of reduced risk. Drugs & Society. 1996;9(1–2):75–96.

**Reason for exclusion: not primary study**

Cordova D, Alers-Rojas F, Lua FM, Bauermeister J, Nurenberg R, Ovadje L, et al. The Usability and Acceptability of an Adolescent mHealth HIV/STI and Drug Abuse Preventive Intervention in Primary Care. Behavioral Medicine. 2018;44(1):36–47.

**Reason for exclusion: off topic**

Cordova D, Bauermeister JA, Fessler K, Delva J, Nelson A, Nurenberg R, et al. A Community-Engaged Approach to Developing an mHealth HIV/STI and Drug Abuse Preventive Intervention for Primary Care: A Qualitative Study. JMIR MHealth and UHealth. 2015;3(4):e106.

**Reason for exclusion: off topic**

Cordova D, Lua FM, Munoz-Velazquez J, Street K, Bauermeister JA, Fessler K, et al. A multilevel mHealth drug abuse and STI/HIV preventive intervention for clinic settings in the United States: A feasibility and acceptability study. PLoS ONE [Electronic Resource]. 2019;14(8).

**Reason for exclusion: off topic**

Cordova D. The preliminary efficacy of a HIV preventive intervention app in an urban youth-centered community health clinic. Journal of Adolescent Health. 2018;62 (2 Supplement 1):S10–1.

**Reason for exclusion: conference abstract, have article**

Corneli AL, McKenna K, Headley J, Ahmed K, Odhiambo J, Skhosana J, et al. A descriptive analysis of perceptions of HIV risk and worry about acquiring HIV among FEM-PrEP participants who seroconverted in Bondo, Kenya, and Pretoria, South Africa. Journal of the International AIDS Society. 2014;17 (3 Supplement 2) (no pagination)(19152).

**Reason for exclusion: method unsuitable**

Cornelius-Schecter A, Safford MM, Jannat-Khah D, Frankel B, Cardillo C, Nicol C, et al. The impact of the patient activated learning system (pals) on knowledge acquisition, recall, and decision making about antihypertensive medication: A pilot rct. Journal of General Internal Medicine. 2018;33 (2 Supplement 1):366–7.

**Reason for exclusion: subject matter**

Cornish EK, Bergner EM, Griffith DM. “THEY HAVE SAID THAT I WAS SLIGHTLY DEPRESSED BUT THERE ARE CIRCUMSTANCES THAT BRING THAT ON”: HOW MIDDLE-AGED AND OLDER AFRICAN AMERICAN MEN DESCRIBE PERCEIVED STRESS AND DEPRESSION. Ethnicity & Disease. 2017;27(4):437–42.

**Reason for exclusion: method unsuitable**

Corrigan PW, Mittal D, Reaves CM, Haynes TF, Han XT, Morris S, et al. Mental health stigma and primary health care decisions. Psychiatry Research. 2014;218(1–2):35–8.

**Reason for exclusion: method unsuitable**

Corrigan PW, Powell KJ, Al-Khouja MA. Examining the impact of public service announcements on help seeking and stigma: Results of a randomized controlled trial. Journal of Nervous and Mental Disease. 2015;203(11):836–42.

**Reason for exclusion: subject matter**

Corsini GU. In memoriam Richard Heikkila. The MPTP story: irony creeps back in. Journal of Neural Transmission - Parkinsons Disease & Dementia Section. 1991;3(4):227–30.

**Reason for exclusion: subject matter**

Cosgriff M. Learning from Leisure: Developing Nature Connectedness in Outdoor Education. Asia-Pacific Journal of Health, Sport and Physical Education. 2011;2(1):51–65.

**Reason for exclusion: subject matter**

Costa GM, Gualda DM. Menopause knowledge and experience for a group of women. [Portuguese]. Revista da Escola de Enfermagem da U S P. 2008;42(1):81–9.

**Reason for exclusion: language**

Costa TL, Souza OM, Carneiro HA, Chiquito Netto C, Pegoraro-Krook MI, Dutka Jd.e C. Multimedia material about velopharynx and primary palatoplasty for orientation of caregivers of children with cleft lip and palate. Codas. 2016;28(1):10–6.

**Reason for exclusion: subject matter**

Costabile KA, Klein SB. Understanding and predicting social events: The effects of narrative construction on inference generation. Social Cognition. 2008;26(4):420–37.

**Reason for exclusion: method unsuitable**

Costi JM, Silva JBG, Min LS, More AOO, Hokama AL. Teaching acupuncture: The brazilian medical residency programme. Journal of Alternative and Complementary Medicine. 2013;19 (7):A43.

**Reason for exclusion: subject matter**

Costi JM, da Silva JB, Min LS, More AO, Hokama AL. Teaching acupuncture: the Brazilian Medical Residency Programme. Acupuncture in Medicine. 2012;30(4):350–3.

**Reason for exclusion: subject matter**

Costi JM, da Silva JBG, Min LS, More AOO, Hokama AL. Teaching acupuncture: the Brazilian Medical Residency Programme. Acupuncture in Medicine. 2012;30(4):350–3.

**Reason for exclusion: method unsuitable**

Cotugno JD, Ferguson M, Harden H, Colquist S, Stack AA, Zimmerman JI, et al. “I wish they could be in my shoes”: Patients’ insights into tertiary health care for type 2 diabetes mellitus. Patient Preference and Adherence. 2015;9:1647–55.

**Reason for exclusion: subject matter**

Counil E, Gauthier MJ, Blouin V, Grey M, Angiyou E, Kauki T, et al. Translational research to reduce trans-fat intakes in Northern Quebec (Nunavik) Inuit communities: a success story? International Journal of Circumpolar Health. 2012;71:18833.

**Reason for exclusion: subject matter**

Coups EJ, Chapman GB. Formation and use of covariation assessments in the real world. Applied Cognitive Psychology. 2002;16(1):51–71.

**Reason for exclusion: method unsuitable**

Courtial JP, Le Dreff G. Analysis of pregnant women’s narratives. [French]. Sante publique (Vandoeuvre-les-Nancy, France). 2004;16(1):105–21.

**Reason for exclusion: language**

Cowley L, McLaughlin J, Finch T, Clavering E, Burn J. Genetic testing and research in Lynch Syndrome - Is it a choice or a responsibility? Hereditary Cancer in Clinical Practice. 2011;1):7.

**Reason for exclusion: subject matter**

Cox TL, Oze C, Horton TW. Iron concretions within a highly altered unit of the Berlins Porphyry, New Zealand: an abiotic or biotic story? Mineralogy and Petrology. 2017;111(2):173–81.

**Reason for exclusion: method unsuitable**

Coxhead L, Rhodes T. Accounting for risk and responsibility associated with smoking among mothers of children with respiratory illness. Sociology of Health & Illness. 2006;28(1):98–121.

**Reason for exclusion: method unsuitable**

Coxon K, Sandall J, Fulop NJ. To what extent are women free to choose where to give birth? How discourses of risk, blame and responsibility influence birth place decisions. Health, Risk and Society. 2014;16(1):51–67.

**Reason for exclusion: subject matter**

Cramer P. Defense mechanisms and physiological reactivity to stress. Journal of Personality. 2003;71(2):221–44.

**Reason for exclusion: method unsuitable**

Crane D, Ball HL. A qualitative study in parental perceptions and understanding of SIDS-reduction guidance in a UK bi-cultural urban community. BMC Pediatrics. 2016;16.

**Reason for exclusion: method unsuitable**

Crate SA. “Eating hay”: The ecology, economy and culture of viliui sakha smallholders of northeastern siberia. Human Ecology. 2008;36(2):161–74.

**Reason for exclusion: method unsuitable**

Craven I. Hitchcock and small-gauge: Shaping the amateur fiction film. Journal of Media Practice. 2012;13(1):19–44.

**Reason for exclusion: off topic**

Cronin V, Guthrie P. Community-led resettlement: From a flood-affected slum to a new society in Pune, India. Environmental Hazards-Human and Policy Dimensions. 2011;10(3–4):310–26.

**Reason for exclusion: method unsuitable**

Crook T. Norms, Forms and Beds: Spatializing Sleep in Victorian Britain. Body & Society. 2008;14(4):15–35.

**Reason for exclusion: method unsuitable**

Cropper DP, Harb NH, Said PA, Lemke JH, Shammas NW. Implementation of a patient safety program at a tertiary health system: A longitudinal analysis of interventions and serious safety events. Journal of Healthcare Risk Management. 2018;37(4):17–24.

**Reason for exclusion: subject matter**

Crossland N, Thomson G, Hall Moran V. Evaluation of Best Beginnings resources. Maternal and Child Nutrition Conference. 2017;14(Supplement 2).

**Reason for exclusion: subject matter**

Croteau SE, Cutter S, Hernandez G, Wicklund B, Dreyer Gillette ML, Haugstad K, et al. Awareness, Care and Treatment In Obesity maNagement to inform Haemophilia Obesity Patient Empowerment (ACTION-TO-HOPE): Results of a survey of US patients with haemophilia and obesity (PwHO) and their partners and caregivers. Haemophilia. 2020;26(S1):3–19.

**Reason for exclusion: no short term sacrifice, long term gain**

Cruikshank J. Glaciers and climate change: Perspectives from oral tradition. Arctic. 2001;54(4):377–93.

**Reason for exclusion: not primary study**

Crumbaugh J. Spectacle as spectralization, untimely timelessness: Marcelino pan y vino and mid-1950s’ Francoism. Journal of Spanish Cultural Studies. 2014;15(3):337–50.

**Reason for exclusion: off topic**

Cruz A. Leprosy as a multilayered biosocial phenomenon: The comparison of institutional responses and illness narratives of an endemic disease in Brazil and an imported disease in Portugal. Clinics in Dermatology. 2016;34(1):16–23.

**Reason for exclusion: subject matter**

Cueva M, Dignan M, Lanier A, Kuhnley R. Qualitative evaluation of a colorectal cancer education CD-ROM for Community Health Aides/practitioners in Alaska. Journal of Cancer Education. 2014;29(4):613–8.

**Reason for exclusion: method unsuitable**

Cueva M, Hicks T, Kuhnley R, Cueva K. A Wellness Course for Community Health Workers in Alaska: “wellness lives in the heart of the community.” International Journal of Circumpolar Health. 2012;71(1):19125.

**Reason for exclusion: off topic**

Cueva M, Kuhnley R, Cueva K. Enhancing Cancer Education through the Arts: Building Connections with Alaska Native People, Cultures and Communities. International Journal of Lifelong Education. 2012;31(3):341–57.

**Reason for exclusion: no short term sacrifice, long term gain**

Cueva M, Kuhnley R, Lanier A, Dignan M, Revels L, Schoenberg NE, et al. Promoting Culturally Respectful Cancer Education Through Digital Storytelling. International Journal of Indigenous Health. 2016;11(1):34–49.

**Reason for exclusion: no short term sacrifice, long term gain**

Cueva M, Kuhnley R, Lanier A, Dignan M. Using theater to promote cancer education in Alaska. Journal of Cancer Education. 2005;20(1):45–8.

**Reason for exclusion: no short term sacrifice, long term gain**

Cueva M, Kuhnley R, Lanier AP, Dignan M. Story: The Heartbeat of Learning Cancer Education for Alaska Native Community Healthcare Providers. Convergence. 2006;39(4):81–9.

**Reason for exclusion: subject matter**

Cueva M, Kuhnley R, Revels L, Schoenberg NE, Dignan M %J I journal of circumpolar health. Digital storytelling: a tool for health promotion and cancer awareness in rural Alaskan communities. 2015;74(1):28781.

**Reason for exclusion: no short term sacrifice, long term gain**

Cueva M, Kuhnley R, Revels L, Schoenberg NE, Dignan M. Digital storytelling: a tool for health promotion and cancer awareness in rural Alaskan communities. International Journal of Circumpolar Health. 2015;74(1):28781.

**Reason for exclusion: no short term sacrifice, long term gain**

Cueva M, Kuhnley R, Revels L, Schoenberg NE, Lanier A, Dignan M. Engaging Elements of Cancer-Related Digital Stories in Alaska. Journal of Cancer Education. 2016;31(3):500–5.

**Reason for exclusion: no short term sacrifice, long term gain**

Cueva M, Kuhnley R, Slatton J, Dignan M, Underwood E, Landis K. Telenovela: an innovative colorectal cancer screening health messaging tool. International Journal of Circumpolar Health. 2013;72:21301.

**Reason for exclusion: no short term sacrifice, long term gain**

Cueva M. A Living Spiral of Understanding: Community-Based Adult Education. New Directions for Adult and Continuing Education. 2010;(125):79–90.

**Reason for exclusion: subject matter**

Cui YL, Wu RL. Molecular dynamics investigations of membrane-bound CYP2C19 polymorphisms reveal distinct mechanisms for peripheral variants by long-range effects on the enzymatic activity. Molecular Biosystems. 2017;13(6):1070–9.

**Reason for exclusion: off topic**

Cullen L, Grenfell P, Rodger A, Orkin C, Mandal S, Rhodes T. “Just another vial”: A qualitative study to explore the acceptability and feasibility of routine blood-borne virus testing in an emergency department setting in the UK. BMJ Open. 2019;9 (4) (no pagination)(24085).

**Reason for exclusion: subject matter**

Cunningham JMZ, Fairburn S. Climate Anticipation: working towards a design proposal for urban resilience and care. Design Journal. 2019;22:1697–714.

**Reason for exclusion: method unsuitable**

Curci JA, Beckman JA. Positron Emission Tomography Fluorine-18-Labeled 2-Deoxy-2-Fluoro-d-Glucose Tells a Complicated Story in the Aortic Aneurysm Wall. Circulation: Cardiovascular Imaging. 2016;9 (11) (no pagination)(e005689).

**Reason for exclusion: subject matter**

Curtis A, De Lacy T. Landcare in Australia: Does it make a difference? Journal of Environmental Management. 1996;46(2):119–37.

**Reason for exclusion: subject matter**

Curtis A, DeLacy T. Landcare in Australia: Does it make a difference? Journal of Environmental Management. 1996;46(2):119–37.

**Reason for exclusion: off topic**

Curtis P, Stapleton H, James A. Intergenerational relations and the family food environment in families with a child with obesity. Annals of Human Biology. 2011;38(4):429–37.

**Reason for exclusion: subject matter**

Cutajar L, Cyna AM. Antenatal education for childbirth-epidural analgesia. Midwifery. 2018;64:48–52.

**Reason for exclusion: subject matter**

Cutting JE. Event segmentation and seven types of narrative discontinuity in popular movies. Acta Psychologica. 2014;149:69–77.

**Reason for exclusion: method unsuitable**

Da Silva Amaro De Oliveira Fabiao JA, Da Cruz Leitao MN. Human female sexuality-young people’s conceptions. Journal of Sexual Medicine. 2013;5):321.

**Reason for exclusion: subject matter**

Dahl SL, Sandberg S. Female Cannabis Users and New Masculinities: The Gendering of Cannabis Use. Sociology-the Journal of the British Sociological Association. 2015;49(4):696–711.

**Reason for exclusion: method unsuitable**

Dahlberg KM, Waern M, Runeson B. Mental health literacy and attitudes in a Swedish community sample - Investigating the role of personal experience of mental health care. BMC Public Health. 2008;8.

**Reason for exclusion: method unsuitable**

Dahlstrom MF, Rosenthal S. Third-Person Perception of Science Narratives: The Case of Climate Change Denial. Science Communication. 2018;40(3):340–65.

**Reason for exclusion: off topic**

Dai Z. What have we missed?: The knowledge of and access to the HPV vaccine and sex education in China. Health Communication. 2020;35(1):96–8.

**Reason for exclusion: off topic**

Daigle M. “This Is How We Travel”: Sex, Love, Intimacy and the Border. Geopolitics. 2019;

**Reason for exclusion: subject matter**

Dailey R, Schwartz KL, Binienda J, Moorman J, Neale AV. Challenges in making therapeutic lifestyle changes among hypercholesterolemic African-American patients and their physicians. Journal of the National Medical Association. 2006;98(12):1895–903.

**Reason for exclusion: method unsuitable**

Dal Cin S, Gibson B, Zanna MP, Shumate R, Fong GT. Smoking in movies, implicit associations of smoking with the self, and intentions to smoke. Psychological Science. 2007;18(7):559–63.

**Reason for exclusion: off topic**

Danet Danet A, Jimenez Cardoso PM, Perez Villares JM. Emotional paths of professional experiences in transplant coordinators. Nefrologia. 2020;

**Reason for exclusion: subject matter**

Daniel DB, Poole DA. Learning for Life: An Ecological Approach to Pedagogical Research. Perspectives on Psychological Science. 2009;4(1):91–6.

**Reason for exclusion: off topic**

Daniels NA, Juarbe T, Rangel-Lugo M, Moreno-John G, Perez-Stable EJ. Focus group interview’s on racial and ethnic attitudes regarding adult vaccinations. Journal of the National Medical Association. 2004;96(11):1455–61.

**Reason for exclusion: method unsuitable**

Daniels S, Endfield GH. Narratives of climate change: introduction. Journal of Historical Geography. 2009;35(2):215–22.

**Reason for exclusion: not primary study**

Darbyshire P, Collins C, McDonald HM, Hiller JE. Taking antenatal group B Streptococcus seriously: women’s experiences of screening and perceptions of risk. Birth. 2003;30(2):116–23.

**Reason for exclusion: method unsuitable**

Darling CA, Rehm M, Coccia C, Cui M. Adolescent Eating Behavior: The Role of Indulgent Parenting. Families in Society-the Journal of Contemporary Social Services. 2015;96(4):257–67.

**Reason for exclusion: method unsuitable**

Datta J, Reid D, Hughes G, Mercer CH, Wayal S, Weatherburn P. Awareness of and attitudes to sexually transmissible infections among gay men and other men who have sex with men in England: a qualitative study. Sexual Health. 2019;16(1):18–24.

**Reason for exclusion: m**

Davey DD. Cervical cytology classification and the Bethesda System. Cancer Journal. 2003;9(5):327–34.

**Reason for exclusion: subject matter**

David M, Schonborn S. Bottom-Up Energy Transition Narratives: Linking the Global with the Local? A Comparison of Three German Renewable Co-Ops. Sustainability. 2018;10(4).

**Reason for exclusion: method unsuitable**

Davidson D, Vanegas SB. The role of emotion on the recall of central and peripheral information from script-based text. Cognition & Emotion. 2015;29(1):76–94.

**Reason for exclusion: method unsuitable**

Davidson T, Moreland A, Bunnell BE, Winkelmann J, Hamblen JL, Ruggiero KJ. Reducing stigma in mental health through digital storytelling. In: Deconstructing Stigma in Mental Health. 2018. p. 169–83.

**Reason for exclusion: no short term sacrifice, long term gain**

Davies J, Bukulatjpi S, Sharma S, Caldwell L, Johnston V, Davis JS. Development of a Culturally Appropriate Bilingual Electronic App About Hepatitis B for Indigenous Australians: Towards Shared Understandings. JMIR Research Protocols. 2015;4(2):e70.

**Reason for exclusion: method unsuitable**

Davies J, Bukulatjpi S, Sharma S, Davis J, Johnston V. “Only your blood can tell the story” - a qualitative research study using semi-structured interviews to explore the hepatitis B related knowledge, perceptions and experiences of remote dwelling Indigenous Australians and their health care providers in northern Australia. BMC Public Health. 2014;14.

**Reason for exclusion: off topic**

Davies J, Bukulatjpi S, Sharma S, Davis J, Johnston V. “Only your blood can tell the story”--a qualitative research study using semi-structured interviews to explore the hepatitis B related knowledge, perceptions and experiences of remote dwelling Indigenous Australians and their health care providers in northern Australia. BMC Public Health. 2014;14:1233.

**Reason for exclusion: off topic**

Davies JE, Spear D, Ziervogel G, Hegga S, Ndapewa Angula M, Kunamwene I, et al. Avenues of understanding: mapping the intersecting barriers to adaptation in Namibia. Climate and Development. 2019;

**Reason for exclusion: off topic**

Davis AC, Wright CJC, Temple-Smith MJ, Hellard ME, Lim MSC. Assessing the usability of SCOPE: A health education website developed to meet young people’s information needs about web-based pornography and sharing of sexually explicit imagery. Journal of Medical Internet Research. 2019;21(8).

**Reason for exclusion: subject matter**

Davis C, Darby K, Moore M, Cadet T, Brown G. Breast care screening for underserved African American women: Community-based participatory approach. Journal of Psychosocial Oncology. 2017;35(1):90–105.

**Reason for exclusion: no short term sacrifice, long term gain**

Davis CS %J QI. Sylvia’s story: Narrative, storytelling, and power in a children’s community mental health system of care. 2006;12(6):1220–43.

**Reason for exclusion: not primary study**

Davis LF, Ramirez-Andreotta MD, McLain JET, Kilungo A, Abrell L, Buxner S. Increasing Environmental Health Literacy through Contextual Learning in Communities at Risk. International Journal of Environmental Research and Public Health. 2018;15(10).

**Reason for exclusion: method unsuitable**

Davis M, Rhodes T. Beyond prevention? Injecting drug user narratives about hepatitis C. International Journal of Drug Policy. 2004;15(2):123–31.

**Reason for exclusion: too clinical**

Davis O. Barbershop cuisine: African American foodways and narratives of health in the black barbershop. International Journal of Men’s Health. 2013;12(2):138–49.

**Reason for exclusion: too unspecific**

Davis PB, Solomon J, Gorenflo G. Driving quality improvement in local public health practice. Journal of Public Health Management & Practice. 2010;16(1):67–71.

**Reason for exclusion: off topic**

Davlin SL, Lapiz SM, Miranda ME, Murray KO. Knowledge, attitudes, and practices regarding rabies in Filipinos following implementation of the Bohol Rabies Prevention and Elimination Programme. Epidemiology and Infection. 2014;142(7):1476–85.

**Reason for exclusion: subject**

Dawson MT, Gifford SM. Social change, migration and sexual health: Chilean women in Chile and Australia. Women and Health. 2003;38(4):39–56.

**Reason for exclusion: subject matter**

Day V %J OTOJ of I in N. Promoting health literacy through storytelling. 2009;14(3):6.

**Reason for exclusion: not primary study**

De Schacht C, Lucas C, Mboa C, Gill M, Macasse E, Dimande SA, et al. Access to HIV prevention and care for HIV-exposed and HIV-infected children: a qualitative study in rural and urban Mozambique. BMC Public Health. 2014;14:1240.

**Reason for exclusion: method unsuitable**

De Vecchi N, Kenny A, Dickson‐Swift V, Kidd S %J IJ of MHN. How digital storytelling is used in mental health: A scoping review. 2016;25(3):183–93.

**Reason for exclusion: not primary study**

DeBruyn L, Fullerton L, Satterfield D, Frank M. Integrating Culture and History to Promote Health and Help Prevent Type 2 Diabetes in American Indian/Alaska Native Communities: Traditional Foods Have Become a Way to Talk About Health. Preventing Chronic Disease. 2020;17:E12.

**Reason for exclusion: no short term sacrifice, long term gain**

DeHart WB, Kaplan BA, Pope DA, Mellis AM, Bickel WK. The Experimental Tobacco Marketplace: Narrative Influence on Electronic Cigarette Substitution. Experimental and Clinical Psychopharmacology. 2019;27(2):115–24.

**Reason for exclusion: off topic**

DeTora LM. What is safety?: Miracles, benefit-risk assessments, and the “right to try.” International Journal of Clinical Practice. 2017;71 (7) (no pagination)(e12966).

**Reason for exclusion: subject matter**

Deacon Z, Pendley J, Hinson WR, Hinson JD. Chokka-Chaffa’ Kilimpi’, Chikashshiyaakni’ Kilimpi’: Strong Family, Strong Nation. American Indian and Alaska Native Mental Health Research: The Journal of the National Center. 2011;18(2):41–63.

**Reason for exclusion: off topic**

Dearing S. On Physical and Spiritual Recovery: Reconsidering the Role of Patients in Early American Restitution Narratives. The Journal of medical humanities. 2019;22.

**Reason for exclusion: subject matter**

Debono J, Fry B. Differential coagulotoxic effects of Asian pitviper snake venoms: Evolutionary, pathophysiology and biodiscovery implications. Research and Practice in Thrombosis and Haemostasis. 2017;1 (Supplement 1):390.

**Reason for exclusion: subject matter**

Degregori MCA. Docudramas and public health: Their role in access to HIV/SIDA prevention and treatment programs in southern Mozambique. Tropical Medicine and International Health. 2009;2):103.

**Reason for exclusion: off topic**

Del Prete E, Unti E, Frosini D, Turcano P, Bonuccelli U, Ceravolo R. Theory of Mind in early drug-naive Parkinson’s disease patients: Role of dopaminergic therapy. Neurological Sciences. 2015;36 (2 Supplement 1):S204.

**Reason for exclusion: subject matter**

Delaney C, Martin-Biggers JT, Povis-Alleman G, Hongu N, Worobey J, Byrd-Bredbenner C. Nudges: Fun, motivational messages to encourage and reassure parents in the homestyles randomized controlled trial. FASEB Journal Conference: Experimental Biology. 2016;30(Meeting Abstracts).

**Reason for exclusion: subject matter**

Delange F, Burgi H, Chen ZP, Dunn JT. World status of monitoring iodine deficiency disorders control programs. Thyroid. 2002;12(10):915–24.

**Reason for exclusion: subject matter**

Delange F, Burgi H, Chen ZP, Dunn JT. World status of monitoring of iodine deficiency disorders control programs. Thyroid. 2002;12(10):915–24.

**Reason for exclusion: method unsuitable**

Delbridge R, Wilson A, Palermo C. Measuring the Impact of a Community of Practice in Aboriginal Health. Studies in Continuing Education. 2018;40(1):62–75.

**Reason for exclusion: off topic**

Delina LL. A rural energy collaboratory: co-production in Thailand’s community energy experiments. Journal of Environmental Studies and Sciences. 2020;10(1):83–90.

**Reason for exclusion: subject matter**

Delmotte S, Couderc V, Mouret JC, Lopez-Ridaura S, Barbier JM, Hossard L. From stakeholders narratives to modelling plausible future agricultural systems. Integrated assessment of scenarios for Camargue, Southern France. European Journal of Agronomy. 2017;82:292–307.

**Reason for exclusion: subject**

Delormier T, Marquis K. Building Healthy Community Relationships Through Food Security and Food Sovereignty. Current Developments in Nutrition. 2019;3:25–31.

**Reason for exclusion: no short term sacrifice, long term gain**

Demir S, Sadi Aykan F, Oztuna D. Latent tuberculosis treatment results in patients that taken TNF-alpha blockers at Ankara Numune training and research hospital chest diseases clinic for last 8 years (2006-2013). [Turkish]. Tuberkuloz ve Toraks. 2014;62(4):286–90.

**Reason for exclusion: off topic**

Deml MJ, Notter J, Kliem P, Buhl A, Huber BM, Pfeiffer C, et al. “We treat humans, not herds!”: A qualitative study of complementary and alternative medicine (CAM) providers’ individualized approaches to vaccination in Switzerland. Social Science and Medicine. 2019;240 (no pagination).

**Reason for exclusion: off topic**

Dempsey AF, O’Leary ST. Human Papillomavirus Vaccination: Narrative Review of Studies on How Providers’ Vaccine Communication Affects Attitudes and Uptake. Academic pediatrics. 2018;18(2):S23–7.

**Reason for exclusion: off topic**

Denby GM, Von Thun L, Terpak K. Piping study at Upriver Dam, Spokane, Washington. In: Association of State Dam Safety Officials - Dam Safety 2010 Proceedings. 2010.

**Reason for exclusion: off topic**

Denham CR, Angood P, Berwick D, Binder L, Clancy CM, Corrigan JM, et al. Chasing zero: can reality meet the rhetoric? Journal of patient safety. 2009;5(4):216–22.

**Reason for exclusion: off topic**

Denis F, Millot I, Abello N, Carpentier M, Peteuil A, Soudry-Faure A. Study protocol: a cluster randomized controlled trial to assess the effectiveness of a therapeutic educational program in oral health for persons with schizophrenia. International Journal of Mental Health Systems. 2016;10.

**Reason for exclusion: method unsuitable**

Dennis SF, Gaulocher S, Carpiano RM, Brown D. Participatory photo mapping (PPM): Exploring an integrated method for health and place research with young people. Health & Place. 2009;15(2):466–73.

**Reason for exclusion: subject matter**

Denniss RJ, Davison A. Self and world in lay interpretations of climate change. International Journal of Climate Change Strategies and Management. 2015;7(2):140–53.

**Reason for exclusion: not primary study**

Denscombe M. Critical incidents and the perception of health risks: the experiences of young people in relation to their use of alcohol and tobacco. Health Risk & Society. 2001;3(3):293–306.

**Reason for exclusion: method unsuitable**

Denzongpa K, Nichols T. We Can’t Step Back: Women Specially…A Narrative Case Study on Resilience, Independence, and Leadership of a Bhutanese Refugee Woman. Affilia - Journal of Women and Social Work. 2020;35(1):129–45.

**Reason for exclusion: off topic**

Depaoli S, van de Schoot R. Improving Transparency and Replication in Bayesian Statistics: The WAMBS-Checklist. Psychological Methods. 2017;22(2):240–61.

**Reason for exclusion: method unsuitable**

Desaive C. A CRITICAL-REVIEW OF A PERSONAL SERIES OF 1000 GASTROPLASTIES. International Journal of Obesity. 1995;19:S56–60.

**Reason for exclusion: method unsuitable**

Desmoulins C, Lionquy G, Constans P, Caby I, Faucher JN. Bacillus anthracis: A dark story [3]. [French]. Medecine et Maladies Infectieuses. 1996;26(3):351–3.

**Reason for exclusion: off topic**

Dessai S, Bhave A, Birch C, Conway D, Garcia-Carreras L, Gosling JP, et al. Building narratives to characterise uncertainty in regional climate change through expert elicitation. Environmental Research Letters. 2018;13(7).

**Reason for exclusion: not primary study**

Dessein AF, Fontaine M, Andresen BS, Gregersen N, Brivet M, Rabier D, et al. A novel mutation of the ACADM gene (c.145C>G) associated with the common c.985A>G mutation on the other ACADM allele causes mild MCAD deficiency: A case report. Orphanet Journal of Rare Diseases. 2010;5 (1) (no pagination)(26).

**Reason for exclusion: subject matter**

Devine CM, Connors MM, Sobal J, Bisogni CA. Sandwiching it in: Spillover of work onto food choices and family roles in low- and moderate-income urban households. Social Science and Medicine. 2003;56(3):617–30.

**Reason for exclusion: not primary study**

Devora PV, Beevers S, Kiselica AM, Benge JF. Normative Data for Derived Measures and Discrepancy Scores for the Uniform Data Set 3.0 Neuropsychological Battery. Archives of clinical neuropsychology : the official journal of the National Academy of Neuropsychologists. 2019;35(1):75–89.

**Reason for exclusion: subject matter**

Devoy RJN, Delaney C, Carter RWG, Jennings SC. Coastal stratigraphies as indicators of environmental changes upon European Atlantic coasts in the Late Holocene. Journal of Coastal Research. 1996;12(3):564–88.

**Reason for exclusion: off topic**

Dhaenens L, De Roo C, Debunne H, Tilleman K, Vandekerckhove F, Gerris J, et al. How indicative are the standard investigations in recurrent pregnancy loss? Human Reproduction. 2014;1):i128.

**Reason for exclusion: not primary study**

Dhillon G. Education through recreation. Indian Journal of Social Work. 1983;44(1):63–8.

**Reason for exclusion: pre-1990**

Di Battista EM, Bracken RM, Stephens JW, Rice S, Williams SP, Thomas M, et al. Cardiovascular risk assessments at occupational health services: employee experiences. Occupational medicine (Oxford, England). 2019;69(2):106–12.

**Reason for exclusion: subject matter**

DiRienzo D. Research Gaps in the Use of Dairy Ingredients in Food Aid Products. Food and Nutrition Bulletin. 2016;37:S51–7.

**Reason for exclusion: method unsuitable**

Dianzani C, Pizzuti A, Gaspardini F, Bernardini L, Rizzo B, Degener AM. Ulerythema ophryogenes, a rare and often misdiagnosed syndrome: analysis of an idiopathic case. International Journal of Immunopathology & Pharmacology. 2011;24(2):523–7.

**Reason for exclusion: subject matter**

Diaz VA, Mainous AG, Pope C. Cultural conflicts in the weight loss experience of overweight Latinos. International Journal of Obesity. 2007;31(2):328–33.

**Reason for exclusion: method unsuitable**

Dickerson SS, Klingman KJ, Jungquist CR. Common meanings of good and bad sleep in a healthy population sample. Sleep health. 2016;2(3):253–9.

**Reason for exclusion: off topic**

Diggs RC, Clark KD. It’s a struggle but worth it: Identifying and managing identities in an interracial friendship. Communication Quarterly. 2002;50(3–4):368–90.

**Reason for exclusion: off topic**

Dijkstra K. The Role of Modality on Correct Recognition and Misinformation in Younger and Older Adults. Journal of Psychophysiology. 2013;1):15–6.

**Reason for exclusion: subject matter**

Dijkzeul D, Wakenge CI. Doing good, but looking bad? Local perceptions of two humanitarian organisations in eastern Democratic Republic of the Congo. Disasters. 2010;34(4):1139–70.

**Reason for exclusion: off topic**

Dillard AJ, Fagerlin A, Cin SD, Zikmund-Fisher BJ, Ubel PA. Narratives that address affective forecasting errors reduce perceived barriers to colorectal cancer screening. Social Science and Medicine. 2010;71(1):45–52.

**Reason for exclusion: no short term sacrifice, long term gain**

Dillard AJ, Fagerlin A, Dal Cin S, Zikmund-Fisher BJ, Ubel PA. Narratives that address affective forecasting errors reduce perceived barriers to colorectal cancer screening. Social Science & Medicine. 2010;71(1):45–52.

**Reason for exclusion: method unsuitable**

Dillard AJ, Hisler G. Enhancing the effects of a narrative message through experiential information processing: An experimental study. Psychology & Health. 2015;30(7):803–20.

**Reason for exclusion: method unsuitable**

Dillard AY, Carpenter DA, Mau EF, Kekauoha BP. Case report from the field: integrating Hawaiian and Western healing arts in Papakolea. Hawai’i Journal of Medicine & Public Health : A Journal of Asia Pacific Medicine & Public Health. 2014;73(12 Suppl 3):26–8.

**Reason for exclusion: subject matter**

Dillon R, Marini S, Miller SF. Plus-50 Success Story. Community College Journal. 2009;80(2):44–7.

**Reason for exclusion: off topic**

Dingle GA, Brander C, Ballantyne J, Baker FA. “To be heard”: The social and mental health benefits of choir singing for disadvantaged adults. Psychology of Music. 2013;41(4):405–21.

**Reason for exclusion: method unsuitable**

Dixon H, Scully M, Wakefield M, Kelly B, Pettigrew S, Chapman K, et al. The impact of unhealthy food sponsorship vs. pro-health sponsorship models on young adults’ food preferences: a randomised controlled trial. BMC Public Health. 2018;18.

**Reason for exclusion: method unsuitable**

Djellouli N, Quevedo-Gomez MC. Challenges to successful implementation of HIV and AIDS-related health policies in Cartagena, Colombia. Social Science & Medicine. 2015;133:36–44.

**Reason for exclusion: off topic**

Dobson MJ. Bitter-sweet solutions for malaria: exploring natural remedies from the past. Parassitologia. 1998;40(1–2):69–81.

**Reason for exclusion: subject matter**

Dobson WL, Wong PTP. Women living with HIV: The role of meaning and spirituality. In: Existential and spiritual issues in death attitudes. 2008. p. 173–207.

**Reason for exclusion: Book not a journal paper of a primary study**

Dohan D, Garrett SB, Rendle KA, Halley M, Abramson C. The Importance Of Integrating Narrative Into Health Care Decision Making. Health Affairs. 2016;35(4):720–5.

**Reason for exclusion: not primary study**

Dohany L, Fuller S, Niemchak T, Swope B, Buis J. Is hexosaminidase A enzyme testing still needed in the genomics age? American Journal of Obstetrics and Gynecology. 2016;1):S401.

**Reason for exclusion: subject matter**

Doi T, Shimada H, Park H, Makizako H, Tsutsumimoto K, Uemura K, et al. Cognitive function and falling among older adults with mild cognitive impairment and slow gait. Geriatrics & gerontology international. 2015;15(8):1073–8.

**Reason for exclusion: subject matter**

Doll KM, Hempstead B, Truitt AR. Seeking Black Women’s Voices in Endometrial Cancer Research via Deliberate Community Engagement. Progress in community health partnerships : research, education, and action. 2019;13(3):253–64.

**Reason for exclusion: no short term sacrifice, long term gain**

Domenech-Dorca G, Giami A. Sexuality, pleasure and HIV infection: Scripts of HIV contamination among men who have sex with men (MSM) in France. Sexologies. 2019;

**Reason for exclusion: can't find study**

Domenech-Dorca G, Giami A. Sexuality, pleasure and HIV infection: Scripts of HIV contamination among men who have sex with men (MSM) in France. Sexologies. 2019;28(3):104–13.

**Reason for exclusion: off topic**

Domino SE, Bodurtha J, Nagel JD. Interdisciplinary research career development: Building interdisciplinary research careers in women’s health program best practices. Journal of Women’s Health. 2011;20(11):1587–601.

**Reason for exclusion: subject matter**

Domizio M, Ambrosini D, Curadelli O. Performance of tuned mass damper against structural collapse due to near fault earthquakes. Journal of Sound and Vibration. 2015;336:32–45.

**Reason for exclusion: method unsuitable**

Dorant E, Krieger T. Contextual exploration of a new family caregiver support concept for geriatric settings using a participatory health research strategy. International Journal of Environmental Research and Public Health. 2017;14 (12) (no pagination)(1467).

**Reason for exclusion: off topic**

Doring N. Sex-related online training for professionals: Weblogs. Zeitschrift fur Sexualforschung. 2019;32(2):100–6.

**Reason for exclusion: subject matter**

Dorji S, Vernes K, Rajaratnam R. Habitat Correlates of the Red Panda in the Temperate Forests of Bhutan. PLoS ONE [Electronic Resource]. 2011;6(10).

**Reason for exclusion: off topic**

Dornig K, Koniak-Griffin D, Lesser J, Gonzalez-Figueroa E, Luna MC, Anderson NLR, et al. “You Gotta Start Thinking Like a Parent”: Hopes, Dreams, and Concerns of Ethnic Minority Adolescent Parents. Families in Society-the Journal of Contemporary Social Services. 2009;90(1):51–60.

**Reason for exclusion: method unsuitable**

Dos Santos Silva Junior H, Nobre LN, De Fatima Do Nascimento M, Ferreira PAA, Machado IML, Chaves AC, et al. Puppet theater as a pedagogical strategy in the food and nutrition education of children in the early childhood education. Diabetology and Metabolic Syndrome Conference: 22nd Brazilian Diabetes Society Congress Brazil. 2019;11(Supplement 1).

**Reason for exclusion: no short term sacrifice, long term gain**

Dossa P. Narrative mediation of conventional and new “mental health” paradigms: Reading the stories of immigrant Iranian women. Medical Anthropology Quarterly. 2002;16(3):341–59.

**Reason for exclusion: no short term sacrifice, long term gain**

Doucet S, Andrews C, Godden-Webster AL, Lauckner H, Nasser S. The Dalhousie Health Mentors Program: introducing students to collaborative patient/client-centered practice. Journal of Interprofessional Care. 2012;26(4):336–8.

**Reason for exclusion: off topic**

Doucette J, Muller AR. Exporting the Saemaul spirit: South Korea’s Knowledge Sharing Program and the “rendering technical” of Korean development. Geoforum. 2016;75:29–39.

**Reason for exclusion: method unsuitable**

Douglas ML, McGhan SL, Tougas D, Fenton N, Sarin C, Latycheva O, et al. Asthma education program for First Nations children: an exemplar of the knowledge-to-action framework. Canadian Respiratory Journal. 2013;20(4):295–300.

**Reason for exclusion: off topic**

Douglas V, Chan HM, Wesche S, Dickson C, Kassi N, Netro L, et al. Reconciling Traditional Knowledge, Food Security, and Climate Change: Experience From Old Crow, YT, Canada. Progress in Community Health Partnerships-Research Education and Action. 2014;8(1):21–7.

**Reason for exclusion: method unsuitable**

Downing M, Knight KR, Vernon KA, Seigel S, Ajaniku I, Acosta PS, et al. This is my story: a descriptive analysis of a peer education HIV/STD risk reduction program for women living in housing developments. AIDS Education & Prevention. 1999;11(3):243–61.

**Reason for exclusion: method unsuitable**

Downs JS. Prescriptive scientific narratives for communicating usable science. Proceedings of the National Academy of Sciences of the United States of America. 2014;111 Suppl 4:13627–33.

**Reason for exclusion: off topic**

Doyal L, Anderson J, Paparini S. “You are not yourself”: Exploring masculinities among heterosexual African men living with HIV in London. Social Science & Medicine. 2009;68(10):1901–7.

**Reason for exclusion: method unsuitable**

Drazkiewicz-Grodzicka E. “State Bureaucrats’ and ”Those NGO People’: Promoting the idea of civil society, hindering the state. Critique of Anthropology. 2016;36(4):341–62.

**Reason for exclusion: off topic**

Drew EM, Schoenberg NE. Deconstructing Fatalism: Ethnographic Perspectives on Women’s Decision Making about Cancer Prevention and Treatment. Medical Anthropology Quarterly. 2011;25(2):164–82.

**Reason for exclusion: method unsuitable**

Drew SE, Duncan RE, Sawyer SM %J Q health research. Visual storytelling: A beneficial but challenging method for health research with young people. 2010;20(12):1677–88.

**Reason for exclusion: not primary study**

Driskell JE, Salas E, Driskell T. Social indicators of deception. Human factors. 2012;54(4):577–88.

**Reason for exclusion: subject matter**

Drost R. Memory and Decision Making: Determining Action when the Sirens Sound. Weather Climate and Society. 2013;5(1):43–54.

**Reason for exclusion: off topic**

Drozdowicz L. Chicken fried. Pharos of Alpha Omega Alpha Honor Medical Society. 2012;75(4):31–3.

**Reason for exclusion: subject matter**

Drummond V, Grubert E. Fault lines: Seismicity and the fracturing of energy narratives in Oklahoma. Energy Research & Social Science. 2017;31:128–36.

**Reason for exclusion: off topic**

DuBois RN. The COX-2 story: Is any drug completely “safe?” Gastroenterology. 2006;130(1):6.

**Reason for exclusion: off topic**

Duclos D, Ekzayez A, Ghaddar F, Checchi F, Blanchet K. Localisation and cross-border assistance to deliver humanitarian health services in North-West Syria: a qualitative inquiry for The Lancet-AUB Commission on Syria. Conflict and Health. 2019;13.

**Reason for exclusion: method unsuitable**

Dugas M, Bedard E, Batona G, Kpatchavi AC, Guedou FA, Dube E, et al. Outreach strategies for the promotion of HIV testing and care: Closing the gap between health services and female sex workers in Benin. Journal of Acquired Immune Deficiency Syndromes. 2015;68(Supplement 2):S198–205.

**Reason for exclusion: method unsuitable**

Duncan EL. Atypical Femoral Fracture: A Fascinating Story in Evolution. Journal of Bone and Mineral Research. 2018;33(12):2089–90.

**Reason for exclusion: subject matter**

Duncker D, Veltmann C. The Wearable Cardioverter/Defibrillator - Toy Or Tool? Journal of Atrial Fibrillation. 2016;8(6):1367.

**Reason for exclusion: subject matter**

Dunkley RA, Franklin A. Failing better: The stochastic art of evaluating community-led environmental action programs. Evaluation and Program Planning. 2017;60:112–22.

**Reason for exclusion: off topic**

Dunlop JM, Holosko MJ. The story behind the story of collaborative networks -- relationships do matter! Journal of Health & Social Policy. 2004;19(3):1–18.

**Reason for exclusion: off topic**

Dupain M, Maguire LL %J AJ of HE. Health digital storytelling projects. 2007;38(1):41–3.

**Reason for exclusion: not primary study**

Duraffour S, Andrei G, Topalis D, Krecmerova M, Crance JM, Garin D, et al. Mutations Conferring Resistance to Viral DNA Polymerase Inhibitors in Camelpox Virus Give Different Drug-Susceptibility Profiles in Vaccinia Virus. Journal of Virology. 2012;86(13):7310–25.

**Reason for exclusion: off topic**

Duwe EAG. Toward a Story Powerful Enough to Reduce Health Inequities in Indian Country: The Case of Diabetes. Qualitative Inquiry. 2016;22(8):624–35.

**Reason for exclusion: method unsuitable**

Dwamena FC, Mavis B, Holmes-Rovner M, Walsh KB, Loyson AC. Teaching medical interviewing to patients: The other side of the encounter. Patient Education and Counseling. 2009;76(3):380–4.

**Reason for exclusion: subject matter**

Dwivedi P. Violation of human rights of leprosy afflicted persons: Some real life experiences. Indian Journal of Leprosy. 2018;90(1):35–45.

**Reason for exclusion: off topic**

Dya AFC, Oretaa AWC. Seismic vulnerability assessment of soft story irregular buildings using pushover analysis. In: Procedia Engineering. 2015. p. 925–32.

**Reason for exclusion: off topic**

Díaz LM. Spiritual Conversation as Religiously Educative. Religious Education. 2017;112(5):477–90.

**Reason for exclusion: subject matter**

D’Amore M, McCloskey L, Bokhour B, Paasche-Orlow M, Parker V. Discussions about pregnancy prevention with health care clinicians: Findings from qualitative interviews with young black women in Boston. Contraception. 2012;86 (3):310–1.

**Reason for exclusion: only conference abstract**

D’Emidio-Caston M, Brown JH. The other side of the story - Student narratives on the California drug, alcohol, and tobacco education programs. Evaluation Review. 1998;22(1):95–117.

**Reason for exclusion: method unsuitable**

D’Emidio-Caston M, Brown JH. The other side of the story. Student narratives on the California Drug, Alcohol, and Tobacco Education Programs. Evaluation Review. 1998;22(1):95–117.

**Reason for exclusion: subject matter**

Eagleson C, Cvejic RC, Weise J, Davies K, Trollor JN. Subspecialty training pathways in intellectual and developmental disability psychiatry in Australia and New Zealand: current status and future opportunities. Australasian Psychiatry.

**Reason for exclusion: method unsuitable**

Eakle R, Bourne A, Jarrett C, Stadler J, Larson H. Motivations and barriers to uptake and use of female-initiated, biomedical HIV prevention products in sub-Saharan Africa: an adapted meta-ethnography. BMC Public Health. 2017;17.

**Reason for exclusion: method unsuitable**

Easley MW. Celebrating 50 years of fluoridation: a public health success story. British Dental Journal. 1995;178(2):72–5.

**Reason for exclusion: subject matter**

Ebbesen LS, Woodard GB, McLean S, Butler-Jones D, Green K, Reeder BA, et al. The Saskatchewan dissemination story. Promotion et Education. 2001;Suppl 1:35–9.

**Reason for exclusion: off topic**

Ebneter DS, Latner JD, O’Brien KS. Just world beliefs, causal beliefs, and acquaintance: Associations with stigma toward eating disorders and obesity. Personality and Individual Differences. 2011;51(5):618–22.

**Reason for exclusion: off topic**

Ebunlomo EO, Lazarus M, Creamer A, Hanley L, Crooks D, Hernandez E, et al. Houston, we have a (heart failure education) problem: Results of a heart failure needs assessment survey and resultant educational program. Journal of Cardiac Failure. 2017;23 (8 Supplement 1):S85.

**Reason for exclusion: subject matter**

Eckerle I, Briciu VT, Ergonul O, Lupse M, Papa A, Radulescu A, et al. Emerging souvenirs-clinical presentation of the returning traveller with imported arbovirus infections in Europe. Clinical Microbiology and Infection. 2018;24(3):240–5.

**Reason for exclusion: off topic**

Eckhardt MR, Kerr J, Taylor WC. Point-of-Decision Signs and Stair Use in a University Worksite Setting: General Versus Specific Messages. American Journal of Health Promotion. 2015;29(5):291–3.

**Reason for exclusion: subject matter**

Eckman CD, Weil BT. Institutional open access funds: now is the time. Plos Biology. 2010;8(5):e1000375.

**Reason for exclusion: subject matter**

Edmonds B. A Context- and Scope-Sensitive Analysis of Narrative Data to Aid the Specification of Agent Behaviour. Jasss-the Journal of Artificial Societies and Social Simulation. 2015;18(1).

**Reason for exclusion: off topic**

Edmondson M. A medical career in the shadow and spotlight of huntington’s disease. Neurotherapeutics. 2012;9 (1):226.

**Reason for exclusion: subject matter**

Edwards YJK, Bryson K, Jones DT. A Meta-Analysis of Microarray Gene Expression in Mouse Stem Cells: Redefining Stemness. PLoS ONE [Electronic Resource]. 2008;3(7).

**Reason for exclusion: off topic**

Egan KM, Sosman JA, Blot WJ. Sunlight and reduced risk of cancer: Is the real story vitamin D? Journal of the National Cancer Institute. 2005;97(3):161–3.

**Reason for exclusion: subject matter**

Egeland G, Yohannes S, Okalik L, Kilabuk J, Racicot C, Wilcke M, et al. The value of Inuit elders’ storytelling to health promotion during times of rapid climate change and uncertain food security. 2013;141–57.

**Reason for exclusion: not primary study**

Egerer M. Alcoholism, brief intervention and the institutional context: A focus-group study with French and Finnish general practitioners. Critical Public Health. 2012;22(3):307–18.

**Reason for exclusion: subject matter**

Eika E. Universally Designed Text on the Web: Towards Readability Criteria Based on Anti-Patterns. Studies in Health Technology & Informatics. 2016;229:461–70.

**Reason for exclusion: subject matter**

Ein-Dor T, Mikulincer M, Shaver PR. Attachment Insecurities and the Processing of Threat-Related Information: Studying the Schemas Involved in Insecure People’s Coping Strategies. Journal of Personality and Social Psychology. 2011;101(1):78–93.

**Reason for exclusion: off topic**

Eisenhauer EA. NDDO honorary award lecture 2012 miles to go before we sleep: Fifteen years of targeted therapy development and the path ahead. Annals of Oncology. 2012;1):i15.

**Reason for exclusion: off topic**

Ekmekci PE. Health and Roma People in Turkey. Balkan Medical Journal. 2016;33(4):377–82.

**Reason for exclusion: off topic**

Ekstrom M, Bajwah S, Bland JM, Currow DC, Hussain J, Johnson MJ. One evidence base; three stories: do opioids relieve chronic breathlessness? Thorax. 2018;73(1):88–90.

**Reason for exclusion: subject matter**

Elhassani SB. Teen pregnancy prevention: not quite a complete success story. Journal - South Carolina Medical Association. 1998;94(8):364.

**Reason for exclusion: off topic**

Eliashberg J, Hui SK, Zhang ZJ. From story line to box office: A new approach for green-lighting movie scripts. Management Science. 2007;53(6):881–93.

**Reason for exclusion: off topic**

Eller NM, Henrikson NB, Opel DJ. Vaccine Information Sources and Parental Trust in Their Child’s Health Care Provider. Health Education and Behavior. 2019;46(3):445–53.

**Reason for exclusion: method unsuitable**

Eller NM, Henrikson NB, Opel DJ. Vaccine Information Sources and Parental Trust in Their Child’s Health Care Provider. Health Education and Behavior. 2019;46(3):445–53.

**Reason for exclusion: off topic**

Ellis JM, Freeman JT, Midgette EP, Sanghvi AP, Sarathy B, Johnson CG, et al. Sharing the Story of the Cardiac Rehab Patient Experience: A QUALITATIVE STUDY. Journal of Cardiopulmonary Rehabilitation and Prevention. 2019;39(4):E13–5.

**Reason for exclusion: no short term sacrifice, long term gain**

Elnashar M, Abdelrahim H, Fetters MD. Cultural competence springs up in the desert: the story of the center for cultural competence in health care at Weill Cornell Medical College in Qatar. Academic Medicine. 2012;87(6):759–66.

**Reason for exclusion: subject matter**

Elrick-Barr CE, Thomsen DC, Preston BL, Smith TF. Perceptions matter: household adaptive capacity and capability in two Australian coastal communities. Regional Environmental Change. 2017;17(4):1141–51.

**Reason for exclusion: off topic**

Elsbach KD. How to pitch a brilliant idea. Harvard Business Review. 2003;81(9):117–23, 134.

**Reason for exclusion: subject matter**

Elshakry M. When science became Western: historiographical reflections. Isis. 2010;101(1):98–109.

**Reason for exclusion: subject matter**

Elwood PC. Aspirin: past, present and future. Clinical Medicine. 2001;1(2):132–7.

**Reason for exclusion: subject matter**

Elwyn G, O’Connor A, Stacey D, Volk R, Edwards A, Coulter A, et al. Developing a quality criteria framework for patient decision aids: online international Delphi consensus process. Bmj-British Medical Journal. 2006;333(7565):417–9.

**Reason for exclusion: off topic**

Emami N, Heinonen J, Marteinsson B, Säynäjoki A, Junnonen JM, Laine J, et al. A life cycle assessment of two residential buildings using two different LCA database-software combinations: Recognizing uniformities and inconsistencies. Buildings. 2019;9(1).

**Reason for exclusion: subject matter**

Emery RJ, Valizadeh F, Kennedy V, Shelton AJ. An analysis of variables influencing the number of radiation overexposure events in Texas from 1970 to 2000. Health Physics. 2005;89(1):46–52.

**Reason for exclusion: subject matter**

Emily N, Joyce O, Robert LR, Anzala A. Clinical research volunteers’ perceptions and experiences of screening for enrolment at KAVI-Institute of Clinical Research, Kenya. BMC Infectious Diseases Conference: Afri Can Forum. 2016;16(Supplement 2).

**Reason for exclusion: subject matter**

Emslie C, Ridge D, Ziebland S, Hunt K. Exploring men’s and women’s experiences of depression and engagement with health professionals: more similarities than differences? A qualitative interview study. BMC Family Practice. 2007;8.

**Reason for exclusion: off topic**

Endris AA, Tadesse M, Alemu E, Musa EO, Abayneh A, Assefa Z. A case-control study to assess risk factors related to cholera outbreak in addis ababa, ethiopia, july 2016. Pan African Medical Journal. 2019;34 (no pagination).

**Reason for exclusion: subject matter**

Eng N. Education Inequality: Broadening Public Attitudes through Framing. Journal of Social Issues. 2016;72(4):676–95.

**Reason for exclusion: off topic**

Engelmann L. Fumigating the Hygienic Model City: Bubonic Plague and the Sulfurozador in Early-Twentieth-Century Buenos Aires. Medical history. 2018;62(3):360–82.

**Reason for exclusion: subject matter**

English R, Poet R. Measuring the revised guessability of graphical passwords. Proceedings - 2011 5th International Conference on Network and System Security, NSS 2011. 2011;364–8.

**Reason for exclusion: off topic**

Englund H. Towards a critique of rights talk in new democracies: the case of legal aid in Malawi. Discourse & Society. 2004;15(5):527–51.

**Reason for exclusion: off topic**

Ennals P, Muchamore I, Langan V, Hall C, Wolstencroft K. Active8: Promoting health beyond the absence of mental illness. The Australian journal of rural health. 2020;14.

**Reason for exclusion: no short term sacrifice, long term gain**

Entwistle VA, France EF, Wyke S, Jepson R, Hunt K, Ziebland S, et al. How information about other people’s personal experiences can help with healthcare decision-making: A qualitative study. Patient Education and Counseling. 2011;85(3):e291–8.

**Reason for exclusion: off topic**

Erez G, Shani M. The untold story--the stories of Ethiopian women. [Hebrew]. Harefuah. 2008;147(12):975–7, 1030.

**Reason for exclusion: subject matter**

Eriksen SJ, Manke B. “Because Being Fat Means Being Sick”: Children at Risk of Type 2 Diabetes. Sociological Inquiry. 2011;81(4):549–69.

**Reason for exclusion: off topic**

Eriksson T, Nilstun T, Edwards A. The ethics of risk communication in lifestyle interventions: Consequences of patient centredness. Health Risk & Society. 2007;9(1):19–36.

**Reason for exclusion: method unsuitable**

Eriksson U, Asplund K, Sellstrom E. Growing up in rural community - children’s experiences of social capital from perspectives of wellbeing. Rural and Remote Health. 2010;10(3).

**Reason for exclusion: subject**

Erlanson DA, De Esch IJP, Jahnke W, Johnson CN, Mortenson PN. Fragment-to-Lead Medicinal Chemistry Publications in 2018. Journal of medicinal chemistry. 2020;8.

**Reason for exclusion: subject matter**

Erviti J, Gorricho J, Saiz LC, Perry T, Wright JM. Rethinking the Appraisal and Approval of Drugs for Fracture Prevention. Frontiers in Pharmacology. 2017;8:265.

**Reason for exclusion: subject matter**

Esacove A. Making sense of sex: Rethinking intentionality. Culture, Health and Sexuality. 2008;10(4):377–90.

**Reason for exclusion: off topic**

Esacove AW, Andringa KR. The process of preventing pregnancy: women’s experiences and emergency contraception use. Qualitative Health Research. 2002;12(9):1235–47.

**Reason for exclusion: no short term sacrifice, long term gain**

Esacove AW. Good sex/bad sex: The individualised focus of US HIV prevention policy in sub-Saharan Africa, 1995-2005. Sociology of Health and Illness. 2013;35(1):33–48.

**Reason for exclusion: off topic**

Escobar ML, Kommers PAM, Beldad A. Using narratives as tools for channeling participation in online communities. Computers in Human Behavior. 2014;37:64–72.

**Reason for exclusion: method unsuitable**

Esen A, Celik P, Yorgancioglu A, Sen FS, Yagiz D. Smoking cessation - Two sides of the story [2]. Journal of Cancer Education. 1999;14(3):176–8.

**Reason for exclusion: off topic**

Esen A, Celik P, Yorgancioglu A, Sen FS, Yagiz D. Smoking cessation--two sides of the story. Journal of Cancer Education. 1999;14(3):176–8.

**Reason for exclusion: off topic**

Esmail R, Duchscherer G, Giesbrecht J, King J, Ritchie P, Zuege D. Prevention of ventilator-associated pneumonia in the calgary health region: a Canadian success story! Healthcare Quarterly. 2008;11(3 Spec No.):129–36.

**Reason for exclusion: subject matter**

Esse C, Koffi VA, Kouame A, Dongo K, Yapi RB, Moro HM, et al. “Koko et les lunettes magiques”: An educational entertainment tool to prevent parasitic worms and diarrheal diseases in Cote d’Ivoire. PLoS Neglected Tropical Diseases [electronic resource]. 2017;11(9):e0005839.

**Reason for exclusion: not a personal story**

Etchegary H. Genetic testing for Huntington’s disease: How is the decision taken? Genetic Testing. 2006;10(1):60–7.

**Reason for exclusion: subject matter**

Evans DG, Barwell J, Eccles DM, Collins A, Izatt L, Jacobs C, et al. The Angelina Jolie effect: how high celebrity profile can have a major impact on provision of cancer related services. Breast Cancer Research. 2014;16(5):442.

**Reason for exclusion: not primary study**

Evans K, Murphy L, de Jong W. Global versus local narratives of REDD: A case study from Peru’s Amazon. Environmental Science & Policy. 2014;35:98–108.

**Reason for exclusion: off topic**

Evans MR, Prout H, Prior L, Tapper-Jones LM, Butler CC. A qualitative study of lay beliefs about influenza immunisation in older people. British Journal of General Practice. 2007;57(538):352–8.

**Reason for exclusion: method unsuitable**

Everson RD. Supplemental Nutrition Assistance Program (SNAP): A Personal Story on Health and Nutrition Education. American Journal of Preventive Medicine. 2017;52(2 Supplement 2):S115–7.

**Reason for exclusion: subject matter**

Eves FF, Webb OJ, Mutrie N. A workplace intervention to promote stair climbing: greater effects in the overweight. Obesity. 2006;14(12):2210–6.

**Reason for exclusion: subject matter**

Eyles JD. Health, environmental assessments and population health: tools for a complex process. Canadian Journal of Public Health Revue Canadienne de Sante Publique. 1999;90 Suppl 1:S31-4.

**Reason for exclusion: subject matter**

Eyre SL, Hoffman V, Millstein SG. The gamesmanship of sex: A model based on African American adolescent accounts. Medical Anthropology Quarterly. 1998;12(4):467–89.

**Reason for exclusion: off topic**

Ezendam NP, Alpay LL, Rovekamp TA, Toussaint PJ. Experimenting with Case-Based Reasoning to Present Educative Health Information on the Internet: The Example of SeniorGezond. Studies in Health Technology & Informatics. 2005;116:867–72.

**Reason for exclusion: off topic**

Ezika EA. Use of case stories to explore the contextual application of cardiovascular health knowledge in Nigeria. Health Education Journal. 2020;

**Reason for exclusion: no short term sacrifice, long term gain**

Ezzy D. Illness narratives: time, hope and HIV. Social Science & Medicine. 2000;50(5):605–17.

**Reason for exclusion: off topic**

Fabelo-Roche JR, Iglesias-More S, Gomez-Garcia AM, Hernandez-Dominguez H, Garcia-Enriquez I. An Intersectoral Intervention to Prevent Early Alcohol Use in Cuban Adolescents. MEDICC review. 2016;18(3):25–8.

**Reason for exclusion: no short term sacrifice, long term gain**

Fails JA, Druin A, Guha ML. Content splitting &amp; space sharing: Collaboratively reading &amp; sharing children’s stories on mobile devices. In: Mobile HCI 2011 - 13th International Conference on Human-Computer Interaction with Mobile Devices and Services. 2011. p. 361–70.

**Reason for exclusion: off topic**

Falzon C, Radel R, Cantor A, d’Arripe-Longueville F. Understanding narrative effects in physical activity promotion: the influence of breast cancer survivor testimony on exercise beliefs, self-efficacy, and intention in breast cancer patients. Supportive Care in Cancer. 2014;23(3):761–8.

**Reason for exclusion: no short term sacrifice, long term gain**

Farach N, Faba G, Julian S, Mejia F, Cabieses B, D’Agostino M, et al. Stories From the Field: The Use of Information and Communication Technologies to Address the Health Needs of Underserved Populations in Latin America and the Caribbean. JMIR Public Health and Surveillance. 2015;1(1):e1.

**Reason for exclusion: off topic**

Farah R, Meri R, Kadis DS, Hutton J, DeWitt T, Horowitz-Kraus T. Hyperconnectivity during screen-based stories listening is associated with lower narrative comprehension in preschool children exposed to screens vs dialogic reading: An EEG study. PLoS ONE [Electronic Resource]. 2019;14(11).

**Reason for exclusion: subject matter**

Farb H, Brown L, Priebe A. Qualitative Methodologies as an Intervention: Beyond Program Planning and Implementation. Journal of Public Health Management & Practice. 2017;23 Suppl 6 Suppl, Gulf Region Health Outreach Program:S59–65.

**Reason for exclusion: subject matter**

Farkas Patenaude A, DeMarco TA, Peshkin BN, Valdimarsdottir H, Garber JE, Schneider KA, et al. Talking to children about maternal BRCA1/2 genetic test results: a qualitative study of parental perceptions and advice. Journal of Genetic Counseling. 2013;22(3):303–14.

**Reason for exclusion: subject matter**

Farnesi BC, Ducharme FM, Blais L, Collin J, Lavoie KL, Bacon SL, et al. Guided asthma self-management or patient self-adjustment? Using patients’ narratives to better understand adherence to asthma treatment. Patient Preference and Adherence. 2019;13:587–97.

**Reason for exclusion: method unsuitable**

Farnesi BC, Ducharme FM, Blais L, Collin J, Lavoie KL, Bacon SL, et al. Guided asthma self-management or patient self-adjustment? Using patients’ narratives to better understand adherence to asthma treatment. Patient Preference and Adherence. 2019;13:587–97.

**Reason for exclusion: no short term sacrifice, long term gain**

Farnsworth A. Cervical screening in Australia: A great success story. Acta Cytologica. 2016;60 (Supplement 1):55.

**Reason for exclusion: off topic**

Farooki A. NCCN Bone Health Task Force: Key recommendations. JNCCN Journal of the National Comprehensive Cancer Network. 2014;12(5 SUPPL.):813–6.

**Reason for exclusion: off topic**

Farrell D, Kostkova P, Lazareck L, Weerasinghe D, Weinberg J, Lecky DM, et al. Developing e-Bug web games to teach microbiology. Journal of Antimicrobial Chemotherapy. 2011;66(SUPPL. 5):v33–8.

**Reason for exclusion: off topic**

Farrell D, Kostkova P, Weinberg J, Lazareck L, Weerasinghe D, Lecky DM, et al. Computer games to teach hygiene: an evaluation of the e-Bug junior game. Journal of Antimicrobial Chemotherapy. 2011;66 Suppl 5:v39-44.

**Reason for exclusion: subject matter**

Favaloro EJ. The Platelet Function Analyser (PFA)-100 and von Willebrand disease: A story well over 16 years in the making. Haemophilia. 2015;21(5):642–5.

**Reason for exclusion: subject matter**

Feagin J, Bennefield Z. Systemic racism and US health care. Social Science & Medicine. 2014;103:7–14.

**Reason for exclusion: method unsuitable**

Featherstone RM, Lyon BJ, Ruffin AB. Library roles in disaster response: an oral history project by the National Library of Medicine. Journal of the Medical Library Association. 2008;96(4):343–50.

**Reason for exclusion: subject matter**

Federici MR. The importance of fidelity in peer-based programs: the case of the Wellness Recovery Action Plan. Psychiatric Rehabilitation Journal. 2013;36(4):314–8.

**Reason for exclusion: subject matter**

Fee M. Racializing narratives: Obesity, diabetes and the “aboriginal” thrifty genotype. Social Science & Medicine. 2006;62(12):2988–97.

**Reason for exclusion: method unsuitable**

Feldman-Savelsberg P. Strange expectations: Cameroonian migrants and their German healthcare providers debate obstetric choices. Global Public Health. 2019;

**Reason for exclusion: no short term sacrifice, long term gain**

Feldman-Savelsberg P. Strange expectations: Cameroonian migrants and their German healthcare providers debate obstetric choices. Global Public Health. 2019;1–13.

**Reason for exclusion: subject matter**

Felt U, Felder K, Penkler M. How differences matter: tracing diversity practices in obesity treatment and health promotion. Sociology of Health & Illness. 2017;39(1):127–42.

**Reason for exclusion: not primary study**

Felton BJ. Defining location in the mental health system: A case study of a consumer-run agency. American Journal of Community Psychology. 2005;36(3–4):373–86.

**Reason for exclusion: off topic**

Feng YH, Bruhn C, Marx D. Evaluation of different food safety education interventions. British Food Journal. 2016;118(4):762–76.

**Reason for exclusion: method unsuitable**

Fepuleai A, Weber E, Nemeth K, Muliaina T, Iese V. Eruption Styles of Samoan Volcanoes Represented in Tattooing, Language and Cultural Activities of the Indigenous People. Geoheritage. 2017;9(3):395–411.

**Reason for exclusion: off topic**

Ferlie E. Public management “reform” narratives and the changing organisation of primary care. London Journal of Primary Care. 2010;(2):76–80.

**Reason for exclusion: subject matter**

Fernald DH, Wearner R, Dickinson WP. The journey of primary care practices to meaningful use: A Colorado Beacon Consortium study. Journal of the American Board of Family Medicine. 2013;26(5):603–11.

**Reason for exclusion: subject matter**

Fernandez WD, Tiriveedhi K. Cowden and map syndrome: The untold story of a screening colonoscopy-case report. American Journal of Gastroenterology. 2019;114 (Supplement):S816–7.

**Reason for exclusion: subject matter**

Fernandez YGE, Paterniti D, Kravitz R. Maternal depression care-seeking initiated in pediatric settings. Clinical and Translational Science. 2013;6 (2):109–10.

**Reason for exclusion: subject matter**

Ferreira DM, Barbosa RMS, Finizola NC, Soares DDB, Henriques P, Pereira S, et al. Perception of the operating agents about the Brazilian National School Feeding Program. Revista de saude publica. 2019;53.

**Reason for exclusion: off topic**

Few R, Lake I, Hunter PR, Tran PG. Seasonality, disease and behavior: Using multiple methods to explore socio-environmental health risks in the Mekong Delta. Social Science & Medicine. 2013;80:1–9.

**Reason for exclusion: subject**

Fiddian-Green A, Kim S, Gubrium AC, Larkey LK, Peterson JC %J H promotion practice. Restor (y) ing Health: A Conceptual Model of the Effects of Digital Storytelling. 2019;20(4):502–12.

**Reason for exclusion: duplicate**

Fiddian-Green A, Kim S, Gubrium AC, Larkey LK, Peterson JC. Restor(y)ing Health: A Conceptual Model of the Effects of Digital Storytelling. Health Promotion Practice. 2019;1524839918825130.

**Reason for exclusion: not primary study**

Fiddian-Green A, Kim S, Gubrium AC, Larkey LK, Peterson JC. Restor(y)ing Health: A Conceptual Model of the Effects of Digital Storytelling. Health Promotion Practice. 2019;20(4):502–12.

**Reason for exclusion: not primary study**

Fielden SJ, Chapman GE, Cadell S. Managing stigma in adolescent HIV: silence, secrets and sanctioned spaces. Culture Health & Sexuality. 2011;13(3):267–81.

**Reason for exclusion: off topic**

Fields EL, Malebranche DJ, Smith KC, Ellen JM. Coping with homo-negativity among young adult and adolescent black men who have sex with men: Implications for HIV prevention. Journal of Adolescent Health. 2013;1):S12–3.

**Reason for exclusion: subject matter**

Fiese BH. Time allocation and dietary habits in the United States: Time for re-evaluation? Physiology & Behavior. 2018;193:205–8.

**Reason for exclusion: off topic**

Figueiro-Filho E, Oliveira V, Breda I, Coelho L, Goes M, Melo L, et al. Association of hereditary thrombophilias and antiphospholipid antibodies with recurrent abortion, fetal loss and severe pre-eclampsia in Brazilian pregnant women. International Journal of Gynecology and Obstetrics. 2009;2):S416–7.

**Reason for exclusion: off topic**

Fijak M, Pilatz A, Hedger MP, Nicolas N, Bhushan S, Michel V, et al. Infectious, inflammatory and “autoimmune” male factor infertility: how do rodent models inform clinical practice? Human Reproduction Update. 2018;24(4):416–41.

**Reason for exclusion: off topic**

Filippova M, Gorbunov I, Chan RW, Shcherbakova O. Unconscious detection of verbal and non-verbal ambiguous stimuli. International Journal of Psychophysiology. 2018;131 (Supplement):S148.

**Reason for exclusion: method unsuitable**

Findholt NE, Michael YL, Davis MM. Photovoice engages rural youth in childhood obesity prevention. Public Health Nursing. 2011;28(2):186–92.

**Reason for exclusion: no short term sacrifice, long term gain**

Finnegan J. The screenplay and the spectator: Exploring audience identification in narrative structure. Journal of Screenwriting. 2016;7(3):319–30.

**Reason for exclusion: off topic**

Fisher C, Heywood W, Lucke J, Brown G. 25 year trends in Australian adolescent sexual health knowledge, perceptions and behaviour. Journal of Sexual Medicine. 2017;14 (5 Supplement 4):e321.

**Reason for exclusion: off topic**

Fisher JW, Brundage SI. The challenge of eliminating cervical cancer in the United States: a story of politics, prudishness, and prevention. Women & Health. 2009;49(2–3):246–61.

**Reason for exclusion: off topic**

Fisher K, Williams M, FitzHerbert S, Instone L, Duffy M, Wright S, et al. Writing difference differently. New Zealand Geographer. 2015;71(1):18–33.

**Reason for exclusion: off topic**

Fisher KM, Green MJ, Orkin FK, Chinchilli VM. A Content Analysis from a US Statewide Survey of Memorable Healthcare Decisions for Individuals with Intellectual Disability. Journal of Intellectual & Developmental Disability. 2009;34(3):258–65.

**Reason for exclusion: subject matter**

Fisher P, Lees J. Narrative approaches in mental health: Preserving the emancipatory tradition. Health. 2016;20(6):599–615.

**Reason for exclusion: off topic**

Fitzgerald G, Ibrahim A, Coffey J, Saunders J, Cahill C, Satti Z, et al. Factors influencing total ischaemic time in stemi. Heart. 2018;104 (Supplement 7):A31–2.

**Reason for exclusion: subject matter**

Fitzgerald K, Paravati E, Green MC, Moore MM, Qian JL. Restorative Narratives for Health Promotion. Health Communication. 2019;1–8.

**Reason for exclusion: subject matter**

Fitzgerald K, Paravati E, Green MC, Moore MM, Qian JL. Restorative Narratives for Health Promotion. Health Communication. 2020;35(3):356–63.

**Reason for exclusion: not primary study**

Fitzpatrick AL, Steinman LE, Tu SP, Ly KA, Ton TG, Yip MP, et al. Using photovoice to understand cardiovascular health awareness in Asian elders. Health Promotion Practice. 2012;13(1):48–54.

**Reason for exclusion: no short term sacrifice, long term gain**

Fitzpatrick K, Allen JM. What does critical health education in schools look like? Two ethnographic narratives of critical practice. Health Education Journal. 2019;

**Reason for exclusion: subject matter**

Fitzpatrick K, Allen JM. What does critical health education in schools look like? Two ethnographic narratives of critical practice. Health Education Journal. 2019;78(6):647–61.

**Reason for exclusion: subject matter**

Flaherty BA. The identification of veterans’ perceptions of health through photographs and storytelling. In: Sigma Theta Tau International 37th Biennial Convention: Proceedings and Awards CD, Toronto, Ontario Canada. 2003.

**Reason for exclusion: can't find study**

Flanagan P. Unpacking Ideas of Sexuality in Childhood: What do primary teachers and parents say? Open Review of Educational Research. 2014;1(1):160–70.

**Reason for exclusion: off topic**

Fleck LM. Just caring: screening needs limits. Journal of medical ethics. 2019;29.

**Reason for exclusion: no short term sacrifice, long term gain**

Fletcher S, Mullett J. Digital stories as a tool for health promotion and youth engagement. Canadian Journal of Public Health-Revue Canadienne De Sante Publique. 2016;107(2):E183–7.

**Reason for exclusion: no short term sacrifice, long term gain**

Fletcher-Lartey SM, Caprarelli G. Application of GIS technology in public health: successes and challenges. Parasitology. 2016;143(4):401–15.

**Reason for exclusion: off topic**

Flisiak R, Prokopowicz D. [Therapeutic vaccination in chronic hepatitis B: chance or utopia?]. Polski Merkuriusz Lekarski. 2003;15(90):503–6.

**Reason for exclusion: off topic**

Florvaag E, Johansson SG. The pholcodine story. Immunology & Allergy Clinics of North America. 2009;29(3):419–27.

**Reason for exclusion: subject matter**

Flottum K, Gjerstad O. Arguing for climate policy through the linguistic construction of narratives and voices: the case of the South-African green paper “National Climate Change Response.” Climatic Change. 2013;118(2):417–30.

**Reason for exclusion: method unsuitable**

Flottum K, Gjerstad U. Narratives in climate change discourse. Wiley Interdisciplinary Reviews-Climate Change. 2017;8(1).

**Reason for exclusion: method unsuitable**

Flynn K, Daiches A, Malpus Z, Yonan N, Sanchez M. “A post-transplant person”: Narratives of heart or lung transplantation and intensive care unit delirium. Health. 2014;18(4):352–68.

**Reason for exclusion: off topic**

Fodeh SJ, Finch D, Bouayad L, Luther SL, Ling H, Kerns RD, et al. Classifying clinical notes with pain assessment using machine learning. Medical and Biological Engineering and Computing. 2018;56(7):1285–92.

**Reason for exclusion: off topic**

Foley R. Performing health in place: The holy well as a therapeutic assemblage. Health and Place. 2011;17(2):470–9.

**Reason for exclusion: off topic**

Fontdevila J. Phenomenologies of the akratic self: Masculinity, regrets, and HIV among men on methadone. Journal of Urban Health-Bulletin of the New York Academy of Medicine. 2006;83(4):586–601.

**Reason for exclusion: method unsuitable**

Foote M, Styles T, Quinn C. Use of unannounced “mystery patient drills” to assess hospital emergency department preparedness for communicable diseases of public health concern in new york city, 2016. Open Forum Infectious Diseases. 2017;4 (Supplement 1):S238.

**Reason for exclusion: off topic**

Forchtner B, Kroneder A, Wetzel D. Being Skeptical? Exploring Far-Right Climate-Change Communication in Germany. Environmental Communication-a Journal of Nature and Culture. 2018;12(5):589–604.

**Reason for exclusion: off topic**

Forge J. Safeguarding children by means of information sharing. Community Practitioner. 2010;83(1):16–9.

**Reason for exclusion: off topic**

Formative Research to Inform Nutrition Interventions in Chuuk and the US Pacific. Journal of the Academy of Nutrition and Dietetics. 2015;115(6):947–53.

**Reason for exclusion: method unsuitable**

Foster GD, Sundal D, Lent MR, McDermott C, Jelalian E, Vojta D. 18-month outcomes of a community-based treatment for childhood obesity. Pediatric Obesity. 2014;9(3):E63–7.

**Reason for exclusion: off topic**

Foster LJJ, Deafenbaugh L, Miller E. Group metaphor map making: Application to integrated arts-based focus groups. Qualitative Social Work. 2018;17(2):305–22.

**Reason for exclusion: off topic**

Fournier B, Illasiak V, Kushner KE, Raine K. The adoption, implementation and maintenance of a school food policy in the Canadian Arctic: A retrospective case study. Health Promotion International. 2019;34(5):902–11.

**Reason for exclusion: subject matter**

Fownes JR, Allred SB. Testing the Influence of Recent Weather on Perceptions of Personal Experience with Climate Change and Extreme Weather in New York State. Weather Climate and Society. 2019;11(1):143–57.

**Reason for exclusion: subject matter**

Fox A, Gillis D, Anderson B, Lordly D. Stronger Together: Use of Storytelling at a Dietetics Conference to Promote Professional Collaboration. Canadian Journal of Dietetic Practice & Research. 2017;78(1):32–6.

**Reason for exclusion: off topic**

Fox DM. The politics of policy development in public health: notes on three stories. Journal of Public Health Management & Practice. 2002;8(1):65–7.

**Reason for exclusion: off topic**

Fox GR, Kaplan J, Damasio H, Damasio A. Neural correlates of gratitude. Frontiers in Psychology. 2015;6.

**Reason for exclusion: off topic**

Fox KR. The influence of physical activity on mental well-being. Public Health Nutrition. 1999;2(3A):411–8.

**Reason for exclusion: off topic**

Foy JE, LoCasto PC, Briner SW, Dyar S. Would a madman have been so wise as this?" The effects of source credibility and message credibility on validation. Memory & Cognition. 2017;45(2):281–95.

**Reason for exclusion: off topic**

Fragu P. Calcitonin’s fantastic voyage: from hormone to marker of a genetic disorder. Gesnerus. 2007;64(1–2):69–92.

**Reason for exclusion: off topic**

Franca S, Marchand C, Craplet C, Basdevant A, d’Ivernois JF. Application of “Concept Mapping” in obese subjects: a pilot study in normo and underreporters. Diabetes & Metabolism. 2003;29(1):72–8.

**Reason for exclusion: off topic**

France EF, Wyke S, Ziebland S, Entwistle VA, Hunt K. How personal experiences feature in women’s accounts of use of information for decisions about antenatal diagnostic testing for foetal abnormality. Social Science and Medicine. 2011;72(5):755–62.

**Reason for exclusion: method unsuitable**

France K, Danesh A, Jirard S. Informing aggression-prevention efforts by comparing perpetrators of brief vs. extended cyber aggression. Computers in Human Behavior. 2013;29(6):2143–9.

**Reason for exclusion: off topic**

Francisco Alvarez E. Finding sequins in the rubble: The journeys of two Latina migrant lesbians in Los Angeles. Journal of Lesbian Studies. 2019;

**Reason for exclusion: off topic**

Franck U, Kruger M, Schwarz N, Grossmann K, Roder S, Schlink U. Heat stress in urban areas: Indoor and outdoor temperatures in different urban structure types and subjectively reported well-being during a heat wave in the city of Leipzig. Meteorologische Zeitschrift. 2013;22(2):167–77.

**Reason for exclusion: off topic**

Franco EL, de Sanjose S, Broker TR, Stanley MA, Chevarie-Davis M, Isidean SD, et al. Human papillomavirus and cancer prevention: gaps in knowledge and prospects for research, policy, and advocacy. Vaccine. 2012;30 Suppl 5:F175-82.

**Reason for exclusion: method unsuitable**

Frank AW. Health stories as connectors and subjectifiers. Health: an Interdisciplinary Journal for the Social Study of Health, Illness & Medicine. 2006;10(4):421–40.

**Reason for exclusion: not primary study**

Frank AW. Interrupted stories, interrupted lives. Second Opinion. 1994;20(1):10–8.

**Reason for exclusion: not primary study**

Frank LB, Murphy ST, Chatterjee JS, Moran MB, Baezconde-Garbanati L. Telling stories, saving lives: creating narrative health messages. Health Communication. 2015;30(2):154–63.

**Reason for exclusion: method unsuitable**

Frank LD, Kavage S. Urban planning and public health: a story of separation and reconnection. Journal of Public Health Management & Practice. 2008;14(3):214–20.

**Reason for exclusion: off topic**

Frank O, Hochreutener M, Wiederkehr P, Staender S. [CIRRNET - learning from errors, a success story]. Therapeutische Umschau. 2012;69(6):341–6.

**Reason for exclusion: not primary study**

Franklin AM, Aga DS, Cytryn E, Durso LM, McLain JE, Pruden A, et al. Antibiotics in Agroecosystems: Introduction to the Special Section. Journal of Environmental Quality. 2016;45(2):377–93.

**Reason for exclusion: off topic**

Frantzeskaki N. Seven lessons for planning nature-based solutions in cities. Environmental Science & Policy. 2019;93:101–11.

**Reason for exclusion: off topic**

Fred HL. Taking the risk: The other side of the story. Texas Heart Institute Journal. 2015;42(1):1.

**Reason for exclusion: subject matter**

Fredenrich-Muhlebach A, Barbe R, Tissot S, Ayerra JM, Guimon J. 10 years of analytical group training “in blocks” in Geneva (Switzerland). European Journal of Psychiatry. 2004;18:44–8.

**Reason for exclusion: off topic**

Freebairn L, Atkinson JA, Osgood ND, Kelly PM, McDonnell G, Rychetnik L. Turning conceptual systems maps into dynamic simulation models: An Australian case study for diabetes in pregnancy. PLoS ONE [Electronic Resource]. 2019;14(6).

**Reason for exclusion: off topic**

Freebairn L, Atkinson JA, Osgood ND, Kelly PM, McDonnell G, Rychetnik L. Turning conceptual systems maps into dynamic simulation models: An Australian case study for diabetes in pregnancy. PLoS ONE [Electronic Resource]. 2019;14(6):e0218875.

**Reason for exclusion: off topic**

Freeman E. Understanding HIV-related stigma in older age in rural Malawi. Social Science and Medicine. 2016;164:35–43.

**Reason for exclusion: method unsuitable**

Freeman R %J I journal of paediatric dentistry. Storytelling, sugar snacking, and toothbrushing rules: a proposed theoretical and developmental perspective on children’s health and oral health literacy. 2015;25(5):339–48.

**Reason for exclusion: off topic**

Freeman R. Storytelling, sugar snacking, and toothbrushing rules: a proposed theoretical and developmental perspective on children’s health and oral health literacy. International journal of paediatric dentistry. 2015;25(5):339–48.

**Reason for exclusion: method unsuitable**

Fricko O, Havlik P, Rogelj J, Klimont Z, Gusti M, Johnson N, et al. The marker quantification of the Shared Socioeconomic Pathway 2: A middle-of-the-road scenario for the 21st century. Global Environmental Change-Human and Policy Dimensions. 2017;42:251–67.

**Reason for exclusion: off topic**

Fried J, Harris B, Eyles J. Hopes interrupted: accessing and experiences of antiretroviral therapy in South Africa. Sexually Transmitted Infections. 2012;88(2):147–51.

**Reason for exclusion: method unsuitable**

Friedland J, Rais H. Helen Primrose LeVesconte: occupational therapy clinician, educator, and maker of history. Canadian Journal of Occupational Therapy - Revue Canadienne d Ergotherapie. 2005;72(3):131–41.

**Reason for exclusion: off topic**

Friedman SR, Mateu-Gelabert P, Sandoval M, Hagan H, Jarlais DCD. Positive deviance control-case life history: A method to develop grounded hypotheses about successful long-term avoidance of infection. BMC Public Health. 2008;8 (no pagination)(94).

**Reason for exclusion: off topic**

Friedman-Wheeler DG, Rizzo-Busack H, McIntosh E, Ahrens AH, Haaga DAF. Manipulating regulatory focus in cigarette smokers. Addictive Behaviors. 2010;35(5):530–2.

**Reason for exclusion: off topic**

Frishkopf M, Zakus D, Abu S, Hamze H, Alhassan M, Zukpeni IA. Traditional music as a sustainable social technology for community health promotion in africa: “singing and dancing for health” in Rural Northern Ghana. Annals of Global Health. 2017;83 (1):38.

**Reason for exclusion: method unsuitable**

Fu SS, Rhodes KL, Robert C, Widome R, Forster JL, Joseph AM. Designing and evaluating culturally specific smoking cessation interventions for American Indian communities. Nicotine & Tobacco Research. 2014;16(1):42–9.

**Reason for exclusion: off topic**

Fullagar S, O’Brien W. Social recovery and the move beyond deficit models of depression: A feminist analysis of mid-life women’s self-care practices. Social Science & Medicine. 2014;117:116–24.

**Reason for exclusion: not primary study**

Fung TK. The role of counterfactual thinking in narrative persuasion: Its impact on patients’ adherence to treatment regimen. Health Communication. 2019;34(12):1482–93.

**Reason for exclusion: no short term sacrifice, long term gain**

Fung TKF. The Role of Counterfactual Thinking in Narrative Persuasion: Its Impact on Patients’ Adherence to Treatment Regimen. Health Communication. 2018;1–12.

**Reason for exclusion: subject matter**

Funnell P. Views from the screen-face: Issues emerging from an exploration of the value of telematics-supported learning. Innovations in Education and Teaching International. 1999;36(3):176–84.

**Reason for exclusion: off topic**

Gabbott T, Roberts H, Briesen S. Assessing the fear of cataract surgery in rural Kenya. Cogent Medicine. 2019;6 (1) (no pagination)(1607434).

**Reason for exclusion: subject matter**

Gabrysiak D. A woman in a man’s world: La Banquière, money, transgression and the world of high finance. Studies in French Cinema. 2015;15(3):225–36.

**Reason for exclusion: off topic**

Gainsbury A, Dowling S. “A little bit offended and slightly patronised”: parents’experiences of National Child Measurement Programme feedback. Public Health Nutrition. 2018;21(15):2884–92.

**Reason for exclusion: method unsuitable**

Gaither BM, Gaither TK. Marketplace Advocacy by the US Fossil Fuel Industries: Issues of Representation and Environmental Discourse. Mass Communication and Society. 2016;19(5):585–603.

**Reason for exclusion: method unsuitable**

Gajewicz A, Jagiello K, Cronin MTD, Leszczynski J, Puzyn T. Addressing a bottle neck for regulation of nanomaterials: quantitative read-across (Nano-QRA) algorithm for cases when only limited data is available. Environmental Science-Nano. 2017;4(2):346–58.

**Reason for exclusion: off topic**

Galanek JD. The Cultural Construction of Mental Illness in Prison: A Perfect Storm of Pathology. Culture Medicine and Psychiatry. 2013;37(1):195–225.

**Reason for exclusion: method unsuitable**

Galea J. Living and Dying in Malta During the Horrible Summer of 1837. Journal of community health. 2019;1.

**Reason for exclusion: subject matter**

Galin D, Raz J, Fein G, Johnstone J, Herron J, Yingling C. EEG spectra in dyslexic and normal readers during oral and silent reading. Electroencephalography and Clinical Neurophysiology. 1992;82(2):87–101.

**Reason for exclusion: off topic**

Galindo L, Maginnis T, Wallace G, Hansen A, Sylvestre D. Education by peers is the key to success. International Journal of Drug Policy. 2007;18(5):411–6.

**Reason for exclusion: off topic**

Galinec M, Korajlija AL. The effect of different ways of providing information about transsexualism on student attitudes. Psychology & Sexuality. 2017;8(4):280–90.

**Reason for exclusion: off topic**

Gallagher B, Green A. In, out and after care: Young adults’ views on their lives, as children, in a therapeutic residential establishment. Children and Youth Services Review. 2012;34(2):437–50.

**Reason for exclusion: method unsuitable**

Gallagher L, McAuley J, Moseley GL. A randomized-controlled trial of using a book of metaphors to reconceptualize pain and decrease catastrophizing in people with chronic pain. Clinical Journal of Pain. 2013;29(1):20–5.

**Reason for exclusion: off topic**

Galvin J, Roe C, Morris J. The AD8 dementia screening test detects mild cognitive impairment. Alzheimer’s and Dementia. 2012;1):P483.

**Reason for exclusion: off topic**

Gambrel LB, Bradley VN, McLaughlin EM. Young children’s comprehension and recall of computer screen displayed text. Journal of Research in Reading. 1987;10(2):156–63.

**Reason for exclusion: off topic**

Game DS, Warrens AN, Lechler RI. Rejection mechanisms in transplantation. Wiener Klinische Wochenschrift. 2001;113(20–21):832–8.

**Reason for exclusion: subject matter**

Ganzer C. USING LITERATURE AS AN AID TO PRACTICE. Families in Society-the Journal of Contemporary Human Services. 1994;75(10):616–23.

**Reason for exclusion: method unsuitable**

Garb Y, Pulver S, VanDeveer SD. Scenarios in society, society in scenarios: toward a social scientific analysis of storyline-driven environmental modeling. Environmental Research Letters. 2008;3(4).

**Reason for exclusion: method unsuitable**

Garcia CR, Kaler SG. Development of the pill. Annals of the New York Academy of Sciences. 2004;1038:223–6.

**Reason for exclusion: off topic**

Garcia DI, Gray-Stanley J, Ramirez-Valles J. “The Priest Obviously Doesn’t Know That I’m Gay”: The Religious and Spiritual Journeys of Latino Gay Men. Journal of Homosexuality. 2008;55(3):411–36.

**Reason for exclusion: off topic**

Garcia-Silberman S. Attitudes toward mental illness and psychiatry: Preliminary results. Salud Mental. 1998;21(4):40–50.

**Reason for exclusion: off topic**

Gard M, Fitzgerald H. Tackling murderball: Disability and the big masculinity, screen. Sport, Ethics and Philosophy. 2008;2(2):126–41.

**Reason for exclusion: off topic**

Gardam M, Reason P, Rykert L. Healthcare culture and the challenge of preventing healthcare-associated infections. Healthcare Quarterly. 2010;13 Spec No:116–20.

**Reason for exclusion: subject matter**

Garista P, Sardu C, Mereu A, Campagna M, Contu P. The mouse gave life to the mountain: Gramsci and health promotion. Health Promotion International. 2015;30(3):746–55.

**Reason for exclusion: off topic**

Garner J. Therioepistemology, a success story: Mouse models of trichotillomania and compulsive skin picking. Laboratory Animals. 2018;52 (2 Supplement 1):19.

**Reason for exclusion: subject matter**

Garrod D. Talkback: a strategic approach to working with maternity service users. Practising Midwife. 2012;15(4):18–20.

**Reason for exclusion: subject matter**

Garvin T, Eyles J. The sun safety metanarrative: Translating science into public health discourse. Policy Sciences. 1997;30(2):47–70.

**Reason for exclusion: method unsuitable**

Gascon J, Vilasanjuan R, Lucas A. The need for global collaboration to tackle hidden public health crisis of Chagas disease. Expert Review of Antiinfective Therapy. 2014;12(4):393–5.

**Reason for exclusion: subject matter**

Gatune JW, Nyamongo IK. An ethnographic study of cervical cancer among women in rural Kenya: Is there a folk causal model? International Journal of Gynecological Cancer. 2005;15(6):1049–59.

**Reason for exclusion: subject matter**

Gault I, Gallagher A, Chambers M. Perspectives on medicine adherence in service users and carers with experience of legally sanctioned detention and medication: a qualitative study. Patient Preference and Adherence. 2013;7:787–99.

**Reason for exclusion: off topic**

Gautam OP, Schmidt WP, Cairncross S, Cavill S, Curtis V. Trial of a Novel Intervention to Improve Multiple Food Hygiene Behaviors in Nepal. American Journal of Tropical Medicine & Hygiene. 2017;96(6):1415–26.

**Reason for exclusion: method unsuitable**

Gaver W. Cultural commentators: Non-native interpretations as resources for polyphonic assessment. International Journal of Human Computer Studies. 2007;65(4):292–305.

**Reason for exclusion: off topic**

Gearty MR, Bradbury-Huang H, Reason P. Learning history in an open system: Creating histories for sustainable futures. Management Learning. 2015;46(1):44–66.

**Reason for exclusion: off topic**

Geerlings E, Kaselitz E, Aborigo RA, Williams J, Youngblood J, Avrakotos A, et al. “I am still confused as to what caused the problem”: Perceptions of mothers on communication regarding newborn illness and death in Northern Ghana. Global Public Health.

**Reason for exclusion: no short term sacrifice, long term gain**

Genrich GL, Brathwaite BA. Response of religious groups to HIV/AIDS as a sexually transmitted infection in Trinidad. BMC Public Health. 2005;5.

**Reason for exclusion: off topic**

Genus A, Theobald K. Creating low-carbon neighbourhoods: a critical discourse analysis. European Urban and Regional Studies. 2016;23(4):782–97.

**Reason for exclusion: off topic**

George MD, Holder HD, McKenzie PN, Mueller HR, Herchek DC, Faile BS. Replication of a Controlled Community Prevention Trial: Results From a Local Implementation of Science-Based Intervention to Reduce Impaired Driving. Journal of Primary Prevention. 2018;39(1):47–58.

**Reason for exclusion: subject matter**

Gerald L. Social determinants of health. North Carolina Medical Journal. 2012;73(5):353–7.

**Reason for exclusion: off topic**

Gerling W. PHOTOGRAPHY IN THE DIGITAL: Screenshot and in-game photography. Photographies. 2018;11(2–3):149–67.

**Reason for exclusion: off topic**

Gershon RRM, Qureshi KA, Rubin MS, Raveis VH. Factors Associated with High-Rise Evacuation: Qualitative Results from the World Trade Center Evacuation Study. Prehospital and Disaster Medicine. 2007;22(3):165–73.

**Reason for exclusion: off topic**

Gesler W. Hans Castorp’s journey-to-knowledge of disease and health in Thomas Mann’s The Magic Mountain. Health and Place. 2000;6(2):125–34.

**Reason for exclusion: off topic**

Gibson B, Butler J, Schnock K, Bates D, Classen D. Design of a safety dashboard for patients. Patient education and counseling. 2019;9.

**Reason for exclusion: subject matter**

Gibson D, Medd D, McCarthy T, Toldo B, Moselely P, Pudin V. Innovative multi-zone water injection: The Enfield ENC05 story. In: Society of Petroleum Engineers - SPE Asia Pacific Oil and Gas Conference and Exhibition 2010, APOGCE 2010. 2010. p. 2003–12.

**Reason for exclusion: off topic**

Gibson E, Gracey G, Ng C, O’Neill T, Smyth R, Rocks G, et al. Higher risk breast screening: cancer detection rates, recall rates, and attendance rates in Northern Ireland. Clinical Radiology. 2019;74(8):654.e1-654.e5.

**Reason for exclusion: off topic**

Gibson E, Gracey G, Ng C, O’Neill T, Smyth R, Rocks G, et al. Higher risk breast screening: cancer detection rates, recall rates, and attendance rates in Northern Ireland. Clinical Radiology. 2019;74(8):654.e1-654.e5.

**Reason for exclusion: no short term sacrifice, long term gain**

Gibson K, Cartwright C, Read J. Conflict in Men’s Experiences With Antidepressants. American Journal of Mens Health. 2018;12(1):104–16.

**Reason for exclusion: method unsuitable**

Gibson KL, Coulson H, Miles R, Kakekakekung C, Daniels E, O’Donnell S. Conversations on telemental health: listening to remote and rural First Nations communities. Rural and Remote Health. 2011;11(2).

**Reason for exclusion: off topic**

Gibson LM. Teaching strategies to facilitate breast cancer screening by African-American women. Journal of National Black Nurses Association. 2008;19(2):42–9.

**Reason for exclusion: method unsuitable**

Gilbert H. Re-visioning Local Biologies: HIV-2 and the Pattern of Differential Valuation in Biomedical Research. Medical Anthropology. 2013;32(4):343–58.

**Reason for exclusion: off topic**

Giles-Vernick T, Traore A, Bainilago L. Incertitude, Hepatitis B, and Infant Vaccination in West and Central Africa. Medical Anthropology Quarterly. 2016;30(2):203–21.

**Reason for exclusion: off topic**

Gillard S, Borschmann R, Turner K, Goodrich-Purnell N, Lovell K, Chambers M. Producing different analytical narratives, coproducing integrated analytical narrative: a qualitative study of UK detained mental health patient experience involving service user researchers. International Journal of Social Research Methodology. 2012;15(3):239–54.

**Reason for exclusion: off topic**

Gillespie S, Hoddinott J, Nisbett N, Arifeen S, van den Bold M. Evidence to Action: Highlights From Transform Nutrition Research (2012-2017). Food & Nutrition Bulletin. 2018;39(3):335–60.

**Reason for exclusion: off topic**

Girard G. HIV risk and sense of community: French gay male discourses on barebacking. Culture Health & Sexuality. 2016;18(1):15–29.

**Reason for exclusion: off topic**

Gizachew B, Astrup R, Vedeld P, Zahabu EM, Duguma LA. REDD plus in Africa: contexts and challenges. Natural Resources Forum. 2017;41(2):92–104.

**Reason for exclusion: off topic**

Gjerde JL, Rortveit G, Adefris M, Mekonnen H, Belayneh T, Blystad A. The lucky ones get cured: Health care seeking among women with pelvic organ prolapse in Amhara Region, Ethiopia. PLoS ONE [Electronic Resource]. 2018;13 (11) (no pagination)(e0207651).

**Reason for exclusion: subject matter**

Glasgow RE. HMC research translation: speculations about making it real and going to scale. American Journal of Health Behavior. 2010;34(6):833–40.

**Reason for exclusion: subject matter**

Glenn NM, Lapalme J, McCready G, Frohlich KL. Young adults’ experiences of neighbourhood smoking-related norms and practices: A qualitative study exploring place-based social inequalities in smoking. Social Science & Medicine. 2017;189:17–24.

**Reason for exclusion: method unsuitable**

Glenton C, Nilsen ES, Carlsen B. Lay perceptions of evidence-based information - A qualitative evaluation of a website for back pain sufferers. BMC Health Services Research. 2006;6 (no pagination)(34).

**Reason for exclusion: subject matter**

Glenton C, Nilsen ES, Carlsen B. Lay perceptions of evidence-based information--a qualitative evaluation of a website for back pain sufferers. BMC Health Services Research. 2006;6:34.

**Reason for exclusion: subject matter**

Glick JL, Lopez A, Pollock M, Theall KP. “Housing Insecurity Seems to Almost Go Hand in Hand with Being Trans”: Housing Stress among Transgender and Gender Non-conforming Individuals in New Orleans. Journal of urban health : bulletin of the New York Academy of Medicine. 2019;96(5):751–9.

**Reason for exclusion: subject matter**

Godakandage SSP, Senarath U, Jayawickrama HS, Siriwardena I, Wickramasinghe S, Arumapperuma P, et al. Policy and stakeholder analysis of infant and young child feeding programmes in Sri Lanka. BMC Public Health. 2017;17.

**Reason for exclusion: method unsuitable**

Goddu AP, Raffel KE, Peek ME. A story of change: The influence of narrative on African-Americans with diabetes. Patient Education and Counseling. 2015;98(8):1017–24.

**Reason for exclusion: no short term sacrifice, long term gain**

Gola KA, Thorne A, Veldhuisen LD, Felix CM, Hankinson S, Pham J, et al. Neural substrates of spontaneous narrative production in focal neurodegenerative disease. Neuropsychologia. 2015;Part A. 79:158–71.

**Reason for exclusion: subject matter**

Goldsteen M, Houtepen R, Proot IM, Abu-Saad HH, Spreeuwenberg C, Widdershoven G. What is a good death? Terminally ill patients dealing with normative expectations around death and dying. Patient Education and Counseling. 2006;64(1–3):378–86.

**Reason for exclusion: subject matter**

Goldstein H, Kelley E, Greenwood C, McCune L, Carta J, Atwater J, et al. Embedded Instruction Improves Vocabulary Learning During Automated Storybook Reading Among High-Risk Preschoolers. Journal of Speech Language & Hearing Research. 2016;59(3):484–500.

**Reason for exclusion: method unsuitable**

Goldstein H, Olszewski A, Haring C, Greenwood CR, McCune L, Carta J, et al. Efficacy of a Supplemental Phonemic Awareness Curriculum to Instruct Preschoolers With Delays in Early Literacy Development. Journal of Speech Language & Hearing Research. 2017;60(1):89–103.

**Reason for exclusion: off topic**

Gomersall T, Madill A, Summers LKM. Getting one’s thoughts straight: A dialogical analysis of women’s accounts of poorly controlled type 2 diabetes. Psychology & Health. 2012;27(3):378–93.

**Reason for exclusion: method slightly unsuitable**

Gomes R, Leal AF, Knauth D, da Silva GSN. Meanings attributed to policy directed to men’s health. Ciencia e Saude Coletiva. 2012;17(10):2589–96.

**Reason for exclusion: subject matter**

Gomm J, Heath MA, Mora P. Analysis of Latino award winning children’s literature. School Psychology International. 2017;38(5):507–22.

**Reason for exclusion: off topic**

Gontijo DT, de Sena e Vasconcelos AC, Monteiro RJ, Facundes VL, Trajano Md.e F, de Lima LS. Occupational Therapy and Sexual and Reproductive Health Promotion in Adolescence: A Case Study. Occupational Therapy International. 2016;23(1):19–28.

**Reason for exclusion: off topic**

Gontijo DT, de Sena e Vasconcelos AC, Monteiro RJ, Facundes VL, Trajano Mde F, de Lima LS. Occupational Therapy and Sexual and Reproductive Health Promotion in Adolescence: A Case Study. Occupational Therapy International. 2016;23(1):19–28.

**Reason for exclusion: subject matter**

Gonzalez CM, Liguori AR, Grayson MS. How assumptions and preferences affect patient care: An introduction to implicit bias for first year medical students. Journal of General Internal Medicine. 2016;1):S823–4.

**Reason for exclusion: subject matter**

Gonzalez R. Approaches to substance abuse in Cuba: Ricardo A. Gonzalez MD PhD DrSc. Psychiatrist and consulting professor, Eduardo B. Ordaz Psychiatric Hospital, Havana. Interviewed by Christina Mills. MEDICC review. 2013;15(4):6–10.

**Reason for exclusion: off topic**

Goode PS, Markland AD, Echt KV, Slay L, Barnacastle S, Hale G, et al. A mobile telehealth program for behavioral treatment of urinary incontinence in women veterans: Development and pilot evaluation of MyHealtheBladder. Neurourology and Urodynamics. 2020;39(1):432–9.

**Reason for exclusion: subject matter**

Goodwill AM, Campbell S, Henderson VW, Gorelik A, Dennerstein L, McClung M, et al. Robust norms for neuropsychological tests of verbal episodic memory in Australian women. Neuropsychology. 2019;33(4):581–95.

**Reason for exclusion: subject matter**

Goodwin SS. iBiology: communicating the process of science. Molecular Biology of the Cell. 2014;25(15):2217–9.

**Reason for exclusion: off topic**

Gordon R, Waitt G, Cooper P, Butler K. Storying energy consumption: Collective video storytelling in energy efficiency social marketing. Journal of Environmental Management. 2018;213:1–10.

**Reason for exclusion: subject matter**

Gorniewicz J, Floyd M, Krishnan K, Bishop TW, Tudiver F, Lang F. Breaking bad news to patients with cancer: A randomized control trial of a brief communication skills training module incorporating the stories and preferences of actual patients. Patient Education and Counseling. 2017;100(4):655–66.

**Reason for exclusion: method slightly unsuitable**

Gosling RD, Whittaker M, Gueye CS, Fullman N, Baquilod M, Kusriastuti R, et al. Malaria elimination gaining ground in the Asia Pacific. Malaria Journal. 2012;11:346.

**Reason for exclusion: subject matter**

Gosselin P, Belanger D, Lapaige V, Labbe Y. The burgeoning field of transdisciplinary adaptation research in Quebec (1998-): a climate change-related public health narrative. Journal of multidisciplinary healthcare. 2011;4:337–48.

**Reason for exclusion: off topic**

Gozali JP, Kan MY, Sundaram H. How do people organize their photos in each event and how does it affect storytelling, searching and interpretation tasks? In: Proceedings of the ACM/IEEE Joint Conference on Digital Libraries. 2012. p. 315–24.

**Reason for exclusion: subject matter**

Gozukara F, Ersin F, Simsek Z, Yildirimkaya G. The fertility characteristics of the seasonal migratory agricultural women in Turkey. European Journal of Contraception and Reproductive Health Care. 2014;1):S148–9.

**Reason for exclusion: subject matter**

Grace D, Chown S, Jollimore J, Parry R, Kwag M, Steinberg M, et al. Seroadaptive behaviours as context-de pendent prevention strategies among HIV-negative gay men. Canadian Journal of Infectious Diseases and Medical Microbiology. 2013;SA):15A.

**Reason for exclusion: method unsuitable**

Grace D, Gaspar M, Paquette R, Rosenes R, Burchell AN, Grennan T, et al. HIV-positive gay men’s knowledge and perceptions of Human Papillomavirus (HPV) and HPV vaccination: A qualitative study. PLoS ONE [Electronic Resource]. 2018;13(11).

**Reason for exclusion: method unsuitable**

Graham CC, van Dopperen R. Roger Casement on Screen: The Background Story on an Historical Film Opportunity, 1915–1916. Historical Journal of Film, Radio and Television. 2016;36(4):493–508.

**Reason for exclusion: off topic**

Graham EL. Frankensteins and cyborgs: visions of the global future in an age of technology. Studies in Christian ethics. 2003;16(1):29–43.

**Reason for exclusion: subject matter**

Graham JV, Hogg DR. John’s story - living with hereditary haemochromatosis. Rural and Remote Health. 2019;19(2).

**Reason for exclusion: off topic**

Graham JV, Hogg DR. John’s story - living with hereditary haemochromatosis. Rural and Remote Health. 2019;19(2).

**Reason for exclusion: off topic**

Graham L, Mphaphuli M. “A Guy ‘Does’ and You Don’t, They Do You Instead”: Young People’s Narratives of Gender and Sexuality in a Low-Income Context of South Africa. SAGE Open. 2018;8(4).

**Reason for exclusion: off topic**

Gram-Hanssen I. The role of flexibility in enabling transformational social change: Perspectives from an Indigenous community using Q-methodology. Geoforum. 2019;100:10–20.

**Reason for exclusion: method slightly unsuitable**

Grammatiki M, Karras S, Kotsa K. The role of vitamin D in the pathogenesis and treatment of diabetes mellitus: a narrative review. Hormones-International Journal of Endocrinology and Metabolism. 2019;18(1):37–48.

**Reason for exclusion: method unsuitable**

Grant C, Anderson N, Machila N. Stakeholder Narratives on Trypanosomiasis, Their Effect on Policy and the Scope for One Health. PLoS Neglected Tropical Diseases. 2015;9 (12) (no pagination)(e0004241).

**Reason for exclusion: subject matter**

Grasser S, Schunko C, Vogl CR. School kids as ethnobotanists - Knowledge transmission in the Biosphere reserve Groses Walsertal (Austria). Revista de Fitoterapia. 2010;1):164.

**Reason for exclusion: subject matter**

Gratzer G, Keeton WS. Mountain Forests and Sustainable Development: The Potential for Achieving the United Nations’ 2030 Agenda. Mountain Research and Development. 2017;37(3):246–53.

**Reason for exclusion: off topic**

Graves JAM. Marsupial genomics meet marsupial reproduction. Reproduction, Fertility and Development. 2019;31(7):1181–8.

**Reason for exclusion: off topic**

Gray RE, Sinding C, Fitch MI. Navigating the social context of metastatic breast cancer: reflections on a project linking research to drama. Health. 2001;5(2):233–48.

**Reason for exclusion: method unsuitable**

Graybill JK. Mapping an emotional topography of an ecological homeland: The case of Sakhalin Island, Russia. Emotion Space and Society. 2013;8:39–50.

**Reason for exclusion: subject matter**

Green CA, Estroff SE, Yarborough BJH, Spofford M, Solloway MR, Kitson RS, et al. Directions for Future Patient-Centered and Comparative Effectiveness Research for People With Serious Mental Illness in a Learning Mental Health Care System. Schizophrenia Bulletin. 2014;40:S1–+.

**Reason for exclusion: off topic**

Green DR, Gershon RK. Contrasuppression: the second law of thymodynamics, revisited. Advances in Cancer Research. 1984;42:277–335.

**Reason for exclusion: subject matter**

Green MJ, Czerwiec M. Graphic medicine: The best of 2016. JAMA - Journal of the American Medical Association. 2016;316(24):2580–1.

**Reason for exclusion: subject matter**

Greene DW. Development of new plant growth regulators from a university perspective. Horttechnology. 2002;12(1):71–4.

**Reason for exclusion: subject**

Greenfield BK, Melwani AR, Bay SM. A Tiered Assessment Framework to Evaluate Human Health Risk of Contaminated Sediment. Integrated Environmental Assessment and Management. 2015;11(3):459–73.

**Reason for exclusion: subject**

Greenhalgh T, Clinch M, Afsar N, Choudhury Y, Sudra R, Campbell-Richards D, et al. Socio-cultural influences on the behaviour of South Asian women with diabetes in pregnancy: Qualitative study using a multi-level theoretical approach. BMC Medicine. 2015;13 (1) (no pagination)(120).

**Reason for exclusion: method slightly unsuitable**

Greenhalgh T, Collard A, Begum N. Sharing stories: complex intervention for diabetes education in minority ethnic groups who do not speak English. BMJ. 2005;330(7492):628.

**Reason for exclusion: no short term sacrifice, long term gain**

Greenhalgh T, Collard A, Campbell-Richards D, Vijayaraghavan S, Malik F, Morris J, et al. Storylines of self-management: Narratives of people with diabetes from a multiethnic inner city population. Journal of Health Services Research and Policy. 2011;16(1):37–43.

**Reason for exclusion: no short term sacrifice, long term gain**

Greenhalgh T, Fahy N. Research impact in the community-based health sciences: An analysis of 162 case studies from the 2014 UK Research Excellence Framework. BMC Medicine. 2015;13 (1) (no pagination)(232).

**Reason for exclusion: subject matter**

Greenhalgh T, Robb N, Scambler G. Communicative and strategic action in interpreted consultations in primary health care: a Habermasian perspective. Social Science & Medicine. 2006;63(5):1170–87.

**Reason for exclusion: method unsuitable**

Greeven S, Kraan O, Chappin EJL, Kwakkel JH. The Emergence of Climate Change Mitigation Action by Society: An Agent-Based Scenario Discovery Study. Jasss-the Journal of Artificial Societies and Social Simulation. 2016;19(3).

**Reason for exclusion: off topic**

Gregg JL. An Unanticipated Source of Hope: Stigma and Cervical Cancer in Brazil. Medical Anthropology Quarterly. 2011;25(1):70–84.

**Reason for exclusion: off topic**

Gregorius S. Healthcare-seeking behaviour and experiences of rehabilitation among disabled youth in Ghana. Tropical Medicine and International Health. 2015;1):88.

**Reason for exclusion: subject matter**

Griffiths EK, Marley JV, Friello D, Atkinson DN. Uptake of long-acting, reversible contraception in three remote Aboriginal communities: a population-based study. Medical Journal of Australia. 2016;205(1):21–5.

**Reason for exclusion: subject**

Grimell J. Advancing an understanding of selves in transition: I-positions as an analytical tool. Culture & Psychology. 2018;24(2):190–211.

**Reason for exclusion: off topic**

Grimes DR. On the viability of conspiratorial beliefs. PLoS ONE [Electronic Resource]. 2016;11 (1) (no pagination)(e0147905).

**Reason for exclusion: off topic**

Grimes TS, Hou SI. “A Breast Ain’t Nothing but a Sandwich”: Narratives of Ella, an African American Social Worker Breast Cancer Survivor. Social Work in Public Health. 2013;28(1):44–53.

**Reason for exclusion: no short term sacrifice, long term gain**

Grimmer-Somers K, Johnston K, Somers E, Luker J, Alemao LA, Jones D. A holistic client-centred program for vulnerable frequent hospital attenders: cost efficiencies and changed practices. Australian and New Zealand Journal of Public Health. 2010;34(6):609–12.

**Reason for exclusion: off topic**

Grimshaw A. Telling stories, screening lives: notes towards an anthropological biography. Social Anthropology. 2020;28(1):168–83.

**Reason for exclusion: off topic**

Grissinger M. Telling true stories is an ISMP hallmark: Here’s why you should tell stories, Too. P and T. 2014;39(10):658–9.

**Reason for exclusion: not primary study**

Groes-Green C. Safe sex pioneers: class identity, peer education and emerging masculinities among youth in Mozambique. Sexual Health. 2009;6(3):233–40.

**Reason for exclusion: off topic**

Grohe B. Measuring residents’ perceptions of defensible space compared to incidence of crime. Risk Management-an International Journal. 2011;13(1–2):43–61.

**Reason for exclusion: off topic**

Groleau D, Zelkowitz P, Cabral IE. Enhancing generalizability: moving from an intimate to a political voice. Qualitative Health Research. 2009;19(3):416–26.

**Reason for exclusion: off topic**

Grolimund AP. Global health promotion in a local context: “Active ageing” in Dar es Salaam, Tanzania. Tropical Medicine and International Health. 2015;1):247.

**Reason for exclusion: off topic**

Grove KJ. From Emergency Management to Managing Emergence: A Genealogy of Disaster Management in Jamaica. Annals of the Association of American Geographers. 2013;103(3):570–88.

**Reason for exclusion: method unsuitable**

Grove NJ, Zwi AB. Our health and theirs: Forced migration, othering, and public health. Social Science & Medicine. 2006;62(8):1931–42.

**Reason for exclusion: off topic**

Grover JM, Alabdrabalnabi T, Patel MD, Bachman MW, Platts-Mills TF, Cabanas JG, et al. Measuring a Crisis: Questioning the Use of Naloxone Administrations as a Marker for Opioid Overdoses in a Large US EMS System. Prehospital Emergency Care. 2018;22(3):281–9.

**Reason for exclusion: off topic**

Groves AK, Maman S, Msomi S, Makhanya N, Moodley D. The complexity of consent: Women’s experiences testing for HIV at an antenatal clinic in Durban, South Africa. AIDS Care - Psychological and Socio-Medical Aspects of AIDS/HIV. 2010;22(5):538–44.

**Reason for exclusion: method unsuitable**

Guber P. The four truths of the storyteller. Harvard Business Review. 2007;85(12):52–9, 142.

**Reason for exclusion: off topic**

Gubrium A %J H promotion practice. Digital storytelling: An emergent method for health promotion research and practice. 2009;10(2):186–91.

**Reason for exclusion: not primary study**

Gubrium A, Krause EL, Lucey K. “Doing Your Life”: Narrative Intervention with Young Mothers as Storytellers. Human Organization. 2018;77(3):214–27.

**Reason for exclusion: subject matter**

Gubrium AC, Difulvio GT %J GS. Girls in the world: Digital storytelling as a feminist public health approach. 2011;4(2):28–46.

**Reason for exclusion: subject matter**

Gubrium AC, Fiddian-Green A, Lowe S, DiFulvio G, Del Toro-Mejias L %J QHR. Measuring down: Evaluating digital storytelling as a process for narrative health promotion. 2016;26(13):1787–801.

**Reason for exclusion: duplicate**

Gubrium AC, Graham LF, Lowe S, Paterno M, Fiddian-Green A. The Ethics and Practice of Digital Storytelling as a Methodology for Community-Based Participatory Research in Public Health. 2017;

**Reason for exclusion: not primary study**

Gucciardi E, Jean-Pierre N, Karam G, Sidani S. Designing and delivering facilitated storytelling interventions for chronic disease self-management: a scoping review. BMC Health Services Research. 2016;16:249.

**Reason for exclusion: off topic**

Gudnadottir U, Fritz J, Zerbel S, Bernardo A, Sethi AK, Safdar N. Reducing health care-associated infections: Patients want to be engaged and learn about infection prevention. American Journal of Infection Control. 2013;41(11):955–8.

**Reason for exclusion: method slightly unsuitable**

Guerrero N, Small AL, Schwei RJ, Jacobs EA. Informing physician strategies to overcome language barriers in encounters with pediatric patients. Patient Education & Counseling. 2018;101(4):653–8.

**Reason for exclusion: subject**

Guha T, Kumar N, Narayanan SS, Smith SL. Computationally deconstructing movie narratives: An informatics approach. In: ICASSP, IEEE International Conference on Acoustics, Speech and Signal Processing - Proceedings. 2015. p. 2264–8.

**Reason for exclusion: not primary study**

Guilloton L, Latombe D, Camdessanche JP, Thomas Anterion C, Mercier B, Roggerone S, et al. Cognitive disorders in multiple sclerosis: A screening tool proposed in current practice. the experience of the Rhone-Alpes SEP Network. Multiple Sclerosis Journal. 2017;23 (3 Supplement 1):833–4.

**Reason for exclusion: subject matter**

Guin S. A qualitative exploration of HIV/AIDS health care services in Indian prisons. Journal of Correctional Health Care. 2009;15(3):179–89.

**Reason for exclusion: off topic**

Guise A, Dimova M, Ndimbii J, Clark P, Rhodes T. A qualitative analysis of transitions to heroin injection in Kenya: Implications for HIV prevention and harm reduction. Harm Reduction Journal. 2015;12 (1) (no pagination)(27).

**Reason for exclusion: method unsuitable**

Gummersbach E, Schmitten JD, Mortsiefer A, Abholz HH, Wegscheider K, Pentzek M. Willingness to Participate in Mammography Screening. Deutsches Arzteblatt International. 2015;112(5):61-U37.

**Reason for exclusion: method unsuitable**

Gunn W, Smedley R, Edwards D, Asthana S, Gleason C. Willingness to be screened for mild cognitive impairment in a community sample of african-americans: Role of stigma and social support. Alzheimer’s and Dementia. 2013;1):P474–5.

**Reason for exclusion: off topic**

Gunnarsson N, Hyden LC. Organizing allergy and being a “good” parent: parents’ narratives about their children’s emerging problems. Health. 2009;13(2):157–74.

**Reason for exclusion: subject matter**

Guo M, Huang J, Ni MY. 3D built-environment attributes and household road traffic noise exposure in Hong Kong. In: IOP Conference Series: Earth and Environmental Science. 2019.

**Reason for exclusion: subject matter**

Gupta E, Agarwala P. Hepatitis E virus infection: An old virus with a new story! Indian Journal of Medical Microbiology. 2018;36(3):317–23.

**Reason for exclusion: method unsuitable**

Gupta GR. The twin stories of Hanumanth Rao and Moses Ezekiel: the impact of reproductive health technologies on individual lives. AIDS. 2001;15 Suppl 1:S83-4.

**Reason for exclusion: off topic**

Gurabardhi Z, Gutteling JM, Kuttschreuter M. The development of risk communication. Science Communication. 2004;25(4):323–49.

**Reason for exclusion: off topic**

Gusdal AK, Obua C, Andualem T, Wahlstrom R, Chalker J, Fochsen G. Peer counselors’ role in supporting patients’ adherence to ART in Ethiopia and Uganda. AIDS Care - Psychological and Socio-Medical Aspects of AIDS/HIV. 2011;23(6):657–62.

**Reason for exclusion: method unsuitable**

Guse K, Spagat A, Hill A, Lira A, Heathcock S, Gilliam M %J AJ of SE. Digital storytelling: A novel methodology for sexual health promotion. 2013;8(4):213–27.

**Reason for exclusion: not primary study**

Guzman L, Lippman L, Moore KA, O’Hare W. Accentuating the Negative: The Mismatch Between Public Perception of Child Well-being and Official Statistics. Child Indicators Research. 2009;2(4):391–416.

**Reason for exclusion: off topic**

Gwandure C. Mubobobo: Women have no sexual fantasies in their sleep. Journal of Sexual Medicine. 2011;3):143.

**Reason for exclusion: subject matter**

Haas RE, Patterson D, Powell S, Eslick R, Cassingham R, Nesley T. Is there postoperative evidence of implicit learning following aural stimuli at moderate hypnotic BIS levels during general anesthesia? AANA journal. 2002;70(3):205–11.

**Reason for exclusion: subject matter**

Haase N, Betsch C, Renkewitz F. Source Credibility and the Biasing Effect of Narrative Information on the Perception of Vaccination Risks. Journal of Health Communication. 2015;20(8):920–9.

**Reason for exclusion: off topic**

Haase TJ, Johnston N. Making Meaning out of Loss: A Story and Study of Young Widowhood. Journal of Creativity in Mental Health. 2012;7(3):204–21.

**Reason for exclusion: subject matter**

Haddow J. Preventing neural tube defects: A major success story, with a chapter yet to be written. Journal of Medical Screening. 1999;6(4):169.

**Reason for exclusion: subject matter**

Haensel JX, Danvers M, Ishikawa M, Itakura S, Tucciarelli R, Smith TJ, et al. Culture modulates face scanning during dyadic social interactions. Scientific Reports. 2020;10(1).

**Reason for exclusion: subject matter**

Hafner Z. Use Measures, Metrics To Tell Your Story Before Someone Else Tells It for You. Managed Care. 2017;26(1):20.

**Reason for exclusion: off topic**

Hafting M, Gullbra F, Anderssen N, Rortveit G, Smith-Sivertsen T, Malterud K. Burdened parents sharing their concerns for their children with the doctor. The impact of trust in general practice: a qualitative study. Scandinavian Journal of Primary Health Care. 2019;37(3):327–34.

**Reason for exclusion: subject matter**

Hagan TL, Cohen SM. A literary analysis of global female identity, health, and equity. Advances in Nursing Science. 2014;37(3):235–48.

**Reason for exclusion: off topic**

Hahn S, Letvak S, Powell K, Christianson C, Wallace D, Speer M, et al. A Community’s Awareness and Perceptions of Genomic Medicine. Public Health Genomics. 2010;13(2):63–71.

**Reason for exclusion: method unsuitable**

Haigh C. “The Times They Are a Changin”: Digital Storytelling as a Catalyst for an Ideological Revolution in Health-Care Research. In: Digital Storytelling in Higher Education. 2017. p. 115–29.

**Reason for exclusion: not primary study**

Haigh N, Haigh F. Facilitating interprofessional learning about human rights in public health contexts: challenges and strategies. Journal of Interprofessional Care. 2007;21(6):605–17.

**Reason for exclusion: off topic**

Haiyasoso M, Trepal H. Survivors’ Stories: Navigating Parenthood After Surviving Child Sexual Abuse. Journal of Counseling and Development. 2019;97(3):281–92.

**Reason for exclusion: subject matter**

Hajdarevic S, Rasmussen BH, Hasle TLO, Ziebland S. Qualitative cross-country comparison of whether, when and how people diagnosed with lung cancer talk about cigarette smoking in narrative interviews. BMJ Open. 2018;8(11).

**Reason for exclusion: method unsuitable**

Hale KL. Family Life and Social Medicine: Discourses and Discontents Surrounding Puebla’s Psychiatric Care. Culture Medicine and Psychiatry. 2017;41(4):499–540.

**Reason for exclusion: off topic**

Haley JF, Bradbury J. Child-headed households under watchful adult eyes: Support or surveillance? Childhood-a Global Journal of Child Research. 2015;22(3):394–408.

**Reason for exclusion: subject**

Hall A, Endfield G. "Snow Scenes’’: Exploring the Role of Memory and Place in Commemorating Extreme Winters. Weather Climate and Society. 2016;8(1):5–19.

**Reason for exclusion: off topic**

Hall J, Jones L, Robertson G, Hiley R, Nathwani D, Perry MR. “The Mould that Changed the World”: a quantitative and qualitative evaluation of knowledge and behavioural change in children in the UK following participation in a musical about antimicrobial resistance. The Lancet. 2019;394 (Supplement 2):S47.

**Reason for exclusion: off topic**

Hall Pistorio K, Brady MP, Morris C. Using literacy-based behavioural interventions to teach self-regulation skills to young children. Early Child Development and Care. 2019;189(10):1682–94.

**Reason for exclusion: subject matter**

Hallett J, Held S, McCormick A, Simonds V, Real Bird S, Martin C, et al. What Touched Your Heart? Collaborative Story Analysis Emerging From an Apsaalooke Cultural Context. Qualitative Health Research. 2017;27(9):1267–77.

**Reason for exclusion: method unsuitable**

Hallett J, Held S, McCormick AKHG, Simonds V, Real Bird S, Martin C, et al. What Touched Your Heart? Collaborative Story Analysis Emerging From an Apsaalooke Cultural Context. Qualitative Health Research. 2017;27(9):1267–77.

**Reason for exclusion: subject matter**

Hallquist T. Narrative inquiry of incarcerated inmate’s perceptions of education and the influence on criminal activity. Dissertation Abstracts International Section A: Humanities and Social Sciences. 2020;81(2–A):No Pagination Specified.

**Reason for exclusion: subject matter**

Halperin DT, de Moya EA, Perez-Then E, Pappas G, Garcia Calleja JM. Understanding the HIV epidemic in the Dominican Republic: a prevention success story in the Caribbean? Journal of Acquired Immune Deficiency Syndromes: JAIDS. 2009;51 Suppl 1:S52-9.

**Reason for exclusion: off topic**

Halpern GM. COX-2 inhibitors: a story of greed, deception and death. Inflammopharmacology. 2005;13(4):419–25.

**Reason for exclusion: subject**

Halter AK. The Power of Storytelling: Digital Stories as a Health Promotion Tool in the Yakima Valley. 2015.

**Reason for exclusion: no short term sacrifice, long term gain**

Hamann C, Pizzinato A, Weber JLA, Rocha KB. Narratives about risk and guilt among patients of a specialized HIV infection service: implications for care in sexual health. Saude e Sociedade. 2017;26(3):651–63.

**Reason for exclusion: off topic**

Hamdallah M, Vargo S, Herrera J. The VOICES/VOCES success story: effective strategies for training, technical assistance and community-based organization implementation. AIDS Education & Prevention. 2006;18(4 Suppl A):171–83.

**Reason for exclusion: not primary study**

Hamela G, Tembo T, Rosenberg NE, Hoffman I, Lee C, Hosseinipour M. Womens experiences of HIV testing and counselling in the labour ward: A case of Bwaila hospital. Malawi Medical Journal. 2013;25(2):36–9.

**Reason for exclusion: off topic**

Hamilton S, Pinfold V, Rose D, Henderson C, Lewis-Holmes E, Flach C, et al. The effect of disclosure of mental illness by interviewers on reports of discrimination experienced by service users: A randomized study. International Review of Psychiatry. 2011;23(1):47–54.

**Reason for exclusion: off topic**

Hammack PL, Toolis EE, Wilson BD, Clark RC, Frost DM. Making meaning of the impact of pre-exposure prophylaxis (PrEP) on public health and sexual culture: Narratives of three generations of gay and bisexual men. Archives of Sexual Behavior. 2019;48(4):1041–58.

**Reason for exclusion: off topic**

Hammer BA, Vallianatos H, Nykiforuk CI, Nieuwendyk LM. Perceptions of healthy eating in four Alberta communities: a photovoice project. Agriculture and Human Values. 2015;32(4):649–62.

**Reason for exclusion: no short term sacrifice, long term gain**

Hammerback K, Hannon PA, Harris JR, Clegg-Thorp C, Kohn M, Parrish A. Perspectives on Workplace Health Promotion Among Employees in Low-Wage Industries. American Journal of Health Promotion. 2015;29(6):384–92.

**Reason for exclusion: off topic**

Hampton S. Policy implementation as practice? Using social practice theory to examine multi-level governance efforts to decarbonise transport in the United Kingdom. Energy Research & Social Science. 2018;38:41–52.

**Reason for exclusion: off topic**

Han CS, Lauby J, Bond L, LaPollo AB, Rutledge SE. Magic Johnson doesn’t worry about how to pay for medicine: experiences of black men who have sex with men living with HIV. Culture Health & Sexuality. 2010;12(4):387–99.

**Reason for exclusion: off topic**

Han CS, Oliffe JL. Photovoice in mental illness research: A review and recommendations. Health. 2016;20(2):110–26.

**Reason for exclusion: not primary study**

Hanrahan C. Critical Social Theory and the Politics of Narrative in the Mental Health Professions: The Mental Health Film Festival as an Emerging Postmodern Praxis. British Journal of Social Work. 2013;43(6):1150–69.

**Reason for exclusion: method unsuitable**

Hansen EC, Nelson MR. Staying a smoker or becoming an ex-smoker after hospitalisation for unstable angina or myocardial infarction. Health. 2017;21(5):461–77.

**Reason for exclusion: off topic**

Hanson E, Magnusson L, Sennemark E. Blended learning networks supported by information and communication technology: an intervention for knowledge transformation within family care of older people. Gerontologist. 2011;51(4):561–70.

**Reason for exclusion: method unsuitable**

Hapeta J, Palmer F, Kuroda Y. Cultural identity, leadership and well-being: how indigenous storytelling contributed to well-being in a New Zealand provincial rugby team. Public Health. 2019;7:7.

**Reason for exclusion: subject**

Haque N, Rosas S. Concept Mapping of Photovoices Sequencing and Integrating Methods to Understand Immigrants’ Perceptions of Neighborhood Influences on Health. Family & Community Health. 2010;33(3):193–206.

**Reason for exclusion: off topic**

Harden J. Parenting a young person with mental health problems: temporal disruption and reconstruction. Sociology of Health & Illness. 2005;27(3):351–71.

**Reason for exclusion: off topic**

Harding C, Fox C. It’s Not About “Freudian Couches and Personality Changing Drugs”: An Investigation Into Men’s Mental Health Help-Seeking Enablers. American Journal of Mens Health. 2015;9(6):451–63.

**Reason for exclusion: off topic**

Harding T, North N, Barton R, Murray E. Lean people ... abundant food: memories of whanau health and food in mid-20th Century everyday life. Health Promotion Journal of Australia. 2011;22(2):142–6.

**Reason for exclusion: off topic**

Hards S. Tales of transformation: The potential of a narrative approach to pro-environmental practices. Geoforum. 2012;43(4):760–71.

**Reason for exclusion: not primary study**

Hardy E, Cu-Uvin S. Care of the HIV-infected pregnant woman in the developed world. Obstetric Medicine. 2015;8(1):13–7.

**Reason for exclusion: not primary study**

Hardy P, SUMNER T %J LJ. Digital Storytelling in health and social care. 2008;3(3):24–31.

**Reason for exclusion: not primary study**

Hardy P, Sumner T. Digital storytelling with users and survivors of the UK mental health system. In: Digital Storytelling. 2017. p. 57–69.

**Reason for exclusion: not primary study**

Harger JRE. AIR-TEMPERATURE VARIATIONS AND ENSO EFFECTS IN INDONESIA, THE PHILIPPINES AND EL-SALVADOR - ENSO PATTERNS AND CHANGES FROM 1866-1993. Atmospheric Environment. 1995;29(16):1919–42.

**Reason for exclusion: off topic**

Harmon BE, Blake CE, Armstead CA, Hebert JR. Intersection of identities. Food, role, and the African-American pastor. Appetite. 2013;67:44–52.

**Reason for exclusion: off topic**

Harocopos A, Goldsamt LA, Kobrak P, Jost JJ, Clatts MC. New injectors and the social context of injection initiation. International Journal of Drug Policy. 2009;20(4):317–23.

**Reason for exclusion: subject matter**

Harper GW, Bangi AK, Contreras R, Pedraza A, Tolliver M, Vess L. Diverse phases of collaboration: working together to improve community-based HIV interventions for adolescents. American Journal of Community Psychology. 2004;33(3–4):193–204.

**Reason for exclusion: off topic**

Harris AM, Hicks LA, Qaseem A, Amer Coll P, Ctr Dis Control P. Appropriate Antibiotic Use for Acute Respiratory Tract Infection in Adults: Advice for High-Value Care From the American College of Physicians and the Centers for Disease Control and Prevention. Annals of Internal Medicine. 2016;164(6):425–+.

**Reason for exclusion: not primary study**

Harris B, Eyles J, Goudge J. Ways of Doing: Restorative Practices, Governmentality, and Provider Conduct in Post-Apartheid Health Care. Medical Anthropology. 2016;35(6):572–87.

**Reason for exclusion: subject matter**

Harris JM. James Edmund Reeves (1829-1896) and the contentious 19th century battle for medical professionalism in the United States. Journal of Medical Biography. 2015;23(3):158–69.

**Reason for exclusion: off topic**

Harris M, Rhodes T, Martin A. Taming systems to create enabling environments for HCV treatment: Negotiating trust in the drug and alcohol setting. Social Science and Medicine. 2013;83:19–26.

**Reason for exclusion: subject matter**

Harris R, Ramaiyer NALNK, Tarawe J. The eBario Story: ICTs for Rural Development. In: Proceeding - 2018 International Conference on ICT for Rural Development: Rural Development through ICT: Concept, Design, and Implication, IC-ICTRuDEv 2018. 2019. p. 63–8.

**Reason for exclusion: off topic**

Harris SB. What works? Success stories in Type 2 diabetes mellitus. Diabetic Medicine. 1998;15 Suppl 4:S20-3.

**Reason for exclusion: not primary study**

Harrison A, Xaba N, Kunene P. Understanding safe sex: gender narratives of HIV and pregnancy prevention by rural South African school-going youth. Reproductive Health Matters. 2001;9(17):63–71.

**Reason for exclusion: method unsuitable**

Hart PS, Nisbet EC. Boomerang Effects in Science Communication: How Motivated Reasoning and Identity Cues Amplify Opinion Polarization About Climate Mitigation Policies. Communication Research. 2012;39(6):701–23.

**Reason for exclusion: off topic**

Harter LM, Ellingson LL, Yamasaki J, Hook C, Walker T. Defining Moments...Telling Stories to Foster Well-being, Humanize Healthcare, and Advocate for Change. Health Communication. 2018;1–6.

**Reason for exclusion: not primary study**

Harter LM, Ellingson LL, Yamasaki J, Hook C, Walker T. Defining Moments…Telling Stories to Foster Well-being, Humanize Healthcare, and Advocate for Change. Health Communication. 2020;35(2):262–7.

**Reason for exclusion: not primary study**

Harter LM, Ellingson LL, Yamasaki J, Hook C, Walker T. Defining moments...telling stories to foster well-being, humanize healthcare, and advocate for change. Health Communication. 2020;35(2):262–7.

**Reason for exclusion: not primary study**

Hartley N, Richardson H. OA11 Public health and hospices: what is really possible? BMJ supportive & palliative care. 2015;5 Suppl 1:A4.

**Reason for exclusion: not primary study**

Hartling L, Scott SD, Johnson DW, Bishop T, Klassen TP. A Randomized Controlled Trial of Storytelling as a Communication Tool. PLoS ONE [Electronic Resource]. 2013;8(10).

**Reason for exclusion: off topic**

Hartwig KA, Kissioki S, Hartwig CD. Church leaders confront HIV/AIDS and stigma: A case study from Tanzania. Journal of Community & Applied Social Psychology. 2006;16(6):492–7.

**Reason for exclusion: off topic**

Harvatt J, Petts J, Chilvers J. Understanding householder responses to natural hazards: flooding and sea-level rise comparisons. Journal of Risk Research. 2011;14(1):63–83.

**Reason for exclusion: off topic**

Harvey J. Design of a comic book intervention for gay male youth at risk for HIV. Journal of Biocommunication. 1997;24(2):16–24.

**Reason for exclusion: method slightly unsuitable**

Hatala AR, Waldram JB, Caal T. Narrative Structures of Maya Mental Disorders. Culture Medicine and Psychiatry. 2015;39(3):449–86.

**Reason for exclusion: off topic**

Hatem D, Ferrara E. Becoming a doctor: fostering humane caregivers through creative writing. Patient Education and Counseling. 2001;45(1):13–22.

**Reason for exclusion: method unsuitable**

Haugen RA. Finance from a new perspective. Financial Management. 1996;25(1):86-.

**Reason for exclusion: method unsuitable**

Hausdorff WP. Prospects for the use of new vaccines in developing countries: cost is not the only impediment. Vaccine. 1996;14(13):1179–86.

**Reason for exclusion: not primary study**

Hawkins AH. Pathography: patient narratives of illness. The Western journal of medicine. 1999;171(2):127–9.

**Reason for exclusion: subject matter**

Hawkins KW, Linvill DL. Public health framing of news regarding childhood obesity in the United States. Health Communication. 2010;25(8):709–17.

**Reason for exclusion: off topic**

Hawthorne TL, Kwan MP. Exploring the unequal landscapes of healthcare accessibility in lower-income urban neighborhoods through qualitative inquiry. Geoforum. 2013;50:97–106.

**Reason for exclusion: method slightly unsuitable**

Haxaire C, Tromeur C, Couturaud F, Leroyer C. A qualitative study to appraise patients and family members perceptions, knowledge, and attitudes towards venous thromboembolism risk. PLoS ONE [Electronic Resource]. 2015;10 (11) (no pagination)(e0142070).

**Reason for exclusion: no short term sacrifice, long term gain**

Hayeems RZ, Moore Hepburn C, Chakraborty P, Odame I, Clarke J, Miller FA, et al. Managing sickle cell carrier results generated through newborn screening in Ontario: A precedent-setting policy story. Genetics in Medicine. 2017;19(6):625–7.

**Reason for exclusion: off topic**

Hazelwood MA, Patterson RM. Scotland’s public health palliative care alliance. Annals of Palliative Medicine. 2018;7(Suppl 2):S99–108.

**Reason for exclusion: subject matter**

Heaton B, Crawford AJ, Wimsatt MA, Henshaw M, Riedy CA, Barker JC, et al. A storytelling intervention reduces childhood caries risk behaviors among American Indian and Alaska Native mothers in Northern California. Journal of Public Health Dentistry. 2019;79(3):183–7.

**Reason for exclusion: no short term sacrifice, long term gain**

Heaton B, Gebel C, Crawford A, Barker JC, Henshaw M, Garcia RI, et al. Using Storytelling to Address Oral Health Knowledge in American Indian and Alaska Native Communities. Preventing Chronic Disease. 2018;15:E63.

**Reason for exclusion: no short term sacrifice, long term gain**

Heilemann MV, Soderlund PD, Kehoe P, Brecht M-L %J J mental health. A transmedia storytelling intervention with interactive elements to benefit latinas’ mental health: feasibility, acceptability, and efficacy. 2017;4(4):e47.

**Reason for exclusion: no short term sacrifice, long term gain**

Hein C. Content of educational programmes for men. Population Education in Asia and the Pacific Newsletter. 1995;(42):27.

**Reason for exclusion: subject matter**

Helle P, Clavagnier I. A patient-expert in patient education. The story of practice. [French]. Revue de L’Infirmiere. 2015;214:21–2.

**Reason for exclusion: ed**

Helle P, Clavagnier I. [A patient-expert in patient education. The story of practice]. Revue de L’Infirmiere. 2015;(214):21–2.

**Reason for exclusion: subject matter**

Helmle KE, Dechant AL, Edwards AL. Implementation of a multidisciplinary educational strategy promoting basal-bolus insulin therapy improves glycemic control and reduces length of stay for inpatients with diabetes. Clinical Diabetes. 2019;37(1):82–5.

**Reason for exclusion: subject matter**

Helzlsouer KJ, Ford DE, Hayward RSA, Midzenski M, Perry H. PERCEIVED RISK OF CANCER AND PRACTICE OF CANCER PREVENTION BEHAVIORS AMONG EMPLOYEES IN AN ONCOLOGY CENTER. Preventive Medicine. 1994;23(3):302–8.

**Reason for exclusion: off topic**

Henckes N. Narratives of change and reform processes: Global and local transactions in French psychiatric hospital reform after the Second World War. Social Science & Medicine. 2009;68(3):511–8.

**Reason for exclusion: off topic**

Henderson NJ. “Top, bottom, versatile”: narratives of sexual practices in gay relationships in the Cape Metropole, South Africa. Culture Health & Sexuality. 2018;20(11):1145–56.

**Reason for exclusion: method unsuitable**

Henderson S, Tanner R, Klanderman N, Mattera A, Webb LM, Steward J. Safe routes to school: A public health practice success story-Atlanta, 2008-2010. Journal of Physical Activity and Health. 2013;10(2):141–2.

**Reason for exclusion: o**

Henderson V, Johnson R, Turino C, Peacock N, Pecha D, Gordon M, et al. Listening to women about what it takes to be a well-woman. Journal of Women’s Health. 2017;26 (4):A22–3.

**Reason for exclusion: subject matter**

Hendersson H, Wamsler C. New stories for a more conscious, sustainable society: claiming authorship of the climate story. Climatic Change. 2020;158(3–4):345–59.

**Reason for exclusion: off topic**

Hendrixson A, Hartmann B. Threats and burdens: Challenging scarcity-driven narratives of “overpopulation.” Geoforum. 2019;101:250–9.

**Reason for exclusion: off topic**

Hengst JA, Duff MC. Clinicians as Communication Partners: Developing a Mediated Discourse Elicitation Protocol. Topics in Language Disorders. 2007;27(1):13–37.

**Reason for exclusion: o**

Hennelly MO, Sly JR, Villagra C, Jandorf L. Narrative message targets within the decision-making process to undergo screening colonoscopy among Latinos: a qualitative study. Journal of Cancer Education. 2015;30(2):268–76.

**Reason for exclusion: off topic**

Henriksen L, Flora JA, Feighery E, Fortmann SP. Effects on youth of exposure to retail tobacco advertising. Journal of Applied Social Psychology. 2002;32(9):1771–89.

**Reason for exclusion: off topic**

Henry CA. Using mhealth to promote hand washing with soap: How do Tanzanian youth perceive text message interventions for hand hygiene? American Journal of Tropical Medicine and Hygiene. 2017;97 (5 Supplement 1):280.

**Reason for exclusion: off topic**

Henschen JAN. Die filmprimadonna (The film primadonna, 1913): A case study of the fiction of a screenplay and the process of filmmaking in german early cinema. Journal of Screenwriting. 2019;10(3):247–59.

**Reason for exclusion: subject matter**

Henshaw A. Pausing along the journey: Learning landscapes, environmental change, and toponymy amongst the Sikusilarmiut. Arctic Anthropology. 2006;43(1):52–66.

**Reason for exclusion: off topic**

Herasme L, Bello A, Moreno L, Moya M, Rosario S. Country watch: Dominican Republic. AIDS Health Promotion Exchange. 1992;(1):9–11.

**Reason for exclusion: subject matter**

Herbert MR. Genetics finding its place in larger living schemes. Critical Public Health. 2002;12(3):221–36.

**Reason for exclusion: subject matter**

Herbert S, Stephens C, Forster M. Socially based trajectories of alcohol use among indigenous Maori in Aotearoa/New Zealand. Critical Public Health. 2018;28(5):596–605.

**Reason for exclusion: off topic**

Herbrand C, Dimond R. Mitochondrial donation, patient engagement and narratives of hope. Sociology of Health & Illness. 2018;40(4):623–38.

**Reason for exclusion: subject matter**

Hercberg S. The history of beta-carotene and cancers: from observational to intervention studies. What lessons can be drawn for future research on polyphenols? American Journal of Clinical Nutrition. 2005;81(1 Suppl):218S–222S.

**Reason for exclusion: not primary study**

Herdt G. Intimate Consumption and New Sexual Subjects Among the Sambia of Papua New Guinea. Oceania. 2019;89(1):36–67.

**Reason for exclusion: subject matter**

Hermansen M. On education and pedagogic development at NHV. Scandinavian Journal of Public Health. 2015;43(16 Supplement):18–20.

**Reason for exclusion: subject matter**

Hernandez SG, Genkova A, Castaneda Y, Alexander S, Hebert-Beirne J. Oral Histories as Critical Qualitative Inquiry in Community Health Assessment. Health Education & Behavior. 2017;44(5):705–15.

**Reason for exclusion: subject matter**

Herrmann S, McKinnon E, John M, Hyland N, Martinez OP, Cain A, et al. Evidence-based, multifactorial approach to addressing non-adherence to antiretroviral therapy and improving standards of care. Internal Medicine Journal. 2008;38(1):8–15.

**Reason for exclusion: off topic**

Hesthammer J, Stefatos A, Boulaenko M, Vereshagin A, Gelting P, Wedberg T, et al. CSEM technology as a value driver for hydrocarbon exploration. Marine and Petroleum Geology. 2010;27(9):1872–84.

**Reason for exclusion: subject matter**

Hey A. History and Practice: Antibodies in Infectious Diseases. Microbiology Spectrum. 2015;3(2):AID-0026-2014.

**Reason for exclusion: off topic**

Heyerdahl LW, Pugliese-Garcia M, Nkwemu S, Tembo T, Mwamba C, Demolis R, et al. “It depends how one understands it:” A qualitative study on differential uptake of oral cholera vaccine in three compounds in Lusaka, Zambia. BMC Infectious Diseases. 2019;19 (1) (no pagination)(421).

**Reason for exclusion: method unsuitable**

Hickling FW, Robertson-Hickling H, Paisley V. Deinstitutionalization and attitudes toward mental illness in Jamaica: a qualitative study. Revista Panamericana De Salud Publica-Pan American Journal of Public Health. 2011;29(3):169–76.

**Reason for exclusion: off topic**

Highet NJ, Hickie IB, Davenport TA. Monitoring awareness of and attitudes to depression in Australia. Medical Journal of Australia. 2002;176:S63–8.

**Reason for exclusion: off topic**

Hild-Mosley KA, Patel DM, Markwell S, Massad LS. Knowledge of Cervical Cancer Screening, Human Papillomavirus, and HPV Vaccine Among Midwestern Gynecology Patients. Journal of Lower Genital Tract Disease. 2009;13(4):200–6.

**Reason for exclusion: off topic**

Hill PS, Murphy GJ. CULTURAL IDENTIFICATION IN ABORIGINAL AND TORRES STRAIT ISLANDER AIDS EDUCATION. Australian Journal of Public Health. 1992;16(2):150–7.

**Reason for exclusion: not primary study**

Hillman A, Lewis J, Elwyn G. Pathways and prospects in cancer research: Securing futures and negotiating boundaries. Biosocieties. 2017;12(3):321–42.

**Reason for exclusion: off topic**

Hinchcliff R, Greenfield D, Moldovan M, Westbrook JI, Pawsey M, Mumford V, et al. Narrative synthesis of health service accreditation literature. BMJ Quality & Safety. 2012;21(12):979–91.

**Reason for exclusion: subject matter**

Hinchliffe S, Ward KJ. Geographies of folded life: How immunity reframes biosecurity. Geoforum. 2014;53:136–44.

**Reason for exclusion: subject matter**

Hinnant A, Subramanian R, Young R. User comments on climate stories: impacts of anecdotal vs. scientific evidence. Climatic Change. 2016;138(3–4):411–24.

**Reason for exclusion: off topic**

Hinyard LJ, Kreuter MW. Using narrative communication as a tool for health behavior change: A conceptual, theoretical, and empirical overview. Health Education & Behavior. 2007;34(5):777–92.

**Reason for exclusion: not primary study**

Ho QV, Lundblad P, Astrom T, Jern M. A web-enabled visualization toolkit for geovisual analytics. Information Visualization. 2012;11(1):22–42.

**Reason for exclusion: subject matter**

Ho Y-YC, Coady MR. English as a Second Language Nurses in the United States: Culture, Communication, and Needs for Continuing Education. Studies in Continuing Education. 2018;40(2):212–33.

**Reason for exclusion: off topic**

Hoddinott P, Craig LCA, Britten J, McInnes RM. A serial qualitative interview study of infant feeding experiences: idealism meets realism. BMJ Open. 2012;2(2).

**Reason for exclusion: off topic**

Hoeken H, Sinkeldam J. The Role of Identification and Perception of Just Outcome in Evoking Emotions in Narrative Persuasion. Journal of Communication. 2014;64(5):935–55.

**Reason for exclusion: off topic**

Hoelscher DM, Springer A, Menendez TH, Cribb PW, Kelder SH. From NIH to Texas schools: policy impact of the Coordinated Approach to Child Health (CATCH) program in Texas. Journal of Physical Activity & Health. 2011;8 Suppl 1:S5-7.

**Reason for exclusion: subject matter**

Hoewe J, Ahern L. First-Person Effects of Emotional and Informational Messages in Strategic Environmental Communications Campaigns. Environmental Communication-a Journal of Nature and Culture. 2017;11(6):810–20.

**Reason for exclusion: off topic**

Hofmann GE, Smith JE, Johnson KS, Send U, Levin LA, Micheli F, et al. High-Frequency Dynamics of Ocean pH: A Multi-Ecosystem Comparison. PLoS ONE [Electronic Resource]. 2011;6(12).

**Reason for exclusion: subject matter**

Hogenraad R, McKenzie DP, Peladeau N. Force and influence in content analysis: The production of new social knowledge. Quality & Quantity. 2003;37(3):221–38.

**Reason for exclusion: off topic**

Hoggart L, Newton VL, Bury L. “Repeat abortion”, a phrase to be avoided? Qualitative insights into labelling and stigma. Journal of Family Planning and Reproductive Health Care. 2017;43(1):26–30.

**Reason for exclusion: off topic**

Hok J, Wachtler C, Falkenberg T, Tishelman C. Using narrative analysis to understand the combined use of complementary therapies and bio-medically oriented health care. Social Science & Medicine. 2007;65(8):1642–53.

**Reason for exclusion: subject matter**

Holbrook M. Bridging the gap from current practice towards wider application of mechanistic approaches. Toxicology Letters. 2017;280 (Supplement 1):S24.

**Reason for exclusion: off topic**

Holden S, Ferguson M, Brimblecombe J, Palermo CE. Can a community of practice equip public health nutritionists to work with remote retail to improve the food supply? Rural & Remote Health. 2015;15(4):3464.

**Reason for exclusion: off topic**

Holland GN. LX Edward Jackson memorial lecture - Ocular toxoplasmosis: A global reassessment. Part 1: Epidemiology and course of disease. American Journal of Ophthalmology. 2003;136(6):973–88.

**Reason for exclusion: subject matter**

Holliday HV, Jenstad LM, Grosjean G, Purves B. “You Can Lead a Horse to Water ...”: Focus Group Perspectives on Initiating and Supporting Hearing Health Change in Older Adults. American Journal of Audiology. 2015;24(3):360–76.

**Reason for exclusion: subject matter**

Holman A, Kellas JK. “Say something instead of nothing”: Adolescents’ perceptions of memorable conversations about sex-related topics with their parents. Communication Monographs. 2018;85(3):357–79.

**Reason for exclusion: subject matter**

Holmes A, Edelstein T. Pressure ulcer success story. Provider. 2007;33(12):37–9, 41.

**Reason for exclusion: off topic**

Holzer C, Warshaw G. Clues to early Alzheimer dementia in the outpatient setting. Archives of Family Medicine. 2000;9(10):1066–70.

**Reason for exclusion: off topic**

Honigh-de Vlaming R, Haveman-Nies A, Ziylan C, Renes RJ. Acceptability of the Components of a Loneliness Intervention among Elderly Dutch People: A Qualitative Study. American Journal of Health Education. 2013;44(3):136–45.

**Reason for exclusion: subject matter**

Hooper CM, Ivory VC, Fougere G. “Dinner’s ready!” A qualitative exploration of the food domain across the lifecourse. Appetite. 2015;92:133–42.

**Reason for exclusion: off topic**

Hope T. QALYs, lotteries and veils: the story so far. Journal of Medical Ethics. 1996;22(4):195–6.

**Reason for exclusion: off topic**

Hopfer S. Effects of a Narrative HPV Vaccination Intervention Aimed at Reaching College Women: A Randomized Controlled Trial. Prevention Science. 2012;13(2):173–82.

**Reason for exclusion: off topic**

Hopper K. When (working) in Rome: Applying anthropology in Caesar’s realm. Human Organization. 2002;61(3):196–209.

**Reason for exclusion: off topic**

Horky S, Andreola J, Black E, Lossius M. Evaluation of a Cross Cultural Curriculum: Changing Knowledge, Attitudes and Skills in Pediatric Residents. Maternal & Child Health Journal. 2017;21(7):1537–43.

**Reason for exclusion: subject matter**

Horn K, McCracken L, Dino G, Brayboy M. Applying community-based participatory research principles to the development of a smoking-cessation program for American Indian teens: “telling our story.” Health Education & Behavior. 2008;35(1):44–69.

**Reason for exclusion: off topic**

Horner SD. Intervening to improve family and child asthma self-management. American Journal of Respiratory and Critical Care Medicine Conference: American Thoracic Society International Conference, ATS. 2018;197(MeetingAbstracts).

**Reason for exclusion: off topic**

Horridge GA, Loughuethiggins HC, Horridge GA. WHAT CAN ENGINEERS LEARN FROM INSECT VISION. Philosophical Transactions of the Royal Society of London Series B-Biological Sciences. 1992;337(1281):271–82.

**Reason for exclusion: subject matter**

Horsfall D, Leonard R, Rosenberg JP, Noonan K. Home as a place of caring and wellbeing? A qualitative study of informal carers and caring networks lived experiences of providing in-home end-of-life care. Health & Place. 2017;46:58–64.

**Reason for exclusion: subject matter**

Hossain MS, Gresock J, Edmonds Y, Helm R, Potts M, Ramakrishnan N. Connecting the Dots between PubMed Abstracts. PLoS ONE [Electronic Resource]. 2012;7(1).

**Reason for exclusion: subject matter**

Hostetler BR, Rosenthal T, Muram D, Crom D. And now for the rest of the story. Journal of the Tennessee Medical Association. 1993;86(1):12.

**Reason for exclusion: off topic**

Hou SI, Fernandez ME, Parcel GS. Development of a cervical cancer educational program for Chinese women using intervention mapping. Health Promotion Practice. 2004;5(1):80–7.

**Reason for exclusion: off topic**

Houet T, Gremont M, Vacquie L, Forget Y, Marriotti A, Puissant A, et al. Downscaling scenarios of future land use and land cover changes using a participatory approach: an application to mountain risk assessment in the Pyrenees (France). Regional Environmental Change. 2017;17(8):2293–307.

**Reason for exclusion: off topic**

Houston TK, Allison JJ, Sussman M, Horn W, Holt CL, Trobaugh J, et al. Culturally appropriate storytelling to improve blood pressure: a randomized trial. Annals of Internal Medicine. 2011;154(2):77–84.

**Reason for exclusion: no short term sacrifice, long term gain**

Houston TK, Fix GM, Shimada SL, Long JA, Gordon HS, Pope C, et al. African American Veterans Storytelling. Medical Care. 2017;55(9 Supplement 2):S50–8.

**Reason for exclusion: subject matter**

Houston TK, Fix GM, Shimada SL, Long JA, Gordon HS, Pope C, et al. African American Veterans Storytelling: A Multisite Randomized Trial to Improve Hypertension. Medical Care. 2017;55 Suppl 9 Suppl 2:S50–8.

**Reason for exclusion: no short term sacrifice, long term gain**

Howarth C. Informing decision making on climate change and low carbon futures: Framing narratives around the United Kingdom’s fifth carbon budget. Energy Research & Social Science. 2017;31:295–302.

**Reason for exclusion: not primary study**

Howe PD, Boudet H, Leiserowitz A, Maibach EW. Mapping the shadow of experience of extreme weather events. Climatic Change. 2014;127(2):381–9.

**Reason for exclusion: off topic**

Howe PD, Leiserowitz A. Who remembers a hot summer or a cold winter? The asymmetric effect of beliefs about global warming on perceptions of local climate conditions in the US. Global Environmental Change-Human and Policy Dimensions. 2013;23(6):1488–500.

**Reason for exclusion: off topic**

Howe PD, Markowitz EM, Lee TM, Ko CY, Leiserowitz A. Global perceptions of local temperature change. Nature Climate Change. 2013;3(4):352–6.

**Reason for exclusion: off topic**

Howe PD, Mildenberger M, Marlon JR, Leiserowitz A. Geographic variation in opinions on climate change at state and local scales in the USA. Nature Climate Change. 2015;5(6):596–603.

**Reason for exclusion: off topic**

Howe PD. Perceptions of seasonal weather are linked to beliefs about global climate change: evidence from Norway. Climatic Change. 2018;148(4):467–80.

**Reason for exclusion: off topic**

Howell RA. It’s not (just) “the environment, stupid!” Values, motivations, and routes to engagement of people adopting lower-carbon lifestyles. Global Environmental Change-Human and Policy Dimensions. 2013;23(1):281–90.

**Reason for exclusion: off topic**

Howse E, Hankey C, Allman-Farinelli M, Bauman A, Freeman B. "Buying Salad Is a Lot More Expensive than Going to McDonalds’: Young Adults’ Views about What Influences Their Food Choices. Nutrients. 2018;10(8).

**Reason for exclusion: off topic**

Hoybye MT, Johansen C, Tjornhoj-Thomsen T. Online interaction. Effects of storytelling in an internet breast cancer support group. Psycho-Oncology. 2005;14(3):211–20.

**Reason for exclusion: no short term sacrifice, long term gain**

Hoyos-Hernandez PA, Mazo JPS, Pineda LTO, Gallego ALV, Ceballos MG, Munoz TO. Social representations associated with HIV/AIDS in Colombian university students’. Saude e Sociedade. 2019;28(2):227–38.

**Reason for exclusion: method unsuitable**

Hrabcik H. [Vaccination--the big success story]. Wiener Medizinische Wochenschrift. 2007;157(5–6):93.

**Reason for exclusion: not primary study**

Hristov N, Strohecker C, Allen L, Merson M. Designing for Broad Understanding of Science Insights from Practice. Integrative and Comparative Biology. 2018;58(1):113–26.

**Reason for exclusion: off topic**

Hrnjak I, Lukic T, Gavrilov MB, Markovic SB, Unkasevic M, Tosic I. Aridity in Vojvodina, Serbia. Theoretical and Applied Climatology. 2014;115(1–2):323–32.

**Reason for exclusion: subject matter**

Hsieh C, Arenson CA, Eanes K, Sifri RD. Reflections of medical students regarding the care of geriatric patients in the continuing care retirement community. Journal of the American Medical Directors Association. 2010;11(7):506–10.

**Reason for exclusion: subject matter**

Hu R. Examining Social Service Providers’ Representation of Trafficking Victims: A Feminist Postcolonial Lens. Affilia - Journal of Women and Social Work. 2019;34(4):421–38.

**Reason for exclusion: subject matter**

Hua Y, Yang E. Building spatial layout that supports healthier behavior of office workers: A new performance mandate for sustainable buildings. Work-a Journal of Prevention Assessment & Rehabilitation. 2014;49(3):373–80.

**Reason for exclusion: subject**

Huang CHS, Rocque G, Niranjan S, Dionne-Odom J, Taylor R, Halilova K, et al. “Our life is a story, and we all want a good ending to the story”: Navigators on advance care planning conversations in older patients with cancer. Journal of Pain and Symptom Management. 2017;53 (2):368–9.

**Reason for exclusion: off topic**

Huang F, Blaschke S, Lucas H. Beyond pilotitis: taking digital health interventions to the national level in China and Uganda. Global Health. 2017;13(1):49.

**Reason for exclusion: off topic**

Hubley J. Use effective communication channels. Health education. AIDS Action. 1988;(2):5.

**Reason for exclusion: off topic**

Huby G, Guthrie B, Grant S, Watkins F, Checkland K, McDonald R, et al. Whither British general practice after the 2004 GMS contract? Stories and realities of change in four UK general practices. Journal of Health Organization & Management. 2008;22(1):63–78.

**Reason for exclusion: subject matter**

Hughes D, Leethongdee S, Osiri S. Using economic levers to change behaviour: the case of Thailand’s universal coverage health care reforms. Social Science & Medicine. 2010;70(3):447–54.

**Reason for exclusion: subject matter**

Hughes SC, Usita PM, Hovell MF, Hofstetter CR. Reactions to Secondhand Smoke by Nonsmokers of Korean Descent: Clash of Cultures? Journal of Immigrant and Minority Health. 2011;13(4):766–71.

**Reason for exclusion: subject**

Hume J, Bressers B. Obituaries online: new connections with the living--and the dead. Omega - Journal of Death & Dying. 2009;60(3):255–71.

**Reason for exclusion: subject matter**

Hurst CG. Encouraging and discouraging breastfeeding: Relational odysseys concerning mothers’ milk. Breastfeeding Medicine. 2009;4 (4):246.

**Reason for exclusion: subject matter**

Husmann MA. Social constructions of obesity target population: an empirical look at obesity policy narratives. Policy Sciences. 2015;48(4):415–42.

**Reason for exclusion: off topic**

Hussain T, McCartney DG, Shipway PH. Bonding between aluminium and copper in cold spraying: story of asymmetry. Materials Science and Technology. 2012;28(12):1371–8.

**Reason for exclusion: subject**

Hussein ZM, Dehham SH, Hasan AAN. The impact of using chunking technique on developing reading skill and perception of intermediate school students. Indian Journal of Public Health Research and Development. 2019;10(6):1186–9.

**Reason for exclusion: subject matter**

Hussey W %J J of homosexuality. Slivers of the journey: the use of Photovoice and storytelling to examine female to male transsexuals’ experience of health care access. 2006;51(1):129–58.

**Reason for exclusion: no short term sacrifice, long term gain**

Hussey W. Slivers of the journey: The use of photovoice and storytelling to examine female to male transsexuals’ experiences of health care. In: The 130th Annual Meeting of APHA. 2002.

**Reason for exclusion: can't locate study**

Hutcheon DA. Malnutrition-Induced Wernicke’s Encephalopathy Following a Water-Only Fasting Diet. Nutrition in Clinical Practice. 2015;30(1):92–9.

**Reason for exclusion: subject**

Hutchinson E, Nayiga S, Nabirye C, Taaka L, Staedke SG. Data value and care value in the practice of health systems: A case study in Uganda. Social Science & Medicine. 2018;211:123–30.

**Reason for exclusion: off topic**

Hwang KO, Trickey AW, Graham AL, Thomas EJ, Street RL, Kraschnewski JL, et al. Acceptability of narratives to promote colorectal cancer screening in an online community. Preventive Medicine. 2012;54(6):405–7.

**Reason for exclusion: method unsuitable**

Hwang KO, Trickey AW, Graham AL, Thomas EJ, Street RL, Vernon SW. Feasibility and acceptability of promoting colorectal cancer screening in an Internet weight loss community through narratives and navigational guidance. Cancer Prevention Research Conference: AACR International Conference on Frontiers in Cancer Prevention Research Philadelphia, PA United States Conference Publication: 2010;3(12 SUPPL. 2).

**Reason for exclusion: abstract only**

Hyde A, Carney M, Drennan J, Butler M, Lohan M, Howlett E. The silent treatment: parents’ narratives of sexuality education with young people. Culture, Health & Sexuality. 2010;12(4):359–71.

**Reason for exclusion: subject**

Hyle EP, Jani IV, Lehe J, Su AE, Wood R, Quevedo J, et al. The Clinical and Economic Impact of Point-of-Care CD4 Testing in Mozambique and Other Resource-Limited Settings: A Cost-Effectiveness Analysis. Plos Medicine. 2014;11(9).

**Reason for exclusion: subject**

Hébert Chatelain É, Breton S, Lemieux H, Blier PU. Epitoky in Nereis (Neanthes) virens (Polychaeta: Nereididae): A story about sex and death. Comparative Biochemistry and Physiology - B Biochemistry and Molecular Biology. 2008;149(1):202–8.

**Reason for exclusion: off topic**

Illes J. A fish story? Brain maps, lie detection, and personhood. Cerebrum. 2004;6(4):73–80.

**Reason for exclusion: subject matter**

Imschloss M, Lorenz J. How mobile app design impacts user responses to mixed self-tracking outcomes: Randomized online experiment to explore the role of spatial distance for hedonic editing. JMIR MHealth and UHealth. 2018;6(4).

**Reason for exclusion: subject matter**

Indirli M, Razafindrakoto H, Romanelli F, Puglisi C, Lanzoni L, Milani E, et al. Hazard Evaluation in Valparaiso: the MAR VASTO Project. Pure and Applied Geophysics. 2011;168(3–4):543–82.

**Reason for exclusion: subject**

Ingram J, Mills J, Dibari C, Ferrise R, Ghaley BB, Hansen JG, et al. Communicating soil carbon science to farmers: Incorporating credibility, salience and legitimacy. Journal of Rural Studies. 2016;48:115–28.

**Reason for exclusion: off topic**

Inhorn MC, Janes CR. The behavioural research agenda in global health: an advocate’s legacy. Global Public Health. 2007;2(3):294–312.

**Reason for exclusion: subject matter**

Inskip H, Ntani G, Westbury L, Di Gravio C, D’Angelo S, Parsons C, et al. Getting started with tables. Archives of Public Health. 2017;75:14.

**Reason for exclusion: subject matter**

Iredale R, Mundy L, Hilgart J. An online resource of digital stories about cancer genetics: qualitative study of patient preferences and information needs. Journal of Medical Internet Research. 2011;13(3):e78.

**Reason for exclusion: not primary study**

Isaac R, Biswajit P, Olver I, Finkel M, Trevena L. The effect of a voice-site on informed choice for women considering cervical cancer screening in rural India: A phase II controlled trial. Asia-Pacific Journal of Clinical Oncology. 2014;9):18.

**Reason for exclusion: method slightly unsuitable**

Isaacs D, Oates K. How to give a better lecture. Journal of Paediatrics and Child Health. 2018;54(12):1290–1.
[truncated: 385,248 more chars]
